# Dataset of chloroplast intergenic spacer sequences and candidate DNA markers for species identification in *Hoya* (Apocynaceae) based on the plastome of *Hoya lockii* V.T. Pham & Aver. from Vietnam

**DOI:** 10.1016/j.dib.2026.112757

**Published:** 2026-04-09

**Authors:** Manivanh Yongsa, Nga Thi Thu Nguyen, Lan Thi Ngoc Nguyen, Thuong Danh Sy, Tan Quang Tu, Mau Hoang Chu

**Affiliations:** Faculty of Biology, Thainguyen University of Education, Thainguyen 23000, Vietnam

**Keywords:** Hoya lockii, Chloroplast genome, Intergenic spacer sequences, Phylogenetic analysis, Candidate molecular markers

## Abstract

*Hoya lockii* V.T. Pham & Aver. is an epiphytic species that exhibits strong morphological similarity to several closely related congeners, which may hinder accurate species identification, especially when specimens are incomplete or degraded. This article presents a curated dataset of chloroplast intergenic spacer sequences to support molecular identification and phylogenetic assessment of *H. lockii*. The dataset includes six plastid intergenic spacer regions (*trnK–rps16, psbI–atpA, trnH–psbA, psbK–psbI, ndhC–trnV,* and *rbcL–accD*) extracted from complete chloroplast genomes. Phylogenetic analyses based on individual loci revealed variation in topological resolution and bootstrap support among regions. Among them, the markers *psbI–atpA* and *ndhC–trnV* showed relatively strong phylogenetic signals. In the *ndhC-trnV* tree, *H. lockii* formed a well-supported clade with *Hoya exilis* (bootstrap = 99%). In the *psbI–atpA* tree, *H. lockii* was recovered as the sister of *Hoya lanceolata*, with strong bootstrap support (99%). Multilocus phylogenetic reconstruction based on concatenated alignments of six intergenic spacers further improved tree stability and resolution, recovering *H. lockii* as the sister taxon of *H. exilis* with strong bootstrap support (100%). These results indicate that *psbK–psbI, psbI–atpA*, and *ndhC–trnV*, together with the concatenated dataset, represent potential DNA marker candidates to support species identification within the genus *Hoya*. The dataset provides a reproducible molecular resource that may facilitate phylogenetic analysis, DNA barcoding, and taxonomic studies of *Hoya* and related taxa in Apocynaceae.

Specifications Table

**Table:** 

Subject	Biological Sciences
Specific subject area	Biotechnology, Genetics diversity, Molecular Phylogenetics, Molecular Evolution
Type of data	Raw, sequence data, tables, figures, text files
Data collection	Illumina NovaSeq 6000 (Illumina) sequencer
Data source location	GenBank: OR475243.1 NCBI Reference Sequence: NC_085235.1
Data accessibility	Repository name: NCBI Data identification number: Not applicable Direct URL to data: The NCBI links to the sequenced data can be accessed at: https://www.ncbi.nlm.nih.gov/nuccore/OR475243.1 https://www.ncbi.nlm.nih.gov/nuccore/NC_085235.1

## 1 Value of the Data

• This dataset provides curated chloroplast intergenic spacer sequences (trnK–rps16, psbI–atpA, trnH–psbA, psbK–psbI, ndhC–trnV, and rbcL–accD) from Hoya lockii, offering valuable molecular resources for species identification and marker development, particularly when morphological traits are ambiguous or specimens are incomplete or degraded.
• The included loci exhibit variable nucleotide diversity and locus-dependent phylogenetic resolution, supplying complementary phylogenetic signals that facilitate the evaluation of interspecific relationships and support multilocus-based discrimination within the genus Hoya.
• The concatenated multilocus dataset provides enhanced phylogenetic resolution and serves as a robust molecular reference for H. lockii, supporting future studies in DNA barcoding, taxonomy, and comparative plastid genomics within the family Apocynaceae.

## 2 Background

*Hoya lockii* V.T. Pham & Aver., a member of the family Apocynaceae [1], is one of the few *Hoya* species endemic to Vietnam. It is a humus-associated epiphytic undershrub that occurs mainly in wet tropical habitats [2]. Several species of the genus *Hoya* have been reported to contain biologically active compounds with potential therapeutic value [3], which increases the need for reliable species identification within this group. However, *H. lockii* is morphologically very similar to several congeners, often making accurate identification difficult, especially when specimens are incomplete or degraded.

Chloroplast DNA is a circular, double-stranded molecule with region-specific mutation rates [4], making it suitable for inferring phylogenetic relationships among plant taxa based on patterns of sequence divergence [5]. Highly variable chloroplast regions are therefore widely used in phylogenetic reconstruction and DNA barcoding studies. The complete chloroplast genome of *H. lockii* has recently been sequenced and deposited in GenBank under the accession number OR475243.1 [6].

Previous studies have demonstrated that chloroplast genomes are relatively conserved but contain highly variable hotspots, primarily located in non-coding regions. Several [7], and chloroplast intergenic spacer regions, including *trnK–rps16, rps16–trnQ, psbI–atpA, trnH–trnF, trnH–psbA, ndhC–trnV,* and *rbcL–accD*, are effective DNA markers for species discrimination and phylogenetic analysis within the genus *Hoya* [3,8,9]. However, their performance has not yet been evaluated for *H. lockii*, an endemic species for which molecular data remain limited. This work therefore provides a dataset of chloroplast intergenic spacer sequences and documents their potential utility as molecular markers for the identification and phylogenetic placement of *H. lockii*.

## 3 Data Description

**Fig. 1:**
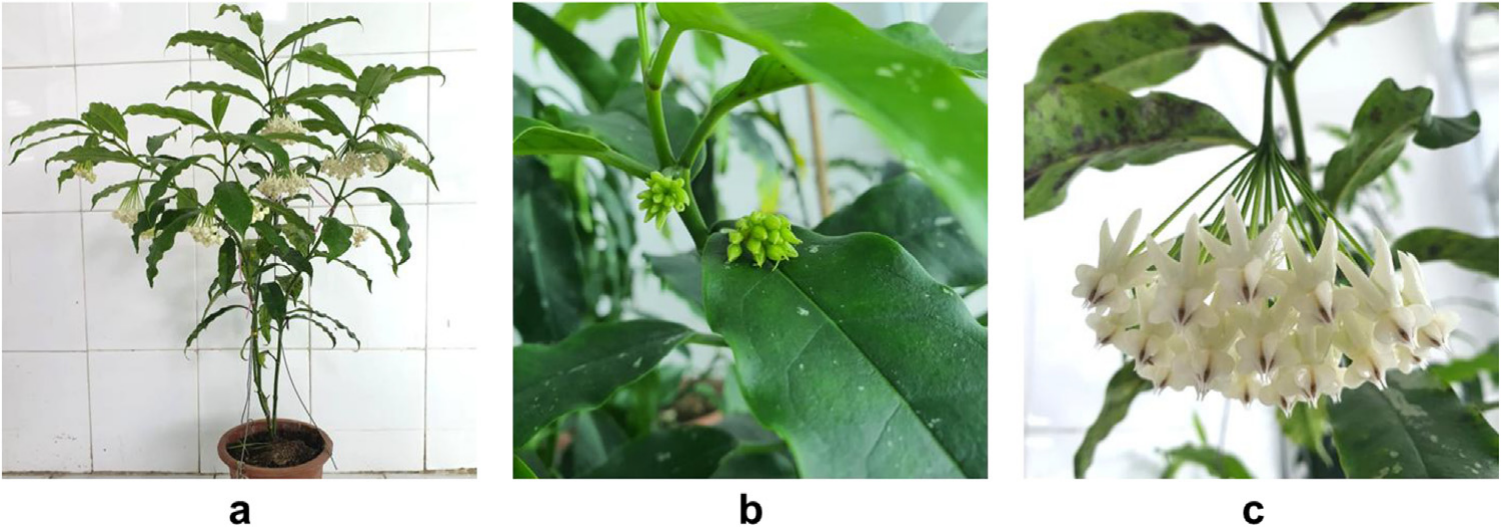
Hoya lockii V.T. Pham & Aver. were taken by Nga Thi Thu Nguyen at the Faculty of Biology experimental garden at Thai Nguyen University of Education, Viet Nam (21° 35′ 55.0921′’ N, 105° 49′ 25.5569′’). (a) Whole plant bearing multiple flowers; (b) inflorescence with a flower bud; (c) fully developed flowers. A specimen was deposited at the Faculty of Biology, Thainguyen University of Education, Vietnam (Nguyen, T.T.N.; ngantt.bio@tnue.edu.vn) under voucher number HL2022-VN01 (Fig. 1).

This dataset comprises chloroplast intergenic spacer sequences of *H. lockii*, extracted from the complete chloroplast genome deposited in GenBank under accession number OR475243.1 [6]. Plant material was collected in September 2022 from Dong Son–Ky Thuong Nature Reserve, Hoanh Bo District, Quang Ninh Province, Vietnam, and subsequently cultivated in the experimental garden of Thainguyen University of Education. The morphological characteristics of *H. lockii* are presented in Fig. 1.

**Fig. 2:**
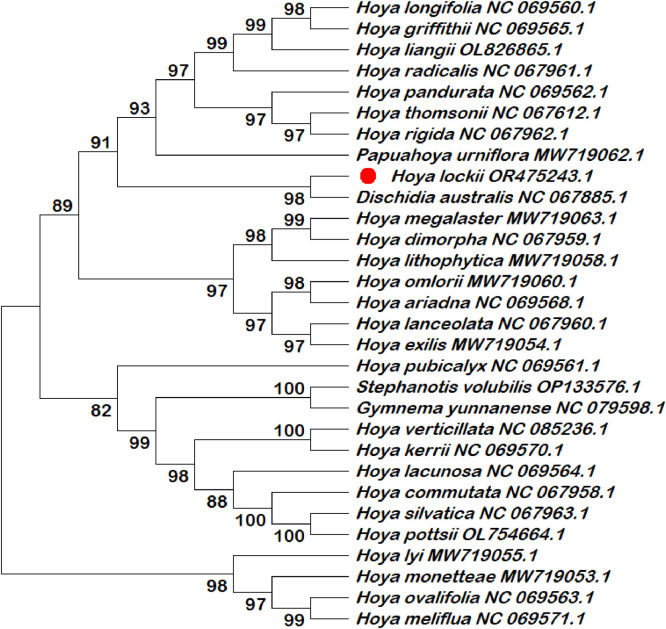
Construction of a phylogenetic tree based on the *trnK-rps16* intergenic spacer region of the chloroplast genome of *Hoya lockii* V.T. Pham & Aver. and other species within the Apocynaceae family.

**Fig. 3:**
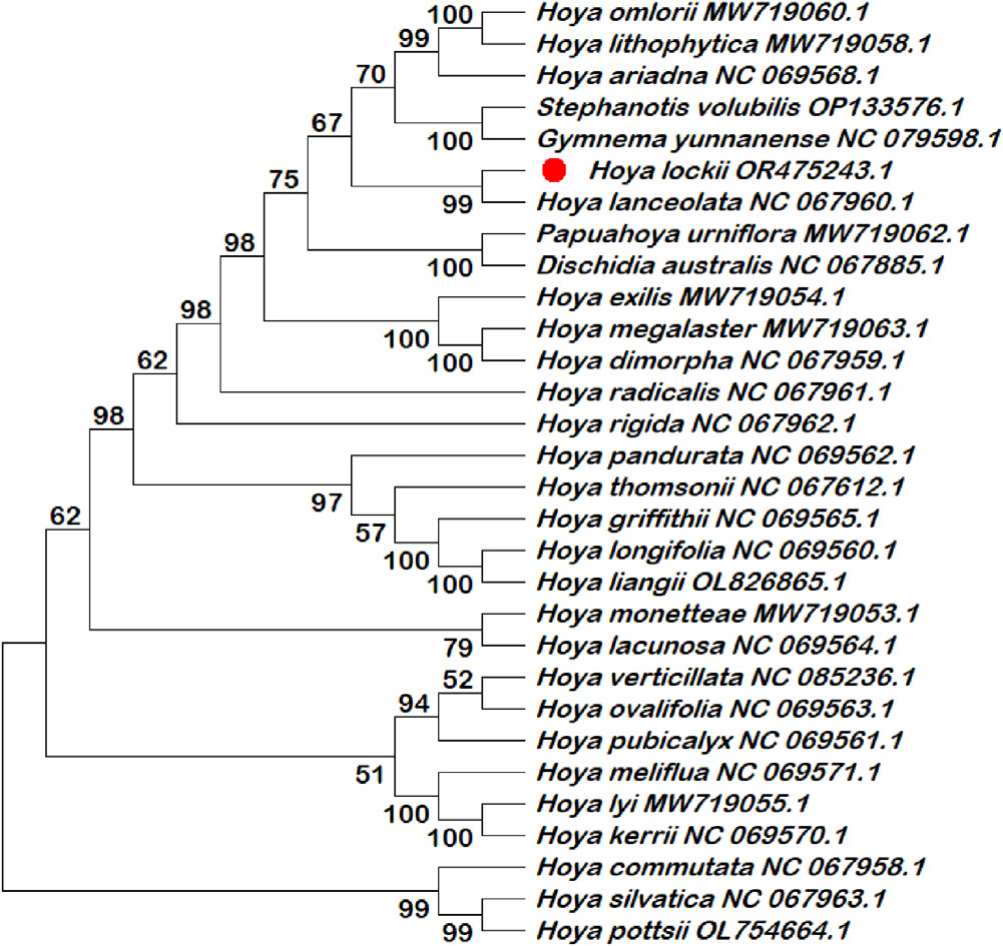
Construction of a phylogenetic tree based on the *psbI-atpA* intergenic spacer region of the chloroplast genome of *Hoya lockii* V.T. Pham & Aver. and other species within the Apocynaceae family.

**Fig. 4:**
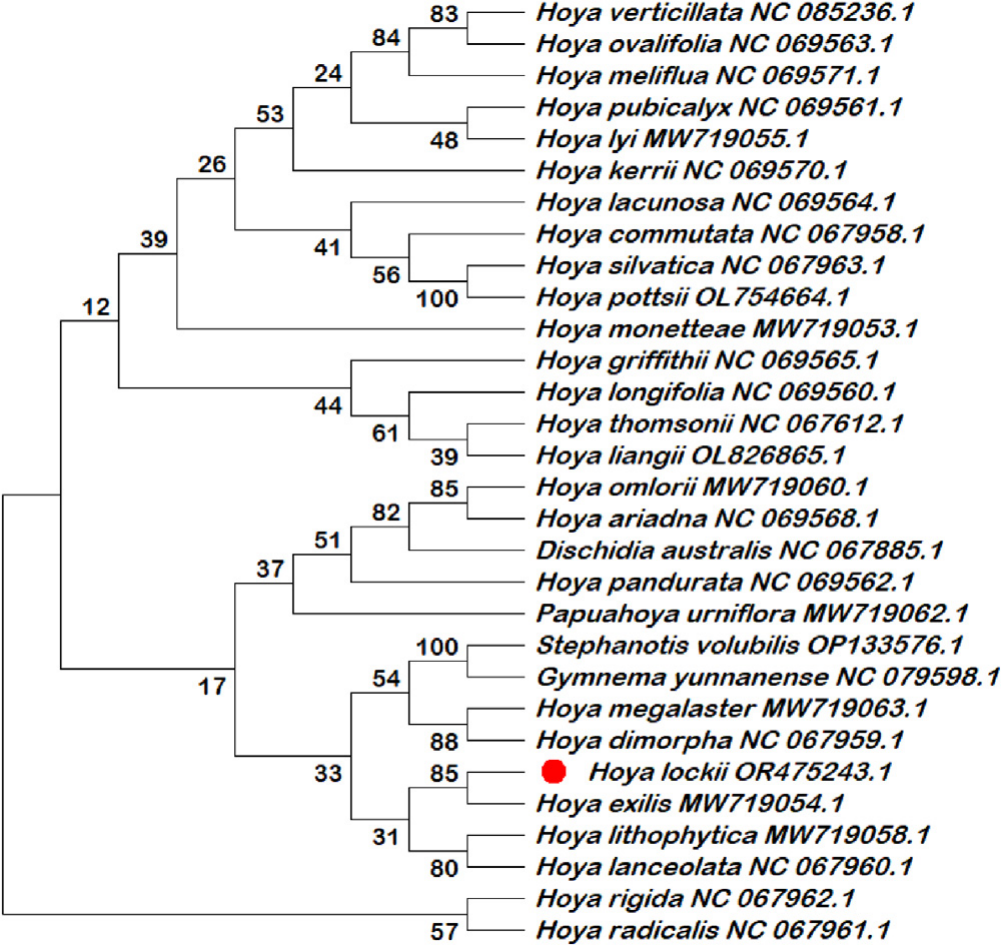
Construction of a phylogenetic tree based on the *trnH-psbA* intergenic spacer region of the chloroplast genome of *Hoya lockii* V.T. Pham & Aver. and other species within the Apocynaceae family.

**Fig. 5:**
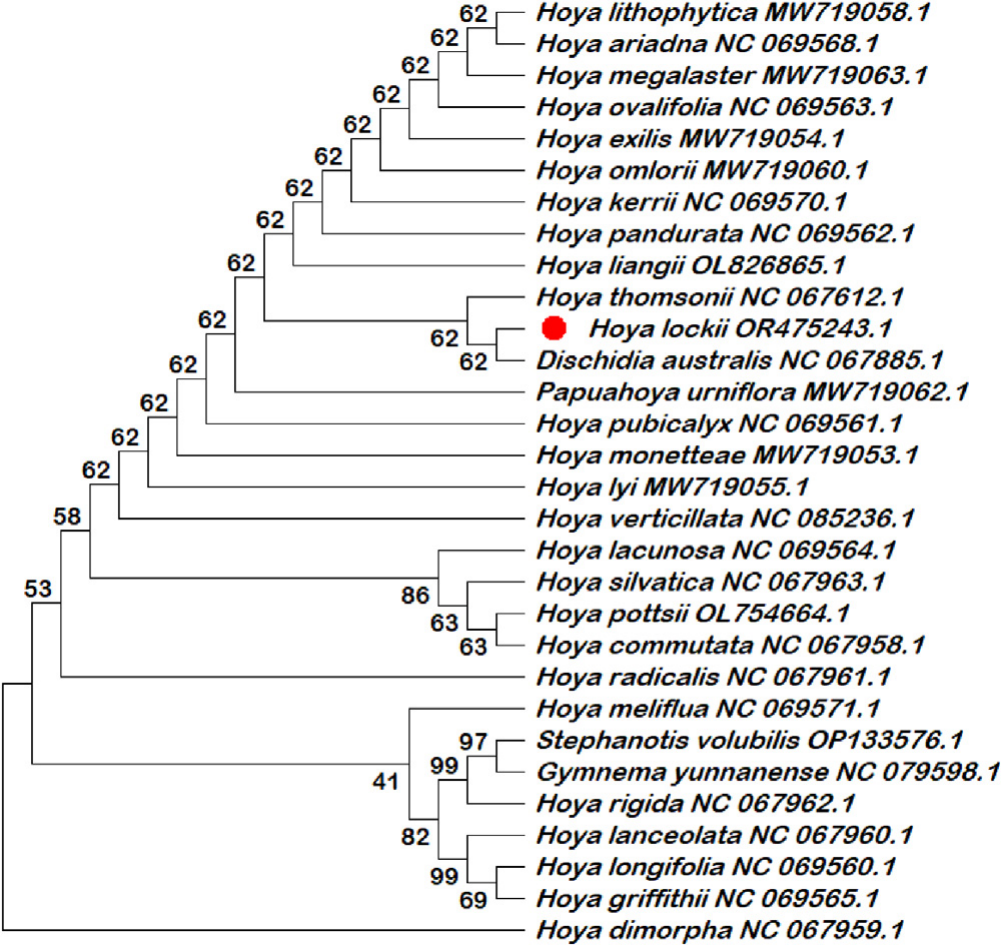
Construction of a phylogenetic tree based on the *psbK-psbI* intergenic spacer region of the chloroplast genome of *Hoya lockii* V.T. Pham & Aver. and other species within the Apocynaceae family.

**Fig. 6:**
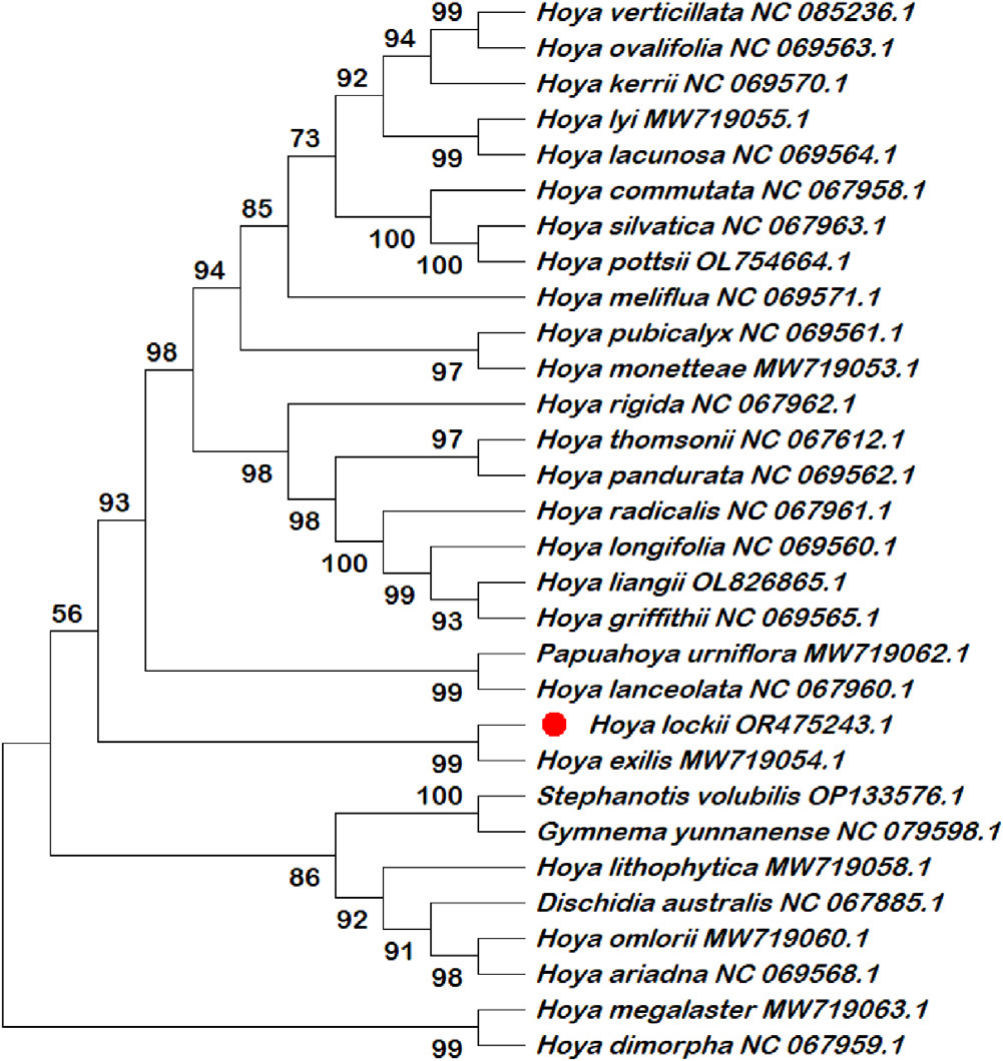
Construction of a phylogenetic tree based on the *ndhC-trnV* intergenic spacer region of the chloroplast genome of *hoya lockii* V.T. Pham & Aver. and other species within the Apocynaceae family.

**Fig. 7:**
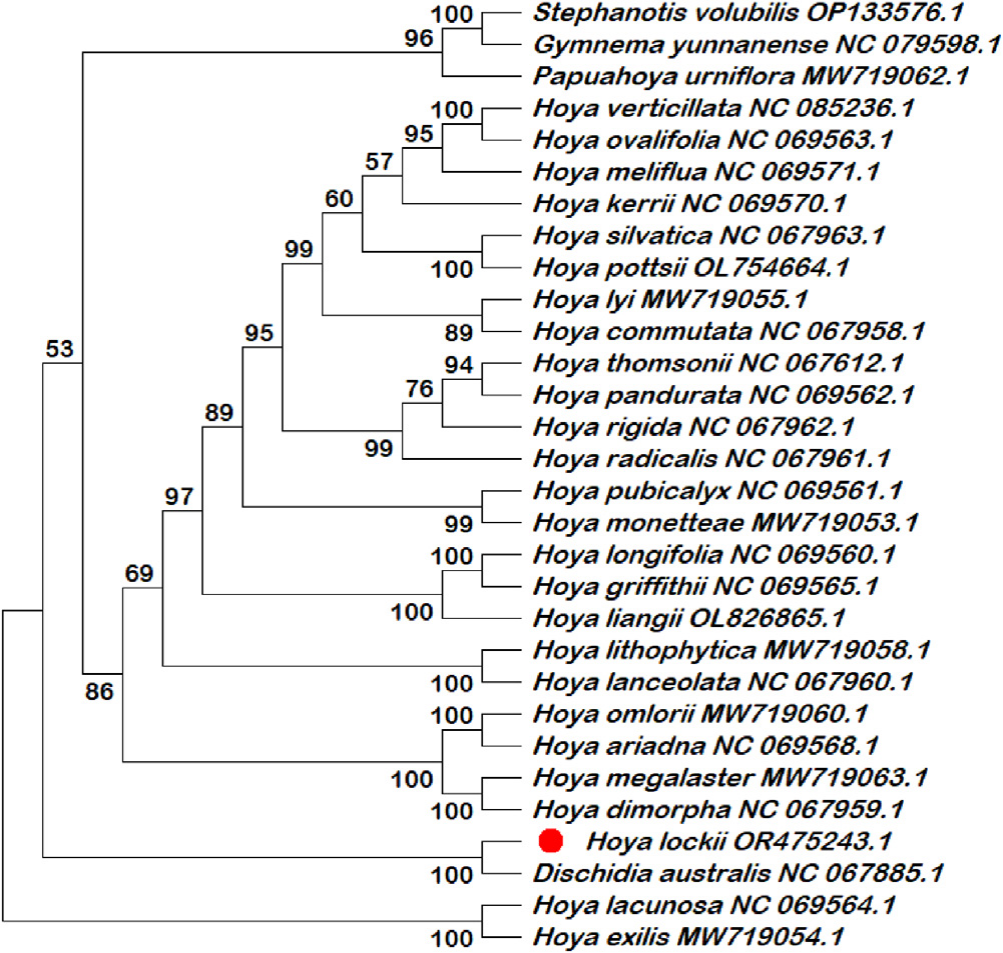
Construction of a phylogenetic tree based on the *rbcL-accD* intergenic spacer region of the chloroplast genome of *Hoya lockii* V.T. Pham & Aver. and other species within the Apocynaceae family.

**Fig. 8:**
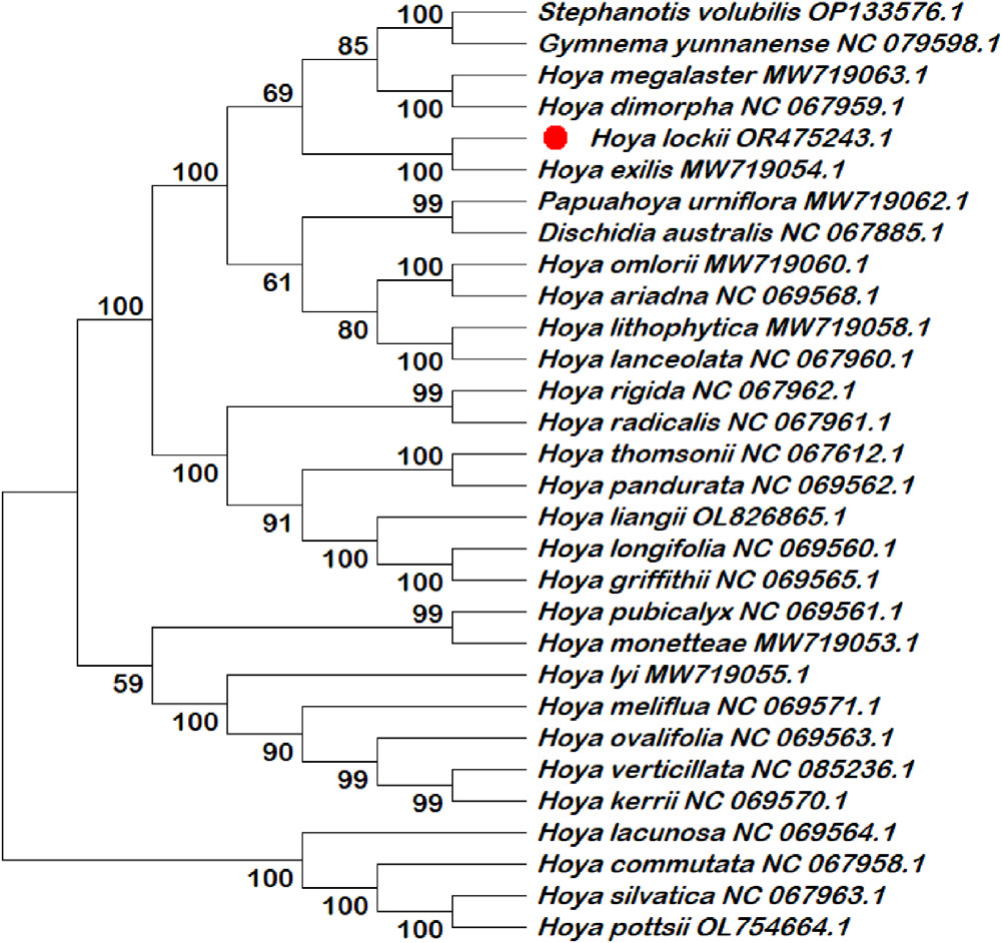
Phylogenetic tree reconstructed based on concatenated alignments of six chloroplast intergenic spacer regions (*trnK–rps16, psbI–atpA, trnH–psbA, psbK–psbI, ndhC–trnV,* and *rbcL-accD*) from the chloroplast genomes of *Hoya lockii* V.T. Pham & Aver. and related species within the family Apocynaceae.

Six chloroplast intergenic spacer regions (*trnK–rps16, psbI–atpA, trnH–psbA, psbK–psbI, ndhC–trnV*, and *rbcL–accD*) were extracted and compiled in this dataset as candidate molecular markers (Table 1; Table S1). These intergenic spacer regions were subjected to phylogenetic reconstruction using both single-locus and multilocus datasets (Fig. 2, Fig. 3, Fig. 4, Fig. 5, Fig. 6, Fig. 7, Fig. 8). Phylogenetic analyses based on individual intergenic spacer regions showed consistent placement of *H. lockii* within the genus *Hoya*, although its closest relatives varied depending on the marker analyzed. In Fig. 3, *H. lockii* forms a clade with *Hoya lanceolata*, supported by a high bootstrap value (99%), indicating strong phylogenetic affinity between the two species. Similarly, the phylogenetic tree in Fig. 6 shows *H. lockii* clustering with *Hoya exilis* (bootstrap = 99%). In Fig. 4, H*. lockii* forms a clade with *H. exilis*, supported by a high bootstrap value (85%), indicating relatively strong phylogenetic affinity between the two species.

**Table 1:** Statistical table of comparison of six intergenic spacer regions in the chloroplast genome of *Hoya lockii* V.T. Pham & Aver.

Intergenic spacer regions	Sequence length (bp)	Genome region	Genomic location/Coordinates
*trnK-rps16*	832	LSC	4226–5057
*psbI-atpA*	2106	LSC	8269–10,374
*trnH–psbA*	284	LSC	77–360
*psbK–psbI*	190	LSC	7968–8157
*ndhC–trnV*	1567	LSC	56,675–58,241
*rbcL–accD*	898	LSC	63,463–64,360

Analyses based on the *trnK–rps16, psbK–psbI, rbcL-accD* intergenic spacer regions (Figs. 2, Fig. 5, Fig. 7) placed *H. lockii* in close association with *Dischidia australis*, with bootstrap values of 98%, 62% and 100%, respectively.

Phylogenetic reconstruction based on concatenated alignments of six spacer regions substantially improved overall tree resolution and branch stability, recovering *H. lockii* as sister to *H. exilis* with strong bootstrap support (100%) (Fig. 8). These results demonstrate the complementary phylogenetic signals provided by individual loci and highlight the enhanced discriminatory power of the multilocus dataset for species identification within the genus *Hoya*.

Overall, phylogenetic analyses based on individual chloroplast intergenic spacer regions (Fig. 2, Fig. 3, Fig. 4, Fig. 5, Fig. 6, Fig. 7) and the concatenated dataset (Fig. 8) consistently resolved the placement of *H. lockii* among the examined taxa.

The chloroplast intergenic spacer regions *psbI–atpA, ndhC–trnV,* and *trnH-psbA* together with the concatenated dataset of six regions, may serve as potential DNA marker candidates for supporting species identification in the genus *Hoya*.

## 4 Materials and Methods

### 4.1 Sample collection and data sources

Plant material of *H. lockii* was collected in September 2022 from Dong Son, Ky Thuong Nature Reserve, Hoanh Bo District, Quang Ninh Province, Vietnam, and has been maintained in the experimental garden of Thainguyen University of Education.

Six chloroplast intergenic spacer regions of *H. lockii, trnK–rps16* (positions 4226–5057), *psbI–atpA* (positions 8269–10,374), *trnH–psbA* (positions 77–360), *psbK–psbI* (positions 7968–8157), *ndhC–trnV* (positions 56,675–58,241), and *rbcL–accD* (positions 63,463–64,360), were extracted from the *H. lockii* complete chloroplast genome deposited in GenBank (NCBI) under accession number OR475243.1 [5,10]. The corresponding FASTA sequences were retrieved for downstream analyses.

Homologous intergenic spacer sequences from other Apocynaceae species were identified through BLAST searches against the NCBI nucleotide database. Candidate sequences were selected based on Total Score, Query Coverage, and Percentage Identity, retaining only high-confidence matches derived from complete chloroplast genomes. Detailed BLAST results and accession information are provided in Supplementary Tables S3–S8. The intergenic spacer sequences found by BLAST on NCBI were verified against extracts from the complete chloroplast genome with defined coordinates (Table S2). Some sequences not found by BLAST were extracted from the complete chloroplast genome. It should be noted that BLAST-based similarity values represent pairwise local alignments and are calculated only from overlapping regions of comparable length. Therefore, when sequence lengths differ among taxa, parameters such as total alignment score and query coverage may better reflect overall alignment support, whereas percentage identity alone may not fully correspond to the phylogenetic relationships inferred from the concatenated sequence dataset.

Six intergenic spacer datasets (*trnK–rps16, psbI–atpA, trnH–psbA, psbK–psbI, ndhC–trnV*, and *rbcL-accD*) were successfully extracted from 30 species and used for comparative analyses (Tables S3–S8). A concatenated dataset of the five intergenic spacer regions was constructed using the shared set of 30 species.

### 4.2 Phylogenetic analysis

Chloroplast intergenic spacer sequences were retrieved from 30 complete chloroplast genomes of *Hoya* and related taxa available in GenBank. Six intergenic spacer regions, *including trnK–rps16, psbI–atpA, trnH–psbA, psbK–psbI, ndhC–trnV*, and *rbcL–accD*, were initially examined. Sequence alignment was performed using the alignment function implemented in MEGA12 [11] (Figs. S1-S6, and deposited athttps://data.mendeley.com/datasets/tb3yyhx8×6/2).

Phylogenetic relationships were reconstructed using the Maximum Likelihood (ML) method under the Tamura–Nei (1993) nucleotide substitution model [12]. Nodal support was evaluated using bootstrap analysis with 1000 replicates, and bootstrap values are indicated on the corresponding branches [13]. Phylogenetic trees were inferred separately for each intergenic spacer region.

Sequences from six intergenic spacer regions (*trnK–rps16, psbI–atpA, trnH–psbA, psbK–psbI, ndhC–trnV* and *rbcL-accD*) were concatenated in MEGA12 (Toolbar: Open Multiple Files → Concatenate) and used to construct a combined phylogenetic tree.Separate phylogenetic trees were generated for each individual intergenic spacer region (Fig. 2, Fig. 3, Fig. 4, Fig. 5, Fig. 6, Fig. 7), while an additional tree was reconstructed from the concatenated dataset (Fig. 8).

## Limitations

The dataset was generated from the complete chloroplast genome of *Hoya lockii*, previously sequenced and deposited in GenBank by our research group. It includes six chloroplast intergenic spacer sequences selected: *trnK–rps16, psbI–atpA, trnH–psbA, psbK–psbI, ndhC–trnV*, and *rbcL–accD.* These loci provide useful molecular information for phylogenetic analysis and preliminary evaluation of potential DNA barcode markers. However, the dataset represents only a subset of chloroplast intergenic regions and may be complemented by additional loci or samples in future studies to further improve comparative analyses.

## Ethics Statement

The authors have read and followed the ethical requirements for publication in Data in Brief and confirmed that the current work does not involve human subjects, animal experiments, or any data collected from social media platforms.

## CRediT Author Statement

**Manivanh Yongsa and Tan Quang Tu:** Conceptualization, Formal analysis, Investigation, Resources, Writing – original draft; **Nga Thi Thu Nguyen:** Data curation, Investigation, Writing – original draft; **Thuong Danh Sy and Lan Thi Ngoc Nguyen:** Formal analysis, Resources, Writing – original draft; **Tan Quang Tu and Mau Hoang Chu:** Conceptualization, Data curation, Methodology, Supervision, Writing – review & editing.

## Data Availability

NCBIComplete chloroplast genome of Hoya lockii (Apocynaceae) (Original data).

## Appendix Supplementary materials

Image, application 1
