# Supplementary material for: Dataset of chloroplast intergenic spacer sequences and candidate DNA markers for species identification in Hoya (Apocynaceae) based on the plastome of Hoya lockii V.T. Pham & Aver. from Vietnam
Source: Data Brief. 2026 Apr 9;66:112757. doi: 10.1016/j.dib.2026.112757 (PMC13099943; doi:10.1016/j.dib.2026.112757)
Supplement: Supplementary file 1 [file mmc1.docx]

# Supplementary

## Table S1. Sequence characteristics of six intergenic spacer regions in the chloroplast genome of *Hoya lockii*

| **Intergenic spacer region** | **Sequence length (bp)** | **Sequence characteristics** |
| --- | --- | --- |
| *trnK-rps16* | 832 | GAAAAGAAGAAACCAATTATAGAAGAGAATCTATCGACTATAAAATATATGTATAATAAAAAAGAAGTGCATAAATACAATCGGAATTAGAAAAAATTAAACAATAAATTAAACAAAGAAATTAGGCGAAGCAATTAAACCATTAAGCTAACAAATCGAAAAAATGGATTTTCTAATGAATTCGGAACATAAAAAAATATATAGATCGGATGACAATATAAGAAATTCTCAAATAAAATTCTATTTAGATAAAGCAAAACAAAAAGAAAAAAAAAATGAAAATAGAGATTAAAAATTTATGAACGGATCCTTTTGTTATCCCCTCTTTTCAATCAACCAAAAAAGCTCGTGTATCAAAGAACCTGTCGACGTAAAGTAAAAAATCAAACAAACCAAATAACTAGATCTAACTTCACTTATCATGATGAATTATATTTGTTCAATACACTGTTGTCAATATAAATGTTACGTCATGATGAATTATATTTGTTCAATACACTGTTGTCAATATAAAAGTTACGAAAAGAAAAGAATAAAAAAAAAAAAGAAATTCAATTGAATTTCTTTTTTTTTTTTATTCTTTTTTGTTAGTTAAATTCAAAATAAAAATGAAAACCATCAAGTTTAAAGTTGTCGCGAATGCCTAAATTTTCTAGGATCAAGGAAATAGGGTTTTGTCGTTATAGAACACAGGATTCGAAGAAAACAATGATAAATAGCTACGAATAGAACGGGAAAAGGAAAAAAGGGGGGGGTAATAAAAAAGAGTAGAGAAAGGTTATCCAAAGTTATATACAAAAAACTGCCCCCCTTTTTTGTATTTCCTTAATCC |
| *psbI-atpA* | 2106 | GGGTTTTTCTTTTCTATTTTCTTCGGATTTTTTGTTTATATAAAAACAAACAATTTGCCAAATTCGAAAAAATAAATAAATAAAGTCATCAAGGGAAGCGGAAAGAGAGGGATTCGAACCCTCGGTACGAATAACTCGTACAACGGATTAGCAATCCGCCGCTTTAGTCCACTCAGCCATCTCTCCTAATTGAAAATTGGGTTAGATTACACATAAAAAAAAAATAAGGAGTATCCCTTTCTCTATTATATAGATATCTACAATTTTTATCAGTAATTTATTTTCGATAAATAAAGAATAAATAAAGAAAAAGGGCTCAAAAGTGCCAACAATATAAAGAAAACAAAAAGCGACCCCTTCGTATTTTGTTCGAAAGACCCTTCTTATTTTTAATTATTTTTAATTTTATTTATTTTATTGTTTATTGTTATTGACACGGCCTGGGCTGGTCAGTACCCAGCCGGGCCTCCTTTTGTTTTGTTCCAACTAATTTTAGAAAAATAATGGTAATTTTTCATTTATCTGATTTGAAAACAAAAATGCTTAGTTTTTTTTGTATTTTTTTTATATATTTTTTTTATATATTTTAATAAATATATATTTTAATAAATATATATTTTAATAAATATATATTTTAATAAATTATAAATTATATATATATATACATATATATATATATATATATATATATAATTAATTATATAGTATTATTATTATATATATATTATATATTATAAAAAAATATATATATATTATAAAAAAATAAAAAAAAAAGATATATAAAAAATATAAATAAAATATATAAAAAAAGGGAATAGGCAATATTCAAGCACGAACAAAAAAAGAAGAAGGACTATTTCCATCCGCGAAAACAAAAATAAAGGAGCGCTAAATTTTTGGATTCTTTGATGATCCCATCTTATTATACCCATTTTCCGGTTCGACAAAAGGTCCAGTTGTATACAATAATCGAATTGTAGCGGGTATAGTTTAGTGGTAAAAGTGTGATTCGTTTTTTTAACCCTTTGATAGTTAAAGGGTCTTTGTTTTCGGTTTGATTCGTATTCCGACAAAAAACTTTATTTGAAAAAAAAAAGGATTTAATCCTTTACCTCTCAATCACGGATTCGAGAAAAAATATACATTCTCGTGATTTGTATCCAAGGATCACTTAGAAAGTGACAAATTGGATTATGAAATTACGAAACATAATTTTGGAATTGGATTAATACTTCAAATTGAATCAGTATGAGTAAAGGATCCATGGATAAAGATAGCAAGTAGGTTTCTAATCGTAACTAAATCTTCAATTTTTTCTTTACAAATAAAAAATTGAATCAAAATAGCTATTAAATGATGACTCTGGTTTACTAGAGTCATCGACCCGTTTTTTTAGCTCGGTGGAAACAAAATCTCTTTCCTCAGGACCGTCTCAAATAAAAATAGAGAACGAAGTAACTAGAAAGATTGTTAGAATTACTCTCTTCTAGAGGGATCATCTATAAAGAAATTAGTAGTCAGACAAAAGTTGACATAGATGTTATGGGTAGAATTTTTTTTTGTAAGTTTGTTCACATCCATATCCATAAAGGAGCCGAATGAAACCAAAGTTTCATGTTCGGTTTTGAATTAGAGACGTTCAAAATGCTGAATCGACGTCGACTATAACCCCTAGCCTTCCAAGCTAACGATGCGGGTTCGATTCCCGCTACCCGCTTTCAATTCTTTTTTTTTATTCGAAATTGAAATTTATATATATTATAATTCTAGACTTAATGCACCATTTAATATACAGTTTCAAAAATTATCTCACATACAATCCGATTCTTTTTTTTTTCGGCGAAAAAGTGGGGAAAGTCAAAATACGAAAAAAATCGGAATGAAAAGCGTCCATTGTCTAATGGATAGGACAGGGGTCTTCTAAACCTTTGGTATAGGTTCAAATCCTATTGGACGCAAATTTTTTCCATATATCTATTTTTTTTTTTTTTTTTGATACCACGAAAGCCTTTTCGAATAACTTGAATTTGAGTGAATTTGAGACCCTTAATTACATTACCATTACCTTTTTAAGATAAATTTAAGATAAAAGAATCCTTTTTGTGC |
| *trnH-psbA* | 284 | CCCCTCGTTCTCTTTATATTTATTTCTTATAATTTTTTTCTTCTATTTATATTAATATGTTTAGGTTAATATTAAGAATAAGAAATTTCATTTTAAATGAAAATTATATTTTATTTATAATATTGTTTCTACCTTTTTTTTCTGATATATTTTCATTTTTGATAATGTACAAAAAAAAGAAAAAAAAAGGAGCAATATTGCCCTCTTGATAGAACAAGAAGGTGGTATTGCTCCCTTATTTTCCAATAACTCCTATACACTAATATACACTAAGACTGAAGGTC |
| *PsbK-psbI* | 190 | GATCCTTAATCTAAAATATCTAAAATAGAAATTTTTTAAATTTAAAAATTTCTTACAAATTTCCTAGATTTCCTCGAAAGTTCGTGATTTTTTCTAGAAAGAAACTCGGCTCTTGATATCCAAATAGGATATGTGGTAGAAAAATGGAGGATCTATTCTCTTTTTTTTTAATTATCTTGGAGATTGTGTA |
| *ndhC-trnV* | 1567 | AGACACACTCCCATGAACGTGGAAAATATACCGAATTTCTCGATTCGAATTGTCAAGTCATCCATAACTGTTTAGTCAAAATCAAAACACGAATTCATTTTTATCGAATTTACTAGTTTCCTTTGTTTACTTATCCCATTTCAAGACAAAATCCTATTTAGATTATACTTCTTTCTATTTTTTCAGTTAGTATAACTTTAGATCTATATCTTATTCTTATACAAATATAAAATTCTTATACAAATATAAAATAGAAAAATTCTCTTATTTTAACCTAGAATTCTCTGTAATCTGTAAAGAAGAAAAAGAATTCAAAAAAATATGGAATTCCCCTTGGAATTTGAAACTTCTTTATCTATTATATATTTCTATTTTTTATATTTTATATATTAATTATATATATATATATATATATATATTATACTATTTTATACTATTTTTTGAAATTGATGAAATTTAATAAAATCAATTGAAATAAATGAAAAAAATATTTAATTCAAATAAAAAAATATAGATTATTTCTTAATTTCTATAATATTATAGCTATAATATTATAGAAATTATATTATAGATTCTGATTCTTTATTATTTTTTTTTTTCATTATTATTATATTATATATTCTAGATTATTTATTATTATTTTTATTTATATTTTTTTATTAAAATTTGAATATTAATTAATAAATTTTTTATTTTTTATTATTAAATACAATCTAATAATACAATTTAAATTCTATTTTATTTCGAATTTGAAATGTTTTAAATTGAATTGCTAATTTTTTTTTATTGAAATTTAATTCTTTTTCTTTATAGATTTCGACTTTTATGAATAGAATCGACAGGTCCTTTGGTTTTTTTTCTTAGTGATTTAGAATAAGAACAAGCAAGAGACTGGCTAGAAATTTCCATTTCAGAATTTAGAATATCTTGGTGTTGTATATTCTTTTTATTAGTATCCTAGCGGAGGACCTTCTCTTGATTGAATAGAAAAAAAGAAGACAGACTACCCCTCGTGCTTCGCTAGGTCTAGGTAAGGTATATGGCGAGCCTATTTTACATTGTTAATTGTTAATAATGAGACTTACCAAAGATATTACATTTTTTAACTTGTACACAAGCACGACAGGTCCTTTCTAGATCCATTGGGGTTTTTAACAGGGTGACTCGTGTCATGATTTTGACTTCAAAAATTCACCAACATTGGCTATACCAAAGAAAGGAAGTCTCAAGAACTCCTTGAATATGAAACCCACCCCTACCCCCAAGATTAATGCCCCAAGATTAATAACAAAAGGTTGCTTTGTTTATCCGCGATTGGAAAACTATCAATTGGATCCACTGAAACGCCTTTTTTTTTCGGGTTTATGTTCTGTTTTAAAAGCTTGCCGTGAGTAAACTTATGGGAAAAATTTATATTTCGATTAACCAACCCGACAGTTCCAAGCAACAAACAATAATGAAAAAATTATACAATTTCGTTTTTTGTATTTGAGTTTTCCTTAATCATTTTGTTTTATATTTTATATTATATATATATAAAATATAGTAGTAATAGTAAGTAATAGTA |
| *rbcL-accD* | 898 | TTACTTTTTGTTATCTTAGTTGAATTGTAATTAAACTCGGCCCAATCTTTTCCTAAAAGGATTGAGCCGAATACAAAGATTCTCTTTTTTTTTTATTATTATTTTTTAAGTATTTAATATTAATACATACTTATATAGATATAGATATAAGATTATATAGATATAAGATTTGAAATAAAAAAATCAAAAAAATCTAAGGCTCAAATCTTTCTATTGTTGTCTTGGATCTACAATTAATCTACGGATCTTTGGGCTTGGGATTGGGCTATTCTTTCTTTTATATCCTGCAGTTTTACTGAATCAAGCCAAGTATCACAATTCTTTCTACCCATCCTGTATATTGTCCTTTTCTTTCCATATTGGTGAAATAGAACCTGAAATTTTTACGTTCTTGGGCGAAATTTTAGATTTATAAAATATTTTTTTTCGATGCGAATTTGATACGACATAAGAAAAGGCGCTCTTTATCATTGTATTTATAATGACAAGAGATTACCTGATATCATATTAATATATTGACAACATTCAAAGAATAATATATATAATATATAATGTATAAAATGTATAAAAATAATGACAAGGGATTCCCTGATATCATATCATATTAATATATTGACAGTATTCAAAAAATAATATATAATGTATAAAATATAAAATAAAATATAAAATGTATAAAAAACTCCCTGATATGATATTAATATATAGTGTATAGTCATATATAGCGTATAGTGAAGTATTACTCCGGATTTTCAAAAAAGAGCATTTTTTTTTTTTCAATAATCACACCTAATACCTTCTTATTGTTTCTTATTGGTTATCTTATTAGTTAATAAAAAATGCTAGCGATTGGTTTAGTCTAATAGGAAAGAAGATATTCAAATAAAGAATTTTTGATCAA |

## Table S2. Statistical comparison of genomic location and sequence length (bp) for six chloroplast intergenic spacer regions between *Hoya lockii* and selected species retrieved from GenBank.

| Genus | Scientific Name | Accession | *trnK-rps16* | | *psbI-atpA* | | *trnH-psbA* | | *psbK-psbI* | | *ndhC-trnV* | | *rbcL-accD* | |
| --- | --- | --- | --- | --- | --- | --- | --- | --- | --- | --- | --- | --- | --- | --- |
|  |  |  | GL | SL | GL | SL | GL | SL | GL | SL | GL | SL | GL | SL |
| *Hoya* | *Hoya lockii* | NC_085235.1 | 4226-5057 | 832 | 8269-10374 | 2106 | 77-360 | 284 | 7968-8157 | 190 | 56675-58241 | 1567 | 63463-64360 | 718 |
|  | *Hoya ariadna* | NC_069568.1 | 4268-5099 | 832 | 8679-10631 | 1953 | 77-403 | 327 | 8365-8567 | 203 | 56933-58464 | 1532 | 63655-64516 | 906 |
|  | *Hoya commutata* | NC_067958.1 | 4224-5003 | 780 | 8477-10434 | 1958 | 77-354 | 278 | 8162-8365 | 204 | 56655-58198 | 1544 | 63423-64177 | 903 |
|  | *Hoya dimorpha* | NC_067959.1 | 4243-5065 | 823 | 8590-10688 | 2099 | 77-383 | 307 | 8276-8478 | 203 | 57214-58715 | 1502 | 63908-64849 | 824 |
|  | *Hoya exilis* | MW719054.1 | 4224-5037 | 814 | 8363-10764 | 2402 | 78-366 | 289 | 8031-8251 | 221 | 57152-58631 | 1480 | 63856-64822 | 967 |
|  | *Hoya griffithii* | NC_069565.1 | 4224-5051 | 828 | 8580-10607 | 2028 | 77-359 | 283 | 8257-8468 | 212 | 56732-58234 | 1504 | 63424-64273 | 911 |
|  | *Hoya kerrii* | NC_069570.1 | 4251-5033 | 783 | 8520-10500 | 1981 | 77-384 | 308 | 8205-8408 | 204 | 56819-58382 | 1564 | 63580-64429 | 829 |
|  | *Hoya lacunosa* | NC_069564.1 | 4212-4934 | 723 | 7936-9816 | 1879 | 77-354 | 278 | 7621-7824 | 204 | 55731-57168 | 1438 | 62358-63221 | 824 |
|  | *Hoya lanceolata* | NC_067960.1 | 4223-5116 | 894 | 8601-10513 | 1913 | 77-361 | 285 | 8308-8489 | 209 | 56223-57640 | 1418 | 62848-63694 | 854 |
|  | *Hoya liangii* | OL826865.1 | 4224-5051 | 828 | 8573-10564 | 1992 | 77-359 | 283 | 8259-8461 | 203 | 56657-58158 | 1502 | 63348-64206 | 916 |
|  | *Hoya lithophytica* | MW719058.1 | 4215-4948 | 734 | 8238-10293 | 2056 | 78-350 | 273 | 7923-8126 | 204 | 55512-57131 | 1620 | 62318-63519 | 1202 |
|  | *Hoya longifolia* | NC_069560.1 | 4224-5050 | 827 | 8581-10588 | 2008 | 77-359 | 283 | 8258-8469 | 212 | 56740-58242 | 1503 | 63432-64281 | 919 |
|  | *Hoya lyi* | MW719055.1 | 4233-5068 | 829 | 8570-10671 | 2102 | 78-371 | 294 | 8268-8458 | 191 | 56999-58577 | 1579 | 63781-64698 | 2524 |
|  | *Hoya megalaster* | MW719063.1 | 4254-5081 | 828 | 8678-10807 | 2130 | 78-390 | 313 | 8364-8566 | 203 | 57306-58818 | 1512 | 64016-65112 | 919 |
|  | *Hoya meliflua* | NC_069571.1 | 4265-5097 | 833 | 8570-10557 | 1988 | 77-397 | 321 | 8267-8458 | 192 | 56895-58358 | 1464 | 63560-64478 | 850 |
|  | *Hoya monetteae* | MW719053.1 | 4218-5053 | 836 | 8574-10639 | 2066 | 78-359 | 282 | 8259-8462 | 204 | 56965-58570 | 1606 | 63768-64603 | 836 |
|  | *Hoya omlorii* | MW719060.1 | 4268-5101 | 834 | 8681-10622 | 1942 | 78-403 | 326 | 8367-8569 | 203 | 57018-58555 | 1538 | 63747-64844 | 2526 |
|  | *Hoya ovalifolia* | NC_069563.1 | 4218-5053 | 836 | 8569-10472 | 1904 | 77-360 | 284 | 8254-8457 | 204 | 56631-58167 | 1537 | 63367-64282 | 898 |
|  | *Hoya pandurata* | NC_069562.1 | 4232-5058 | 827 | 8594-10466 | 1873 | 77-363 | 287 | 8293-8482 | 190 | 56470-57982 | 1513 | 63188-64041 | 859 |
|  | *Hoya pottsii* | OL754664.1 | 4254-5051 | 798 | 8510-10400 | 1891 | 77-383 | 307 | 8206-8398 | 193 | 56142-57640 | 1499 | 62872-63695 | 847 |
|  | *Hoya pubicalyx* | NC_069561.1 | 4248-5080 | 833 | 8594-10654 | 2061 | 77-375 | 299 | 8268-8482 | 215 | 56858-58337 | 1480 | 63535-64363 | 864 |
|  | *Hoya radicalis* | NC_067961.1 | 4218-5040 | 823 | 8401-10265 | 1865 | 77-357 | 281 | 8099-8289 | 191 | 56395-57910 | 1516 | 63109-64038 | 930 |
|  | *Hoya rigida* | NC_067962.1 | 4239-5063 | 825 | 8554-10583 | 2030 | 77-373 | 297 | 8249-8442 | 194 | 56796-58334 | 1539 | 63526-64436 | 850 |
|  | *Hoya silvatica* | NC_067963.1 | 4254-5051 | 798 | 8510-10400 | 1891 | 77-383 | 307 | 8206-8398 | 193 | 56142-57640 | 1499 | 62872-63695 | 850 |
|  | *Hoya thomsonii* | NC_067612.1 | 4233-5061 | 829 | 8562-10537 | 1976 | 77-365 | 289 | 8248-8450 | 203 | 56569-58094 | 1535 | 63287-64189 | 942 |
|  | *Hoya verticillata* | NC_085236.1 | 4234-5026 | 793 | 8524-10401 | 1878 | 77-368 | 292 | 8210-8412 | 203 | 56595-58087 | 1492 | 63278-64183 | 755 |
| *Dischidia* | *Dischidia australis* | NC_067885.1 | 4245-5089 | 845 | 8604-10569 | 1966 | 77-367 | 291 | 8301-8492 | 192 | 56501-58024 | 1524 | 63220-64043 | 862 |
| *Gymnema* | *Gymnema yunnanense* | NC_079598.1 | 4413-5204 | 790 | 8586-10584 | 1999 | 77-541 | 465 | 8292-8474 | 183 | 56815-58236 | 1422 | 63426-64123 | 698 |
| *Papuahoya* | *Papuahoya urniflora* | MW719062.1 | 4221-4889 | 669 | 8468-10533 | 2066 | 78-363 | 286 | 8166-8356 | 191 | 56587-58164 | 1578 | 63365-64293 | 718 |
| *Stephanotis* | *Stephanotis volubilis* | OP133576.1 | 4448-5227 | 780 | 8619-10539 | 1921 | 77-585 | 509 | 8326-8507 | 182 | 56774-58160 | 1387 | 63334-64051 | 824 |

Remarks: “GL” Genomic location/Coordinates; “SL” Sequence length (bp).

## Table S3. Statistical table of comparison results for the *trnK-rps16* region between *H. lockii* species and some other species on GenBank

| **Genus** | **Scientific Name** | **Accession** | **Total Score** | **Query Cover (%)** | **Per. Ident (%)** |
| --- | --- | --- | --- | --- | --- |
| *Hoya* | *Hoya lockii* | NC_085235.1 | 1717 | 100 | 100 |
|  | *Hoya griffithii* | NC_069565.1 | 1602 | 100 | 97.48 |
|  | *Hoya liangii* | OL826865.1 | 1602 | 100 | 97.48 |
|  | *Hoya longifolia* | NC_069560.1 | 1600 | 100 | 97.48 |
|  | *Hoya thomsonii* | NC_067612.1 | 1507 | 100 | 97.36 |
|  | *Hoya omlorii* | MW719060.1 | 1589 | 100 | 97.36 |
|  | *Hoya radicalis* | NC_067961.1 | 1490 | 100 | 97.12 |
|  | *Hoya dimorpha* | NC_067959.1 | 1580 | 100 | 97.12 |
|  | *Hoya megalaster* | MW719063.1 | 1569 | 100 | 96.89 |
|  | *Hoya ariadna* | NC_069568.1 | 1573 | 100 | 96.78 |
|  | *Hoya rigida* | NC_067962.1 | 1464 | 100 | 96.53 |
|  | *Hoya exilis* | MW719054.1 | 1450 | 100 | 96.51 |
|  | *Hoya lyi* | MW719055.1 | 1453 | 100 | 96.09 |
|  | *Hoya ovalifolia* | NC_069563.1 | 1453 | 100 | 96.09 |
|  | *Hoya monetteae* | MW719053.1 | 1442 | 100 | 95.85 |
|  | *Hoya pubicalyx* | NC_069561.1 | 1528 | 100 | 95.86 |
|  | *Hoya meliflua* | NC_069571.1 | 1427 | 100 | 95.62 |
|  | *Hoya pandurata* | NC_069562.1 | 1355 | 100 | 94.33 |
|  | *Hoya lithophytica* | MW719058.1 | 1419 | 89 | 96.86 |
|  | *Hoya lanceolata* | NC_067960.1 | 1683 | 100 | 96.23 |
|  | *Hoya verticillata* | NC_085236.1 | 1387 | 100 | 95.65 |
|  | *Hoya kerrii* | NC_069570.1 | 1357 | 100 | 95.63 |
|  | *Hoya liangii* | NC_042245.1 | 1361 | 100 | 95.63 |
|  | *Hoya silvatica* | NC_067963.1 | 1283 | 100 | 94.66 |
|  | *Hoya pottsii* | OL754664.1 | 1283 | 100 | 94.66 |
|  | *Hoya lacunosa* | NC_069564.1 | 1040 | 84 | 92.87 |
| *Dischidia* | *Dischidia australis* | NC_067885.1 | 1515 | 100 | 95.77 |
| *Gymnema* | *Gymnema yunnanense* | NC_079598.1 | 1278 | 100 | 93.97 |
| *Papuahoya* | *Papuahoya urniflora* | MW719062.1 | 1324 | 81 | 96.82 |
| *Stephanotis* | *Stephanotis volubilis* | OP133576.1 | 1276 | 100 | 93.57 |

## Table S4. Statistical table of comparison results for the *psbI-atpA* region between *H. lockii* species and some other species on GenBank

| **Genus** | **Scientific Name** | **Accession** | **Total Score** | **Query Cover (%)** | **Per. Ident (%)** |
| --- | --- | --- | --- | --- | --- |
| *Hoya* | *Hoya lockii* | NC_085235.1 | 3950 | 100 | 100 |
|  | *Hoya dimorpha* | NC_067959.1 | 2995 | 100 | 92.08 |
|  | *Hoya lyi* | MW719055.1 | 3131 | 99 | 93.46 |
|  | *Hoya monetteae* | MW719053.1 | 3120 | 99 | 93.39 |
|  | *Hoya pubicalyx* | NC_069561.1 | 3026 | 99 | 93.51 |
|  | *Hoya lithophytica* | MW719058.1 | 3059 | 98 | 93.58 |
|  | *Hoya rigida* | NC_067962.1 | 3114 | 98 | 93.36 |
|  | *Hoya megalaster* | MW719063.1 | 3133 | 99 | 92.74 |
|  | *Hoya griffithii* | NC_069565.1 | 3044 | 99 | 92.78 |
|  | *Hoya exilis* | MW719054.1 | 3333 | 99 | 94.53 |
|  | *Hoya longifolia* | NC_069560.1 | 3254 | 99 | 94.88 |
|  | *Hoya meliflua* | NC_069571.1 | 3180 | 99 | 95.19 |
|  | *Hoya kerrii* | NC_069570.1 | 3234 | 99 | 95.18 |
|  | *Hoya omlorii* | MW719060.1 | 3132 | 96 | 96.18 |
|  | *Hoya thomsonii* | NC_067612.1 | 3230 | 99 | 95.18 |
|  | *Hoya liangii* | OL826865.1 | 3212 | 99 | 94.7 |
|  | *Hoya ariadna* | NC_069568.1 | 3116 | 96 | 95.66 |
|  | *Hoya commutata* | NC_067958.1 | * | * | * |
|  | *Hoya verticillata* | NC_085236.1 | 3101 | 91 | 97.02 |
|  | *Hoya pandurata* | NC_069562.1 | 3110 | 89 | 97.03 |
|  | *Hoya lanceolata* | NC_067960.1 | 3161 | 90 | 97.30 |
|  | *Hoya silvatica* | NC_067963.1 | 3130 | 91 | 96.95 |
|  | *Hoya pottsii* | OL754664.1 | 3130 | 91 | 96.95 |
|  | *Hoya lacunosa* | NC_069564.1 | 3092 | 91 | 96.44 |
|  | *Hoya ovalifolia* | NC_069563.1 | 3133 | 90 | 96.91 |
|  | *Hoya radicalis* | NC_067961.1 | 3027 | 87 | 97.87 |
| *Dischidia* | *Dischidia australis* | NC_067885.1 | 3146 | 97 | 94.97 |
| *Gymnema* | *Gymnema yunnanense* | NC_079598.1 | 2997 | 98 | 92.82 |
| *Papuahoya* | *Papuahoya urniflora* | MW719062.1 | 3041 | 98 | 93.31 |
| *Stephanotis* | *Stephanotis volubilis* | OP133576.1 | 2881 | 88 | 94.75 |

Remarks: * Not detected in BLAST results; manually extracted from complete genome.

## Table S5. Statistical table of comparison results for the *trnH-psbA* region between *H. lockii* species and some other species on GenBank

| **Genus** | **Scientific Name** | **Accession** | **Total Score** | **Query Cover (%)** | **Per. Ident (%)** |
| --- | --- | --- | --- | --- | --- |
| *Hoya* | *Hoya lockii* | NC_085235.1 | 525 | 100 | 100 |
|  | *Hoya radicalis* | NC_067961.1 | 396 | 89 | 94.92 |
|  | *Hoya griffithii* | NC_069565.1 | 381 | 89 | 93.77 |
|  | *Hoya liangii* | OL826865.1 | 375 | 89 | 93.41 |
|  | *Hoya exilis* | MW719054.1 | 374 | 93 | 91.76 |
|  | *Hoya longifolia* | NC_069560.1 | 374 | 89 | 93.39 |
|  | *Hoya monetteae* | MW719053.1 | 364 | 89 | 92.64 |
|  | *Hoya thomsonii* | NC_067612.1 | 359 | 89 | 92.02 |
|  | *Hoya lithophytica* | MW719058.1 | 359 | 93 | 91.42 |
|  | *Hoya rigida* | NC_067962.1 | 350 | 89 | 91.04 |
|  | *Hoya commutata* | NC_067958.1 | 342 | 89 | 91.41 |
|  | *Hoya lacunosa* | NC_069564.1 | 337 | 89 | 91.02 |
|  | *Hoya ariadna* | NC_069568.1 | 322 | 88 | 89.51 |
|  | *Hoya dimorpha* | NC_067959.1 | 316 | 77 | 92.44 |
|  | *Hoya megalaster* | MW719063.1 | 315 | 77 | 92.38 |
|  | *Hoya omlorii* | MW719060.1 | 311 | 90 | 88.43 |
|  | *Hoya kerrii* | NC_069570.1 | * | * | * |
|  | *Hoya lanceolata* | NC_067960.1 | * | * | * |
|  | *Hoya lyi* | MW719055.1 | * | * | * |
|  | *Hoya meliflua* | NC_069571.1 | * | * | * |
|  | *Hoya ovalifolia* | NC_069563.1 | * | * | * |
|  | *Hoya pandurata* | NC_069562.1 | * | * | * |
|  | *Hoya pottsii* | OL754664.1 | * | * | * |
|  | *Hoya pubicalyx* | NC_069561.1 | * | * | * |
|  | *Hoya silvatica* | NC_067963.1 | * | * | * |
|  | *Hoya verticillata* | NC_085236.1 | * | * | * |
| *Dischidia* | *Dischidia australis* | NC_067885.1 | 324 | 88 | 90.51 |
| *Gymnema* | *Gymnema yunnanense* | NC_079598.1 | * | * | * |
| *Papuahoya* | *Papuahoya urniflora* | MW719062.1 | 368 | 89 | 92.64 |
| *Stephanotis* | *Stephanotis volubilis* | OP133576.1 | * | * | * |

Remarks: * Not detected in BLAST results; manually extracted from complete genome.

**Table S6.** Statistical table of comparison results for the *psbK-psbI* region between *H. lockii* species and some other species on GenBank

| **Genus** | **Scientific Name** | **Accession** | **Total Score** | **Query Cover (%)** | **Per. Ident (%)** |
| --- | --- | --- | --- | --- | --- |
| *Hoya* | *Hoya lockii* | NC_085235.1 | 351 | 100 | 100 |
|  | *Hoya meliflua* | NC_069571.1 | 326 | 100 | 97.40 |
|  | *Hoya radicalis* | NC_067961.1 | 324 | 100 | 97.38 |
|  | *Hoya pandurata* | NC_069562.1 | 324 | 100 | 97.37 |
|  | *Hoya lyi* | MW719055.1 | 318 | 100 | 96.86 |
|  | *Hoya silvatica* | NC_067963.1 | 309 | 100 | 95.85 |
|  | *Hoya pottsii* | OL754664.1 | 309 | 100 | 95.85 |
|  | *Hoya rigida* | NC_067962.1 | 289 | 100 | 93.88 |
|  | *Hoya lanceolata* | NC_067960.1 | 287 | 91 | 96.55 |
|  | *Hoya verticillata* | NC_085236.1 | 285 | 96 | 95.11 |
|  | *Hoya ariadna* | NC_069568.1 | 285 | 96 | 95.11 |
|  | *Hoya griffithii* | NC_069565.1 | 285 | 96 | 95.11 |
|  | *Hoya liangii* | OL826865.1 | 285 | 96 | 95.11 |
|  | *Hoya dimorpha* | NC_067959.1 | 285 | 96 | 95.11 |
|  | *Hoya longifolia* | NC_069560.1 | 285 | 96 | 95.11 |
|  | *Hoya exilis* | MW719054.1 | 283 | 85 | 98.16 |
|  | *Hoya thomsonii* | NC_067612.1 | 283 | 85 | 98.16 |
|  | *Hoya ovalifolia* | NC_069563.1 | 281 | 96 | 94.59 |
|  | *Hoya lithophytica* | MW719058.1 | 281 | 96 | 94.59 |
|  | *Hoya monetteae* | MW719053.1 | 281 | 96 | 94.59 |
|  | *Hoya omlorii* | MW719060.1 | 279 | 96 | 94.57 |
|  | *Hoya megalaster* | MW719063.1 | 279 | 96 | 94.57 |
|  | *Hoya pubicalyx* | NC_069561.1 | 279 | 84 | 98.14 |
|  | *Hoya lacunosa* | NC_069564.1 | 276 | 96 | 94.05 |
|  | *Hoya commutata* | NC_067958.1 | 276 | 96 | 94.05 |
|  | *Hoya kerrii* | NC_069570.1 | 270 | 100 | 91.67 |
| *Dischidia* | *Dischidia australis* | NC_067885.1 | 315 | 100 | 96.35 |
| *Gymnema* | *Gymnema yunnanense* | NC_079598.1 | 267 | 91 | 94.29 |
| *Papuahoya* | *Papuahoya urniflora* | MW719062.1 | 324 | 100 | 97.38 |
| *Stephanotis* | *Stephanotis volubilis* | OP133576.1 | 270 | 91 | 94.83 |

## Table S7. Statistical table of comparison results for the *ndhC-trnV* region between *H. lockii* species and some other species on GenBank

| **Genus** | **Scientific Name** | **Accession** | **Total Score** | **Query Cover (%)** | **Per. Ident (%)** |
| --- | --- | --- | --- | --- | --- |
| *Hoya* | *Hoya lockii* | NC_085235.1 | 2894 | 100 | 100 |
|  | *Hoya ariadna* | NC_069568.1 | 2174 | 100 | 92.21 |
|  | *Hoya megalaster* | MW719063.1 | 2165 | 100 | 92.28 |
|  | *Hoya omlorii* | MW719060.1 | 2124 | 100 | 91.62 |
|  | *Hoya kerrii* | NC_069570.1 | 2109 | 100 | 91.24 |
|  | *Hoya ovalifolia* | NC_069563.1 | 2102 | 100 | 91.49 |
|  | *Hoya dimorpha* | NC_067959.1 | 2091 | 100 | 91.54 |
|  | *Hoya lyi* | MW719055.1 | 2065 | 99 | 90.75 |
|  | *Hoya rigida* | NC_067962.1 | 2030 | 100 | 90.79 |
|  | *Hoya radicalis* | NC_067961.1 | 2030 | 100 | 90.84 |
|  | *Hoya thomsonii* | NC_067612.1 | 2012 | 100 | 90.46 |
|  | *Hoya pandurata* | NC_069562.1 | 1977 | 100 | 90.26 |
|  | *Hoya commutata* | NC_067958.1 | 1977 | 99 | 90.18 |
|  | *Hoya liangii* | OL826865.1 | 1975 | 100 | 90.20 |
|  | *Hoya griffithii* | NC_069565.1 | 1971 | 100 | 90.15 |
|  | *Hoya longifolia* | NC_069560.1 | 1971 | 100 | 90.15 |
|  | *Hoya monetteae* | MW719053.1 | 1971 | 98 | 90.14 |
|  | *Hoya pottsii* | OL754664.1 | 1947 | 100 | 90.00 |
|  | *Hoya silvatica* | NC_067963.1 | 1947 | 100 | 90.00 |
|  | *Hoya exilis* | MW719054.1 | 1862 | 93 | 90.69 |
|  | *Hoya lithophytica* | MW719058.1 | 2074 | 99 | 91.14 |
|  | *Hoya pubicalyx* | NC_069561.1 | 2059 | 94 | 92.95 |
|  | *Hoya meliflua* | NC_069571.1 | 2039 | 95 | 93.25 |
|  | *Hoya verticillata* | NC_085236.1 | 1989 | 98 | 89.71 |
|  | *Hoya lacunosa* | NC_069564.1 | 1930 | 92 | 95.33 |
|  | *Hoya lanceolata* | NC_067960.1 | 1994 | 90 | 92.75 |
| *Dischidia* | *Dischidia australis* | NC_067885.1 | 1805 | 99 | 88.60 |
| *Gymnema* | *Gymnema yunnanense* | NC_079598.1 | 1784 | 98 | 90.28 |
| *Papuahoya* | *Papuahoya urniflora* | MW719062.1 | 2200 | 100 | 92.26 |
| *Stephanotis* | *Stephanotis volubilis* | OP133576.1 | 1823 | 95 | 93.34 |

## Table S8. Statistical table of comparison results for the *rbcL-accD* region between *H. lockii* species and some other species on GenBank

| **Genus** | **Scientific Name** | **Accession** | **Total Score** | **Query Cover (%)** | **Per. Ident (%)** |
| --- | --- | --- | --- | --- | --- |
| *Hoya* | *Hoya lockii* | NC_085235.1 | 1659 | 100 | 100 |
|  | *Hoya exilis* | MW719054.1 | 1116 | 100 | 89.66 |
|  | *Hoya liangii* | OL826865.1 | 1086 | 100 | 89.47 |
|  | *Hoya radicalis* | NC_067961.1 | 1079 | 100 | 89.66 |
|  | *Hoya griffithii* | NC_069565.1 | 1068 | 100 | 89.22 |
|  | *Hoya longifolia* | NC_069560.1 | 1068 | 100 | 89.22 |
|  | *Hoya ariadna* | NC_069568.1 | 1062 | 100 | 88.99 |
|  | *Hoya dimorpha* | NC_067959.1 | 1053 | 100 | 88.85 |
|  | *Hoya rigida* | NC_067962.1 | 1000 | 100 | 88.20 |
|  | *Hoya lyi* | MW719055.1 | 979 | 100 | 87.58 |
|  | *Hoya monetteae* | MW719053.1 | 977 | 100 | 87.69 |
|  | *Hoya meliflua* | NC_069571.1 | 966 | 100 | 87.51 |
|  | *Hoya verticillata* | NC_085236.1 | 963 | 100 | 87.47 |
|  | *Hoya ovalifolia* | NC_069563.1 | 950 | 100 | 87.13 |
|  | *Hoya pubicalyx* | NC_069561.1 | 920 | 100 | 86.70 |
|  | *Hoya megalaster* | MW719063.1 | 1215 | 100 | 89.25 |
|  | *Hoya ignorata* | MW719061.1 | 982 | 100 | 85.62 |
|  | *Hoya omlorii* | MW719060.1 | 1182 | 100 | 88.38 |
|  | *Hoya pandurata* | NC_069562.1 | 1182 | 100 | 89.87 |
|  | *Hoya lithophytica* | MW719058.1 | 1187 | 99 | 87.59 |
|  | *Hoya thomsonii* | NC_067612.1 | 1139 | 100 | 88.14 |
|  | *Hoya kerrii* | NC_069570.1 | 1082 | 97 | 90.48 |
|  | *Hoya lacunosa* | NC_069564.1 | 1045 | 100 | 87.4 |
|  | *Hoya pottsii* | OL754664.1 | 1038 | 98 | 89.18 |
|  | *Hoya silvatica* | NC_067963.1 | 1038 | 98 | 89.18 |
|  | *Hoya commutata* | NC_067958.1 | 1052 | 100 | 86.61 |
| *Dischidia* | *Dischidia australis* | NC_067885.1 | 1065 | 91 | 91.11 |
| *Gymnema* | *Gymnema yunnanense* | NC_079598.1 | 733 | 78 | 86.46 |
| *Papuahoya* | *Papuahoya urniflora* | MW719062.1 | 1086 | 100 | 89.66 |
| *Stephanotis* | *Stephanotis volubilis* | OP133576.1 | 799 | 82 | 88.32 |

# Supplementary

## Dataset of alignment files of six chloroplast intergenic spacer regions (*trnK–rps16, psbI–atpA, trnH–psbA, psbK–psbI, ndhC–trnV,* and *rbcL–accD*), accessible at <https://data.mendeley.com/datasets/tb3yyhx8x6/2>

## Figure S1. Alignment results of 30 *ndhC-trnV* sequences from the studied species

**10 20 30 40 50 60 70**

**....|....|....|....|....|....|....|....|....|....|....|....|....|....|**

**Dischidia australis NC_067885.** **AGACACACTCCCATGAACGTGAAAAATATACCGAATTTCTCGATTCGAATTGTCAAGTCATCCATAACTG** 70

**Gymnema yunnanense NC_079598.1** **AGACACACTCCCATGAACGTGGAAAATATACCGAATTGCTCGATTCGAATTGTCAAGTCATCCATAACTG** 70

**Hoya ariadna NC_069568.1**  **AGACACACTCCCATGAACGTGGAAAATATACCGAATTTCTCGATTCGAATTGTCAAGTCATCCATAACTG** 70

**Hoya commutata NC_067958.1**  **AGACACACTCCCATGAACGTGGAAAATATACCGAATTTCTCGATTCGAATTGTCAAGTCATCCATAACTG** 70

**Hoya dimorpha NC_067959.1**  **AGACACACTCCCATGAACGTGGAAAATATACCGAATTTCTCGATTCGAATTGTCAAGTCATCCATAACTG** 70

**Hoya exilis MW719054.1**  **AGACACACTCCCATGAACGTGGAAAATATACCGAATTTCTCGATTCGAATTGTCAAGTCATCCATAACTG** 70

**Hoya griffithii NC_069565.1**  **AGACACACTCCCATGAACGTGGAAAATATACCGAATTTCTCGATTCGAATTGTCAAGTCATCCATAACTG** 70

**Hoya kerrii NC_069570.1**  **AGACACACTCCCATGAACGTGGAAAATATACCGAATTTCTCGATTCGAATTGTCAAGTCATCCATAACTG** 70

**Hoya lacunosa NC_069564.1**  **AGACACACTCCCATGAACGTGGAAAATATACCGAATTTCTCGATTCGAATTGTCAAGTCATCCATAACTG** 70

**Hoya lanceolata NC_067960.1**  **AGACGCACTCCCATGAACGTGGAAAATATACCGAATTTCTCGATTCGAATTGTCAAGTCATCCATAACTG** 70

**Hoya liangii OL826865.1**  **AGACACACTCCCATGAACGTGGAAAATATACCGAATTTCTCGATTCGAATTGTCAAGTCATCCATAACTG** 70

**Hoya lithophytica MW719058.1**  **AGACACACTCCCATGAACGTGGAAAATATACCGAATTTCTCGATTCGAATTGTCAAGTCATCCATAACTG** 70

**Hoya lockii OR475243.1**  **AGACACACTCCCATGAACGTGGAAAATATACCGAATTTCTCGATTCGAATTGTCAAGTCATCCATAACTG** 70

**Hoya longifolia NC_069560.1**  **AGACACACTCCCATGAACGTGGAAAATATACCGAATTTCTCGATTCGAATTGTCAAGTCATCCATAACTG** 70

**Hoya lyi MW719055.1**  **AGACACACTCCCATGAACGTGGAAAATATACCGAATTTCTCGATTCGAATTGTCAAGTCATCCATAACTG** 70

**Hoya megalaster MW719063.1**  **AGACACACTCCCATGAACGTGGAAAATATACCGAATTTCTCGATTCGAATTGTCAAGTCATCCATAACTG** 70

**Hoya meliflua NC_069571.1**  **AGACACACTCCCATGAACGTGGAAAATATACCGAATTTCTCGATTCGAATTGTCAAGTCATCCATAACTG** 70

**Hoya monetteae MW719053.1**  **AGACACACTCCCATGAACGTGGAAAATATACCGAATTTCTCGATTCGAATTGTCAAGTCATCCATAACTG** 70

**Hoya omlorii MW719060.1**  **AGACACACTCCCATGAACGTGGAAAATATACCGAATTTCTCGATTCGAATTGTCAAGTCATCCATAACTG** 70

**Hoya ovalifolia NC_069563.1**  **AGACACACTCCCATGAACGTGGAAAATATACCGAATTTCTCGATTCGAATTGTCAAGTCATCCATAACTG** 70

**Hoya pandurata NC_069562.1**  **AGACACACTCCCATGAACGTGGAAAATATACCGAATTTCTCGAGTCGAATTGTCAAGTCATCCATAACTG** 70

**Hoya pottsii OL754664.1**  **AGACACACTCCCATGAACGTGGAAAATATACCGAATTTCTCGATTCGAATTGTCAAGTCATCCATAACTG** 70

**Hoya pubicalyx NC_069561.1**  **AGACACACTCCCATGAACGTGGAAAATATACCGAATTTCTCGATTCGAATTGTCAAGTCATCCATAACTG** 70

**Hoya radicalis NC_067961.1**  **AGACACACTCCCATGAACGTGGAAAATATACCGAATTTCTCGATTCGAATTGTCAAGTCATCCATAACTG** 70

**Hoya rigida NC_067962.1**  **AGACACACTCCCATGAACGTGGAAAATATACCGAATTTCTCGATTCGAATTGTCAAGTCATCCATAACTG** 70

**Hoya silvatica NC_067963.1**  **AGACACACTCCCATGAACGTGGAAAATATACCGAATTTCTCGATTCGAATTGTCAAGTCATCCATAACTG** 70

**Hoya thomsonii NC_067612.1**  **AGACACACTCCCATGAACGTGGAAAATATACCGAATTTCTCGATTCGAATTGTCAAGTCATCCATAACTG** 70

**Hoya verticillata NC_085236.1**  **AGACACACTCCCATGAACGTGGAAAATATACCGAATTTCTCGATTCGAATTGTCAAGTCATCCATAACTG** 70

**Papuahoya urniflora MW719062.1** **AGACACACTCCCATGAACGTGGAAAATATACCGAATTGCTCGATTCGAATTGTCAAGTCATCCATAACTG** 70

**Stephanotis volubilis OP133576** **AGACACACTCCCATGAACGTGGAAAATATACCGAATTGCTCGATTCGAATTGTCAAGTCATCCATAACTG** 70

**80 90 100 110 120 130 140**

**....|....|....|....|....|....|....|....|....|....|....|....|....|....|**

**Dischidia australis NC_067885.** **TTTAGTCAAAATAAAAACAAGAATTCATTTTTATCGAATTTACTAGTTTCCTTTGTTTACTTATCCCATT** 140

**Gymnema yunnanense NC_079598.1** **TTTAGTCAAAATCAAAACAAGAATTCATTTTGATCGAATTTACTAGTTTCCTTTGTTTACTTATCCCATT** 140

**Hoya ariadna NC_069568.1**  **TTTAGTCAAAATCAAAACAAGAATTCATTTTTATCGAATTTACTAGTTTCCTTTGTTTACTTATCCCATT** 140

**Hoya commutata NC_067958.1**  **TTTAGTCAAAATCAAAACAAGAATTCATTTTTATCGAATTTACTAGTTTCCTTTGTTTACTTATCCCATT** 140

**Hoya dimorpha NC_067959.1**  **TTTAGTCAAAATCAAAACAAGAATTCATTTTTATCGAATTTACTAGTTTCCTTTGTTTACTTATCCCATT** 140

**Hoya exilis MW719054.1**  **TTTAGTCAAAATCAAAACAAGAATTCATTTTTATCGAATTTACTAGTTTCCTTTGTTTACTTATCCCATT** 140

**Hoya griffithii NC_069565.1**  **TTTAGTCAAAATCAAAACAAGAATTCATTTTTATCGAATTTACTAGTTTCCTTTGTTTACTTATCCCATT** 140

**Hoya kerrii NC_069570.1**  **TTTAGTCAAAATCAAAACAAGAATTCATTTTTATCGAATTTACTAGTTTCCTTTGTTTACTTATCCCATT** 140

**Hoya lacunosa NC_069564.1**  **TTTAGTCAAAATCAAAACAAGAATTAATTTTTATCGAATTTACTAGTTTCCTTTGTTTACTTATCCCATT** 140

**Hoya lanceolata NC_067960.1**  **TTTAGTCAAAATCAAAACAAGAATTCATTTTTATCGAATTTACTAGTTTCCTTTGTTTACTTATCCCATT** 140

**Hoya liangii OL826865.1**  **TTTAGTCAAAATCAAAACAAGAATTCATTTTTATCGAATTTACTAGTTTCCTTTGTTTACTTATCCCATT** 140

**Hoya lithophytica MW719058.1**  **TTTAGTCAAAATCAAAACAAGAATTCATTTTTATCGAATTTACTAGTTTCCTTTGTTTACTTATCCCATT** 140

**Hoya lockii OR475243.1**  **TTTAGTCAAAATCAAAACACGAATTCATTTTTATCGAATTTACTAGTTTCCTTTGTTTACTTATCCCATT** 140

**Hoya longifolia NC_069560.1**  **TTTAGTCAAAATCAAAACAAGAATTCATTTTTATCGAATTTACTAGTTTCCTTTGTTTACTTATCCCATT** 140

**Hoya lyi MW719055.1**  **TTTAGTCAAAATCAAAACAAGAATTCATTTTTATCGAATTTACTAGTTTCCTTTGTTTACTTATCCCATT** 140

**Hoya megalaster MW719063.1**  **TTTAGTCAAAATCAAAACAAGAATTCATTTTTATCGAATTTACTAGTTTCCTTTGTTTACTTATCCCATT** 140

**Hoya meliflua NC_069571.1**  **TTTAGTCAAAATCAAAACAAGAATTCATTTTTATCGAATTTACTAGTTTCCTTTGTTTACTTATCCCATT** 140

**Hoya monetteae MW719053.1**  **TTTAGTCAAAATCAAAACAAGAATTCATTTTTATCGAATTTACTAGTTTCCTTTGTTTACTTATCCCATT** 140

**Hoya omlorii MW719060.1**  **TTTAGTCAAAATCAAAACAAGAATTCATTTTTATCGAATTTACTAGTTTCCTTTGTTTACTTATCCCATT** 140

**Hoya ovalifolia NC_069563.1**  **TTTAGTCAAAATCAAAACAAGAATTCATTTTTATCGAATTTACTAGTTTCCTTTGTTTACTTATCCCATT** 140

**Hoya pandurata NC_069562.1**  **TTTAGTCAAAATCAAAACAAGAATTTATTTTTATCGAATTTACTAGTTTCCTTTGTTTACTTATCCCATT** 140

**Hoya pottsii OL754664.1**  **TTTAGTCAAAATCAAAACAAGAATTCATTTTTATCTAATTTACTAGTTTCCTTTGTTTACTTATCCCATT** 140

**Hoya pubicalyx NC_069561.1**  **TTTAGTCAAAATCAAAACAAGAATTCATTTTTATCGAATTTACTAGTTTCCTTTGTTTACTTATCCCATT** 140

**Hoya radicalis NC_067961.1**  **TTTAGTCAAAATCAAAACAAGAATTCATTTTTATCGAATTTACTAGTTTCCTTTGTTTACTTATCCCATT** 140

**Hoya rigida NC_067962.1**  **TTTAGTCAAAATCAAAACAAGAATTCATTTTTATCGAATTTACTAGTTTCCTTTGTTTACTTATCCCATT** 140

**Hoya silvatica NC_067963.1**  **TTTAGTCAAAATCAAAACAAGAATTCATTTTTATCTAATTTACTAGTTTCCTTTGTTTACTTATCCCATT** 140

**Hoya thomsonii NC_067612.1**  **TTTAGTCAAAATCAAAACAAGAATTCATTTTTATCGAATTTACTAGTTTCCTTTGTTTACTTATCCCATT** 140

**Hoya verticillata NC_085236.1**  **TTTAGTCAAAATCAAAACAAGAATTCATTTTTATCGAATTTACTAGTTTCCTTTGTTTACTTATCCCATT** 140

**Papuahoya urniflora MW719062.1** **TTTAGTCAAAATCAAAACAAGAATTCATTTTTATCGAATTTACTAGTTTCCTTTGTTTACTTATCCCATT** 140

**Stephanotis volubilis OP133576** **TTTAGTCAAAATCAAAACAAGAATTCATTTTGATCGAATTTACTAGTTTCCTTTGTTTACTTATCCCATT** 140

**150 160 170 180 190 200 210**

**....|....|....|....|....|....|....|....|....|....|....|....|....|....|**

**Dischidia australis NC_067885.** **TCAAGACAAAATCCTATTTAGATTATACTTATTTCTATTTTTTCAGTTAGTATAACTTTAGATCTAT---** 207

**Gymnema yunnanense NC_079598.1** **TCAAGACAAAATCCTATTTAGATTATACTTATTTCTATTTTTT-AGTTAGTATAACTTTAGATCTATATC** 209

**Hoya ariadna NC_069568.1**  **TCAAGACAAAATCCTATTTAGATTATACTTATTTCTATTTTTTCAGTTAGTATAACTTTAGATCTATATC** 210

**Hoya commutata NC_067958.1**  **TCAAGACAAAATCCTATTTAGATTTTACTTATTTCTTTTTTTTCAGTTAGTATAACTTTAGATCTAT---** 207

**Hoya dimorpha NC_067959.1**  **TCAAGACAAAATCCTATTTAGATTATACTTCTTTCTATTTTTTCAGTTAGTATAACTTTAGATCTAT---** 207

**Hoya exilis MW719054.1**  **TCAAGACAAAATCCTATTTAGATTATACTTCTTTCTATTTTTTCAGTTAGTATAACTTTAGATCTATATC** 210

**Hoya griffithii NC_069565.1**  **TCAAGACAAAATCCTATTTAGATTATACTTATTTCTATTTTTTCAGTTAGTATAACTTTAGATCTAT---** 207

**Hoya kerrii NC_069570.1**  **TCAAGACAAAATCCTATTTAGATTATACTTATTTCTATTTTTTCAGTTAGTATAACTTTAGATCTAT---** 207

**Hoya lacunosa NC_069564.1**  **TCAAGACAAAATCCTATTTAGATTATACTTATTTCTTTTTTTTCAGTTAGTATAACTTTAGATCTAT---** 207

**Hoya lanceolata NC_067960.1**  **TCAAGACAAAATCCTATTTAGATTATACTTATTTCTATTTTTTCAGTTAGTATAACTTTAGATCTAT---** 207

**Hoya liangii OL826865.1**  **TCAAGACAAAATCCTATTTAGATTATACTTATTTCTATTTTTTCAGTTAGTATAACTTTAGATCTAT---** 207

**Hoya lithophytica MW719058.1**  **TCAAGACAAAATCCTATTTAGATTATACTTATTTCTATTTTTTCAGTTAGTATAACTTTAGATCTAT---** 207

**Hoya lockii OR475243.1**  **TCAAGACAAAATCCTATTTAGATTATACTTCTTTCTATTTTTTCAGTTAGTATAACTTTAGATCTATATC** 210

**Hoya longifolia NC_069560.1**  **TCAAGACAAAATCCTATTTAGATTATACTTATTTCTATTTTTTCAGTTAGTATAACTTTAGATCTAT---** 207

**Hoya lyi MW719055.1**  **TCAAGACAAAATCCTATTTAGATTATACTTATTTCTATTTTTTCAGTTAGTATAACTTTAGATCTAT---** 207

**Hoya megalaster MW719063.1**  **TCAAGACAAAATCCTATTTAGATTATACTTCTTTCTATTTTTTCAGTTAGTATAACTTTAGATCTAT---** 207

**Hoya meliflua NC_069571.1**  **TCAAGACAAAATCCTATTTAGATTATACTTATTTCTATTTTTTCAGTTAGTATAACTTTAGATCTAT---** 207

**Hoya monetteae MW719053.1**  **TCAAGACAAAATCCTATTTAGATTATACTTATTTCTATTTTTTCAGTTAGTATAACTTTAGATCTAT---** 207

**Hoya omlorii MW719060.1**  **TCAAGACAAAATCCTATTTAGATTATACTTATTTCTATTTTTTCAGTTAGTATAACTTTAGATCTAT---** 207

**Hoya ovalifolia NC_069563.1**  **TCAAGACAAAATCCTATTTAGATTATACTTATTTCTTTTTTTTCAGTTAGTATAACTTTAGATCTAT---** 207

**Hoya pandurata NC_069562.1**  **TCAAGACAAAATCCTATTTAGATTATACTTATTTCTATTTTTTCAGTTAGTATAACTTTAGATCTAT---** 207

**Hoya pottsii OL754664.1**  **TCAAGACAAAATCCTATTTAGATTTTACTTATTTCTTTTTTTTCAGTTAGTATAACTTTAGATCTAT---** 207

**Hoya pubicalyx NC_069561.1**  **TCAAGACAAAATCCTATTTAGATTATACTTATTTCTATTTTTTCAGTTAGTATAACTTTAGATCTAT---** 207

**Hoya radicalis NC_067961.1**  **TCAAGACAAAATCCTATTTAGATTATACTTATTTCTATTTTTTCAGTTAGTATAACTTTAGATCTAT---** 207

**Hoya rigida NC_067962.1**  **TCAAGACAAAATCCTATTTAGATTATACTTATTTCTATTTTTTCAGTTAGTATAACTTTAGATCTAT---** 207

**Hoya silvatica NC_067963.1**  **TCAAGACAAAATCCTATTTAGATTTTACTTATTTCTTTTTTTTCAGTTAGTATAACTTTAGATCTAT---** 207

**Hoya thomsonii NC_067612.1**  **TCAAGACAAAATCCTATTTAGATTATACTTATTTCTATTTTTTCAGTTAGTATAACTTTAGATCTAT---** 207

**Hoya verticillata NC_085236.1**  **TCAAGACAAAATCCTATTTAGATTATACTTATTTCTTTTTTTTCAGTTAGTATAACTTTAGATCTAT---** 207

**Papuahoya urniflora MW719062.1** **TCAAGACAAAATCCTATTTAGATTATACTTATTTCTATTTTTTCAGTTAGTATAACTTTAGATCTAT---** 207

**Stephanotis volubilis OP133576** **TCAAGACAAAATCCTATTTAGATTATACTTATTTCTATTTTTTTAGTTAGTATAACTTTAGATCTA----** 206

**220 230 240 250 260 270 280**

**....|....|....|....|....|....|....|....|....|....|....|....|....|....|**

**Dischidia australis NC_067885.** **--------------------------ATCTTATTCTTA--TACAAA------------------------** 225

**Gymnema yunnanense NC_079598.1** **TTATTCTTATACATTATAAAAACTATATCTTATTCTTA--TCCAAA------------------------** 253

**Hoya ariadna NC_069568.1**  **TTATTCTT------------------ATACAAATATAAAATACAAA------------------------** 238

**Hoya commutata NC_067958.1**  **--------------------------ATCTTATTCTTA--TACAAA------------------------** 225

**Hoya dimorpha NC_067959.1**  **--------------------------ATCTTATTCTTA--TACAAA------------------------** 225

**Hoya exilis MW719054.1**  **TTATTCTTATACAA----------ATATAAAAT-------------------------------------** 233

**Hoya griffithii NC_069565.1**  **--------------------------ATCTTATTCTTA--GACAAA------------------------** 225

**Hoya kerrii NC_069570.1**  **--------------------------ATCTTATTCTTA--TACAAA------------------------** 225

**Hoya lacunosa NC_069564.1**  **--------------------------ATCTTATTCTTA--TACAAA------------------------** 225

**Hoya lanceolata NC_067960.1**  **--------------------------ATCTTATTCTTA--TACAAA------------------------** 225

**Hoya liangii OL826865.1**  **--------------------------ATCTTATTCTTA--GACAAA------------------------** 225

**Hoya lithophytica MW719058.1**  **--------------------------ATCTTATTCTTA--TACAAA------------------------** 225

**Hoya lockii OR475243.1**  **TTATTCTTATACAA----------ATATAAAATTCTTA--TACAAA------------------------** 244

**Hoya longifolia NC_069560.1**  **--------------------------ATCTTATTCTTA--GACAAA------------------------** 225

**Hoya lyi MW719055.1**  **--------------------------ATCTTATTCTTA--TACAAA------------------------** 225

**Hoya megalaster MW719063.1**  **--------------------------ATCTTATTCTTA--TACAAA------------------------** 225

**Hoya meliflua NC_069571.1**  **--------------------------ATCTTATTCTTA--TACAAA------------------------** 225

**Hoya monetteae MW719053.1**  **--------------------------ATCTTATTCTTA--TACAAA------------------------** 225

**Hoya omlorii MW719060.1**  **--------------------------ATCTTATTCTTA--TACAAA------------------------** 225

**Hoya ovalifolia NC_069563.1**  **--------------------------ATCTTATTCTTA--TACAAA------------------------** 225

**Hoya pandurata NC_069562.1**  **--------------------------ATCTTATTCTTA--GACAAA------------------------** 225

**Hoya pottsii OL754664.1**  **--------------------------ATCTTATTCTTA--TACAAA------------------------** 225

**Hoya pubicalyx NC_069561.1**  **--------------------------ATCTTATTCTTA--TACAAA------------------------** 225

**Hoya radicalis NC_067961.1**  **--------------------------ATCTTATTCTTA--GACAAA------------------------** 225

**Hoya rigida NC_067962.1**  **--------------------------ATCTTATTCTTA--TACAAA------------------------** 225

**Hoya silvatica NC_067963.1**  **--------------------------ATCTTATTCTTA--TACAAA------------------------** 225

**Hoya thomsonii NC_067612.1**  **--------------------------ATCTTATTCTTA--GACAAA------------------------** 225

**Hoya verticillata NC_085236.1**  **--------------------------ATCTTATTCTTA--TACAAA------------------------** 225

**Papuahoya urniflora MW719062.1** **--------------------------ATCTTATTCTTA--TACAAAATCTATATCTTATTCTTATACAAA** 249

**Stephanotis volubilis OP133576** **--------------------------ATCTTATTCTTA--TACAAA------------------------** 224

**290 300 310 320 330 340 350**

**....|....|....|....|....|....|....|....|....|....|....|....|....|....|**

**Dischidia australis NC_067885.** **TATAAAATAGAAAAATTCTCTTATTTTCACCTAGAATTCTCTGTAA-------AGAAGAAAAGGAATTCA** 288

**Gymnema yunnanense NC_079598.1** **TA--AAATATACAAATTCTCTTATTTTCACCTAGAATTCTCTGTAA-------AGAAGAAAAGGAATTCA** 314

**Hoya ariadna NC_069568.1**  **TATAAAATAGAAAAATTCTCTTATTTTCACCTAGAATTCTCTGTAA-------AGAAGAAAAGGAATTCA** 301

**Hoya commutata NC_067958.1**  **TATAAAATAGAAAAATTCTCTTATTTTCACCTAGAATTCTCTGTAA-------AGAAGAAAAGGAATTCA** 288

**Hoya dimorpha NC_067959.1**  **TATAAAATAGAAAAATTCTCTTATTTTCACCTAGAATTCTCTGTAA-------AGAAGAAAAGGAATTCA** 288

**Hoya exilis MW719054.1**  **--------AGAAAAATTCTCTTATTTTAACCTAGAATTCTCTGTAA-------AGAAGAAAAGGAATTCA** 288

**Hoya griffithii NC_069565.1**  **TATAAAATAGAAAAATTCTCTTATTTTCACCTAGAATTCTCTGTAA-------AGAAGAAAAGGAATTCA** 288

**Hoya kerrii NC_069570.1**  **TATAAAATAGAAAAATTCTCTTATTTTCACCTAGAATTCTCTGTAA-------AGAAGAAAAGGAATTCA** 288

**Hoya lacunosa NC_069564.1**  **TATAAAATAGAAAAATTCTCTTATTTTCACCTAGAATTCTCTGTAA-------AGAAGAAAAGGAATTCA** 288

**Hoya lanceolata NC_067960.1**  **---------TAGAAATT-----------------------------------------------------** 233

**Hoya liangii OL826865.1**  **TATAAAATAGAAAAATTCTCTTATTTTCACCTAGAATTCTCTGTAA-------AGAAGAAAAGGAATTCA** 288

**Hoya lithophytica MW719058.1**  **TATAAAATAGAAAAATTCTCTTATTTTCACCTAGAATTCTCTGTAA-------AGAAGAAAAGGAATTCA** 288

**Hoya lockii OR475243.1**  **TATAAAATAGAAAAATTCTCTTATTTTAACCTAGAATTCTCTGTAATCTGTAAAGAAGAAAAAGAATTCA** 314

**Hoya longifolia NC_069560.1**  **TATAAAATAGAAAAATTCTCTTATTTTCACCTAGAATTCTCTGTAA-------AGAAGAAAAGGAATTCA** 288

**Hoya lyi MW719055.1**  **TATAAAATAGAAAAATTCTCTTATTTTCACCTAGAATTCTCTGTAA-------AGAAGAAAAGGAATTCA** 288

**Hoya megalaster MW719063.1**  **TATAAAATAGAAAAATTCTCTTATTTTCACCTAGAATTCTCTGTAA-------AGAAGAAAAGGAATTCA** 288

**Hoya meliflua NC_069571.1**  **TATAAAATAGAAAAATTCTCTTATTTTCACCTAGAATTCTCTGTAA-------AGAAGAAAAGGAATTCA** 288

**Hoya monetteae MW719053.1**  **TATAAAATAGAAAAATTCTCTTATTTTCACCTAGAATTCTCTGTAA-------AGAAGAAAAGGAATTCA** 288

**Hoya omlorii MW719060.1**  **TATAAAATAGAAAAATTCTCTTATTTTGACCTAGAATTCTCTGTAA-------AGAAGAAAAGGAATTCA** 288

**Hoya ovalifolia NC_069563.1**  **TATAAAATAGAAAAATTCTCTTATTTTCACCTAGAATTCTCTGTAA-------AGAAGAAAAGGAATTCA** 288

**Hoya pandurata NC_069562.1**  **TATAAAATAGAAAAATTCTCTTATTTTCACCTAGAATTCTCTGTAA-------AGAAGAAAAGGAATTCA** 288

**Hoya pottsii OL754664.1**  **TATAAAATAGAAAAATTCTCTTATTTTCACCTAGAATTCTCTGTAA-------AGAAGAAAAGGAATTCA** 288

**Hoya pubicalyx NC_069561.1**  **TATAAAATAGAAAAATTCTCTTATTTTCACCTAGAATTCTCTGTAA-------AGAAGAAAAGGAATTCA** 288

**Hoya radicalis NC_067961.1**  **TATAAAATAGAAAAATTCTCTTATTTTCACCTAGAATTCTCTGTAA-------AGAAGAAAAGGAATTCA** 288

**Hoya rigida NC_067962.1**  **TATAAAATAGAAAAATTCTCTTATTTTCACCTAGAATTCTCTGTAA-------AGAAGAAAAGGAATTCA** 288

**Hoya silvatica NC_067963.1**  **TATAAAATAGAAAAATTCTCTTATTTTCACCTAGAATTCTCTGTAA-------AGAAGAAAAGGAATTCA** 288

**Hoya thomsonii NC_067612.1**  **TATAAAATAGAAAAATTCTCTTATTTTCACCTAGAATTCTCTGTAA-------AGAAGAAAAGGAATTCA** 288

**Hoya verticillata NC_085236.1**  **TATAAAATAGAAAAATTCTCTTATTTTCACCTAGAATTCTCTGTAA-------AGAAGAAAAGGAATTCA** 288

**Papuahoya urniflora MW719062.1** **TATAAAATATAAAAATTTTATTATTTTCACCTAGAATTCTCTGTAA-------AGAAGAAAAGGAATTAA** 312

**Stephanotis volubilis OP133576** **TA--AAATATACAAATTCTCTTATTTTCACCTAGAATTCTCTGTAA-------AGAAGAAAAGGAATTCA** 285

**360 370 380 390 400 410 420**

**....|....|....|....|....|....|....|....|....|....|....|....|....|....|**

**Dischidia australis NC_067885.** **AAAAAATATGGAATTCCCCTTGGAATTTTGAACTTCTTTATTTTTT---TATTTTT-----------AAA** 344

**Gymnema yunnanense NC_079598.1** **AAAAAATATGGAATTCCCTTTGGAATTTGGAACTTCTTTATTTTTT---T--TTTT-----------TAA** 368

**Hoya ariadna NC_069568.1**  **AAAAAATATGGAATTCCCCTTGGAATTTTGAACTTCTTTATTTATT---TATTTTT-----------GAA** 357

**Hoya commutata NC_067958.1**  **AAAAAATATGGAATTCCCCTTGGAATTTTGAACTTCTTTATTTATT---TATTTTT-----------TTT** 344

**Hoya dimorpha NC_067959.1**  **AAAAAATATGGAATTCCCCTTGGAATTTGGAACTTCTTTATTTATT---TATTTTT-----------TAA** 344

**Hoya exilis MW719054.1**  **AAAAAATATGGAATTCCCCTTGGAATTTGGAACTTCTTTATTTATT---TATTTTT-----------GAA** 344

**Hoya griffithii NC_069565.1**  **AAAAAATATGGAATTCCCCTTGGAATTTGGAACTTCTTTATTTATT---TTTTTTT-----------TAA** 344

**Hoya kerrii NC_069570.1**  **AAAAAATATGGAATTCCCCTTGGAATTTGGAACTTCTTTATTTATT---TTTTTATTTATTTTTATTTAA** 355

**Hoya lacunosa NC_069564.1**  **AAAAAATATGGAATTCCACTTGGAATTTGGAACTTCTTTTTTTATT---TATTTTT-----------TAA** 344

**Hoya lanceolata NC_067960.1**  **---------------------GGAATTTGGAACTTCTTTATTTATT---TATTTTT-----------TAA** 268

**Hoya liangii OL826865.1**  **AAAAAATATGGAATTCCCCTTGGAATTTGGAACTTCTTTATTTATT---TTTTTTT-----------TAA** 344

**Hoya lithophytica MW719058.1**  **AAAAAATATGGAATTCCCCTTGGAATTTTGAACTTCTTGATTTATT---TTTTTTT-----------TAA** 344

**Hoya lockii OR475243.1**  **AAAAAATATGGAATTCCCCTTGGAATTTGAAACTTCTTTATCTATTATATATTTCT-----------ATT** 373

**Hoya longifolia NC_069560.1**  **AAAAAATATGGAATTCCCCTTGGAATTTGGAACTTCTTTATTTATT---TTTTTTT-----------TAA** 344

**Hoya lyi MW719055.1**  **AAAAAATATGGAATTCCCCTTGGAATTTGGAACTTCTTTATTTATT---TTTTTTT-----------TAA** 344

**Hoya megalaster MW719063.1**  **AAAAAATATGGAATTCCCCTTGGAATTTGGAACTTCTTTATTTATT---TATTTTT------------AA** 343

**Hoya meliflua NC_069571.1**  **AAAAAATATGGAATTCCCCTTGGAATTTGGAACTTCTTTATTTATT---T-TTTTT-----------TAA** 343

**Hoya monetteae MW719053.1**  **AAAAAATATGGAATTCCCCTTGGAATTTTGAACTTCTTTATTTATT---TATTTTT-----------TAA** 344

**Hoya omlorii MW719060.1**  **AAAAAATATGGAATTCCCCTTGGAATTTGGAACTTCTTTATTTATT---TATTTTT-----------AAA** 344

**Hoya ovalifolia NC_069563.1**  **AAAAAATATGGAATTCCAAGGGGAATTTTGAACTTCTTTATTTATT---TTTTTTTT----------TAA** 345

**Hoya pandurata NC_069562.1**  **AAAAAATATGGAATTCCCCTTGGAATTTTGAACTTCTTTATTTATT---TATTTTT-----------TAA** 344

**Hoya pottsii OL754664.1**  **AAAAAATATGGAATTCCCCTTGGAATTTTGAACTTCTTTATTTATT---TATTTTT-----------TAA** 344

**Hoya pubicalyx NC_069561.1**  **AAAAAATATGGAATTCCCCTTGGAATTTGGAACTTCTTTATTTATT---TATTTTT-----------TAA** 344

**Hoya radicalis NC_067961.1**  **AAAAAATATGGAATTCCCCTTGGAATTTGGAACTTCTTTATTTATT---TATTTTT-----------TAA** 344

**Hoya rigida NC_067962.1**  **AAAAAATATGGAATTCCCCTTGGAATTTGGAACTTCTTTATTTATT---T-----T-----------TAA** 339

**Hoya silvatica NC_067963.1**  **AAAAAATATGGAATTCCCCTTGGAATTTTGAACTTCTTTATTTATT---TATTTTT-----------TAA** 344

**Hoya thomsonii NC_067612.1**  **AAAAAATATGGAATTCCCCTTGGAATTTGGAACTTCTTTATTTATT---TATTTTT-----------TAA** 344

**Hoya verticillata NC_085236.1**  **AAAAAATATGGAATTCCAAGGGGAATTTGGAACTTCTTTATTTATT---TTTTTT------------TAA** 343

**Papuahoya urniflora MW719062.1** **AAAAAATATGGAATTCCCCTTGGAATTTGGAACTTCTTTATTTATT---TTTTTTT-----------TTA** 368

**Stephanotis volubilis OP133576** **AAAAAATATGGAATTCCCCTTGGAATTTGGAACTTCTTTATTTATT---TATTTTT-----------TAA** 341

**430 440 450 460 470 480 490**

**....|....|....|....|....|....|....|....|....|....|....|....|....|....|**

**Dischidia australis NC_067885.** **TTAAATAATTA-----TTATTATTATCTAT-TATATATTTATCTATTATAA-------------------** 389

**Gymnema yunnanense NC_079598.1** **TTAAAAAATT-----ATTATTACTATCTAT-TATATATTT------------------------------** 402

**Hoya ariadna NC_069568.1**  **TTAAATAATT-----ATTATTACTATCTAT-TATATATTTATC---------------------------** 394

**Hoya commutata NC_067958.1**  **TTTTAA--TTT-----TTATTACTATCTAT-TATATATTT-----------------A------------** 377

**Hoya dimorpha NC_067959.1**  **TTAAATAATT-----ATTATTACTATCTAT-TATATATTTATC---------------------------** 381

**Hoya exilis MW719054.1**  **TTAAATAATT-----ATTATTACTATCTAT-TATATATTTATCTAT------------------------** 384

**Hoya griffithii NC_069565.1**  **TTAAATAATTT-----TTATTACTATCTAT-TATATATTT------------------------------** 378

**Hoya kerrii NC_069570.1**  **TTAAATAATTT-----TTATTACTATCTAT-TATATATTT------------------------------** 389

**Hoya lacunosa NC_069564.1**  **TTAAAAAATTT-----TTATTACTATCTAT-TATATATTACTATCTATTATATATTTAGA----------** 398

**Hoya lanceolata NC_067960.1**  **TTAAATAATTA-----TTATTACTATCTAT-TATATATTT------------------------------** 302

**Hoya liangii OL826865.1**  **TTAAATAATTT-----TTATTACTATCTAT-TATATATTT------------------------------** 378

**Hoya lithophytica MW719058.1**  **TTAAATAATT-----ATTATTACTATCTAT-TATATATTTATAAATAAAATTTATAAATAAAATAATTAT** 408

**Hoya lockii OR475243.1**  **TTTTATATTTTATATATTAATTATATATATATATATATATATATAT------------------------** 419

**Hoya longifolia NC_069560.1**  **TTAAATAATTT-----TTATTACTATCTAT-TATATATTT------------------------------** 378

**Hoya lyi MW719055.1**  **TTAAATAATTT-----TTATTACTATCTAT-TATATTTTATTA-CTATC---------------------** 386

**Hoya megalaster MW719063.1**  **TTAAATAATT-----ATTATTACTATCTAT-TATATATTTATC---------------------------** 380

**Hoya meliflua NC_069571.1**  **TTAAAAAATTT-----TTATTACTATCTAT-TATATATTT------------------------------** 377

**Hoya monetteae MW719053.1**  **TTAAAAAATTG-----TTATTACTATCTAT-TATATATTT------------------------------** 378

**Hoya omlorii MW719060.1**  **TTAAATAATT-----ATTATTACTATCTAT-TATATATTTATC---------------------------** 381

**Hoya ovalifolia NC_069563.1**  **TTAAAAAATTT-----TTATTACTATCTAT-TATATATTT------------------------------** 379

**Hoya pandurata NC_069562.1**  **TTAAATAATTT-----TTATTACTATCTAT-TATATATTT------------------------------** 378

**Hoya pottsii OL754664.1**  **TTAAAAAATTT-----TTATTACTATCTAT-TATATATTT-----------------A------------** 379

**Hoya pubicalyx NC_069561.1**  **TTAAATAATTT-----TTATTACTAT--------------------------------------------** 365

**Hoya radicalis NC_067961.1**  **TTAAAAAATTT-----TTATTACTATCTAT-TATATATTT------------------------------** 378

**Hoya rigida NC_067962.1**  **TTAAATAATTT-----TTATTACTATCTAT-TATATATTT------------------------------** 373

**Hoya silvatica NC_067963.1**  **TTAAAAAATTT-----TTATTACTATCTAT-TATATATTT-----------------A------------** 379

**Hoya thomsonii NC_067612.1**  **TTAAATAAATT-----TTATTACTATCTAT-TATATATTT------------------------------** 378

**Hoya verticillata NC_085236.1**  **TTAAATAATTT-----TTATTACTATCTAT-TATATATTT------------------------------** 377

**Papuahoya urniflora MW719062.1** **TTAAATAATTA-----TTATTACTATCTAT-TATATATTT------------------------------** 402

**Stephanotis volubilis OP133576** **TTAAATAATT-----ATTATTACTATCTAT-TATATATTT------------------------------** 375

**500 510 520 530 540 550 560**

**....|....|....|....|....|....|....|....|....|....|....|....|....|....|**

**Dischidia australis NC_067885.** **-------------------------------------------------------------ATTTATTAT** 398

**Gymnema yunnanense NC_079598.1** **----------------------------------------------------------------------** 402

**Hoya ariadna NC_069568.1**  **----------------------------------------------------------------TATTAT** 400

**Hoya commutata NC_067958.1**  **--------------------------------------------------------------TATATTAT** 385

**Hoya dimorpha NC_067959.1**  **----------------------------------------------------------------TATGAT** 387

**Hoya exilis MW719054.1**  **--------------------------------------------------------------TATATC--** 390

**Hoya griffithii NC_069565.1**  **-------------------------------------------------------------ATCTATTAT** 387

**Hoya kerrii NC_069570.1**  **-------------------------------------------------------------ATCTATTAT** 398

**Hoya lacunosa NC_069564.1**  **-------------------------------------------------TATTATATAAATATCTATTAT** 419

**Hoya lanceolata NC_067960.1**  **-------------------------------------------------------------ATCTATTAT** 311

**Hoya liangii OL826865.1**  **-------------------------------------------------------------ATCTATTAT** 387

**Hoya lithophytica MW719058.1**  **TATTACTATCTATTATATATTTATAAATAAAATAATTATTATTACTATCTATTATATATTTATATATTAT** 478

**Hoya lockii OR475243.1**  **--------------------------------------------------------------TATACTAT** 427

**Hoya longifolia NC_069560.1**  **-------------------------------------------------------------ATCTATTAT** 387

**Hoya lyi MW719055.1**  **-------------------------------------------------TATTATATATTTATCTATTAT** 407

**Hoya megalaster MW719063.1**  **----------------------------------------------------------------TATGAT** 386

**Hoya meliflua NC_069571.1**  **-------------------------------------------------------------ATCTATTA-** 385

**Hoya monetteae MW719053.1**  **-------------------------------------------------------------ATCTATTAT** 387

**Hoya omlorii MW719060.1**  **----------------------------------------------------------------TATTAT** 387

**Hoya ovalifolia NC_069563.1**  **-------------------------------------------------------------ATCTATTAT** 388

**Hoya pandurata NC_069562.1**  **-------------------------------------------------------------ATCTATTAT** 387

**Hoya pottsii OL754664.1**  **--------------------------------------------------------------TCTATAAT** 387

**Hoya pubicalyx NC_069561.1**  **----------------------------------------------------------------------** 365

**Hoya radicalis NC_067961.1**  **-------------------------------------------------------------ATCTATTAT** 387

**Hoya rigida NC_067962.1**  **-------------------------------------------------------------ATCTATTAT** 382

**Hoya silvatica NC_067963.1**  **--------------------------------------------------------------TCTATAAT** 387

**Hoya thomsonii NC_067612.1**  **-------------------------------------------------------------CTATTTTTT** 387

**Hoya verticillata NC_085236.1**  **-------------------------------------------------------------ATCTATTAT** 386

**Papuahoya urniflora MW719062.1** **-------------------------------------------------------------ATCTATTAT** 411

**Stephanotis volubilis OP133576** **----------------------------------------------------------------------** 375

**570 580 590 600 610 620 630**

**....|....|....|....|....|....|....|....|....|....|....|....|....|....|**

**Dischidia australis NC_067885.** **ATATTTCTAT----------TTTTTGAAATTGATGAAATTGAATAAAATCAATTGAAATAAATGAAA---** 455

**Gymnema yunnanense NC_079598.1** **------CTAT----------TTTTTGAAATTGATGAAATTGAATAAAATCAATTTCAATAAATGATAAAT** 456

**Hoya ariadna NC_069568.1**  **ATATTTCTAT----------TTTTTTCAATTGATGAAATTGAATAAAATCAATTGAAATAAATG------** 454

**Hoya commutata NC_067958.1**  **ATATTTCTAT----------TTTTTGAAATTGATGAAATTGAATAAAATCAATTGAAATAAATGAA----** 441

**Hoya dimorpha NC_067959.1**  **ATATTTCTAT----------TTTTTGAAATTGATGAAATTGAATAAAATCAATTGAAATAAATG------** 441

**Hoya exilis MW719054.1**  **TTTATA---T----------TTTTTGAAATTGATGAAATTGAATAAAATCAATTGAAATAAATGA-----** 442

**Hoya griffithii NC_069565.1**  **ATATTTCTAT----------TTTTTGAAATTGATGAAA---------------TTGAATAAAATCA----** 428

**Hoya kerrii NC_069570.1**  **ATATTTATAT----------TTTTTGAAATTGATGAAATTGAATAAAATCAATTGAAATAAATGAA----** 454

**Hoya lacunosa NC_069564.1**  **ATATTTCTAT----------TTTTTGAAATTGATGAAATTGAATAAAATCAATTGAAATAAATGAA----** 475

**Hoya lanceolata NC_067960.1**  **ATATATATATTTCTA----TTTTTTGAAATTGATGAAATTGAATAAAATCAATTGAAATAAAAAAA----** 373

**Hoya liangii OL826865.1**  **ATATTTCTAT----------TTTTTGAAATTGATGAAA---------------TTGAATAAAATCA----** 428

**Hoya lithophytica MW719058.1**  **ATATTTCTAT----------TTTTTTAAATTGATGAAATTGAATAAAATCAATTGAAATAAAT-------** 531

**Hoya lockii OR475243.1**  **TTTATACTAT----------TTTTTGAAATTGATGAAATTTAATAAAATCAATTGAAATAAATGA-----** 482

**Hoya longifolia NC_069560.1**  **ATATTTCTAT----------TTTTTGAAATTGATGAAA---------------TTGAATAAAATCA----** 428

**Hoya lyi MW719055.1**  **ATATTTCTAT----------TTTTTGAAATTGATGAAATTGAATAAAATCAATTGAAATAAATGAA----** 463

**Hoya megalaster MW719063.1**  **ATATTTCTAT----------TTTTTGAAATTGATGAAATTGAATAAAATCAATTGAAATAAATG------** 440

**Hoya meliflua NC_069571.1**  **----------------------------------------------------------------------** 385

**Hoya monetteae MW719053.1**  **ATATTTATAT----------TTTTTGAAATTGATGAAATTGAATAAAATCAATTCAAATAAATGAA----** 443

**Hoya omlorii MW719060.1**  **ATATTTCTAT----------TTTTTTAAATTGATGAAATTGAATAAAATCAATTGAAATAAATG------** 441

**Hoya ovalifolia NC_069563.1**  **ATATTTATAT----------TTTTTGAAATTGATGAAATTGAATAAAATCAATTGAAATAAATGAA----** 444

**Hoya pandurata NC_069562.1**  **ATATTTCTAT----------TTTTTGAAATTGATGAAATTGAAT---------TTCAATAAATGAA----** 434

**Hoya pottsii OL754664.1**  **ATATTTATAT----------TTTTTGAAATTGATGAAATTGAATAAAATCAATTGAAATAAATGAA----** 443

**Hoya pubicalyx NC_069561.1**  **-----------------------------------------------------TGAAATAAATGAA----** 378

**Hoya radicalis NC_067961.1**  **ATATTTCTAT----------TTTTTGAAATTGATGAAA---------------TTGAATAAAATCA----** 428

**Hoya rigida NC_067962.1**  **ATATTTCTATATATTTCTATTTTTTGAAATTGATGAAATTGAATAAAAAAAATTTCAATAAATGAA----** 448

**Hoya silvatica NC_067963.1**  **ATATTTATAT----------TTTTTGAAATTGATGAAATTGAATAAAATCAATTGAAATAAATGAA----** 443

**Hoya thomsonii NC_067612.1**  **GAAATTGATG----------AAATTGAATAAAATCAAT---------------TTCAATAAATGAA----** 428

**Hoya verticillata NC_085236.1**  **ATATTTATAT----------TTTTTGAAATTGATGAAATTGAATAAAATCAATTGAAATAAATGAA----** 442

**Papuahoya urniflora MW719062.1** **ATATTTCTATTTTT-----TTTTTTGAAATTGATGAAATTGAATAAAATCAATTGAAATAAATGAA----** 472

**Stephanotis volubilis OP133576** **------CTAT----------TTTTTGAAATTGATGAAATTGAATAAAATCAATTGAAATAAA-------T** 422

**640 650 660 670 680 690 700**

**....|....|....|....|....|....|....|....|....|....|....|....|....|....|**

**Dischidia australis NC_067885.** **AAAAAAAAAATA------GAATTCAAATAAGAAAATATA----------------------GATTATTTC** 497

**Gymnema yunnanense NC_079598.1** **GAAAAAAAAATC------GAATTCAAATAAGAAAATATA----------------------GATTATTTC** 498

**Hoya ariadna NC_069568.1**  **AAAAAAAAAATA------GAATTCAAATAAGAAAATAAAAAAATATA--------------GATTATTTC** 504

**Hoya commutata NC_067958.1**  **AAAAAAA--ATATATATATAATTCAAATAAAAAAATATA----------------------GATTATTTC** 487

**Hoya dimorpha NC_067959.1**  **AAAAAAAAAATA------TAATTCAAATAATAAAATATA----------------------GATTATTTC** 483

**Hoya exilis MW719054.1**  **AAAAAAAATATA------TAATTCAAATAATAAAATATA----------------------GATTATTTC** 484

**Hoya griffithii NC_069565.1**  **ATTTCAAT--AA------AT-----GAAAAAAAAATATA----------------------GATTATTTC** 463

**Hoya kerrii NC_069570.1**  **AAAAA----ATA------TAATTCAAATAAAAAAATATA----------------------GATTATTTC** 492

**Hoya lacunosa NC_069564.1**  **AAAATAT--ATA----TATAATTCAAATAAAAAAATATA----------------------GATTATTTC** 517

**Hoya lanceolata NC_067960.1**  **AAAAAAAAAAAAAA----TAATTCAAATAATAAAATATA----------------------GATTATTTC** 417

**Hoya liangii OL826865.1**  **ATTTCAAT--AA------AT-----GAAAAAAAAATATA----------------------GATTATTTC** 463

**Hoya lithophytica MW719058.1**  **-GAAAAAAAATA------GAATTCAAATAAGAAAATATAAAAATATAGATTATTTATTATAGATTATTTA** 594

**Hoya lockii OR475243.1**  **AAAAAA--TATT------TAATTCAAATAAAAAAATATA----------------------GATTATTTC** 522

**Hoya longifolia NC_069560.1**  **ATTTCAAT--AA------AT-----GAAAAAAAAATATA----------------------GATTATTTC** 463

**Hoya lyi MW719055.1**  **AAAAAAA--AAAAA-GTATAATTCAAATAAAAAAATATA----------------------GATTATTTC** 508

**Hoya megalaster MW719063.1**  **AWAAAAATA-TA------TAATTCAAATAATAAAATATA----------------------GATTATTTC** 481

**Hoya meliflua NC_069571.1**  **-----------------------------AAAAAATATA----------------------GATTATTTC** 404

**Hoya monetteae MW719053.1**  **AAAAAAAA-ATA------TAATTAAAATAAAAAAATATA----------------------GATTATTTC** 484

**Hoya omlorii MW719060.1**  **AAAAAAAAAATA------GAATTCAAATAAGAAAATAAAAAAATATA--------------GATTATTTC** 491

**Hoya ovalifolia NC_069563.1**  **AAAAAAAT-ATA------TAATTCAAATAAAAAAATATA----------------------GATTATTTC** 485

**Hoya pandurata NC_069562.1**  **AAAAAAAATATA------TAATACAAATAAAAAAATATA----------------------GATTATTTC** 476

**Hoya pottsii OL754664.1**  **AAAAAAA--ATA--------ATTCAAATAAAAAAATATA----------------------GATTATTTC** 481

**Hoya pubicalyx NC_069561.1**  **AAAAAAA---TA------TAATTCAAATAAAAAAATATA----------------------GATTATTTC** 417

**Hoya radicalis NC_067961.1**  **ATTTCAA----------------TAAATAAAAAAATATA----------------------GATTATTTC** 460

**Hoya rigida NC_067962.1**  **AAAAAAAATATA------TAATTCAAATAAAAAAATATA----------------------GATTATTTC** 490

**Hoya silvatica NC_067963.1**  **AAAAAAA--ATA--------ATTCAAATAAAAAAATATA----------------------GATTATTTC** 481

**Hoya thomsonii NC_067612.1**  **AAAAAAAA--TA------TAATTCAAATAAAAAAATATA----------------------GATTATTTC** 468

**Hoya verticillata NC_085236.1**  **AAAAAAA--ATA------TAGATTATTT-------CTTA----------------------ATTTCTATA** 475

**Papuahoya urniflora MW719062.1** **AAAAAAAATAGAA--------TTCAAATAATAAAATATA----------------------GATTATTTC** 512

**Stephanotis volubilis OP133576** **GAAAAAAAAATC------GAATTCAAATAAGAAAATATA----------------------GATTATTTC** 464

**710 720 730 740 750 760 770**

**....|....|....|....|....|....|....|....|....|....|....|....|....|....|**

**Dischidia australis NC_067885.** **TTAATTTATATA----------------------------ATCTTAATTAATAT--------------TA** 525

**Gymnema yunnanense NC_079598.1** **TTAATTTCTATA--------------------------TTATTATAGCTATA----------------TT** 526

**Hoya ariadna NC_069568.1**  **TTAATTTCTATA--------------------------ATATTATAGCTATATTATTATA--------TT** 540

**Hoya commutata NC_067958.1**  **TTAATTTCTATA--------------------------ATATTATAGCTATA-------------TTATT** 518

**Hoya dimorpha NC_067959.1**  **TTAATTTCTATA--------------------------ATATTATAGCTATATTATTATA--------TT** 519

**Hoya exilis MW719054.1**  **TTAATTTATATA--------------------------ATATTATAATTATAGCTATATT-------ATT** 521

**Hoya griffithii NC_069565.1**  **TTAATTTCTATA-------------A-------------TATTATAGCTATATT-------------ATT** 494

**Hoya kerrii NC_069570.1**  **TCAATTTCTATA--------------------------ATATTATAGCTATA-------------TTATT** 523

**Hoya lacunosa NC_069564.1**  **TTAATTTCTATA--------------------------ATATTATAGCTATA-------------ATATT** 548

**Hoya lanceolata NC_067960.1**  **TTAATTTCTATA--------------------------ATATTATAGTTATATATTATAGCTATATTATT** 461

**Hoya liangii OL826865.1**  **TTAATTTCTATA-------------A-------------TATTATAGCTATATT-------------ATT** 494

**Hoya lithophytica MW719058.1**  **TTAATTTCTATA--------------------------ATATTATAGCTATATTATTATA--------TT** 630

**Hoya lockii OR475243.1**  **TTAATTTCTATA--------------------------ATATTATAGCTATAATATTATAG----AAATT** 562

**Hoya longifolia NC_069560.1**  **TTAATTTCTATA-------------A-------------TATTATAGCTATATT-------------ATT** 494

**Hoya lyi MW719055.1**  **TTAATTTCTATA--------------------------ATATTATAGCTATATATTATAGCTATAATATT** 552

**Hoya megalaster MW719063.1**  **TTAATTTCTATA--------------------------ATATTATAGCTATATTATTATA--------TT** 517

**Hoya meliflua NC_069571.1**  **TTAATTTCTATA-------------ATATTATAGCTATATATTATAGCTATATT-------------ATT** 448

**Hoya monetteae MW719053.1**  **TTAATTTATATA--------------------------ATATTATAGCTATATT-------------ATT** 515

**Hoya omlorii MW719060.1**  **TTAATTTCTATA--------------------------ATATTATAGCTATATTATTATA--------TT** 527

**Hoya ovalifolia NC_069563.1**  **TTAATTTCTATA--------------------------ATATTATAGCTATA-------------TTATT** 516

**Hoya pandurata NC_069562.1**  **TTAATTTCTATA--------------------------ATATTATAGCTATATT-------------ATT** 507

**Hoya pottsii OL754664.1**  **TTAATTTATATA--------------------------ATAAATTAGCTATA-------------TTATT** 512

**Hoya pubicalyx NC_069561.1**  **TTAATTTCTATA--------------------------ATATTATAGCTATATT-------------ATT** 448

**Hoya radicalis NC_067961.1**  **TTAATTTCTATA-------------ATATTATAGCTATATATTATAGCTATATT-------------ATT** 504

**Hoya rigida NC_067962.1**  **TTAATTTCTATA--------------------------ATATTATAGCTATATTTTT--------TTATT** 526

**Hoya silvatica NC_067963.1**  **TTAATTTATATA--------------------------ATAAATTAGCTATA-------------TTATT** 512

**Hoya thomsonii NC_067612.1**  **TTAATTTCTATACTTAATTTCTATAATATTATAGCTATATATTATAGCTATATT-------------ATT** 525

**Hoya verticillata NC_085236.1**  **ATATTTTCTATA--------------------------ATATTATAGCTATA-------------TTATT** 506

**Papuahoya urniflora MW719062.1** **TTAATTTCTAAA--------------------------ATATTATAGCTATATATTATAGCTATATTATT** 556

**Stephanotis volubilis OP133576** **TTAATTTCTATA--------------------------TTATTATAGCTATAGC--TATA--------TT** 498

**780 790 800 810 820 830 840**

**....|....|....|....|....|....|....|....|....|....|....|....|....|....|**

**Dischidia australis NC_067885.** **ATATTATAG-------------------------CTATATTATTATATT---------------------** 549

**Gymnema yunnanense NC_079598.1** **AT--------------------------------------------------------------------** 528

**Hoya ariadna NC_069568.1**  **ATA------------------------GA---TTCTTTATTATTTTTTTTCATTATTATT----------** 573

**Hoya commutata NC_067958.1**  **ATATTATAGATTATTTATTATTTTTTTT------CATTATTATTATATT-AATTATTATA------TTAA** 575

**Hoya dimorpha NC_067959.1**  **ATA------------------------GA---TTCTTTATTATTTTTTTTCATTATTATT----------** 552

**Hoya exilis MW719054.1**  **ATATTATAGATTCT-------------------TTAATATTTTTTTTT--CATTATTATT----------** 560

**Hoya griffithii NC_069565.1**  **ATATTATAGATTCTTTATTATTTTTTTT------CATTATTATTATATT-AATTATAT------------** 545

**Hoya kerrii NC_069570.1**  **ATATTATAGATTCTTTATTATTTTTTTTCATTATTATTATTATTATATT-AATTATATTT------ATAT** 586

**Hoya lacunosa NC_069564.1**  **ATATTATAGATTCTTTATTATTTTTTTT------CATTATTATTATATT-AATTAT------------AT** 599

**Hoya lanceolata NC_067960.1**  **ATATTATAGATTCTTTATTATTTTTTTT------CATTATTATTATTAT---------------------** 504

**Hoya liangii OL826865.1**  **ATATTATAGATTCTTTATTATTTTTTTT------CATTATTATTATATT-AATTATAT------------** 545

**Hoya lithophytica MW719058.1**  **ATA-----------------TATTCTAGA---TTCTTTATTATTTTTTTTCATTATTATT----------** 670

**Hoya lockii OR475243.1**  **ATATTATAGATTCTGATTC-------------TTTATTATTTTTTTTTTTCATTATTATT----------** 609

**Hoya longifolia NC_069560.1**  **ATATTATAGATTCTTTATTATTTTTTTT------CATTATTATTATATT-AATTATAT------------** 545

**Hoya lyi MW719055.1**  **ATATTATAGATTCTTTATTATTTTTTTT------CATTATTATTATATT-AATTAT------------AT** 603

**Hoya megalaster MW719063.1**  **ATA------------------------GA---TTCTTTATTATTTTTTT-CATTATTATT----------** 549

**Hoya meliflua NC_069571.1**  **ATATTATAGATTCTTTATTATTTTTTTTT-----CATTATTTTTATATT-AATTATAT------------** 500

**Hoya monetteae MW719053.1**  **ATATTATAGATTCTTTATTATTTTTTTT------CATTATTATTATATT-AATTATATATATTAATTATA** 578

**Hoya omlorii MW719060.1**  **ATTATATTATAATATTATTATATTATAGA---TTCTTTATTATTTTTTTTCATTATTATT----------** 584

**Hoya ovalifolia NC_069563.1**  **ATATTATAGATTCTTTATTATTTTTTTTT-----CATTATTATTATATT-AATTATATT-------ATAT** 573

**Hoya pandurata NC_069562.1**  **ATATTATAGATTCTTTATTATT------------CATTATTATTATATT-AATTATAT------------** 552

**Hoya pottsii OL754664.1**  **ATATTATAGATTATTTATTATTTTTTTTT-----CATTATTATTATATT-AATTAT--------------** 562

**Hoya pubicalyx NC_069561.1**  **ATATTATAGATTCTTTATTATTTTTTTT------CATTATTATTATATT-TATTATTTATAT-------A** 504

**Hoya radicalis NC_067961.1**  **ATATTATAGATTCTTTATTATTTTTTTT------CATTATTATTATATT-AATTATAT------------** 555

**Hoya rigida NC_067962.1**  **ATATTATAGATTCTTTATTAGTTTTTTT------CATTATAATTATATT-ATATATAT------------** 577

**Hoya silvatica NC_067963.1**  **ATATTATAGATTATTTATTATTTTTTTTT-----CATTATTATTATATT-AATTAT--------------** 562

**Hoya thomsonii NC_067612.1**  **ATATTATAGATTCTTTATTATTTTTTTT------CATTATTATTATATT-AATTATAT------------** 576

**Hoya verticillata NC_085236.1**  **ATATTATAGATTCTTTATTATTTTTTTT------CATTATTATTATATT-AATAATATT-------ATAT** 562

**Papuahoya urniflora MW719062.1** **ATATTATAGATTCCTTATTATTTTTTTT------CATTATTATT---AT---------------------** 596

**Stephanotis volubilis OP133576** **AT--------------------------------------------------------------------** 500

**850 860 870 880 890 900 910**

**....|....|....|....|....|....|....|....|....|....|....|....|....|....|**

**Dischidia australis NC_067885.** **-----------------ATAG-----ATTATTTATTATTATTTATTTAATATTATTTTTATTT-------** 590

**Gymnema yunnanense NC_079598.1** **------------------TAT-----ATTATAGATTATTTATTATT-ATTTTT-----TATT--------** 561

**Hoya ariadna NC_069568.1**  **------------ATATTATAT-----ATTATAGATTATTTATTATT-ATTTTTATTTATATTTTTTT---** 622

**Hoya commutata NC_067958.1**  **TTATAATTAATTATATTATAT-----ATTCTAGATTATTTATTATT-ATTTTTATTTATATTT-------** 632

**Hoya dimorpha NC_067959.1**  **------------ATAATATAT-----ATTCTATATTATTTATTATT-ATTCTTATTTAGATTTTTTT---** 601

**Hoya exilis MW719054.1**  **------------ATATTATAT-----ATTCTAGATTATTTATTATT-ATTTTTATTTATATTTTTTT---** 609

**Hoya griffithii NC_069565.1**  **----------------TATAT-----ATTCTAGATTATTTATTATTTATTATTATTTTTATTTCTAT---** 591

**Hoya kerrii NC_069570.1**  **TAATTATATTAGATATTCTTATTCTAGTTCTAGATTATTTATTATT-ATTTTTATTTCTATTT-------** 648

**Hoya lacunosa NC_069564.1**  **TTATATTAATTAGAATTATAT-----ATTCTAGATTATTTATTATT-ATTATT-------TTT-------** 649

**Hoya lanceolata NC_067960.1**  **--------------ATTATAT-----ATTCTAGATTATTTATTATT-ATTTTTATTTATATTT-------** 547

**Hoya liangii OL826865.1**  **----------------TATAT-----ATTCTAGATTATTTATTATTTATTATTATTTTTATTTCTAT---** 591

**Hoya lithophytica MW719058.1**  **------------ATATTATAT-----ATTATAGATTATTTATTATT-ATTTTTATTTATATTTTTTTATA** 722

**Hoya lockii OR475243.1**  **------------ATATTATAT-----ATTCTAGATTATTTATTATT-ATTTTTATTTATATTTTTTT---** 658

**Hoya longifolia NC_069560.1**  **----------------TATAT-----ATTCTAGATTATTTATTATTTATTATTATTTTTATTTCTAT---** 591

**Hoya lyi MW719055.1**  **TTATATATATTATA-TTATAT-----ATTCTAGATTATTTATTATT-ATTTTTATTTATATTT-------** 659

**Hoya megalaster MW719063.1**  **------------ATAATATAT-----ATTCTATATTATTTATTATT-ATTTTTATTTAGATTTTTTT---** 598

**Hoya meliflua NC_069571.1**  **----------------TATAT-----ATTCTAGATTATTTATTATT-ATTTTTATTTCTATTT-------** 541

**Hoya monetteae MW719053.1**  **TTTATATTAATTATATTATAT-----ATTCTAGATTATTTATTATTATTTTTTTTTTTATTTT-------** 636

**Hoya omlorii MW719060.1**  **------------ATATTATAT-----ATTATAGATTATTTATTATT-ATTTTTATTTATATATTTTT---** 633

**Hoya ovalifolia NC_069563.1**  **--------------------------ATTCTAGATTATTTATTATT-ATTTTTATTTCTATTT-------** 609

**Hoya pandurata NC_069562.1**  **----------------TATAT-----ATTCTAGATTATTTATTA------TTTATTATTATTT-------** 588

**Hoya pottsii OL754664.1**  **--------------ATTATAT-----ATTCTAGATTATTTATTTTT-TTTTTTATTAAAATTT-------** 605

**Hoya pubicalyx NC_069561.1**  **TATATATTA------TTATAT-----ATTCTAGATTATTTATTA---TTTTTTTTTTTTTTTT-------** 553

**Hoya radicalis NC_067961.1**  **----------------TATAT-----ATTCTAGATTATTTATTATTTATTATTATTTATATTT-------** 597

**Hoya rigida NC_067962.1**  **----------------TATAT-----ATTATAGATTATTTAT-----------------ATTT-------** 602

**Hoya silvatica NC_067963.1**  **--------------ATTATAT-----ATTCTAGATTATTTATTTTT-TTTTTTATTAAAATTT-------** 605

**Hoya thomsonii NC_067612.1**  **----------------TATAT-----ATTCTAGATTATTTATTATTTATTATTATTTTTATTTATAT---** 622

**Hoya verticillata NC_085236.1**  **--------------------------ATTCTAGATTATTTATTATT-ATTTTTATTTCTATTT-------** 598

**Papuahoya urniflora MW719062.1** **--------------ATTATGT-----ATTATAGATTATTTCTTATT-ATTTTTATTTATATTT-------** 639

**Stephanotis volubilis OP133576** **------------------TAT-----ATTATAGATTATTTATTATT-ATCTTT-----TATT--------** 533

**920 930 940 950 960 970 980**

**....|....|....|....|....|....|....|....|....|....|....|....|....|....|**

**Dischidia australis NC_067885.** **----TTTTATTAAAATTTGAATATTTATTAATAAATTT-------TTTATTATTAAA-------TACAAT** 642

**Gymnema yunnanense NC_079598.1** **-------------------AATATTAATAAA--AATTTTTT--------TTATTAAA-------TACAAT** 595

**Hoya ariadna NC_069568.1**  **-------TATTAAAATTTGAATATTAATTAATAAATTTTTTA-------TTATTAAA-------TACAAT** 671

**Hoya commutata NC_067958.1**  **---TTTTTTTTTATTAAATAC------------AATCT-------AATAATACAAAAA------TACAAT** 674

**Hoya dimorpha NC_067959.1**  **--------ATTAAAATTTGAATATTAATTAATAAATTTTTTA-------TTATTAAA-------TACAAT** 649

**Hoya exilis MW719054.1**  **--------ATTAAAATTTGAATATTAATTAATAAATTAA---------ATTAATAAA-------T-----** 650

**Hoya griffithii NC_069565.1**  **--TTTTTTATTAAAATTTGAATATTAATTAATAAATTT-------TTTATTATTAAA-------TACAAT** 645

**Hoya kerrii NC_069570.1**  **---TTTTTATTAAAATTTGAATATTAATTAATAAATTT-------TTTATTATTAAA-------TACAAT** 701

**Hoya lacunosa NC_069564.1**  **---TTTTT----------------------------TT-------TTTATTATTGAA-------------** 668

**Hoya lanceolata NC_067960.1**  **----TTTTATTAAAATTTGAATATTAATTAATAAATTT-------TTTATTATTAAA-------TACAAT** 599

**Hoya liangii OL826865.1**  **--TTTTTTATTAAAATTTGAATATTAATTAATAAATTT-------TTTATTATTAAA-------TACAAT** 645

**Hoya lithophytica MW719058.1**  **TTTTTTTTATTAAAATTTGAATATTAATTAATAAATTTTTTA-------TTATTAAA-------TACAAT** 778

**Hoya lockii OR475243.1**  **--------ATTAAAATTTGAATATTAATTAATAAATTTTTTATTTTTTATTATTAAA-------TACAAT** 713

**Hoya longifolia NC_069560.1**  **--TTTTTTATTAAAATTTGAATATTAATTAATAAATTT-------TTTATTATTAAA-------TACAAT** 645

**Hoya lyi MW719055.1**  **---TTTTTATTAAAATTTGAATATTAATTAATAAATTT-------TTTATTATTAAATACAATCTAATAA** 719

**Hoya megalaster MW719063.1**  **--------ATTAAAATTTGAATATTAATTAATAAATTTTTTA-------TTATTAAA-------TACAAT** 646

**Hoya meliflua NC_069571.1**  **---TTTTTATTAAAATTTGAATATTAATTAATAAATTT-------TTTATTATTAAA-------TACAAT** 594

**Hoya monetteae MW719053.1**  **----TTTTATTAAAATTTGAATATTAATTAATAAATTTTTTATTTTTTATTATTAAA-------TACAAT** 695

**Hoya omlorii MW719060.1**  **-----TTTATTAAAATTTGAATATTAATTAATAAATTTTTTA-------TTATTAAA-------TACAAT** 684

**Hoya ovalifolia NC_069563.1**  **---TTTTTATTAAAATTTGAATATTAATTAATAAATTT-------TTTATTATTAAA-------TACAAT** 662

**Hoya pandurata NC_069562.1**  **----TTTTATTAAAATTTGAATATTAATTAATAAATTT-------TTTATTATTAAA-------TACAAT** 640

**Hoya pottsii OL754664.1**  **---GAATATT--AATTAATA-------------AATTT-------ATTATTATTAAA-------TACAAT** 643

**Hoya pubicalyx NC_069561.1**  **----TTTTATTAAAATTTGAATATTAATTAATAAATTT-------TTTATTATTAAA-------TACAAT** 605

**Hoya radicalis NC_067961.1**  **---TTTT-ATTAAAATTTGAATATTAATTAATAAATTT-------TTTTTTATTAAA-------TACAAT** 649

**Hoya rigida NC_067962.1**  **----TTTTATTAAAATTTGACTATTAATTAATAAATTT-------TTTATTATTAAA-------TACAAT** 654

**Hoya silvatica NC_067963.1**  **---GAATATT--AATTAATA-------------AATTT-------ATTATTATTAAA-------TACAAT** 643

**Hoya thomsonii NC_067612.1**  **--TTTTTTATTAAAATTTGAATATTAATTAATAAATTT-------TTTATTATTAAA-------TACAAT** 676

**Hoya verticillata NC_085236.1**  **---TTTTTATTAAAATTTGAATATTAATTAATAAATTT-------TTTATTATTAAA-------TACAAT** 651

**Papuahoya urniflora MW719062.1** **----TTTTATTAAAATTTGAATATTAATTAATAAATTT-------ATTATTATTAAA-------TACAAT** 691

**Stephanotis volubilis OP133576** **-------------------TATATTTTTTATTTAATATTTT--------TTATTAAA-------AACAAT** 569

**990 1000 1010 1020 1030 1040 1050**

**....|....|....|....|....|....|....|....|....|....|....|....|....|....|**

**Dischidia australis NC_067885.** **CTAATAATA-----------------------CAATTTAAATTATATTTTATTTC-----GAATTTGAAA** 684

**Gymnema yunnanense NC_079598.1** **CTAATAATA-----------------------CAATTTCAATTCTATTTTATTTC-----GAATTTGAAA** 637

**Hoya ariadna NC_069568.1**  **CTAATAATA-----------------------CAATTTAAATTCTATTTTATTTC-----GAATTTGAAA** 713

**Hoya commutata NC_067958.1**  **CTAATAATA-----------------------CAATTTTAATTCTATTTTATTTC-----GAATTTGAAA** 716

**Hoya dimorpha NC_067959.1**  **CTAATAATA-----------------------CAATTTAAATTCTATTTTATTTC-----GAATTTGAAA** 691

**Hoya exilis MW719054.1**  **-TAATAATA-----------------------CAATTTAAATTATATTTTATTTC-----GAATTTGAAA** 691

**Hoya griffithii NC_069565.1**  **CTAATAATA-----------------------CAATTTAAATTCTATTTTATTTC-----GAATTTGAAA** 687

**Hoya kerrii NC_069570.1**  **CTAATAAAA-----------------------CAATTTAAATTCTATTTTATTTC-----GAATTTGAAA** 743

**Hoya lacunosa NC_069564.1**  **----------------------------------------------------------------------** 668

**Hoya lanceolata NC_067960.1**  **CTAATAATA-----------------------CAATTTTAATTCTATTTTATTT-----CGAATTTAAAA** 641

**Hoya liangii OL826865.1**  **CTAATAATA-----------------------CAATTTAAATTCTATTTTATTTC-----GAATTTGAAA** 687

**Hoya lithophytica MW719058.1**  **CTAATAATA-----------------------CAATTTAAATTCTATTTTATTTC-----GAATTTGAAA** 820

**Hoya lockii OR475243.1**  **CTAATAATA-----------------------CAATTTAAATTCTATTTTATTTC-----GAATTTGAAA** 755

**Hoya longifolia NC_069560.1**  **CTAATAATA-----------------------CAATTTAAATTCTATTTTATTTC-----GAATTTGAAA** 687

**Hoya lyi MW719055.1**  **TACAATTTT-----------------------AATTCTATTTTCTATTTTATTTC-----GAATTTGAAA** 761

**Hoya megalaster MW719063.1**  **CTAATAATA-----------------------CAATTTAAATTCTATTTTATTTC-----GAATTTGAAA** 688

**Hoya meliflua NC_069571.1**  **CTAATAAAA-----------------------CAATTTAAATTCTATTTTATTTC-----GAATTTGAAA** 636

**Hoya monetteae MW719053.1**  **CTAATAATACTAATAATACAATTTAAATAATACAATTTAAATTATATTTTATTTC-----GAATTTGAAA** 760

**Hoya omlorii MW719060.1**  **CTAATAATA-----------------------CAATTTAAATTCTATTTTATTTC-----GAATTTGAAA** 726

**Hoya ovalifolia NC_069563.1**  **CTAATAATA-----------------------CAATTTAAATTCTATTTTATTTC-----GAATTTGAAA** 704

**Hoya pandurata NC_069562.1**  **CTAATAATA-----------------------CAATTTAAATTCTATTTAATTTC-----GAATTTGAAA** 682

**Hoya pottsii OL754664.1**  **CTAATAATA-----------------------CAATTTTAATTCTATTTTATTTC-----GAATTTGAAA** 685

**Hoya pubicalyx NC_069561.1**  **CTAATAATA-----------------------CAATTTAAATTCTATTTTATTTC-----GAATTTGAAA** 647

**Hoya radicalis NC_067961.1**  **CTAATAATA-----------------------CAATTTAAATTCTATTTTATTTC-----GAATTTGAAA** 691

**Hoya rigida NC_067962.1**  **CTAATAATA-----------------------CAATTTAAATTCTATTTTTTTTTTT--AGAATTTGAAA** 699

**Hoya silvatica NC_067963.1**  **CTAATAATA-----------------------CAATTTTAATTCTATTTTATTTC-----GAATTTGAAA** 685

**Hoya thomsonii NC_067612.1**  **CTAATAATA-----------------------CAATTTAAATTATATTTTATTTC-----GAATTTGAAA** 718

**Hoya verticillata NC_085236.1**  **CTAATAATA-----------------------CAATTTAAATTCTATTTCATTTC-----GAATTTGAAA** 693

**Papuahoya urniflora MW719062.1** **CTAATAATA-----------------------CAATTTAAATTCTATTTTATTTTATTTCGAATTTGAAA** 738

**Stephanotis volubilis OP133576** **CTAATAATA-----------------------CAATTTAAATTCTATTTTATTTC-----AAATTTGAAA** 611

**1060 1070 1080 1090 1100 1110 1120**

**....|....|....|....|....|....|....|....|....|....|....|....|....|....|**

**Dischidia australis NC_067885.** **TGTTTTAA-------ATTGAATTGCTAATTTTTTTTT-ATTTATTG------AAATTAAATTCTTTTTCT** 740

**Gymnema yunnanense NC_079598.1** **TGTTTTAA-------ATTGAATTGCTAATTGATTTTG-ATTTATTG------AAATTCAATTCTTTTTCT** 693

**Hoya ariadna NC_069568.1**  **TGTTTTAA-------ATTGAATTGCTAATTTTTTTTT-ATTTATTG------AAATTCAATTCTTTTTCT** 769

**Hoya commutata NC_067958.1**  **TGTTTTAA-------ATTGAATTGCTAATTTTTTTTTTATTTATTG------AAATTCAATTCTTTTTCT** 773

**Hoya dimorpha NC_067959.1**  **TGTTTTAA-------ATTGAATTGCTAATTTTTTTTT-ATTTATTG------AAATTCAATTCTTTTTCT** 747

**Hoya exilis MW719054.1**  **TGTTTTAA-------ATTGAATTGCTAATTTTTTTTA--TTTATTG------AAATTCAATTCTTTTTCT** 746

**Hoya griffithii NC_069565.1**  **TGTTTTAA-------ATTGAATTGCTAATTTTTTTTTTTTT-ATTG------AAATTCAATTCTTTTTCT** 743

**Hoya kerrii NC_069570.1**  **TGTTTTAA-------ATTGAATTGCTAATTTTTTTT--ATTTATTG------AAATTCAATTCTTTTTCT** 798

**Hoya lacunosa NC_069564.1**  **---------------ATTAAATT-----CTTTTTCTTTATAGATT-------------------------** 693

**Hoya lanceolata NC_067960.1**  **TGTTTTAA-------ATTGAATTGCTAATTTTTTTTT-ATTTATTG------AAATTCAATTCTTTTTCT** 697

**Hoya liangii OL826865.1**  **TGTTTTAA-------ATTGAATTGCTAATTTTTTTTTTTTT-ATTG------AAATTCAATTCTTTTTCT** 743

**Hoya lithophytica MW719058.1**  **TGTTTTAA-------ATTGAATTGCTAATTTTTTTTTTATTTATTGTTATTAAAATTCAATTCTTTTTCT** 883

**Hoya lockii OR475243.1**  **TGTTTTAA-------ATTGAATTGCTAATTTTTTT-----TTATTG------AAATTTAATTCTTTTTCT** 807

**Hoya longifolia NC_069560.1**  **TGTTTTAA-------ATTGAATTGCTAATTTTTTTTTTTTTTATTG------AAATTCAATTCTTTTTCT** 744

**Hoya lyi MW719055.1**  **TGTTTTAA-------ATTGAATTGCTAATTTTTTTTTTATTTATTG------AAATTCAATTCTTTTTCT** 818

**Hoya megalaster MW719063.1**  **TGTTTTAA-------ATTGAATTGCTAATCTTTTTTT-ATTTATTG------AAATTCAATTCTTTTTCT** 744

**Hoya meliflua NC_069571.1**  **TGTTTTAA-------ATTGAATTGCTAATTTTTTTTATTT--ATTG------AAATTCAATTCTTTTTCT** 691

**Hoya monetteae MW719053.1**  **TGTTTTAA-------ATTGAATTGCTAATTTTTTTTTTTTTTATTG------AAATTCAATTCTTTTTCT** 817

**Hoya omlorii MW719060.1**  **TGTTTTAA-------ATTGAATTGCTAATTTTTTTTT-ATTTATTG------AAATTAAATTCTTTTTCT** 782

**Hoya ovalifolia NC_069563.1**  **TGTTTTAA-------ATTGAATTGCTAATTTTTTTT--ATTTATTG------AAATTCAATTCTTTTTCT** 759

**Hoya pandurata NC_069562.1**  **TGTTTTAA-------ATTGAATTGCTAATTTTTTTTTTTTT-ATTG------AAATTAAATTCTTTTTCT** 738

**Hoya pottsii OL754664.1**  **TGTTTTAA-------ATTGAATTGCTAATTTTTTTTT-ATTTATTG------AAATTCAATTCTTTTTCT** 741

**Hoya pubicalyx NC_069561.1**  **TGTTTTAA-------ATTGAATTGCTAATTTTTTTTTATTT-ATTG------AAATTCAATTCTTTTTCT** 703

**Hoya radicalis NC_067961.1**  **TGTTTTAA-------ATTGAATTGCTAATTTTTTTT---T--ATTG------AAATTCAATTCTTTTTCT** 743

**Hoya rigida NC_067962.1**  **TGTTTTAA-------ATTGAATTGCTCATTTTTTTTATTT--ATTG------AAATTCAATTCTTTTTCT** 754

**Hoya silvatica NC_067963.1**  **TGTTTTAA-------ATTGAATTGCTAATTTTTTTTT-ATTTATTG------AAATTCAATTCTTTTTCT** 741

**Hoya thomsonii NC_067612.1**  **TGTTTTAA-------ATTGAATTGCTAATTTTTTTT---T--ATTG------AAATTCAATTCTTTTTCT** 770

**Hoya verticillata NC_085236.1**  **TGTTTTAA-------ATTGAATTGCTAATTTTTTTTT-ATTTATTG------AAATTCAATTCTTTTTCT** 749

**Papuahoya urniflora MW719062.1** **TGTTTTAAGTTTTAAATTGAATTGCTAATTTCTTTTT-ATTTATTG------AAATTCAATTCTTTTTCT** 801

**Stephanotis volubilis OP133576** **TGTTTTAA-------ATTGAATTGCTAATTGATTTTG-ATTTATTG------AAATTCAATTCTTTTTCT** 667

**1130 1140 1150 1160 1170 1180 1190**

**....|....|....|....|....|....|....|....|....|....|....|....|....|....|**

**Dischidia australis NC_067885.** **TTATAGATTTCGACTTTTATGAATAGAATCGACAGGTCCTTTG--GTTTTTTTCTTAGTGATTTAGAAAA** 808

**Gymnema yunnanense NC_079598.1** **TTATAGATTTCTATTTTTATGAATAGAATCGATAGGTCCTTCGGTTTTTTTTTCTTAGTGATTTAGAATA** 763

**Hoya ariadna NC_069568.1**  **TTATAGATTTCGACTTTTATGAATAGAATCGACAGGTCCTTTG--GTTTTTTTCTTAGTGATTTAGAATA** 837

**Hoya commutata NC_067958.1**  **TTCTAGATTTCGACTTTTATGAATAGAATCGACAGGTCCTTTT--GTTTTTTTCTTAGTGATTTAGAATA** 841

**Hoya dimorpha NC_067959.1**  **TTATAGATTTCGACTTTTATGAATAGAATCGACAGGTCCTTTG-GTTTTTTT-CTTAGTGATTTAGAATA** 815

**Hoya exilis MW719054.1**  **TTATAGATTTCGACTTTTATGAATAGAATCGACAGGTCCTTTG-GTTTTTTT-CTTAGTGATTTAGAATA** 814

**Hoya griffithii NC_069565.1**  **TTATAGATTTCGACTTTTATGAATAGAATCGACAGGTCCTTTG--GTTTTTTTCTTAGTGATTTAGAAAA** 811

**Hoya kerrii NC_069570.1**  **TTATAGATTTCGACTTTTATGAATAGAATCGACAGGTCCTTTG--GTTTTTTTCTTAGTGATTTAGAATA** 866

**Hoya lacunosa NC_069564.1**  **---TCGATTTCGACTTTTATGAATAGAATCGACAGGTCCTTTT--GTTTTTTTCTTAGTGATTTAGAATA** 758

**Hoya lanceolata NC_067960.1**  **TTATAGATTTCGACTTTTATGAATAGAATCGACAGGTCCTTTG--GTTTTTTTCTTAGTGATTTAGAATA** 765

**Hoya liangii OL826865.1**  **TTATAGATTTCGACTTTTATGAATAGAATCGACAGGTCCTTTG--GTTTTTTTCTTAGTGATTTAGAAAA** 811

**Hoya lithophytica MW719058.1**  **TTATAGATTTCGACTTTTATGAATAGAATCGACAGGTCCTTTG--GTTTTTTTCTTAGTGATTTAGAATA** 951

**Hoya lockii OR475243.1**  **TTATAGATTTCGACTTTTATGAATAGAATCGACAGGTCCTTTG-GTTTTTTTTCTTAGTGATTTAGAATA** 876

**Hoya longifolia NC_069560.1**  **TTATAGATTTCGACTTTTATGAATAGAATCGACAGGTCCTTTG--GTTTTTTTCTTAGTGATTTAGAAAA** 812

**Hoya lyi MW719055.1**  **TTATAGATTTCGACTTTTATGAATAGAATCGACAGGTCCTTTG--GTTTTTTTCTTAGTGATTTAGAATA** 886

**Hoya megalaster MW719063.1**  **TTATAGATTTCGACTTTTATGAATAGAATCGACAGGTCCTTTG-GTTTTTTTTCTTAGTGATTTAGAATA** 813

**Hoya meliflua NC_069571.1**  **TTATAGATTTCGACTTTTATGAATAGAATCGACAGGTCCTTTG--GTTTTTTTCTTAGTGATTTAGAATA** 759

**Hoya monetteae MW719053.1**  **TTATAGATTTCGACTTTTATGAATAGAATCGACAGGTCCTTTT--GTTTTTTTCTTAGTGATTTAGAATA** 885

**Hoya omlorii MW719060.1**  **TTATAGATTTCGACTTTTATGAATAGAATCGACAGGTCCTTTG--GTTTTTTTCTTAGTGATTTAGAATA** 850

**Hoya ovalifolia NC_069563.1**  **TTATAGATTTCGACTTTTATGAATAGAATCGACAGGTCCTTTG--GTTTTTTTCTTAGTGATTTAGAATA** 827

**Hoya pandurata NC_069562.1**  **TTATAGATTTTGACTTTTATGAATAGAATCGACAGGTCCTTTG--GTTTTTTTCTTAGTGATTTAGAAAA** 806

**Hoya pottsii OL754664.1**  **TTATAGATTTCGACTTTTATGAATAGAATCGACAGGTCCTTTT--GTTTTTTTCTTAGTGATTTAGAATA** 809

**Hoya pubicalyx NC_069561.1**  **TTATAGATTTCGACTTTTATGAATAGAATCGACAGGTCCTTTG--GTTTTTTTCTTAGTGATTTAGAATA** 771

**Hoya radicalis NC_067961.1**  **TTATAGATTTCGACTTTTATGAATAGAATCGACAGGTCCTTTG--GTTTTTTTATTAGTGATTTAGAAAA** 811

**Hoya rigida NC_067962.1**  **TTATAGATTTCGACTTTTATGAATAGAATCGACAGGTCCTTTG--GTTTTTTTCTTAGTGATTTAGAATA** 822

**Hoya silvatica NC_067963.1**  **TTATAGATTTCGACTTTTATGAATAGAATCGACAGGTCCTTTT--GTTTTTTTCTTAGTGATTTAGAATA** 809

**Hoya thomsonii NC_067612.1**  **TTATAGATTTCGACTTTTATGAATAGAATCGACAGGTCCTTTG--GTTTTTTTCTTAGTGATTTAGAAAA** 838

**Hoya verticillata NC_085236.1**  **TTATAGATTTCGACTTTTATGAATAGAATCGACAGGTCCTTTG--GTTTTTTTCTTAGTGATTTAGAATA** 817

**Papuahoya urniflora MW719062.1** **TTATAGATTTCGACTTTTATGAATAGAATCGACAGGTCCTTTG--GTTTTTTTCTTAGTGATTTAGAATA** 869

**Stephanotis volubilis OP133576** **TTATAGATTTCTATTTTTATGAATAGAATCGATAGGTCCTTCG-TTTTTTTTTCTTAGTGATTTAGAATA** 736

**1200 1210 1220 1230 1240 1250 1260**

**....|....|....|....|....|....|....|....|....|....|....|....|....|....|**

**Dischidia australis NC_067885.** **AGAACAAGCAAGAGACTGGCTAGAAATTTCCATTTCAGAATTTAGAATATCTTGGTGTTGTATATTCTTT** 878

**Gymnema yunnanense NC_079598.1** **AGAACAAGCAAGAGACTGGCTAGAAATTTCCATTTCAGAATTTATAATATCTTGGTGTTGTATATTCTTT** 833

**Hoya ariadna NC_069568.1**  **AGAACAAGCAAGAGACTGGCTAGAAATTTCCATTTCAGAATTTAGAATATCTTGGTGTTGTATATTCTTT** 907

**Hoya commutata NC_067958.1**  **AGAACAAGCAAGAGACTGACTAGAAATTTCCATTTCAGAATTTAGAATATCTTGGTGTTGTATATTCTTT** 911

**Hoya dimorpha NC_067959.1**  **AGAACAAGCAAGAGACTGGCTAGAAATTTCCATTTCAGAATTTAGAATATCTTGGTGTTGTATATTCTTT** 885

**Hoya exilis MW719054.1**  **AGAACAAGCAAGAGACTGGCTAGAAATTTCCATTTCAGAATTTAGAATATCTTGGTGTTGTATATTCTTT** 884

**Hoya griffithii NC_069565.1**  **AGAACAAGCAAGAGACTGGCTAGAAATTTCCATTTCAGAATTTAGAATATCTTGGTGTTGTATATTCTTT** 881

**Hoya kerrii NC_069570.1**  **AGAACAAGCAAGAGACTGGCTAGAAATTTCCATTTCAGAATTTAGAATATCTTGGTGTTGTATATTCTTT** 936

**Hoya lacunosa NC_069564.1**  **AGAACAAGCAAGAGACTGACTAGAAATTTCCATTTCAGAATTTAGAATATCTTGGTCTTGTATATTCTTT** 828

**Hoya lanceolata NC_067960.1**  **AGAACAAGCAAGAGACTGGCTAGAAATTTCCATTTCAGAATTTAGAATATCTTGGTGTTGTATATTCTTT** 835

**Hoya liangii OL826865.1**  **AGAACAAGCAAGAGACTGGCTAGAAATTTCCATTTCAGAATTTAGAATATCTTGGTGTTGTATATTCTTT** 881

**Hoya lithophytica MW719058.1**  **AGAACAAGCAAGAGACTGGCTAGAAATTTCCATTTCAGAATTTAGAATATCTTGGTGTTGTATATTCTTT** 1021

**Hoya lockii OR475243.1**  **AGAACAAGCAAGAGACTGGCTAGAAATTTCCATTTCAGAATTTAGAATATCTTGGTGTTGTATATTCTTT** 946

**Hoya longifolia NC_069560.1**  **AGAACAAGCAAGAGACTGGCTAGAAATTTCCATTTCAGAATTTAGAATATCTTGGTGTTGTATATTCTTT** 882

**Hoya lyi MW719055.1**  **AGAACAAGCAAGAGACTGGCTAGAAATTTCCATTTCAGAATTTAGAATATCTTGGTGTTGTATATTCTTT** 956

**Hoya megalaster MW719063.1**  **AGAACAAGCAAGAGACTGGCTAGAAATTTCCATTTCAGAATTTAGAATATCTTGGTGTTGTATATTCTTT** 883

**Hoya meliflua NC_069571.1**  **AGAACAAGCAAGAGACTGGCTAGAAATTTCCATTTCAGAATTTAGAATATCTTGGTGTTGTATATTCTTT** 829

**Hoya monetteae MW719053.1**  **AGAACAAGCAAGAGACTGACTAGAAATTTCCATTTCAGAATTTAGAATATCTTGGTGTTGTATATTCTTT** 955

**Hoya omlorii MW719060.1**  **AGAACAAGCAAGAGACTGGCTAGAAATTTCCATTTCAGAATTTTGAATATCTTGGTGTTGTATATTCTTT** 920

**Hoya ovalifolia NC_069563.1**  **AGAACAAGCAAGAGACTGGCTAGAAATTTCCATTTCAGAATTTAGAATATCTTGGTGTTGTATATTCTTT** 897

**Hoya pandurata NC_069562.1**  **AGAACAAGCAAGAGACTGGCTAGAAATTTCCATTTCAGAATTTAGAATATCTTGGTGTTGTATATTCTTT** 876

**Hoya pottsii OL754664.1**  **AGAACAAGCAAGAGACTGACTAGAAATTTCCATTTCAGAATTTATAATATCTTGGTGTTGTATATTCTTT** 879

**Hoya pubicalyx NC_069561.1**  **AGAACAAGCAAGAGACTGGCTAGAAATTTCCATTTCAGAATTTAGAATATCTTGGTGTTGTATATTCTTT** 841

**Hoya radicalis NC_067961.1**  **AGAACAAGCAAGAGACTGGCTAGAAATTTCCATTTCAGAATTTAGAATATCTTGGTGTTGTATATTCTTT** 881

**Hoya rigida NC_067962.1**  **AGAACAAGCAAGAGACTGGCTAGAAATTTCCATTTCAGAATTTAGAATATCTTGGTGTTGTATATTCTTT** 892

**Hoya silvatica NC_067963.1**  **AGAACAAGCAAGAGACTGACTAGAAATTTCCATTTCAGAATTTATAATATCTTGGTGTTGTATATTCTTT** 879

**Hoya thomsonii NC_067612.1**  **AGAACAAGCAAGAGACTGGCTAGAAATTTCCATTTCAGAATTTAGAATATCTTGGTGTTGTATATTCTTT** 908

**Hoya verticillata NC_085236.1**  **AGAACAAGCAAGAGACTGGCTAGAAATTTCCATTTCAGAATTTAGAATATCTTGGTGTTGTATATTCTTT** 887

**Papuahoya urniflora MW719062.1** **AGAACAAGCAAGAGACTGGCTAGAAATTTCCATTTCAGAATTTAGAATATCTTGGTGTTGTATATTCTTT** 939

**Stephanotis volubilis OP133576** **AGAACAAGCAAGAGACTGGCTAGAAATTTCCATTTCAGAATTTAGAATATCTTGGTGTTGTATATTCTTT** 806

**1270 1280 1290 1300 1310 1320 1330**

**....|....|....|....|....|....|....|....|....|....|....|....|....|....|**

**Dischidia australis NC_067885.** **TTATTAGTATCCTAGCGGAGGACCCTCTCTTGATTGAATAG-------AAAAAAAGAAGACAGACTACCC** 941

**Gymnema yunnanense NC_079598.1** **TTATTAGTATCCTAGCGGAGGGCCTTCTCTTGATTGAATA-------GAAAAAAAGAAGACCGACTACCC** 896

**Hoya ariadna NC_069568.1**  **TTATTAGTATCCTAGCGGAGGACCTTCTCTTGATTGAATA-------GAAAAAAAGAAGACAGACTACCC** 970

**Hoya commutata NC_067958.1**  **TTATTAATATCCTAGCGGAGGACCTTCTCTTGATTGAATAT-------AAAAAAAGAAGACAGACTACCC** 974

**Hoya dimorpha NC_067959.1**  **TTATTAGTATCCTAGCGGAGGACCTTCTCTTGATTGAATA----------------AAGACAGACTACCC** 939

**Hoya exilis MW719054.1**  **TTATTAGTATCCTAGCGGAGGACCTTCTCTTGATTGAATATGAATAGAAAAAAAAGAAGACAGACTACCC** 954

**Hoya griffithii NC_069565.1**  **TTATTAGTATCCTAGCGGAGGACCTTCTCTTGATTGAATAT-------AAAAAAAGAAGACAGACTACCC** 944

**Hoya kerrii NC_069570.1**  **TTATTAATATCCTAGCGGAGGACCTTCTCTTGATTGAATAT-------AAAAAAAGAAGACAGACTACCC** 999

**Hoya lacunosa NC_069564.1**  **TTATTAATATCCTAGCGGAGGACCTTCTCTTGATTGAATAT-------AAAAAAAGAAGACAGACTACCC** 891

**Hoya lanceolata NC_067960.1**  **TTA--------------------------------------------------------ACAGACTACCC** 849

**Hoya liangii OL826865.1**  **TTATTAGTATCCTAGCGGAGGACCTTCTCTTGATTGAATAT-------AAAAAAAGAAGACAGACTACCC** 944

**Hoya lithophytica MW719058.1**  **TTATTAGTATCCTAGCGGAGGACCTTCTCTTGATTGAATA-------GAAAAAAAGAAGACAGACTACCC** 1084

**Hoya lockii OR475243.1**  **TTATTAGTATCCTAGCGGAGGACCTTCTCTTGATTGAATA-------GAAAAAAAGAAGACAGACTACCC** 1009

**Hoya longifolia NC_069560.1**  **TTATTAGTATCCTAGCGGAGGACCTTCTCTTGATTGAATAT-------AAAAAAAGAAGACAGACTACCC** 945

**Hoya lyi MW719055.1**  **TTATTAATATCCTAGCGGAGGACCTTCTCTTGATTGAATAT-------AAAAAAAGAAGACAGACTACCC** 1019

**Hoya megalaster MW719063.1**  **TTATTAGTATCCTAGCGGAGGACCTTCTCTTGATTGAATA-------GAAAAAAAGAAGACAGACTACCC** 946

**Hoya meliflua NC_069571.1**  **TTATTAATATCCTAGCGGAGGACCTTCTCTTGATTGAATAT-------AAAAAAAGAAGACAGACTACCC** 892

**Hoya monetteae MW719053.1**  **TTATTAATATCCTAGCGGAGGACCTTCTCTTGATTGAATAT-------AAAAAAAGAAGACAGACTACCC** 1018

**Hoya omlorii MW719060.1**  **TTATTAGTATCCTAGCGGAGGACCTTCTCTTGATTGAATA-------GAAAAAAAGAAGACAGACTACCC** 983

**Hoya ovalifolia NC_069563.1**  **TTATTAATATCCTAGCGGAGGACCTTCTCTTGATTGAATAT-------AAAAAAAGAAGACAGACTACCC** 960

**Hoya pandurata NC_069562.1**  **TTATTAGTATCCTAGCGGAGGACCTTCTCTTGATTGAATAT-------AAAAAAAGAAGACAGACTACCC** 939

**Hoya pottsii OL754664.1**  **TTATTAATATCCTAGCGGAGGACCTTCTCTTGATTGAATAT-------AAAAAAAGAAGACAGACTACCC** 942

**Hoya pubicalyx NC_069561.1**  **TTATTAATATCCTAGCGGAGGACCTTCTCTTGATTGAATAT-------AAAAAAAGAAGACAGACTACCC** 904

**Hoya radicalis NC_067961.1**  **TTATTAGTATCCTAGCGGAGGACCTTCTCTTGATTGAATAT-------AAAAAAAGAAGACAGACTACCC** 944

**Hoya rigida NC_067962.1**  **TTATTAGTATCCTAGCGGAGGACCTTCTCTTGATTGAATAT-------AAAAAAAGAAGACAGACTACCC** 955

**Hoya silvatica NC_067963.1**  **TTATTAATATCCTAGCGGAGGACCTTCTCTTGATTGAATAT-------AAAAAAAGAAGACAGACTACCC** 942

**Hoya thomsonii NC_067612.1**  **TTATTAGTATCCTAGCGGAGGACCTTCTCTTGATTGAATAT-------AAAAAAAGAAGACAGACTACCC** 971

**Hoya verticillata NC_085236.1**  **TTATTAATATCCTAGCGGAGGACCTTCTCTTGATTGAATAT-------AAAAAAAGAAGACAGACTACCC** 950

**Papuahoya urniflora MW719062.1** **TTATTAGTATCCTAGCGGAGGACCTTCTCTTGATTGAATAG-------AAAAAAAGAAGACAGACTACCC** 1002

**Stephanotis volubilis OP133576** **TTATGAGTATCCTAGCGGAGGGCCTTCTCTTGATTGAATA-------GAAAAAAAGAAGACAGACTACCC** 869

**1340 1350 1360 1370 1380 1390 1400**

**....|....|....|....|....|....|....|....|....|....|....|....|....|....|**

**Dischidia australis NC_067885.** **CTCGTGCTTCGCTAGGTCTATGTAAGGTATATGGCGAGCCTATTTTACA-------TTGTTAAT------** 998

**Gymnema yunnanense NC_079598.1** **CTCGTGCTTCGCTAGGTCTAGGTAAGGTATATGGCGAGCCTATTTTACA-------TTGGTAAT------** 953

**Hoya ariadna NC_069568.1**  **CTCGTGCTTCGCTAGGTCTAGGTAAGGTATATGGCGAGCCTATTTTACA-------TTGTTAAT------** 1027

**Hoya commutata NC_067958.1**  **CTCGTGCTTCGCTAGGTCTAGGTAAGGTATATGGCGAGCCTATTTTAC-------ATTGTTAAT------** 1031

**Hoya dimorpha NC_067959.1**  **CTCGTGCTTCGCTAGGTCTAGGTAAGGTATATGGCGAGCCTATTTTACA-------TTGTTAAT------** 996

**Hoya exilis MW719054.1**  **CTCGTGCTTCGCTAGGTCTAGGTAAGGTATATGGCGAGCCTATTTTACA-------TTGTTAAT------** 1011

**Hoya griffithii NC_069565.1**  **CTCGTGCTTCGCTAGGTCTAGGTAAGGTATATGGCGAGCCTATTTTACA-------TTGTTAAT------** 1001

**Hoya kerrii NC_069570.1**  **CTCGTGCTTCGCTAGGTCTAGGTAAGGTATATGGCGAGCCTATTTTACATTTTACATTGTTAAT------** 1063

**Hoya lacunosa NC_069564.1**  **CTCGTGCTTCGCTAGGTCTAGGTAAGGTATATGGCGAGCCTATTTTAC--TTTACATTGTTAAT------** 953

**Hoya lanceolata NC_067960.1**  **CTCGTGCTTCGCTAGGTCTAGGTAAGGTATATGGCGAGCCTATTTTACA-------TTGTTAAT------** 906

**Hoya liangii OL826865.1**  **CTCGTGCTTCGCTAGGTCTAGGTAAGGTATATGGCGAGCCTATTTTACA-------TTGTTAAT------** 1001

**Hoya lithophytica MW719058.1**  **CTCGTGCTTCGCTAGATCTAGGTAAGGTATATGGCGAGCCTATTTTACA-------TTGTTAAT------** 1141

**Hoya lockii OR475243.1**  **CTCGTGCTTCGCTAGGTCTAGGTAAGGTATATGGCGAGCCTATTTTACA-------TTGTTAATTGTTAA** 1072

**Hoya longifolia NC_069560.1**  **CTCGTGCTTCGCTAGGTCTAGGTAAGGTATATGGCGAGCCTATTTTACA-------TTGTTAAT------** 1002

**Hoya lyi MW719055.1**  **CTCGTGCTTCGCTAGGTCTAGGTAAGGTATATGGCGAGCCTATTTTACATTTTACATTATTAAT------** 1083

**Hoya megalaster MW719063.1**  **CTCGTGCTTCGCTAGGTCTAGGTAAGGTATATGGCGAGCCTATTTTACA-------TTGTTAAT------** 1003

**Hoya meliflua NC_069571.1**  **CTCGTGCTTCGCTAGGTCTAGGTAAGGTATATGGCGAGCCTATTTTACATTTTACATTGTTAAT------** 956

**Hoya monetteae MW719053.1**  **CTCGTGCTTCGCTAGGTCTAGGTAAGGTATATGGCGAGCCTATTTTACA-------TTGTTAAT------** 1075

**Hoya omlorii MW719060.1**  **CTCGTGCTTCGCTAGGTCTAGGTAAGGTATATGGCGAGCCTATTTTACA-------TTGTTAAT------** 1040

**Hoya ovalifolia NC_069563.1**  **CTCGTGCTTCGCTAGGTCTAGGTAAGGTATATGGCGAGCCTATTTTACATTTTACATTGTTAAT------** 1024

**Hoya pandurata NC_069562.1**  **CTCGTGCTTCGCTAGGTCTAGGTAAGGTATATGGCGAGCCTATTTTACA-------TTGTTAAT------** 996

**Hoya pottsii OL754664.1**  **CTCGTGCTTCGCTAGGTCTAGGTAAGGTATATGGCGAGCCTATTTTAC-------ATTGTTAAT------** 999

**Hoya pubicalyx NC_069561.1**  **CTCGTGCTTCGCTAGGTCTAGGTAAGGTATATGGCGAGCCTATTTTACA-------TTGTTAAT------** 961

**Hoya radicalis NC_067961.1**  **CTCGTGCTTCGCTAGGTCTAGGTAAGGTATATGGCGAGCCTATTTTACA-------TTGTTAAT------** 1001

**Hoya rigida NC_067962.1**  **CTCGTGCTTCGCTAGGTCTAGGTAAGGTATATGGCGAGCCTATTTTACA-------TTGTTAAT------** 1012

**Hoya silvatica NC_067963.1**  **CTCGTGCTTCGCTAGGTCTAGGTAAGGTATATGGCGAGCCTATTTTAC-------ATTGTTAAT------** 999

**Hoya thomsonii NC_067612.1**  **CTCGTGCTTCGCTAGGTCTAGGTAAGGTATATGGCGAGCCTATTTTACA-------TTGTTAAT------** 1028

**Hoya verticillata NC_085236.1**  **CTCGTGCTTCGCTAGGTCTAGGTAAGGTATATGGCGAGCCTATTTTACATTTTACATTGTTAAT------** 1014

**Papuahoya urniflora MW719062.1** **CTCGTGCTTCGCTAGGTCTAGGTAAGGTATATGGCGAGCCTATTTTACA-------TTGTTAAT------** 1059

**Stephanotis volubilis OP133576** **CTCGTGCTTCGCTAGGTCTAGGTAAGGTATATGGCGAGCCTATTTTACA-------TTGGTAAT------** 926

**1410 1420 1430 1440 1450 1460 1470**

**....|....|....|....|....|....|....|....|....|....|....|....|....|....|**

**Dischidia australis NC_067885.** **-AATGAGACTTACCAAAGATATTACATTTTTTAACTTGTACACAAGCACGACAGGTCCTTTCTAGATCCA** 1067

**Gymnema yunnanense NC_079598.1** **-AATGAGACTTACCAAAGATATTACATTTTTTAACTTGTACACAAGCACGACAGGTCCTTTCTAGGTCCA** 1022

**Hoya ariadna NC_069568.1**  **-AATGAGACTTACCAAAGATATTACATTTTTTAACTTGTACACAAGCACGACAGGTCCTTTCTAGATCCA** 1096

**Hoya commutata NC_067958.1**  **-AATGAGACTTACCAAAGATATTACATTTTTTAACTTGTACACAAGCACGACAGGTCTTTTCTAGATCCA** 1100

**Hoya dimorpha NC_067959.1**  **-AATGAGACTTACCAAAGATATTACATTTTTTAACTTGTACACAAGCACGACAGGTCCTTTCTAGATCCA** 1065

**Hoya exilis MW719054.1**  **-AATGAGACTTACCAAAGATATTACATTTTTTAACTTGTACACAAGCACGACAGGTCCTTTCTAGATCCA** 1080

**Hoya griffithii NC_069565.1**  **-AATGAGACTTACCAAAGATATTACATTTTTTAACTTGTACACAAGCACGACAGGTCTTTTCTAGATCCA** 1070

**Hoya kerrii NC_069570.1**  **-AATGAGACTCACCAAAGATATTACATTTTTTAACTTGTACACAAGCACGACAGGTCTTTTCTAGATCCA** 1132

**Hoya lacunosa NC_069564.1**  **-AATGAGACTTACCAAAGATATTACATTTTTTAACTTGTACACAAGCACGACAGGTCTTTTCTAGATCCA** 1022

**Hoya lanceolata NC_067960.1**  **-AATGAGACTTACCAAAGATATTACATTTTTTAACTTGTACACAAGCACGACAGGTCCTTTCTAGATCCA** 975

**Hoya liangii OL826865.1**  **-AATGAGACTTACCAAAGATATTACATTTTTTAACTTGTACACAAGCACGACAGGTCTTTTCTAGATCCA** 1070

**Hoya lithophytica MW719058.1**  **-AATGAGACTTACCAAAGATATTACATTTTTTAACTTGTACACAAGCACGACAGGTCCTTTCTAGATCCA** 1210

**Hoya lockii OR475243.1**  **TAATGAGACTTACCAAAGATATTACATTTTTTAACTTGTACACAAGCACGACAGGTCCTTTCTAGATCCA** 1142

**Hoya longifolia NC_069560.1**  **-AATGAGACTTACCAAAGATATTACATTTTTTAACTTGTACACAAGCACGACAGGTCTTTTCTAGATCCA** 1071

**Hoya lyi MW719055.1**  **-AATGAGACTTACCAAAGATATTACATTTTTTAACTTGTACACAAGCACGACAGGTCTTTTCTAGATCCA** 1152

**Hoya megalaster MW719063.1**  **-AATGAGACTTACCAAAGATATTACATTTTTTAACTTGTACACAAGCACGACAGGTCCTTTCTAGATCCA** 1072

**Hoya meliflua NC_069571.1**  **-AATGAGACTCACCAAAGATATTACATTTTTTAACTTGTACACAAGCACGACAGGTCTTTTCTAGATCCA** 1025

**Hoya monetteae MW719053.1**  **-AATGAGACTTACCAAAGATATTACATTTTTTAACTTGTACACAAGCACGACAGGTCTTTTCTAGATCCA** 1144

**Hoya omlorii MW719060.1**  **-AATGAGACTTACCAAAGATATTACATTTTTTAACTTGTACACAAGCACGACAGGTCCTTTCTAGATCCA** 1109

**Hoya ovalifolia NC_069563.1**  **-AATGAGACTTACCAAAGATATTACATTTTTTAACTTGTACACAAGCACGACAGGTCTTTTCTAGATCCA** 1093

**Hoya pandurata NC_069562.1**  **-AATGAGACTTACCAAAGATATTACATTTTTTAACTTGTACACAAGCACGACAGGTCTTTTCTAGATCCA** 1065

**Hoya pottsii OL754664.1**  **-AATGAGACTTACCAAAGATATTACATTTTTTAACTTGTACACAAGCACGACAGGTCTTTTCTAGATCCA** 1068

**Hoya pubicalyx NC_069561.1**  **-AATGAGACTTACCAAAGATATTACATTTTTTAACTTGTACACAAGCACGACAGGTCTTTTCTAGATCCA** 1030

**Hoya radicalis NC_067961.1**  **-AATGAGACTTACCAAAGATATTACATTTTTTAACTTGTACACAAGCACGACAGGTCTTTTCTAGATCCA** 1070

**Hoya rigida NC_067962.1**  **-AATGAGACTTACCAAAGATATTACATTTTTGAACTTGTACACAAGCACGACAGGTCTTTTCTAGATCCA** 1081

**Hoya silvatica NC_067963.1**  **-AATGAGACTTACCAAAGATATTACATTTTTTAACTTGTACACAAGCACGACAGGTCTTTTCTAGATCCA** 1068

**Hoya thomsonii NC_067612.1**  **-AATGAGACTTACAAAAGATATTACATTTTTTAACTTGTACACAAGCACGACAGGTCTTTTCTAGATCCA** 1097

**Hoya verticillata NC_085236.1**  **-AATGAGACTTACCAAAGATATTACATTTTTTAACTTGTACACAAGCACGACAGGTCTTTTCTAGATCCA** 1083

**Papuahoya urniflora MW719062.1** **-AATGAGACTTACCAAAGATATTACATTTTTTAACTTGTACACAAGCACGACAGGTCCTTTCTAGATCCA** 1128

**Stephanotis volubilis OP133576** **-AATGAGACTTCCCAAAGATATTACATTTTTTAACTTGTACACAAGCACGACAGGTCCTTTCTAGATCCA** 995

**1480 1490 1500 1510 1520 1530 1540**

**....|....|....|....|....|....|....|....|....|....|....|....|....|....|**

**Dischidia australis NC_067885.** **TTGGGGTTTTTAACAGGGTGACTCGTGTCATGATTTTAACTTCAAAAATTCACCAACATTGGCTATACCA** 1137

**Gymnema yunnanense NC_079598.1** **TTGGGGTTTTTAACAGGGTGACTCGTGTCATGATTTTGACTTCAAAAATTCACCAACATTGGCTATACCA** 1092

**Hoya ariadna NC_069568.1**  **TTGGGGTTTTTAAGAGGGTGACTCGTGTCATGATTTTGACTTCAAAAATTCACCAACATTGGCTATACCA** 1166

**Hoya commutata NC_067958.1**  **TTGGGGTTTTTAACAGGGTGACTTGTGTCATGATTTTTACTTCAAAAATTCACCAACATTGGCTATACCA** 1170

**Hoya dimorpha NC_067959.1**  **TTGGGGTTTTTAACAGGGTGACTCGTGTCATGATTTTGACTTCAAAAATTCACCAACATTGGCTATACCA** 1135

**Hoya exilis MW719054.1**  **TTGGGGTTTTTAACAGGGTGACTCGTGTCATGATTTTGACTTCAAAAATTCACCAACATTGGCTATACCA** 1150

**Hoya griffithii NC_069565.1**  **TTGGGGTTTTTAACAGGGTGACTTGTGTCATGATTTTGACTTCAAAAATTCACCAACATTGGCTATACCA** 1140

**Hoya kerrii NC_069570.1**  **TTGGGGTTTTTAACAGGGTGACTTGTGTCATGATTTTGACTTCAAAAATTCACCAACATTGGCTATGCCA** 1202

**Hoya lacunosa NC_069564.1**  **TTGGTGTTTTTAACAGGGTGACTTGTGTCATGATTTTGACTTCAAAAATTCACCAACATTGGCTATACCA** 1092

**Hoya lanceolata NC_067960.1**  **TTGGTGTTTTTAACAGGGTGACTCGTGTCATGATTTTGACTTCAAAAATTCACCAACATTGGCTATACCA** 1045

**Hoya liangii OL826865.1**  **TTGGGGTTTTTAACAGGGTGACTTGTGTCATGATTTTGACTTCAAAAATTCACCAACATTGGCTATACCA** 1140

**Hoya lithophytica MW719058.1**  **TTGGGGTTTTTAACAGGGTGACTCGTGTCATGATTTTGACTTCAAAAATTCACCAACATTGGCTATACCA** 1280

**Hoya lockii OR475243.1**  **TTGGGGTTTTTAACAGGGTGACTCGTGTCATGATTTTGACTTCAAAAATTCACCAACATTGGCTATACCA** 1212

**Hoya longifolia NC_069560.1**  **TTGGGGTTTTTAACAGGGTGACTTGTGTCATGATTTTGACTTCAAAAATTCACCAACATTGGCTATACCA** 1141

**Hoya lyi MW719055.1**  **TTGGGGTTTTTAACAGGGTGACTTGTGTCATGATTTTGACTTCAAAAATTAACCAACATTGGCTATACCA** 1222

**Hoya megalaster MW719063.1**  **TTGGGGTTTTTAACAGGGTGACTCGTGTCATGATTTTGACTTCAAAAATTCACCAACATTGGCTATACCA** 1142

**Hoya meliflua NC_069571.1**  **TTGGGGTTTTTAACAGGGTGACTTGTGTCATGATTTTGCCTTCAAAAATTCACCAACATTGGCTATACCA** 1095

**Hoya monetteae MW719053.1**  **TTGGGGTTTTTAACAGGGTGACTTGTGTCATGATTTTGACTTCAAAAATTCACCAACATTGGCTATACCA** 1214

**Hoya omlorii MW719060.1**  **TTGGGGTTTTTAACAGGGTGACTCGTGTCATGATTTTGACTTCAAAAATTCACCAACATTGGCTATACCA** 1179

**Hoya ovalifolia NC_069563.1**  **TTGGGGTTTTTAACAGGGTGACTTGTGTCATGATTTTGACTTCAAAAATTCACCAACATTGGCTATACCA** 1163

**Hoya pandurata NC_069562.1**  **TTGGGGTTTTTAACAGGGTGACTTGTGTCATGATTTTGACTTCAAAAATTCACCAACATTGGCTATACCA** 1135

**Hoya pottsii OL754664.1**  **TTGGGGTTTTTAACAGGGTGACTTGTGTCATGATTTTTACTTCAAAAATTCACCAACATTGGCTATACCA** 1138

**Hoya pubicalyx NC_069561.1**  **TTGGGGTTTTTAACAGGGTGACTTGTGTCATGATTTTGACTTCAAAAATTCACCAACATTGGCTATACCA** 1100

**Hoya radicalis NC_067961.1**  **TTGGGGTTTTTAACAGGGTGACTTGTGTCATGATTTTGACTTCAAAAATTCACCAACATTGGCTATACCA** 1140

**Hoya rigida NC_067962.1**  **TTGGGGTTTTTAACAGGGTGACTTGTGTAATGATTTTGACTTCAAAAATTCACCAACATTGGCTATACCA** 1151

**Hoya silvatica NC_067963.1**  **TTGGGGTTTTTAACAGGGTGACTTGTGTCATGATTTTTACTTCAAAAATTCACCAACATTGGCTATACCA** 1138

**Hoya thomsonii NC_067612.1**  **TTGGGGTTTTTAACAGGGTGACTTGTGTCATGATTTTGACTTCAAAAATTCACCAACATTGGCTATACCA** 1167

**Hoya verticillata NC_085236.1**  **T-------------------------------ATTTTGACTTCAAAAATTCACCAACATTGGCTATACCA** 1122

**Papuahoya urniflora MW719062.1** **TTGGGGTTTTTAACAGAGTGACTCGTGTCATGATTTTGACTTCAAAAATTCACCAACATTGGCTATACCA** 1198

**Stephanotis volubilis OP133576** **TTGGGGTTTTTAACAGGGTGACTCGTGTCATGATTTTGACTTCAAAAATTCACCAACATTGGATATACCA** 1065

**1550 1560 1570 1580 1590 1600 1610**

**....|....|....|....|....|....|....|....|....|....|....|....|....|....|**

**Dischidia australis NC_067885.** **AAGAAAGGGAGTCTCAAGAACTCCTTGAATATGAAACTCGCCTCTACC------CCCAAGATTAATGCCC** 1201

**Gymnema yunnanense NC_079598.1** **AAGAAAGGGAGTATCAAGAACTCCTTGAATATGAAACTCGCCCCTACC------CCCAAGATTAGTG---** 1153

**Hoya ariadna NC_069568.1**  **AAGAAAGGGAGTCTCAAGAACTCCTTGAATATGAAACTCGCCTCTACC------CCCAAGATTAATGCCC** 1230

**Hoya commutata NC_067958.1**  **AAGAAAGGGAGTCTCAAGAACTCGTTGAATATGAAATTCGCCTCTACC------CCCAAGATTAATGCCC** 1234

**Hoya dimorpha NC_067959.1**  **AAGAAAGGGAGTCTCAAGAACTCCTTGAATATGAAACTCGCCTCTACT------CCCAAGATTAATGCCC** 1199

**Hoya exilis MW719054.1**  **AAGAAAGGGAGTCTCAAGAACTCCTTGAATATGAAACTCGCCTCTACC------CCCAAGATTAAT----** 1210

**Hoya griffithii NC_069565.1**  **AAGAAAGGGAGTCTCAAGAACTCCTTGAATATGAAACTCGCCTCTACC------CCCAAGGTTAATGCCC** 1204

**Hoya kerrii NC_069570.1**  **AAGAAAGGGAGTATCAAGAACTCCTTGAATATGAAATTCGCCTCTACC------CCCAAGATTAATGCCC** 1266

**Hoya lacunosa NC_069564.1**  **AAGAAAGGGAGTCTCAAGAACTCCTTGAATATGAAATTCGCCTCTACC------CCCAAGATTAATGCCC** 1156

**Hoya lanceolata NC_067960.1**  **AAGAAAGGGAGTCTCAAGAACTCCTTGAATATGAAACTCGCCTCTACC------CCCAAGATTAATGCCC** 1109

**Hoya liangii OL826865.1**  **AAGAAAGGGAGTCTCAAGAACTCCTTGAATATGAAACTCGCCTCTACC------CCCAAGGTTAATGCCC** 1204

**Hoya lithophytica MW719058.1**  **AAGAAAGGGAGTCTCAAGAACTCCTTGAATATGAAACTCGCCTCTACC------CCCAAGATTAATGCCC** 1344

**Hoya lockii OR475243.1**  **AAGAAAGGAAGTCTCAAGAACTCCTTGAATATGAAACCCACCCCTACC------CCCAAGATTAATGCCC** 1276

**Hoya longifolia NC_069560.1**  **AAGAAAGGGAGTCTCAAGAACTCCTTGAATATGAAACTCGCCTCTACC------CCCAAGGTTAATGCCC** 1205

**Hoya lyi MW719055.1**  **AAGAAAGGGAGTCTCAAGAACTCCTTGAATATGAAATTCGCCTCTACC------CCCAAGATTAATGCCC** 1286

**Hoya megalaster MW719063.1**  **AAGAAAGGGAGTCTCAAGAACTCCTTGAATATGAAACTCGCCTCTACT------CCCAAGATTAATGCCC** 1206

**Hoya meliflua NC_069571.1**  **AAGAAAGGGAGTCTCAAGAACTCCTTGAATATGAAATTCGCCTCTACC------CCCAAGATTAATGCCC** 1159

**Hoya monetteae MW719053.1**  **AAGAAAGGGAGTCTCAAGAACTCCTTGAATATGAAATTCGCCTCTACC------CCCAAGATTAATGCCC** 1278

**Hoya omlorii MW719060.1**  **AAGAAAGGGAGTCTCAAGAACTCCTTGAATATGAAACTCGCCTCTACC------CCCAAGATTAATGCCC** 1243

**Hoya ovalifolia NC_069563.1**  **AAGAAAGGGAGTCTCAAGAACTCCTTGAATATGAAATTCGCCTCTACC------CCCAAGATTAATGCCC** 1227

**Hoya pandurata NC_069562.1**  **AAGAAAGGGAGTCTCAAGAACTCCTTGAATATGAAACTCGCCTCTACC------CCCAAGGTTAATGCCC** 1199

**Hoya pottsii OL754664.1**  **AAGAAAGGGA---------ACTCGTTGAATATGAAATTCGCCTCTACC------CCCAAGATTAATGCCC** 1193

**Hoya pubicalyx NC_069561.1**  **AAGAAAGGGAGTCTCAAGAACTCCTTGAATATGAAATTCGCCTCTACC------CCCAAGATTAATGCCC** 1164

**Hoya radicalis NC_067961.1**  **AAGAAAGGGAGTCTCAAGAACTCCTTGAATATGAAACTCGCCTCTACC------CCCAAGGTTAATGCCC** 1204

**Hoya rigida NC_067962.1**  **AAGAAAGGGAGTCTCAAGAACTCCTTGAATATGAAACTCGCCTCTACCTCTACCCCCAAGGTTAATGCCC** 1221

**Hoya silvatica NC_067963.1**  **AAGAAAGGGA---------ACTCGTTGAATATGAAATTCGCCTCTACC------CCCAAGATTAATGCCC** 1193

**Hoya thomsonii NC_067612.1**  **AAGAAAGGGAGTCTCAAGAACTCCTTGAATATGAAACTCGCCTCTACC------CCCAAGGTTAATGCCC** 1231

**Hoya verticillata NC_085236.1**  **AAGAAAGGGAGTCTCAAGAACTCCTTGAATATGAAATTCGCCTCTACC------CCCAAGATTAATGCCC** 1186

**Papuahoya urniflora MW719062.1** **AAGAAAGGTAGTCTCAAGAACTCCTTGAATATGAAACTCGCCTCTACC------CCCAAGATTAATGCCC** 1262

**Stephanotis volubilis OP133576** **AAGAAAGGGAATATCAAGAACTCCTTGAATATGAAACTCACCCCTACC------CCCAAGATTAATG---** 1126

**1620 1630 1640 1650 1660 1670 1680**

**....|....|....|....|....|....|....|....|....|....|....|....|....|....|**

**Dischidia australis NC_067885.** **CAAGATTAATAACAAAAGGTTGCTTTGTTTATCCGCGATTGGAAAACTATCAATTGAAACGCCTTTTTTT** 1271

**Gymnema yunnanense NC_079598.1** **-----------GCAAAAGGTTGCTTTGTTTATCCGCGATTGGAAAACTATCAATCGGATCCACTGAAACG** 1212

**Hoya ariadna NC_069568.1**  **CAAGATTAATAACAAAAGGTTGCTTTGTTTATCCTCGATTGGAAAACTATCAATTGGATCCACTGAAACG** 1300

**Hoya commutata NC_067958.1**  **CAAGATTAATAACAAAAGGTTTCTTTGTTTATCCGCGATTGGAAAACTATCAATTGGGTCCACTGAAACG** 1304

**Hoya dimorpha NC_067959.1**  **CAAGATTAATAACAAAAGGTTGCTTTGTTTATCCGCGATTGGAAAACTATCAATTGGATCCACTGAAACG** 1269

**Hoya exilis MW719054.1**  **----------AACAAAAGGTTGCTTTGTTTATCCGCGATTGGAAAACTATCAATTGGATCCACTGAAACG** 1270

**Hoya griffithii NC_069565.1**  **CAAGATTAATAACAAAAGGTTGCTTTGTTTATCCGCGATTGGAAAACTATCAATTGGATCCACCGAAACG** 1274

**Hoya kerrii NC_069570.1**  **CAAGATTAATAACAAAAGGTTTCTTTGTTTATCCGCGATTGGAAAACTATCAATTGGGTCCACTGAAACG** 1336

**Hoya lacunosa NC_069564.1**  **CAAGATTAATAACAAAAGGTTTCTTTGTTTATCCGCGATTGGAAAACTATCAATTGGGTCCACTGAAACG** 1226

**Hoya lanceolata NC_067960.1**  **CAAGATTAATAACAAAAGGTTGCTTTGTTTATCCGCGATTGGAAAACTATCAATTGGATCCACTGAAACG** 1179

**Hoya liangii OL826865.1**  **CAAGATTAATAACAAAAGGTTGCTTTGTTTATCCGCGATTGGAAAACTATCAATTGGATCCACCGAAACG** 1274

**Hoya lithophytica MW719058.1**  **CAAGATTAATAACAAAAGGTTGCTTTGTTTATCCGCGATTGAAAAACTATCAATTGGATCCACTGAAACG** 1414

**Hoya lockii OR475243.1**  **CAAGATTAATAACAAAAGGTTGCTTTGTTTATCCGCGATTGGAAAACTATCAATTGGATCCACTGAAACG** 1346

**Hoya longifolia NC_069560.1**  **CAAGATTAATAACAAAAGGTTGCTTTGTTTATCCGCGATTGGAAAACTATCAATTGGATCCACCGAAACG** 1275

**Hoya lyi MW719055.1**  **CAAGATTAATAACAAAAGGTTTCTTTGTTTATCCGCGATTGGAAAACTATCAATTGGGTCCACTGAAACG** 1356

**Hoya megalaster MW719063.1**  **CAAGATTAATAACAAAAGGTTGCTTTGTTTATCCGCGATTGGAAAACTATCAATTGGATCCACTGAAACG** 1276

**Hoya meliflua NC_069571.1**  **CAAGATTAATAACAAAAGGTTTCTTTGTTTATCCGCGATTGGAAAACTATCAATTGGGTCCACTGAAACG** 1229

**Hoya monetteae MW719053.1**  **CAAGATTAATAACAAAAGGTTTCTTTGTTTATCCGCGATTGGAAAACTATCAATTGGGTCCACTGAAACG** 1348

**Hoya omlorii MW719060.1**  **CAAGATTAATAACAAAAGGTTGCTTTGTTTATCCTCGATTGGAAAACTATCAATTGGATCCACTGAAACG** 1313

**Hoya ovalifolia NC_069563.1**  **CAAGATTAATAACAAAAGGTTTCTTTGTTTATCCGCGATTGGAAAACAATCAATTGGGTCCACTGAAACG** 1297

**Hoya pandurata NC_069562.1**  **CAAGATTAATAACAAAAGGTTGCTTTGTTTATCCGCGATTGGAAAACAATCAATTGGATCCACTGAAACG** 1269

**Hoya pottsii OL754664.1**  **CAAGATTAATAACAAAAGGTTTCTTTGTTTATCCGCGATTGGAAAACTATCAATTGGGTCCACTGAAACG** 1263

**Hoya pubicalyx NC_069561.1**  **CAAGATTAATAACAAAAGGTTTCTTTGTTTATCCGCGATTGGAAAAATATCAATTGGGTCCACTGAAACG** 1234

**Hoya radicalis NC_067961.1**  **CAAGATTAATAACAAAAGGTTGCTTTGTTTATCCGCGATTGGAAAACTATCAATTGGATCCACTGAAACG** 1274

**Hoya rigida NC_067962.1**  **CAAGATTAATAACAAAAGGTTGCTTTGTTTATCCACGATTGGAAAACTATCAATTGGATCCACTGAAACG** 1291

**Hoya silvatica NC_067963.1**  **CAAGATTAATAACAAAAGGTTTCTTTGTTTATCCGCGATTGGAAAACTATCAATTGGGTCCACTGAAACG** 1263

**Hoya thomsonii NC_067612.1**  **CAAGATTAATAACAAAAGGTTGCTTTGTTTATCCGCGATTGGAAAACAATCAATTGGATCCACTGAAACG** 1301

**Hoya verticillata NC_085236.1**  **CAAGATTAATAACAAAAGGTTTCTTTGTTTATCCGCGATTGGAAAACTATCAATTGGGTCCACTGAAACG** 1256

**Papuahoya urniflora MW719062.1** **CAAGATTAATAACAAAAGGTTGCTTTGTTTATCCGCGATTGGAAAACTATCAATTGGATCCACTGAAACG** 1332

**Stephanotis volubilis OP133576** **-----------ACAAAAGGTTGCTTTGTTTATCCGCGATTGGAAAACTATCAATTGGATCCACTGAAACG** 1185

**1690 1700 1710 1720 1730 1740 1750**

**....|....|....|....|....|....|....|....|....|....|....|....|....|....|**

**Dischidia australis NC_067885.** **TTTTTTTTTTTT--------ATTTCGGGTTTATGTTCTGTTTTAAAAGCTTGCCGTAAGTAAACTTATGG** 1333

**Gymnema yunnanense NC_079598.1** **CCTTTTTTTTT--------TATTTCGGGTTTATGTTCTGTTTTAAAAGCTTGCCGTGAGTAAACTTATGG** 1274

**Hoya ariadna NC_069568.1**  **CCTTTTTTTTTT-------TATTTCGGGTTTATGTTCTGTTTTAAAAGCTTGCCGTGAGTAAACTTATGG** 1363

**Hoya commutata NC_067958.1**  **CCTTTTTTTTTT--------ATTTCGGGTTTATGTTCTGTTTTAAAAGCTTGCCGTGAGTAAACTTATGG** 1366

**Hoya dimorpha NC_067959.1**  **CCTTTTTTTTTT--------ATTTCGGGTTTATGTTCTGTTTTAAAAGCTTGCCGTGAGTAAACTTATGG** 1331

**Hoya exilis MW719054.1**  **CCTTTTCTTTT---------ATTTCGGGTTTATGTTCTGTTTTAAAAGCTTGCCGTGAGTAAACTTATGG** 1331

**Hoya griffithii NC_069565.1**  **CCTTTTTTTTTTTT------ATTTCGGGTTTATGTTCTGTTTTAAAAGCTTGCCGTGAGTAAACTTATGG** 1338

**Hoya kerrii NC_069570.1**  **CCTTTTTTTTT---------ATTTCGGGTTTATGTTCTGTTTTAAAAGCTTGCCGTGAGTAAACTTATGG** 1397

**Hoya lacunosa NC_069564.1**  **CCTTTTTTTTT-------------CGGGTTTATGTTCTGTTTTAAAAGCTTGCCGTGAGTAAACTTATGG** 1283

**Hoya lanceolata NC_067960.1**  **CCTTTTTTTTTTTTTTTTTTATTTCGGGTTTATGTTCTGTTTTAAAAGCTTGCCGTGAGTAAACTTATGG** 1249

**Hoya liangii OL826865.1**  **CCTTTTTTTTTTT-------ATTTCGGGTTTATGTTCTGTTTTAAAAGCTTGCCGTGAGTAAACTTATGG** 1337

**Hoya lithophytica MW719058.1**  **CCTTTTTTTTT--------TATTTCGGGTTTATGTTCTGTTTTAAAAGCTTGCCGTGAGTAAACTTATGG** 1476

**Hoya lockii OR475243.1**  **CCTTTTTTT-------------TTCGGGTTTATGTTCTGTTTTAAAAGCTTGCCGTGAGTAAACTTATGG** 1403

**Hoya longifolia NC_069560.1**  **CCTTTTTTTTTTT-------ATTTCGGGTTTATGTTCTGTTTTAAAAGCTTGCCGTGAGTAAACTTATGG** 1338

**Hoya lyi MW719055.1**  **CCTTTTTTTTTTTTT-TATTATTTCGGGTTTATGTTCTGTTTTAAAAGCTTGCCGTGAGTAAACTTATGG** 1425

**Hoya megalaster MW719063.1**  **CCTTTTTTTTTTT------TATTTCGGGTTTATGTTCTGTTTTAAAAGCTTGCCGTGAGTAAACTTATGG** 1340

**Hoya meliflua NC_069571.1**  **CCTTTTTTTTTT--------ATTTCGGGTTTATGTTCT--------AGCTTGCCGTGAGTAAACTTATGG** 1283

**Hoya monetteae MW719053.1**  **CCTTTTTTTTTTT-------ATTTCGGGTTTATGTTCTGTTTTAAAAGCTTGCCGTGAGTAAACTTATGG** 1411

**Hoya omlorii MW719060.1**  **CCTTTTTTTTTT-------TATTTCGGGTTTATGTTCTGTTTTAAAAGCTTGCCGTGAGTAAACTTATGG** 1376

**Hoya ovalifolia NC_069563.1**  **CCTTTTTTTTTT--------ATTTCGGGTTTATGTTCTGTTTTAAAAGCTTGCCGTGAGTAAACTTATGG** 1359

**Hoya pandurata NC_069562.1**  **CCTTTTTTTT--T-------ATTTCGGGTTTATGTTCTGTTTTAAAAGCTTGCCGTGAGTAAACTTATGG** 1330

**Hoya pottsii OL754664.1**  **CCTTTTTTTTT---------ATTTCGGGTTTATGTTCTGTTTTAAAAGCTTGCCGTGAGTAAACTTATGG** 1324

**Hoya pubicalyx NC_069561.1**  **CCTTTTTTTTTTT-------ATTTCGGGTTTATGTTCTGTTTTAAAAGCTTGCCGTGAGTAAACTTCTGG** 1297

**Hoya radicalis NC_067961.1**  **CCTTTTTTTTTT--------ATTTCGGGTTTATGTTCTGTTTTAAAAGCTTGCCGTGAGTAAACTTATGG** 1336

**Hoya rigida NC_067962.1**  **CCTTTTTTTTTT---------TTTCGGGTTTATGTTCTGTTTTAAAAGCTTGCCGTGAGTAAACTTATGG** 1352

**Hoya silvatica NC_067963.1**  **CCTTTTTTTTT---------ATTTCGGGTTTATGTTCTGTTTTAAAAGCTTGCCGTGAGTAAACTTATGG** 1324

**Hoya thomsonii NC_067612.1**  **CCTTTTTTTTTT--------ATTTCGGGTTTATGTTCTGTTTTAAAAGCTTGCCGTGAGTAAACTTATGG** 1363

**Hoya verticillata NC_085236.1**  **CCTTTTTTTTTTT-------ATTTCGGGTTTATGTTCTGTTTTAAAAGCTTGCCGTGAGTAAACTTATGG** 1319

**Papuahoya urniflora MW719062.1** **CCTTTTTT--------TTTTATTTCGGGTTTATGTTCTGTTTTAAAAGCTTGCCGTGAGTAAACTTATGG** 1394

**Stephanotis volubilis OP133576** **CCTTTTTTT----------TATTTCGGGTTTATGTTCTGTTTTAAAAGCTTGCCGTGAGTAAACTTATGG** 1245

**1760 1770 1780 1790 1800 1810 1820**

**....|....|....|....|....|....|....|....|....|....|....|....|....|....|**

**Dischidia australis NC_067885.** **GAAAAATTTAGATTTCGATTAACCAACCCGACAGTTCCAAGCAACAAACAATAATGAAAAAA-TTATACA** 1402

**Gymnema yunnanense NC_079598.1** **GAAATTTTTAGATTTCGATTAACCAACCCGACAGTTCCAAGCAACAAACAATAACGAAAAAA-TTATACA** 1343

**Hoya ariadna NC_069568.1**  **GAAAAATTTAGATTTCGATTAACCAACCCGACAGTTCCAAGCAACAAACAATAATGAAAAAA-TTATACA** 1432

**Hoya commutata NC_067958.1**  **GAAAAAATTATATTTCGATTAACCAACCCGACAGTTCCAAGCAACAAACAATAATGAAAAAA-TTATACA** 1435

**Hoya dimorpha NC_067959.1**  **GAAAAATTTTGATTTCGATTAACCAACCCGACAGTTCCAAGCAACAAACAATAATGAAAAAA-TTATACA** 1400

**Hoya exilis MW719054.1**  **GAAAAATTTAGATTTCGATTAACCAACCCGACAGTTACAAGCAACAAACAAT------------------** 1383

**Hoya griffithii NC_069565.1**  **GAAAAATTTAGATTTCGATTAACCAACCCGACAGTTCCAAGCAACAAACAATAATGAAAAAA-TTATACA** 1407

**Hoya kerrii NC_069570.1**  **GAAAAATTTAGATTTCGATTAACCAACCCGACAGTTCCAAGCAACAAACAATAATGAAAAAT-TTATACA** 1466

**Hoya lacunosa NC_069564.1**  **GAAAAATTTAGATTTCGATTAACCAACCCGACAGTTCCAAGCAACAAACAATAATGAAAAAAATTATACA** 1353

**Hoya lanceolata NC_067960.1**  **GAAAAATTTAGATTTCGATTAACCAACCCGACAGTTCCAAGCAACAAACAATAATGAAAAAA-TTATACA** 1318

**Hoya liangii OL826865.1**  **GAAAAATTTAGATTTCGATTAACCAACCCGACAGTTCCAAGCAACAAACAATAATGAAAAAA-TTATACA** 1406

**Hoya lithophytica MW719058.1**  **GAAAAATTTAGATTTCGATTAACCAACCCGACAGTTCCAAGCAACAAACAATAATGAAAAAA-TTATACA** 1545

**Hoya lockii OR475243.1**  **GAAAAATTTATATTTCGATTAACCAACCCGACAGTTCCAAGCAACAAACAATAATGAAAAAA-TTATACA** 1472

**Hoya longifolia NC_069560.1**  **GAAAAATTTAGATTTCGATTAACCAACCCGACAGTTCCAAGCAACAAACAATAATGAAAAAA-TTATACA** 1407

**Hoya lyi MW719055.1**  **GAAAAATTTAGATTTCGATTAACCAACCCGACAGTTCCAAGCAACAAACAATAATGAAAAAA-TTATACA** 1494

**Hoya megalaster MW719063.1**  **GAAAAATTTAGATTTCGATTAACCAACCCGACAGTTCCAAGCAACAAACAATAATGAAAAAA-TTATACA** 1409

**Hoya meliflua NC_069571.1**  **GAAAAATTTAGATTTCGATTAACCAACCCGACAGTTCCAAGCAACAAACAATAATGAAAAAA-TTATACA** 1352

**Hoya monetteae MW719053.1**  **GAAAAATTTAGATTTCGATTAACCAACCCGACAGTTCCAAGCAACAAACAATAATGAAAAAA-TTATACA** 1480

**Hoya omlorii MW719060.1**  **GAAAAATTTAGATTTCGATTAACCAACCAGACAGTTCCAAGCAACAAACAATAATGAAAAAA-TTATACA** 1445

**Hoya ovalifolia NC_069563.1**  **GAAAAATTTAGATTTCGATTAACCAACCCGACAGTTCCAAGCAACAAACAATAATGAAAAAA-TTATACA** 1428

**Hoya pandurata NC_069562.1**  **TAAAAATTTAGATTTCGATTAACCAACCCGACAGTTCCAAGCAACAAACAATAATGAAAAAA-TTATACA** 1399

**Hoya pottsii OL754664.1**  **GAAAAATTTAGATTTCGATTAACCAACCCGACAGTTCCAAGCAACAAACAATAATGAAAAAA-TTATACA** 1393

**Hoya pubicalyx NC_069561.1**  **GAAAAATTTAGATTTCGATTAACCAACCCGACAGTTCCAAGCAACAAACAATAATGAAAAAA-TTATACA** 1366

**Hoya radicalis NC_067961.1**  **GAAAAATTTAGATTTCGATTAACCAACCCGACAGTTCCAAGCAACAAACAATAATGAAAAAA-TTATACA** 1405

**Hoya rigida NC_067962.1**  **GAAAAATTTAGATTTCGATTAACCAACCCGACAGTTCCAAGCAACAAACAATAATGAAAAAA-TTATACA** 1421

**Hoya silvatica NC_067963.1**  **GAAAAATTTAGATTTCGATTAACCAACCCGACAGTTCCAAGCAACAAACAATAATGAAAAAA-TTATACA** 1393

**Hoya thomsonii NC_067612.1**  **GAAAAATTTAGATTTCGATTAACCAACCCGACAGTTCCAAGCAACAAACAATAATGAAAAAA-TTATACA** 1432

**Hoya verticillata NC_085236.1**  **GAAAAATTTAGATTTCGATTAACCAACCCGACAGTTCCAAGCAACAAACAATAATGAAAAAA-TTATACA** 1388

**Papuahoya urniflora MW719062.1** **GAAAAATTTAGATTTCGATTAACCAACCCGACAGTTCCAAGCAACAAACAATAATGAAAAAA-TTATACA** 1463

**Stephanotis volubilis OP133576** **GAAAAATTTAGATTTCGATTAACCAACCCGACAGTTCCAAGCAACAAACAATAATGAAAAAA-TTATACA** 1314

**1830 1840 1850 1860 1870 1880 1890**

**....|....|....|....|....|....|....|....|....|....|....|....|....|....|**

**Dischidia australis NC_067885.** **ATTTCATTTTTTTTATT------TGAGTTTTCCTTAATCATTTTGTTTTATTATATATATATATATATAT** 1466

**Gymnema yunnanense NC_079598.1** **ATTGAATTTTTTGTATTTGTATTTGAATTTTCCTTAATCATTTTGTTTTATATTTTTTAT-TATAAT--T** 1410

**Hoya ariadna NC_069568.1**  **ATTTCATTTTTTGTATT------TTAGTTTTCCTTAATCATTTTGTTTTCTATTTTATAT-TATATAATT** 1495

**Hoya commutata NC_067958.1**  **ATTTCATTTTTTGTATT------TGAGTTTTCCTTAATCATTTTGTTTTATATTTTATAT-TATATAATT** 1498

**Hoya dimorpha NC_067959.1**  **ATTTCATTTTTTGTATT------TGAGTTTTCCTTAATCATTTTGTTTTATATTTTATAT-TATAAAATT** 1463

**Hoya exilis MW719054.1**  **---------TTTGTATT------TGAGTTTTCCTTAATCATTTTGTTTTATATTTTATAT-TATAAAATT** 1437

**Hoya griffithii NC_069565.1**  **ATTTCATTTTTTGTATT------TGAGTTTTCCTTAATCATTTTGTTTTATAT--------TATAAAATT** 1463

**Hoya kerrii NC_069570.1**  **ATTTCATTTTTTGTATT------TGAGTTTTCCTTAATCATTTTGTTTTATATTTTATAT-TATAAAATT** 1529

**Hoya lacunosa NC_069564.1**  **ATTTCATTTTTTGTATT------TGAGTTTTCCTTAATCATTTTGTTTTATATTTTATAT-TATATAATT** 1416

**Hoya lanceolata NC_067960.1**  **ATTTCATTTTTTGTATT------TGAGTTTTACTTA------TTGTTTTATATTTTATAT-TATA-----** 1370

**Hoya liangii OL826865.1**  **ATTTCATTTTTTGTATT------TGAGTTTTCCTTAATCATTTTGTTTTATAT--------TATAAAATT** 1462

**Hoya lithophytica MW719058.1**  **ATTTCCTTTTTTGTATT------TGAGTTTTCCTTAATCATTTTGTTTTATATTTTATAT-TATAATATT** 1608

**Hoya lockii OR475243.1**  **ATTTCGTTTTTTGTATT------TGAGTTTTCCTTAATCATTTTGTTTTATATTTTATAT-TATATA--T** 1533

**Hoya longifolia NC_069560.1**  **ATTTCATTTTTTGTATT------TGAGTTTTCCTTAATCATTTTGTTTTATAT--------TATAAAATT** 1463

**Hoya lyi MW719055.1**  **ATTTCATTTTTTGTATT------TGAGTTTTCCTTAATCATTTTGTTTTATATTTTATAT-TATAAAATT** 1557

**Hoya megalaster MW719063.1**  **ATTTCATTTTTTGTATT------TGAGTTTTCCTTAATCATTTTGTTTTATATTTTATAT-TATATAATT** 1472

**Hoya meliflua NC_069571.1**  **ATTTCATTTTTTGTATT------TGAGTTTTCCTTAATCATTTTGTTTTATATTTTATAT-TATAAAATT** 1415

**Hoya monetteae MW719053.1**  **ATTTCATTTTTTGTATT------TGAGTTTTCCTTAATCATTTTGTTTTATAT--------TTTATATTA** 1536

**Hoya omlorii MW719060.1**  **ATTTCATTTTTTGTATT------TGAGTTTTCCTTAATCATTTTGTTTTATATTTTATAT-TAAAAAATT** 1508

**Hoya ovalifolia NC_069563.1**  **ATTTCATTTTTTGTATT------TGAGTTTTCCTTAATCATTTTGTTTTATATTATAAA--------ATT** 1484

**Hoya pandurata NC_069562.1**  **ATTTCATTTTTTGTATT------TGAGTTTTCCTTA------TTGTTTTATAT--------TATAAAATT** 1449

**Hoya pottsii OL754664.1**  **ATTTCATTTTTTGTATT------TGAGTTTTCCTTAATCATTTTGTTTTATATTTTATAT-TATAAAATT** 1456

**Hoya pubicalyx NC_069561.1**  **ATTTCATTTTTTGTATT------TGAGTTTTCCTTAATCATTTTGTTTTATAT--------TATATAATT** 1422

**Hoya radicalis NC_067961.1**  **ATTTCATTTTTTGTTTT------TGAGTTTTCCTTAATCATTTTGTTTTATAT--------TATAAAATT** 1461

**Hoya rigida NC_067962.1**  **ATTTCATTTTTTGTATT------TGAGTTTTCCTTAATCATTTTGTTTTATATTTTATAT-TATAATATT** 1484

**Hoya silvatica NC_067963.1**  **ATTTCATTTTTTGTATT------TGAGTTTTCCTTAATCATTTTGTTTTATATTTTATAT-TATAAAATT** 1456

**Hoya thomsonii NC_067612.1**  **ATTTCATTTTTTGTATT------TGAGTTTTCCTTAATCATTA---ATTATAT-----------AAAATT** 1482

**Hoya verticillata NC_085236.1**  **ATTTCATTTTTTGTATT------TGAGTTTTCCTTAATCATTTTGTTTTATATTTTATAT-TAAAATATT** 1451

**Papuahoya urniflora MW719062.1** **ATTTCATTTTTTGTATT------TGAGTTTTCCTTAATCATTTTGTTTTATATTTTATAT-TATAGAATT** 1526

**Stephanotis volubilis OP133576** **ATTGAATTTTTTGTATT------TGAATTTTCCTTAATCATTTTGTTTTATATTTTATAT-TATAAT--T** 1375

**1900 1910 1920 1930 1940 1950 1960**

**....|....|....|....|....|....|....|....|....|....|....|....|....|....|**

**Dischidia australis NC_067885.** **TTATATATTATATAAATATATTAATATTAATAATTATACTAATAGTATAGTAATAGTA------------** 1524

**Gymnema yunnanense NC_079598.1** **ATA--------------GTAATA------GTA--------------------------------------** 1422

**Hoya ariadna NC_069568.1**  **ATA--------------GTAATA------GTAATAATT--ATAGTAATAGTAATAGTAT-----------** 1532

**Hoya commutata NC_067958.1**  **TTATATA-------ATTATAGTA------ATAGT-ATTT-TATATAATTATAGTAATAGTA---------** 1544

**Hoya dimorpha NC_067959.1**  **ATAGTA-ATAGT-AA--ATTATA------GTAATAGT---ATAGTAATAGTA------------------** 1502

**Hoya exilis MW719054.1**  **ATAGTAATAGTA-AATTATAGTA------ATAGTAGTA--ATAGTAATAGTA------------------** 1480

**Hoya griffithii NC_069565.1**  **TTATATA-------ATTATAGTA------ATAGTAATA--ATTATAGTAATAGTAT--------------** 1504

**Hoya kerrii NC_069570.1**  **TTATATA-------ATTATAGTA------ATAGTAATAG-TAAATAGTA---------------------** 1564

**Hoya lacunosa NC_069564.1**  **TTATATA-------ATTATAGTA------ATAGT-A----------------------------------** 1438

**Hoya lanceolata NC_067960.1**  **----TTTTTATA-TTATATAATTCTAGTAATAGTAATA--ATTATAGTAATAGTA---------------** 1418

**Hoya liangii OL826865.1**  **TTATATA-------ATTATAGTA------ATAGTAATA--ATTATAGTAATAGTA---------------** 1502

**Hoya lithophytica MW719058.1**  **ATA--------------GTAATA------GTA--------------------------------------** 1620

**Hoya lockii OR475243.1**  **ATA-------TA-AAATATAGTA------GTAATAGTA--A--GTAATAGTA------------------** 1567

**Hoya longifolia NC_069560.1**  **TTATATA-------ATTATAGTA------ATAGTAATA--ATTATAGTAATAGTA---------------** 1503

**Hoya lyi MW719055.1**  **TTATATA-------ATTATAGTA------ATAGT-A----------------------------------** 1579

**Hoya megalaster MW719063.1**  **ATATTATATAAT-TATAGTAATA------GTAA-ATT---ATAGTAATAGTA------------------** 1513

**Hoya meliflua NC_069571.1**  **TTATATA-------ATTATAGTA------ATAGTAATA--ATTATAATAATTATAGT---AATAGTA---** 1464

**Hoya monetteae MW719053.1**  **TAATATTTT--A-TATTATAAAATTTTATATAATTATATAATTATAGTAATACTAATAATTATAGTAATA** 1603

**Hoya omlorii MW719060.1**  **ATA--------------GTAATA------GTAATAATT--ATAGTAATAGTA------------------** 1538

**Hoya ovalifolia NC_069563.1**  **TTATATA-------ATTATAGTAT-----ATAATTATAG-TAA-TAGTAATAATTATAGTAATAGTA---** 1537

**Hoya pandurata NC_069562.1**  **TTATATAT---A-TATTTTTTATATAATTATAGTAATA--ATTATAGTAATAGTAAT---TATAGTAATA** 1510

**Hoya pottsii OL754664.1**  **ATATATA-------ATAATAGTA------ATA---ATTA-TA-GTAATAGTAGTAATAGTA---------** 1499

**Hoya pubicalyx NC_069561.1**  **TTATATA-------ATTATAAAA------ATAGTAATA--ATTATAGTAATAGTAAATAGTATATAAATA** 1477

**Hoya radicalis NC_067961.1**  **TTATATA-------ATTATAGTA------ATAGTAATA--ATTATAGTAATAGTAAT---TATAGTAATA** 1513

**Hoya rigida NC_067962.1**  **TTATATA-------ATTATAGTA------ATAGTAATA--ATTATAGTAATAGTAAT---TATAGTAATA** 1536

**Hoya silvatica NC_067963.1**  **ATATATA-------ATAATAGTA------ATA---ATTA-TA-GTAATAGTAGTAATAGTA---------** 1499

**Hoya thomsonii NC_067612.1**  **TTATATA-------ATTATAGTA------ATAGTAATT--ATAGTAATAGTA--AAT---TATAGTAATA** 1532

**Hoya verticillata NC_085236.1**  **TTATATA-------TATATAGTAT-----ATAATTATAG-TAA-TAGTAATAGTA---------------** 1492

**Papuahoya urniflora MW719062.1** **ATAATTATAGTA-ATAGATAATTATAGTAATAGTAATA--ATTATAGTAATAGTA---------------** 1578

**Stephanotis volubilis OP133576** **ATA--------------GTAATA------GTA--------------------------------------** 1387

**...**

**Dischidia australis NC_067885.** **---** 1524

**Gymnema yunnanense NC_079598.1** **---** 1422

**Hoya ariadna NC_069568.1**  **---** 1532

**Hoya commutata NC_067958.1**  **---** 1544

**Hoya dimorpha NC_067959.1**  **---** 1502

**Hoya exilis MW719054.1**  **---** 1480

**Hoya griffithii NC_069565.1**  **---** 1504

**Hoya kerrii NC_069570.1**  **---** 1564

**Hoya lacunosa NC_069564.1**  **---** 1438

**Hoya lanceolata NC_067960.1**  **---** 1418

**Hoya liangii OL826865.1**  **---** 1502

**Hoya lithophytica MW719058.1**  **---** 1620

**Hoya lockii OR475243.1**  **---** 1567

**Hoya longifolia NC_069560.1**  **---** 1503

**Hoya lyi MW719055.1**  **---** 1579

**Hoya megalaster MW719063.1**  **---** 1513

**Hoya meliflua NC_069571.1**  **---** 1464

**Hoya monetteae MW719053.1**  **GTA** 1606

**Hoya omlorii MW719060.1**  **---** 1538

**Hoya ovalifolia NC_069563.1**  **---** 1537

**Hoya pandurata NC_069562.1**  **GTA** 1513

**Hoya pottsii OL754664.1**  **---** 1499

**Hoya pubicalyx NC_069561.1**  **GTA** 1480

**Hoya radicalis NC_067961.1**  **GTA** 1516

**Hoya rigida NC_067962.1**  **GTA** 1539

**Hoya silvatica NC_067963.1**  **---** 1499

**Hoya thomsonii NC_067612.1**  **GTA** 1535

**Hoya verticillata NC_085236.1**  **---** 1492

**Papuahoya urniflora MW719062.1** **---** 1578

**Stephanotis volubilis OP133576** **---** 1387

## Figure S2. Alignment results of 30 *psbI-atpA* sequences from the studied species

**10 20 30 40 50 60 70**

**....|....|....|....|....|....|....|....|....|....|....|....|....|....|**

**Dischidia australis NC_067885.** **GGGTTTTTCTTTTCTATTTTCTTCGGATTTTTTGTTTA------TATAAAAACAAACGATTTGCCAAATT** 64

**Gymnema yunnanense NC_079598.1** **GGGTTTTTCTTTTCTATTTTCTTCGGATTTTTTGTTTA------TATAAAAACAAACGATTTGCCAAATT** 64

**Hoya ariadna NC_069568.1**  **GGGTTTTTCTTTTCTATTTTCTTCGGATTTTTTGTTTA------TATAAAAACAAACGATTTGCCAAATT** 64

**Hoya commutata NC_067958.1**  **GGGTTTTTCTTTTCTATTTTCTTCGGATTTTTTGTTTA------TATAAAAATGCCAAATTTTAAAAAAT** 64

**Hoya dimorpha NC_067959.1**  **GGGTTTTTCTTTTCTATTTTCTTCGGATTTTTTGTTTA------TATAAAAACAAACGATTTGCCAAATT** 64

**Hoya exilis MW719054.1**  **GGGTTTTTCTTTTCTATTTTCTTCGGATTTTTTGTTTA------TATAAAAACAAACGATTTGCCAAATT** 64

**Hoya griffithii NC_069565.1**  **GGGTTTTTCTTTTCTATTTTCTTCGGATTTTTTGTTTA------TATAAAAACAAACGATTTGCCAAATT** 64

**Hoya kerrii NC_069570.1**  **GGGTTTTTCTTTTCTATTTTCTTCGGATTTTTTGTTTA------TATAAAAATGCCAAATTTGAAAAAAA** 64

**Hoya lacunosa NC_069564.1**  **GGGTTTTTCTTTTCTATTTTCTTCGGATTTTTTGTTTA------TATAAAAATGCCAAATTTGAAAAA--** 62

**Hoya lanceolata NC_067960.1**  **GGGTTTTTCTTTTCTATTTTCTTCGGATTTTTTGTTTATATTTATATAAAAACAAACGATTTGCCAAATT** 70

**Hoya liangii OL826865.1**  **GGGTTTTTCTTTTCTATTTTCTTCGGATTTTTTGTTTA------TATAAAAACAAACGATTTGCCAAATT** 64

**Hoya lithophytica MW719058.1**  **GGGTTTTTCTTTTCTATTTTCTTCGGATTTTTTGTTTA------TATAAAAACAAACGATTTGCCAAATT** 64

**Hoya lockii OR475243.1**  **GGGTTTTTCTTTTCTATTTTCTTCGGATTTTTTGTTTA------TATAAAAACAAACAATTTGCCAAATT** 64

**Hoya longifolia NC_069560.1**  **GGGTTTTTCTTTTCTATTTTCTTCGGATTTTTTGTTTA------TATAAAAACAAACGATTTGCCAAATT** 64

**Hoya lyi MW719055.1**  **GGGTTTTTCTTTTCTATTTTCTTCGGATTTTTTGTTTA------TATAAAAATGCCAAATTTGAAAAAAA** 64

**Hoya megalaster MW719063.1**  **GGGTTTTTCTTTTCTATTTTCTTCGGATTTTTTGTTTA------TATAAAAACAAACGATTTGCCAAATT** 64

**Hoya meliflua NC_069571.1**  **GGGTTTTTCTTTTCTATTTTCTTCGGATTTTTTGTTTA------TATAAAAATGCCAAATTTGAAAAAAA** 64

**Hoya monetteae MW719053.1**  **GGGTTTTTCTTTTCTATTTTCTTCGGATTTTTTGTTTA------TATAAAAATGCCAAATTTGAAAAAAA** 64

**Hoya omlorii MW719060.1**  **GGGTTTTTCTTTTCTATTTTCTTCGGATTTTTTGTTTA------TATAAAAACAAACGATTTGCCAAATT** 64

**Hoya ovalifolia NC_069563.1**  **GGGTTTTTCTTTTCTATTTTCTTCGGATTTTTTGTTTA------TATAAAAATGCCAAATTTGAAAAAAA** 64

**Hoya pandurata NC_069562.1**  **GGGTTTTTCTTTTCTATTTTCTTCGGATTTTTTGTTTA------TATAAAAACA-----TTTGCCAAATT** 59

**Hoya pottsii OL754664.1**  **GGGTTTTTCTTTTCTATTTTCTTCGGATTTTTTGTTTA------TATAAAAATGCCAAATTTGAAAAAAA** 64

**Hoya pubicalyx NC_069561.1**  **GGGTTTTTCTTTTCTATTTTCTTCGGATTTTTTGTTTA------TATAAAAATGCCAAATTTGAAAAAAA** 64

**Hoya radicalis NC_067961.1**  **GGGTTTTTCTTTTCTATTTTCTTCGGATTTTTTGTTTA------TATAAAAACAAACGATTTGCCAAATT** 64

**Hoya rigida NC_067962.1**  **---------GTTTCTATTTTCTTCGGATTTTTTGTTTA------TATAAAAACAAAAGATTTGCCAAATT** 55

**Hoya silvatica NC_067963.1**  **GGGTTTTTCTTTTCTATTTTCTTCGGATTTTTTGTTTA------TATAAAAATGCCAAATTTGAAAAAAA** 64

**Hoya thomsonii NC_067612.1**  **GGGTTTTTCTTTTCTATTTTCTTCGGATTTTTTGTTTA------TATAAAAACAAACGATTTGCCAAATT** 64

**Hoya verticillata NC_085236.1**  **GGGTTTTTCTTTTCTATTTTCTTCGGATTTTTTGTTTA------TATAAAAATGCCAAATTTGAAAAAAA** 64

**Papuahoya urniflora MW719062.1** **GGGTTTTTCTTTTCTATTTTCTTCGGATTTTTTGTTTA------TATAAAAACAAACGATTTGCAAAATT** 64

**Stephanotis volubilis OP133576** **GGGTTTTTCTTTTCTATTTTCTTCGGATTTTTTGTTTA------TA--AAAACAAACGATTTGCCAAATT** 62

**80 90 100 110 120 130 140**

**....|....|....|....|....|....|....|....|....|....|....|....|....|....|**

**Dischidia australis NC_067885.** **TGAAAAAAAAAA----------TAAATAAAGTCATCAAGGGAACCGGAAAGAGAGGGATTCGAACCCTCG** 124

**Gymnema yunnanense NC_079598.1** **-------------AAAAAAAAAAAAATAAAGTCATCAAGGGAAGCGGAAAGAGAGGGATTCGAACCCTCG** 121

**Hoya ariadna NC_069568.1**  **TGAAAAAAAAAA-ATAAATAAATAAATAAAGTCATCAAGGGAAGCGGAAAGAGAGGGATTCGAACCCTCG** 133

**Hoya commutata NC_067958.1**  **AA-----------ATAAATAAATAAATAAAGTCATCAAGGGAAGCGGAAAGAGAGGGATTCGAACCCTCG** 123

**Hoya dimorpha NC_067959.1**  **TGAAAAAAAAA-AATTAAAAAATAAATAAAGTCATCAAGGGAAGCGGAAAGAGAGGGATTCGAACCCTCG** 133

**Hoya exilis MW719054.1**  **TGAAAAAA-----ATAAATAAATAAATAAAGTCATCAAGGGAAGCGGAAAGAGAGGGATTCGAACCCTCG** 129

**Hoya griffithii NC_069565.1**  **TGAAAAAAA-----TAAATAAATAAATAAAGTCATCAAGGGAAGCGGAAAGAGAGGGATTCGAACCCTCG** 129

**Hoya kerrii NC_069570.1**  **AAAA---------AAA--TAAATAAATAAAGTCATCAAGGGAAGCGGAAAGAGAGGGATTCGAACCCTCG** 123

**Hoya lacunosa NC_069564.1**  **------------------TAAATAAATAAAGTCATCAAGGGAAGCGGAAAGAGAGGGATTCGAACCCTCG** 114

**Hoya lanceolata NC_067960.1**  **TGAAAAAAAAAAATAAATAAAATAAATAAAGTCATCAAGGTAAGGGGAAAGAGAGGGATTCGAACCCTCG** 140

**Hoya liangii OL826865.1**  **TGAAAAAAAA----TAAATAAATAAATAAAGTCATCAAGGGAAGCGGAAAGAGAGGGATTCGAACCCTCG** 130

**Hoya lithophytica MW719058.1**  **TGAAAAAAAAAATA-AAATAAATAAATAAAGTCATCAAGGGAAGCGGAAAGAGAGGGATTCGAACCCTCG** 133

**Hoya lockii OR475243.1**  **CGAAAAAAT----------AAATAAATAAAGTCATCAAGGGAAGCGGAAAGAGAGGGATTCGAACCCTCG** 124

**Hoya longifolia NC_069560.1**  **TGAAAAAAAA--A-AAAATAAATAAATAAAGTCATCAAGGGAAGCGGAAAGAGAGGGATTCGAACCCTCG** 131

**Hoya lyi MW719055.1**  **AAAA--------------TAAATAAATAAAGTCATCAAGGGAAGCGGAAAGAGAGGGATTCGAACCCTCG** 120

**Hoya megalaster MW719063.1**  **TGAAAAAAAAA-AATTAAAAAATAAATAAAGTCATCAAGGGAAGCGGAAAGAGAGGGATTCGAACCCTCG** 133

**Hoya meliflua NC_069571.1**  **AA----------------TAAATAAATAAAGTCATCAAGGGAAGCGGAAAGAGAGGGATTCGAACCCTCG** 118

**Hoya monetteae MW719053.1**  **AAAA---------TAAAATAAATAAATAAAGTCATCAAGGGAAGCGGAAAGAGAGGGATTCGAACCCTCG** 125

**Hoya omlorii MW719060.1**  **TGAAAAAAAAAAAATAAATAAATAAATAAAGTCATCAAGGGAAGCGGAAAGAGAGGGATTCGAACCCTCG** 134

**Hoya ovalifolia NC_069563.1**  **AAAA-AAATA--AATAAATAAATAAATAAAGTCATCAAGGAAAGCGGAAAGAGAGGGATTCGAACCCTCG** 131

**Hoya pandurata NC_069562.1**  **TGAAAAAATA-----AAATAAATAAATAAAGTCATCAAGGGAAGCGGAAAGAGAGGGATTCGAACCCTCG** 124

**Hoya pottsii OL754664.1**  **AA-----------ATAAATAAATAAATAAAGTCATCAAGGGAAGCGGAAAGAGAGGGATTCGAACCCTCG** 123

**Hoya pubicalyx NC_069561.1**  **AAAATAAAAA--AATAAATAAATAAATAAAGTCATCAAGGGAAGCGGAAAGAGAGGGATTCGAACCCTCG** 132

**Hoya radicalis NC_067961.1**  **TGAAAATA----AATAAATAAATAAATAAAGTCATCAAGGGAAGCGGAAAGAGAGGGATTCGAACCCTCG** 130

**Hoya rigida NC_067962.1**  **TGAA-----A--AATAAATAAATAAATAAAGTCATCAAGGGAAGCGGAAAGAGAGGGATTCGAACCCTCG** 118

**Hoya silvatica NC_067963.1**  **AA-----------ATAAATAAATAAATAAAGTCATCAAGGGAAGCGGAAAGAGAGGGATTCGAACCCTCG** 123

**Hoya thomsonii NC_067612.1**  **TGAAAAAAAA--AATAAATAAATAAATAAAGTCATCAAGGGAAGCGGAAAGAGAGGGATTCGAACCCTCG** 132

**Hoya verticillata NC_085236.1**  **-------------ATAAATAAATAAATAAAGTCATCAAGGAAAGCGGAAAGAGAGGGATTCGAACCCTCG** 121

**Papuahoya urniflora MW719062.1** **TGAAAAAAAAAA-AGAAAAAAATAAATAAAGTCATCAAGGGAAGCGGAAAGAGAGGGATTCGAACCCTCG** 133

**Stephanotis volubilis OP133576** **TGAA---------AAAAATAAATAAATAAAGTCATCAAGGGAAGCGGAAAGAGAGGGATTCGAACCCTCG** 123

**150 160 170 180 190 200 210**

**....|....|....|....|....|....|....|....|....|....|....|....|....|....|**

**Dischidia australis NC_067885.** **GTACGAATAACTCGTACAACGGATTAGCAATCCGCCGCTTTAGTCCACTCAGCCATCTCTCCTAATTGAA** 194

**Gymnema yunnanense NC_079598.1** **GTACGAATAACTCGTACAACGGATTAGCAATCCGCCGCTTTAGTCCACTCAGCCATCTCTCCTAATTGAA** 191

**Hoya ariadna NC_069568.1**  **GTACGAATAACTCGTACAACGGATTAGCAATCCGCCGCTTTAGTCCACTCAGCCATCTCTCCTAATTGAA** 203

**Hoya commutata NC_067958.1**  **GTACGAATAACTCGTACAACGGATTAGCAATCCGCCGCTTTAGTCCACTCAGCCATCTCTCCTAATTGAA** 193

**Hoya dimorpha NC_067959.1**  **GTACGAATAACTCGTACAACGGATTAGCAATCCGCCGCTTTAGTCCACTCAGCCATCTCTCCTAATTGAA** 203

**Hoya exilis MW719054.1**  **GTACGAATAACTCGTACAACGGATTAGCAATCCGCCGCTTTAGTCCACTCAGCCATCTCTCCTAATTGAA** 199

**Hoya griffithii NC_069565.1**  **GTACGAATAACTCGTACAACGGATTAGCAATCCGCCGCTTTAGTCCACTCAGCCATCTCTCCTAATTGAA** 199

**Hoya kerrii NC_069570.1**  **GTACGAATAACTCGTACAACGGATTAGCAATCCGCCGCTTTAGTCCACTCAGCCATCTCTCCTAATTGAA** 193

**Hoya lacunosa NC_069564.1**  **GTACGAATAACTCGTACAACGGATTAGCAATCCGCCGCTTTAGTCCACTCAGCCATCTCTCCTAATTGAA** 184

**Hoya lanceolata NC_067960.1**  **GTACGAATAACTCGTACAACGGATTAGCAATCCGCCGCTTTAGTCCACTCAGCCATCTCTCCTAATTGAA** 210

**Hoya liangii OL826865.1**  **GTACGAATAACTCGTACAACGGATTAGCAATCCGCCGCTTTAGTCCACTCAGCCATCTCTCCTAATTGAA** 200

**Hoya lithophytica MW719058.1**  **GTACGAATAACTCGTACAACGGATTAGCAATCCGCCGCTTTAGTCCACTCAGCCATCTCTCCTAATTGAA** 203

**Hoya lockii OR475243.1**  **GTACGAATAACTCGTACAACGGATTAGCAATCCGCCGCTTTAGTCCACTCAGCCATCTCTCCTAATTGAA** 194

**Hoya longifolia NC_069560.1**  **GTACGAATAACTCGTACAACGGATTAGCAATCCGCCGCTTTAGTCCACTCAGCCATCTCTCCTAATTGAA** 201

**Hoya lyi MW719055.1**  **GTACGAATAACTCGTACAACGGATTAGCAATCCGCCGCTTTAGTCCACTCAGCCATCTCTCCTAATTGAA** 190

**Hoya megalaster MW719063.1**  **GTACGAATAACTCGTACAACGGATTAGCAATCCGCCGCTTTAGTCCACTCAGCCATCTCTCCTAATTGAA** 203

**Hoya meliflua NC_069571.1**  **GTACGAATAACTCGTACAACGGATTAGCAATCCGCCGCTTTAGTCCACTCAGCCATCTCTCCTAATTGAA** 188

**Hoya monetteae MW719053.1**  **GTACGAAAAACTCGTACAACGGATTAGCAATCCGCCGCTTTAGTCCACTCAGCCATCTCTCCTAATTGAA** 195

**Hoya omlorii MW719060.1**  **GTACGAATAACTCGTACAACGGATTAGCAATCCGCCGCTTTAGTCCACTCAGCCATCTCTCCTAATTGAA** 204

**Hoya ovalifolia NC_069563.1**  **GTACGAATAACTCGTACAACGGATTAGCAATCCGCCGCTTTAGTCCACTCAGCCATCTCTCCTAATTGAA** 201

**Hoya pandurata NC_069562.1**  **GTACGAATAACTCATACAACGGATTAGCAATCCGCCGCTTTAGTCCACTCAGCCATCTCTCCTAATTGAA** 194

**Hoya pottsii OL754664.1**  **GTACGAATAACTCGTACAACGGATTAGCAATCCGCCGCTTTAGTCCACTCAGCCATCTCTCCTAATTGAA** 193

**Hoya pubicalyx NC_069561.1**  **GTACGAATAACTCGTACAACGGATTAGCAATCCGCCGCTTTAGTCCACTCAGCCATCTCTCCTAATTGAA** 202

**Hoya radicalis NC_067961.1**  **GTACGAATAACTCGTACAACGGATTAGCAATCCGCCGCTTTAGTCCACTCAGCCATCTCTCCTAATTGAA** 200

**Hoya rigida NC_067962.1**  **GTACGAATAACTCGTACAACGGATTAGCAATCCGCCGCTTTAGTCCACTCAGCCATCTCTCCTAATTGAA** 188

**Hoya silvatica NC_067963.1**  **GTACGAATAACTCGTACAACGGATTAGCAATCCGCCGCTTTAGTCCACTCAGCCATCTCTCCTAATTGAA** 193

**Hoya thomsonii NC_067612.1**  **GTACGAATAACTCGTACAACGGATTAGCAATCCGCCGCTTTAGTCCACTCAGCCATCTCTCCTAATTGAA** 202

**Hoya verticillata NC_085236.1**  **GTACGAATAACTCGTACAACGGATTAGCAATCCGCCGCTTTAGTCCACTCAGCCATCTCTCCTAATTGAA** 191

**Papuahoya urniflora MW719062.1** **GTACGAATAACTCGTACAACGGATTAGCAATCCGCCGCTTTAGTCCACTCAGCCATCTCTCCTAATTGAA** 203

**Stephanotis volubilis OP133576** **GTACGAATAACTCGTACAACGGATTAGCAATCCGCCGCTTTAGTCCACTCAGCCATCTCTCCTAATTGAA** 193

**220 230 240 250 260 270 280**

**....|....|....|....|....|....|....|....|....|....|....|....|....|....|**

**Dischidia australis NC_067885.** **AATTGGGTTAGATTACACATAA----AAAAAAA-TAAGGAGTATTTCTTTCTCTATTATATAGATTATAT** 259

**Gymnema yunnanense NC_079598.1** **AATTAGATTAGATTACACATAAAAACAAAACAAATAAGGAGTATTTCTTTCTCTATTATATAGAT-----** 256

**Hoya ariadna NC_069568.1**  **AATTAGGTTAGATTACACATAA----AAAAAA--TAAGGAGTATTTCTTTCTATATTATATAGAT-----** 262

**Hoya commutata NC_067958.1**  **AATTGGGTTAGATTACACATAA----AAAAAA--TAAGGAGTATTTCTTTCTCTATTATATAGAT-----** 252

**Hoya dimorpha NC_067959.1**  **AATTGGGTTAGATTACACATAA----AAAAAAA-TAAGGAGTATTTCTTTCTCTATTATATAGAT-----** 263

**Hoya exilis MW719054.1**  **AATTGGGTTAGATTACACATAA----AAAAAA--TAAGGAGTATTTCTTTCTCTATTATATAGAT-----** 258

**Hoya griffithii NC_069565.1**  **AATTGGGTTAGATTACACATAA----AAAAAA--TGAAGAGTATTTCTTTCTCTATTATATAGAT-----** 258

**Hoya kerrii NC_069570.1**  **AATTGGGTTAGATTACACATAA----AAAAAAA-TAAGGAGTATTTCTTTCTCTATTATATAGAT-----** 253

**Hoya lacunosa NC_069564.1**  **AATTGGGTTAGATTACACATAA----AAAAAA--TAAGGAGTATTTCTTTCTCTATTATATAGAT-----** 243

**Hoya lanceolata NC_067960.1**  **AATTGGGTTAAATTACACATAA----AAAAAA--TAAGGAGTATTTCTTTCTCTATTATATAGAT-----** 269

**Hoya liangii OL826865.1**  **AATTGGGTTAGATTACACATAA----AAAAAA--TGAAGAGTATTTCTTTCTCTATTATATAGAT-----** 259

**Hoya lithophytica MW719058.1**  **AATTAGGTTAGATTACACATAA----AAAAAA--GAAGGAGTATTTCTTTCTCTATTATATAGAT-----** 262

**Hoya lockii OR475243.1**  **AATTGGGTTAGATTACACATAA----AAAAAAAATAAGGAGTATCCCTTTCTCTATTATATAGAT-----** 255

**Hoya longifolia NC_069560.1**  **AATTGGGTTAGATTACACATAA----AAAAAA--TGAAGAGTATTTCTTTCTCTATTATATAGAT-----** 260

**Hoya lyi MW719055.1**  **AATTGGGTTAGATTACACATAA----AAAAAA--TAAGGAGTATTTCTTTCTCTATTATATAGAT-----** 249

**Hoya megalaster MW719063.1**  **AATTGGGTTAGATTACACATTA----AAAAAAA-TAAGGAGTATTTCTTTCTCTATTATATAGAT-----** 263

**Hoya meliflua NC_069571.1**  **AATTGGGTTAGATTACACATAA----AAAAAA--GAAGGAGTATTTCTTTCTCTATTATATAGAT-----** 247

**Hoya monetteae MW719053.1**  **AATTGGGTTAGATTACACATAA----AAAAAA--TAAGGAGTATTTCTTTCTCTATTATATAGAT-----** 254

**Hoya omlorii MW719060.1**  **AATTGGGTTAGATTACACATAA----AAAAAA--TAAGGAGTATTTCTTTCTCTATTATATAGAT-----** 263

**Hoya ovalifolia NC_069563.1**  **AATTGGGTTAGATTACACATAA----AAAAAA--TAAGGAGTATTTCTTTCTCTATTATATAGAT-----** 260

**Hoya pandurata NC_069562.1**  **AATTGGGTTAGATTACACATAA----AAAAAAAATAAAGAGTATTTCTTTCTCTATTATATAGAT-----** 255

**Hoya pottsii OL754664.1**  **AATTGGGTTAGATTACACATAA----AAAAAA--GAAGGAGTATTTCTTTCTCTATTATATAGAT-----** 252

**Hoya pubicalyx NC_069561.1**  **AATTGGGTTAGATTACACATAA----AAAAAAA-TAAGGAGTATTTCTTTCTCTATTATATAGAT-----** 262

**Hoya radicalis NC_067961.1**  **AATTGGGTTAGATTACACAT-A----AAAAAAA-TAAAGAGTATTTCTTTCTCTATTATATAGAT-----** 259

**Hoya rigida NC_067962.1**  **AATTGGGTTAGATTACACATAA----AAAAAA--GAAGGAGTATTTCTTTCTCTATTATATAGAT-----** 247

**Hoya silvatica NC_067963.1**  **AATTGGGTTAGATTACACATAA----AAAAAA--GAAGGAGTATTTCTTTCTCTATTATATAGAT-----** 252

**Hoya thomsonii NC_067612.1**  **AATTGGGTTAGATTACACATAA----AAAAAAA-TAAAGAGTATTTCTTTCTCTATTATATAGAT-----** 262

**Hoya verticillata NC_085236.1**  **AATTGGGTTAGATTACACATAA----AAAAAA--TAAGGAGTATTTCTTTCTCTATTATATAGAT-----** 250

**Papuahoya urniflora MW719062.1** **AATTGGGTTAGATTACACATAA----AAAAAA--TAAGGAGTATTTCTTTCTCTATTATATAGAT-----** 262

**Stephanotis volubilis OP133576** **AATTAGATTAGATTACACATAA----AAAAAAAATAAGGAGTATTTCTTTCTCTATTATATAGAT-----** 254

**290 300 310 320 330 340 350**

**....|....|....|....|....|....|....|....|....|....|....|....|....|....|**

**Dischidia australis NC_067885.** **AGATATGTAC------------------TATTTTTATCAGTAATTTTTTTT--CGATAAATATAAATATA** 309

**Gymnema yunnanense NC_079598.1** **----ATGTAC------------------AATTTTTATCAGTAATTTATTTT--CGATAAATA--------** 294

**Hoya ariadna NC_069568.1**  **----ATGTAC------------------AATTTTTATCAGTAATTTATTTT--CGATAAATA--------** 300

**Hoya commutata NC_067958.1**  **----ATTTAC------------------CATTTTTATCAGTAATTCTTTTT--CGATAAATA--------** 290

**Hoya dimorpha NC_067959.1**  **----ATGTAC------------------AATTTTTATCAATAATTTTTTTTT-CGATAAATA--------** 302

**Hoya exilis MW719054.1**  **----ATGTAC------------------AATTTTTATCAGTAATTTCTTTT--CGATAAATA--------** 296

**Hoya griffithii NC_069565.1**  **----ATGTGC------------------CATTTTTATCAGTAATTTTTTTTT-CGATAAATA--------** 297

**Hoya kerrii NC_069570.1**  **----ATGTAC------------------CTTTTTTATCAGTAATTTTTTTT--CGATAAATA--------** 291

**Hoya lacunosa NC_069564.1**  **----ATGTAC------------------CATTTTTATCAGTAATTTTTTTT--CGATAAATA--------** 281

**Hoya lanceolata NC_067960.1**  **----ATGCAC------------------AATTTTTATCAGTAATTTTTTTTT-CGATAAATA--------** 308

**Hoya liangii OL826865.1**  **----ATGTGC------------------CATTTTTATCAGTAATTTTTTTTT-CGATAAATA--------** 298

**Hoya lithophytica MW719058.1**  **----ATGTAC------------------AATTTTTATCAGTAATTTTTTTT--CGATAAATA--------** 300

**Hoya lockii OR475243.1**  **----ATCTAC------------------AATTTTTATCAGTAATTTATTTT--CGATAAATA--------** 293

**Hoya longifolia NC_069560.1**  **----ATGTGC------------------CATTTTTATCAGTAATTTTTTTTT-CGATAAATA--------** 299

**Hoya lyi MW719055.1**  **----ATGTAC------------------CATTTTTATCAGTAATTTTTTTT--CGATAAATA--------** 287

**Hoya megalaster MW719063.1**  **----ATGTAC------------------AATTTTTATCAATAATTTTTTTT--CGATAAATA--------** 301

**Hoya meliflua NC_069571.1**  **----ATGTAC------------------CATTTTTATCAGTAATTTTTTTTT-CGATAAATA--------** 286

**Hoya monetteae MW719053.1**  **----ATGTAC------------------CTTTTTTATCAGTAATTTTTTTT--CGATAAATA--------** 292

**Hoya omlorii MW719060.1**  **----ATGTAC------------------AATTTTTATCAGTAATTTTTTTT--CGATAAATA--------** 301

**Hoya ovalifolia NC_069563.1**  **----ATGTAC------------------CATTTTTATCAGTAATTTTTTTT--CGATAAATA--------** 298

**Hoya pandurata NC_069562.1**  **----ATGTAC------------------CATTTTTATCAGTAATTTTTTTTTTCGATAAATA--------** 295

**Hoya pottsii OL754664.1**  **----ATTTAC------------------CATTTTTATCAGTAATTTTTTTT--CGATAAATA--------** 290

**Hoya pubicalyx NC_069561.1**  **----ATGTACTATTATATAGATATGTACCATTTTTATCAGTAATTTTTTTT--CGATAAATA--------** 318

**Hoya radicalis NC_067961.1**  **----ATGTAC------------------CATTTTTATCAGTAATTTTTTTTT-CGATAAATA--------** 298

**Hoya rigida NC_067962.1**  **----ATGTAC------------------CATTTTTATCAGTAATTTTTTTTT-CGATAAATA--------** 286

**Hoya silvatica NC_067963.1**  **----ATTTAC------------------CATTTTTATCAGTAATTTTTTTT--CGATAAATA--------** 290

**Hoya thomsonii NC_067612.1**  **----ATGTAC------------------CATTTTTATCAGTAATTTTTTTTT-CGATAAATA--------** 301

**Hoya verticillata NC_085236.1**  **----ATGTAC------------------CATTTTTATCAGTAATTTTTTTT--CGATAAATA--------** 288

**Papuahoya urniflora MW719062.1** **----ATGTAC------------------AATTTTTATCAGTAATTTATTTT--CGATAAATA--------** 300

**Stephanotis volubilis OP133576** **----ATGTAC------------------AATTTTTATCAGTAATTTATTTT--CAATAAATA--------** 292

**360 370 380 390 400 410 420**

**....|....|....|....|....|....|....|....|....|....|....|....|....|....|**

**Dischidia australis NC_067885.** **AATAAAGAATAAATGAAATAAATAAAGAATAAATAAAGAAAAGGGCTCGAAAGTGCCAACAA--------** 371

**Gymnema yunnanense NC_079598.1** **----AAGAA-------------------------------AAGGGCTCGAAAGTGCCAACAA--------** 321

**Hoya ariadna NC_069568.1**  **----AAGAATAAATGAAATAAATAAAGAATAAATAAAGAAAAGGGCTCGAAAGTGCCAACAA--------** 358

**Hoya commutata NC_067958.1**  **----AAGAA---------TAAATAAAGA-----------GAAGGGCTCGAAAGTGCCAACAA--------** 328

**Hoya dimorpha NC_067959.1**  **----AAGAA---------TAAATAAAGA-----------AAAGGGCTCGAAAGTGCCAACAA--------** 340

**Hoya exilis MW719054.1**  **----AAGAA--------------AAAGA-----------AAAGGGCTCGAAAGTGCCAACAA--------** 329

**Hoya griffithii NC_069565.1**  **----AAGAA---------TAAATAAGGA-----------GAAGGGCTCGAAAGTGCCAACAA--------** 335

**Hoya kerrii NC_069570.1**  **----AAGAA---------TAAATAAAGA-----------GAAGGGCTCGAAAGTGCCAACAA--------** 329

**Hoya lacunosa NC_069564.1**  **----AAGAA---------TAAATAAAGA-----------GAAGGGCTCGAAAGTGCCAACAA--------** 319

**Hoya lanceolata NC_067960.1**  **----AAGAA---------TAAATAAAGAA----------AA-GGGCTCGAAAGTGCCAACAA--------** 346

**Hoya liangii OL826865.1**  **----AAGAA---------TAAATAAGGA-----------GAAGGGCTCGAAAGTGCCAACAA--------** 336

**Hoya lithophytica MW719058.1**  **----AAGAATAAATGAAATAAATAAAGAATAAATAAAGAAAAGGGCTCGAAAGTGCCAACAA--------** 358

**Hoya lockii OR475243.1**  **----AAGAA---------TAAATAAAGAA----------AAAGGGCTCAAAAGTGCCAACAA--------** 332

**Hoya longifolia NC_069560.1**  **----AAGAA---------TAAATAAGGA-----------GAAGGGCTCGAAAGTGCCAACAA--------** 337

**Hoya lyi MW719055.1**  **----AAGAA---------TAAATAAAGAATAAATAAAGAGAAGGGCTCGAAAGTGCCAACAA--------** 336

**Hoya megalaster MW719063.1**  **----AAGAA---------TAAATAAAGA-----------AAAGGGCTCGAAAGTGCCAACAA--------** 339

**Hoya meliflua NC_069571.1**  **----AAGAA---------TAAATAAAGA-----------GAAGGGCTCGAAAGTGCCAACAAAACAATAT** 332

**Hoya monetteae MW719053.1**  **----AAGAA---------TAAATAAAGA-----------GAAGGGCTCGAAAGTGACAACAA--------** 330

**Hoya omlorii MW719060.1**  **----AAGAATAAATGAAATAAATAAAGAATAAATAAAGAAAAGGGCTCGAAAGTGCCAACAA--------** 359

**Hoya ovalifolia NC_069563.1**  **----AAGAA---------TAAATAAAGA-----------GAAGGGCTCGAAAGTGCCAACAA--------** 336

**Hoya pandurata NC_069562.1**  **----AAGAA---------TAAATAAAGA-----------GAAGGGCTCGAAAGTGCCAACAA--------** 333

**Hoya pottsii OL754664.1**  **----AAGAA---------TAAATAAAGA-----------GAAGGGCTCGAAAGTGCCAACAA--------** 328

**Hoya pubicalyx NC_069561.1**  **----AAGAA---------TAAATAAAGA-----------GAAGGGCTCGAAAGTGCCAACAA--------** 356

**Hoya radicalis NC_067961.1**  **----AAGAA---------TAAATAAAGA-----------GAAGGGCTCGAAAGTGCCAACAA--------** 336

**Hoya rigida NC_067962.1**  **----AAGAA---------TAAATAAAGA-----------GAAGGGCTCGAAAGTGCCAACAA--------** 324

**Hoya silvatica NC_067963.1**  **----AAGAA---------TAAATAAAGA-----------GAAGGGCTCGAAAGTGCCAACAA--------** 328

**Hoya thomsonii NC_067612.1**  **----AAGAA---------TAAATAAAGA-----------GAAGGGCTCGAAAGTGCCAACAA--------** 339

**Hoya verticillata NC_085236.1**  **----AAGAAATAAAGAA-TAAATAAAGA-----------GAAGGGCTCGAAAGTGCCAACAA--------** 334

**Papuahoya urniflora MW719062.1** **----AAGAATAAATGAAATAAATAAAGAATAAATAAAGAAAAGGGCTCGAAAGTGCCAACAA--------** 358

**Stephanotis volubilis OP133576** **----AAGAA-------------------------------AAGGGCTCGAAAGTGCCAACAA--------** 319

**430 440 450 460 470 480 490**

**....|....|....|....|....|....|....|....|....|....|....|....|....|....|**

**Dischidia australis NC_067885.** **--TATAAAGAAAACAAAAAGCGACCCCTTCGTATTTTGTTCGAAAGACCCTTCTTATT------------** 427

**Gymnema yunnanense NC_079598.1** **--TATAAAGAAAACAAAAAGCGACCCCTTCGTATTTTGTTCGAAAGACCCTTCTTATTGATTAT------** 383

**Hoya ariadna NC_069568.1**  **--TATAAAGAAAACAAAAAGCGACCCCTTCGTATTTTGTTCGAAAGACCCTTCTTATT------------** 414

**Hoya commutata NC_067958.1**  **--TATAAAGAAAACCAAAAGCGACCCCTTCGTATTTTGTTCGAAAGACCCTTCTTATTGTTAT-TT---G** 392

**Hoya dimorpha NC_067959.1**  **--TATAAAGAAAACAAAAAGCGACCCCTTCGTATTTTGTTCGAAAGACCCTTCTTATTGTTATTTTATTG** 408

**Hoya exilis MW719054.1**  **--TATAAAGAAAAGAAAAAGCGACCCCTTCGTATTTTGTTCGAAAGACCCTTGTTATTGTTATT------** 391

**Hoya griffithii NC_069565.1**  **--TATAAAGAAAACCAAAAGCGACCCCTTCGTATTTTGTTCGAAAGACCCTTCTTATTGTTATT------** 397

**Hoya kerrii NC_069570.1**  **--TATAAAGAAAACCAAAAGCGACCCCTTCGTATTTTGTTCGAAAGACCCTTCTTATTGTTATT------** 391

**Hoya lacunosa NC_069564.1**  **--TATAAAGAAAACCAAAAGTGACCCCTTCGTATTTTGTTCGAAAGACCCTTCTTATTGTTATTT----G** 383

**Hoya lanceolata NC_067960.1**  **--TATAAAGAAAACAAAAAGAA---------TATAAAGAAAACAAAAAGCGACCCCTTCGTATTTTGTTC** 405

**Hoya liangii OL826865.1**  **--TATAAAGAAAACCAAAAGCGACCCCTTCGTATTTTGTTCGAAAGACCCTTCTTATTGTTATT------** 398

**Hoya lithophytica MW719058.1**  **--TATAAAGAAAACAAAAAGCGACCCCTTCGTATTTTGTTCGAAAGACCCTTCTTATT------------** 414

**Hoya lockii OR475243.1**  **--TATAAAGAAAACAAAAAGCGACCCCTTCGTATTTTGTTCGAAAGACCCTTCTTATTTTTAATTATTTT** 400

**Hoya longifolia NC_069560.1**  **--TATAAAGAAAACCAAAAGCGACCCCTTCGTATTTTGTTCGAAAGACCCTTCTTATTGTTATT------** 399

**Hoya lyi MW719055.1**  **--TATAAAGAAAACCAAAAGCGACCCCTTCGTATTTTGTTCGAAAGACCCTTCTTATTGTTATT------** 398

**Hoya megalaster MW719063.1**  **--TATAAAGAAAACAAAAAGCGACCCCTTCGTATTTTGTTCGAAAGACCCTTCTTATTGT----------** 397

**Hoya meliflua NC_069571.1**  **AATATAAAGAAAACCAAAAGCGACCCCTTCGTATTTTGTTCGAAAGACCCTTCTTATTGTTATT------** 396

**Hoya monetteae MW719053.1**  **--TATAAAGAAAACCAAAAGCGACCCCTTCGTATTTTGTTCGAAAGACCCTTCTTATTGTTATT------** 392

**Hoya omlorii MW719060.1**  **--TATAAAGAAAACAAAAAGCGACCCCTTCGTATTTTGTTCGAAAGACCCTTCTTATT------------** 415

**Hoya ovalifolia NC_069563.1**  **--TATAAAGAAAACCAAAAGCGACCCCTTCGTATTTTGTTCGAAAGACCCTTCTTATTGTTAT-------** 397

**Hoya pandurata NC_069562.1**  **--TATAAAGAAAACCAAAAGCGACCCCTTCGTATTTTGTTCGAAAGACCCTTCTTATTGTTATT------** 395

**Hoya pottsii OL754664.1**  **--TATAAAGAAAACCAAAAGCGACCCCTTCGTATTTTGTTCGAAAGACCCTTCTTATTGTTTTATT---G** 393

**Hoya pubicalyx NC_069561.1**  **--TATAAAGAAAACCAAAAGCGACCCCTTCGTATTTTGTTCGAAAGACCCTTCTTATTGTTAT-------** 417

**Hoya radicalis NC_067961.1**  **--TATAAAGAAAACCAAAAGCGACCCCTTCGTATTTTGTTCGAAAGACCCTTCTTATTGT----------** 394

**Hoya rigida NC_067962.1**  **--TATAAAGAAAACCAAAAGCGACCCCTTCGTATTTTGTTCGAAAGACCCTTCTTATTGTTATT------** 386

**Hoya silvatica NC_067963.1**  **--TATAAAGAAAACCAAAAGCGACCCCTTCGTATTTTGTTCGAAAGACCCTTCTTATTGTTTTATT---G** 393

**Hoya thomsonii NC_067612.1**  **--TATAAAGAAAACCAAAAGCGACCCCTTCGTATTTTGTTCGAAAGACCCTTCTTATTGTTATT------** 401

**Hoya verticillata NC_085236.1**  **--TATAAAGAAAACCAAAAGCGACCCCTTCGTATTTTGTTCGAAAGACCCTTCTTATTGTTAT-------** 395

**Papuahoya urniflora MW719062.1** **--TATAAAGAAAACAAAAAGTGACCCCTTCGTATTTTGTTCGAAAGACCCTTCTTATT------------** 414

**Stephanotis volubilis OP133576** **--TATAAAGAAAACAAAAAGCGACCCCTTCGTATTTTGTTCGAAAGACCCTTCTT-------AT------** 374

**500 510 520 530 540 550 560**

**....|....|....|....|....|....|....|....|....|....|....|....|....|....|**

**Dischidia australis NC_067885.** **--------------------------------GATACGGCCTGGTCTGGTCAGTACCCAGCCGGGCCTCT** 465

**Gymnema yunnanense NC_079598.1** **-------------------------------TGACACGGCCTGGCCTGGTCAGTACCCAGCCGGGCCTCT** 422

**Hoya ariadna NC_069568.1**  **--------------------------------GACACGGCCTGGTCTGGTCAGTACCCAGCCGGGCCTCT** 452

**Hoya commutata NC_067958.1**  **-------T--------------------TATTGACACGGCCTGGTCTGGTCAGTACCCAGCCGGGCCTCT** 435

**Hoya dimorpha NC_067959.1**  **-------T--------------------TATTGACACGGCCTGGTCTGGTCAGTACCCAGCCGGGCCTCT** 451

**Hoya exilis MW719054.1**  **--------------------------------GACACGGCCTGGTCTGGTCAGTACCCAGCCGGGCCTCT** 429

**Hoya griffithii NC_069565.1**  **--------------------------------GACACGGCCTGGTCTGGTCAGTACCCAGCCGGGCCTCT** 435

**Hoya kerrii NC_069570.1**  **--------------------------------GACACGGCCTGGTCTGGTCAGTACCCAGCCGGGCCTCT** 429

**Hoya lacunosa NC_069564.1**  **-------T--------------------TATTGACACGGCCTGGTCTGGTCAGTACCCAGCCGGGCCTCT** 426

**Hoya lanceolata NC_067960.1**  **GAAAG--ACCCTTCTTATTGTTTATTGTTATTGACACGGCCTGGTCTGGTCAGTACCCAGCCGGGCCTCT** 473

**Hoya liangii OL826865.1**  **--------------------------------GACACGGCCTGGTCTGGTCAGTACCCAGCCGGGCCTCT** 436

**Hoya lithophytica MW719058.1**  **--------------------------------GACACGGCCTGGTCTGGTCAGTACCCAGCCGGGCCTCT** 452

**Hoya lockii OR475243.1**  **TAATTTTATTTATTTTATTGTTTATTGTTATTGACACGGCCTGGGCTGGTCAGTACCCAGCCGGGCCTCC** 470

**Hoya longifolia NC_069560.1**  **--------------------------------GACACGGCCTGGTCTGGTCAGTACCCAGCCGGGCCTCT** 437

**Hoya lyi MW719055.1**  **--------------------------------GACACGGCCTGGTCTGGTCAGTACTCAGCCGGGCCTCT** 436

**Hoya megalaster MW719063.1**  **----------------------------TATTGACACGGCCTGGTCTGGTCAGTACCCAGCCGGGCCTCT** 439

**Hoya meliflua NC_069571.1**  **--------------------------------GACACGGCCTGGTCTGGTCAGTACCCAGCCGGGCCTCT** 434

**Hoya monetteae MW719053.1**  **--------------------------------GACACGGCCTGGTCTGGTCAGTACCCAGCCGGGCCTCT** 430

**Hoya omlorii MW719060.1**  **--------------------------------GACACGGCCTGGTCTGGTCAGTACCCAGCCGGGCCTCT** 453

**Hoya ovalifolia NC_069563.1**  **-------------------------------TGACACGGCCTGGTCTGGTCAGTACCCAGCCGGGCCTCT** 436

**Hoya pandurata NC_069562.1**  **--------------------------------GATACGGCCTGGTCTGGTCAGTACCCAGCCGGGCCTCT** 433

**Hoya pottsii OL754664.1**  **-------T--------------------TATTGACACGGCCTGGTCTGGTCAGTACCCAGCCGGGCCTCT** 436

**Hoya pubicalyx NC_069561.1**  **-------------------------------TGACACGGCCTGGTCTGGTCAGTACCCAGCCGGGCCTCT** 456

**Hoya radicalis NC_067961.1**  **----------------------------TATTGACACGGCCTGGTCTGGTCAGTACCCAGCCGGGCCTCT** 436

**Hoya rigida NC_067962.1**  **--------------------------------GACACGGCCTGGTCTGGTCAGTACCCAGCCGGGCCTCT** 424

**Hoya silvatica NC_067963.1**  **-------T--------------------TATTGACACGGCCTGGTCTGGTCAGTACCCAGCCGGGCCTCT** 436

**Hoya thomsonii NC_067612.1**  **--------------------------------GACACGGCCTGGTCTGGTCAGTACCCAGCCGGGCCTCT** 439

**Hoya verticillata NC_085236.1**  **-------------------------------TGACACGGCCTGGTCTGGTCAGTACCCAGCCGGGCCTCT** 434

**Papuahoya urniflora MW719062.1** **--------------------------------GACACGGCCTGGTCTGGTCAGTACCCAGCCGGGCCTCT** 452

**Stephanotis volubilis OP133576** **-------------------------------TGACACGGCCTGGCCTGGTCAGTACCCAGCCGGGCCTCT** 413

**570 580 590 600 610 620 630**

**....|....|....|....|....|....|....|....|....|....|....|....|....|....|**

**Dischidia australis NC_067885.** **TTTTGTTTTGTTCCAACTAATTATAGAAAAATAATGATAATTTTTAATTTATCTGA------------TT** 523

**Gymnema yunnanense NC_079598.1** **TTTTGTTTTGTTCCAACTAATTATAGAAAAATAATGATGATTTTTCGTTTATCTGA------------TT** 480

**Hoya ariadna NC_069568.1**  **TTTTGTTTTGTTCCAACTAATTATAGAAAAATAATGATAATTTTTAATTTATCTGA------------TT** 510

**Hoya commutata NC_067958.1**  **TTTTGTTTTGTTCCAACTAATTCTAGAAAAATAATGAGAATTTTTCATTTATCTGA------------TT** 493

**Hoya dimorpha NC_067959.1**  **TTTTGTTTTGTTCCAACTAATTATAGAAAAATAATGATAATTTTTCATTTATCTGATTTGATATCTGATT** 521

**Hoya exilis MW719054.1**  **TTTTGTTTTGTTCCAACTAATTATAGAAAAATAATGATAATTTTTCATTTATCTGA------------TT** 487

**Hoya griffithii NC_069565.1**  **TTTTGTTTTGTTCCAACTAATTATAGAAAAATAATGATAATTTTTCATTTATCTGA------------TT** 493

**Hoya kerrii NC_069570.1**  **TTTTGTTTTGTTCCAACTAATTATAGAAAAATAATGATAATTTTTCATTTATCTGA------------TT** 487

**Hoya lacunosa NC_069564.1**  **TTTTGTTTTGTTCCAACTAATTATAGAAAAATAATGATAATTTTTCATTTATCTGA------------TT** 484

**Hoya lanceolata NC_067960.1**  **TTTTGTTTTGTTCCAACTAATTATAGAAAAATAATGATAATTTTTCATTTATCTGA------------TT** 531

**Hoya liangii OL826865.1**  **TTTTGTTTTGTTCCAACTAATTATAGAAAAATAATGATAATTTTTCATTTATCTGA------------TT** 494

**Hoya lithophytica MW719058.1**  **TTTTGTTTTGTTCCAACTAATTATAGAAATATAATGATAATTTTTAATTTATCTGA------------TT** 510

**Hoya lockii OR475243.1**  **TTTTGTTTTGTTCCAACTAATTTTAGAAAAATAATGGTAATTTTTCATTTATCTGA------------TT** 528

**Hoya longifolia NC_069560.1**  **TTTTGTTTTGTTCCAACTAATTATAGAAAAATAATGATAATTTTTCATTTATCTGA------------TT** 495

**Hoya lyi MW719055.1**  **TTTTGTTTTGTTCCAACTAATTATAGAAAAATAATGATAATTTTTCATTTATCTGA------------TT** 494

**Hoya megalaster MW719063.1**  **TTTTGTTTTGTTCCAACTAATTATAGAAAAATAATGATAATTTTTCATTTATCTGATTTGATATCTGATT** 509

**Hoya meliflua NC_069571.1**  **TTTTGTTTTGTTCCAACTAATTATAGAAAAATAATGATAATTTTTCATTTATCTGA------------TT** 492

**Hoya monetteae MW719053.1**  **TTTTGTTTTGTTCCAACTAATTATAGAAAAATAATGACAATTTTTCATTTATCTGA------------TT** 488

**Hoya omlorii MW719060.1**  **TTTTGTTTTGTTCCAACTAATTATAGAAAAATAATGAGAATTTTTAATTTATCTGA------------TT** 511

**Hoya ovalifolia NC_069563.1**  **TTTTGTTTTGTTCCAACTAATTATAGAAAAATAATGATAATTTTTCATTTATCTGA------------TT** 494

**Hoya pandurata NC_069562.1**  **TTTTGTTTTGTTCCAACTAATTATAGAAAAATAATGATAATTTTTAATTTATCTGA------------TT** 491

**Hoya pottsii OL754664.1**  **TTTTGTTTTGTTCCAACTAATTCTAGAAAAATAATGAGAATTTTTCATTTATCTGA------------TT** 494

**Hoya pubicalyx NC_069561.1**  **TTTTGTTTTGTTCCAACTAATTATAGAAAAATAATGATAATTTTTCATTTATCTGA------------TT** 514

**Hoya radicalis NC_067961.1**  **TTTTGTTTTGTTCCAACTAATTATAGAAAAATAATGATAATTTTTCATTTATCTGA------------TT** 494

**Hoya rigida NC_067962.1**  **TTTTGTTTTGTTCCAACTAATTATAGAAAAATAATGATAATTTTTCATTTATCTGA------------TT** 482

**Hoya silvatica NC_067963.1**  **TTTTGTTTTGTTCCAACTAATTCTAGAAAAATAATGAGAATTTTTCATTTATCTGA------------TT** 494

**Hoya thomsonii NC_067612.1**  **TTTTGTTTTGTTCCAACTAATTATAGAAAAATAATGATAATTTTTCATTTATCTGA------------TT** 497

**Hoya verticillata NC_085236.1**  **TTTTGTTTTGTTCCAACTAATTATAGAAAAATAATGATAATTTTTCATTTATCTGA------------TT** 492

**Papuahoya urniflora MW719062.1** **TTTTGTTTTGTTCCAACTAATTATATAAAAATAATGAGAATTTTTCATTTATCTGA------------TT** 510

**Stephanotis volubilis OP133576** **TTTTGTTTTGTTCCAACTAATTATAGAAAAATAATGATGATTTTTCATTTATCTGA------------TT** 471

**640 650 660 670 680 690 700**

**....|....|....|....|....|....|....|....|....|....|....|....|....|....|**

**Dischidia australis NC_067885.** **TGAAAACAAAAATGCTTAGTATTTTTA---------TTATATA---TTATTTTAT--------------A** 567

**Gymnema yunnanense NC_079598.1** **TGAAAACAAAAATGCTTAGTATTATTAGTA------TTATATAA-ACTTTATTTTTATTATGTA------** 537

**Hoya ariadna NC_069568.1**  **TGAAAACAAAAATGCTTAGTATTTTTA---------GTATTTT---TATTATATA---------------** 553

**Hoya commutata NC_067958.1**  **TGAAAACAAAAATGCTTAGTATTTTTAGTA------TTATATT---ATATATATATTATATAT-------** 547

**Hoya dimorpha NC_067959.1**  **TGAAAACAAAAATGCTTAGTATTTTTA---------TTATATT---TTTTTTATTATATATTAT-----T** 574

**Hoya exilis MW719054.1**  **TGAAAACAAAAATGCTTAGTATTTTTAGTATTTTTATTATATTTTATTATATATTATATTGTATATTATT** 557

**Hoya griffithii NC_069565.1**  **TGAAAACAAAAATGCTTAGTATTTTTAGTA------TTTTAT----ATATATATATTATA----------** 543

**Hoya kerrii NC_069570.1**  **TGAAAACAAAAATGCTTAGTATTTTTAGTA------TTTTAT----ATATATATATTATA----------** 537

**Hoya lacunosa NC_069564.1**  **TGAAAACAAAAATGCTTAGTATTTTTACTA------TTTTTT----ATATAT-TTATATA----------** 533

**Hoya lanceolata NC_067960.1**  **TGAAAACAAAAATGCTTAGT---------ATTTTTATTATAT----ATTTATTATATAT-----------** 577

**Hoya liangii OL826865.1**  **TGAAAACAAAAATGCTTAGTATTTTTAGTA------TTTTAT----ATATATATATTATA----------** 544

**Hoya lithophytica MW719058.1**  **TGAAAACAAAAATTCTTAGTATTTTTA---------GTATTTT---TATTATATATTAATTATA----TA** 564

**Hoya lockii OR475243.1**  **TGAAAACAAAAATGCTTAGTTTTTTTTGTATTTTTTTTATAT----ATTTTTTTTATATATTTT--AATA** 592

**Hoya longifolia NC_069560.1**  **TGAAAACAAAAATGCTTAGTATTTTTAGTA------TTTTAT----ATATATATATTATA----------** 545

**Hoya lyi MW719055.1**  **TGAAAACAAAAATGCTTAGTATTTTTAGTA------TTTTAT----ATATATATATTATAGTAT--TATA** 552

**Hoya megalaster MW719063.1**  **TGAAAACAAAAATGCTTAGTATTTTTAGTATTTTTATTATATT---TTTTTTATTATATATTATA-TAGT** 575

**Hoya meliflua NC_069571.1**  **TGAAAACAAAAATGCTTAGTATTTTTAGTA------TTATAT----ATATATATATTATAGTA-------** 545

**Hoya monetteae MW719053.1**  **TGAAAACAAAAATGCCTAGTATTTTTAGTA------TTTTAT----ATATATATTATATA----------** 538

**Hoya omlorii MW719060.1**  **TGAAAACAAAAATGCTTAGTATTTTTA---------GTATTTT---TATTATAT----------------** 553

**Hoya ovalifolia NC_069563.1**  **TGAAAACAAAAATGCTTAGTATTTTTAGTA------TTATAT----ATATATATATTATA----------** 544

**Hoya pandurata NC_069562.1**  **TGAAAACAAAAATGCTTAGTATTTTTAGTA------TTTTAT----ATATA-------------------** 532

**Hoya pottsii OL754664.1**  **TGAAAACAAAAATGCTTAGTATTTTTAGTA------TTATATT---ATATATATATTAT-----------** 544

**Hoya pubicalyx NC_069561.1**  **TGAAAACAAAAATGCTTAGTATTTTTAGTA------TTATAT----ATATATATAATATA----------** 564

**Hoya radicalis NC_067961.1**  **TGAAAACAAAAATGCTTAGTATTTTTA-----------GTATT---TTATATATTATATAT---------** 541

**Hoya rigida NC_067962.1**  **TGAAAACAAAAATGCTTAGTATTTTTAGTA------TTTTAT----ATATATATATATTAGTAT--TATA** 540

**Hoya silvatica NC_067963.1**  **TGAAAACAAAAATGCTTAGTATTTTTAGTA------TTATATT---ATATATATATTAT-----------** 544

**Hoya thomsonii NC_067612.1**  **TGAAAACAAAAATGCTTAGTATTTTTAGTA------TTATAT----ATATATATATTATA----------** 547

**Hoya verticillata NC_085236.1**  **TGAAAACAAAAATGCTTAGTATTTTTAGTA------TTATAT----ATATATAT--TATA----------** 540

**Papuahoya urniflora MW719062.1** **TGAAAACAAAAATGCTTAGTATTTTTT---------TTATATA---TTATATATTATATAGTAT----TA** 564

**Stephanotis volubilis OP133576** **TGAAAACAAAAATGCTTAGTATTATTAGTA------TTATACAA-ACCTTATT-----------------** 517

**710 720 730 740 750 760 770**

**....|....|....|....|....|....|....|....|....|....|....|....|....|....|**

**Dischidia australis NC_067885.** **TTTATATATATAAATAAATAAAGTAGTATAGAAATAGTATTATATA------------------------** 613

**Gymnema yunnanense NC_079598.1** **TTATTATATAAAATTATGTTTTATTATATAAAGTTAGTATT-----------------------------** 578

**Hoya ariadna NC_069568.1**  **--------------ATTATATATTATATTATATAATTATATATT--------------------------** 583

**Hoya commutata NC_067958.1**  **-----ATATT-ATATATATATATTATTATTATATATAT--------------------------------** 579

**Hoya dimorpha NC_067959.1**  **ATTATATATA-------ATATACTATTA--AATATAGTATTATATAT-TTATATAAT-------------** 621

**Hoya exilis MW719054.1**  **ATTATATAGTATTATATATATAGTATTATATAAACTTTATTATATATATTATATAAAGTTAGTATTATAT** 627

**Hoya griffithii NC_069565.1**  **-----GTATT-ATATATTTATATAATTATATATTTATATATATAAA------------------------** 583

**Hoya kerrii NC_069570.1**  **--TTTATAT--ATATAAA----------------------------------------------------** 551

**Hoya lacunosa NC_069564.1**  **------TAT--ATATATA----------------------------------------------------** 543

**Hoya lanceolata NC_067960.1**  **----TATATTT---------------------------------------------A-------------** 585

**Hoya liangii OL826865.1**  **-----GTATT-ATATATTTATATAATTATATATTTATATATATAAA------------------------** 584

**Hoya lithophytica MW719058.1**  **TTATATAGTATTATATTATATAGTATTATATATTTATATATATATAATTAGTAT----------------** 618

**Hoya lockii OR475243.1**  **AATATATATTTTAATAAATATATATTTTAATAAATATATATTTTAATAAATTATAAA-------------** 649

**Hoya longifolia NC_069560.1**  **-----GTATT-ATATATTTATATAATTATATATTTATATATATAAA------------------------** 585

**Hoya lyi MW719055.1**  **TATTTATAT--ATATAAATACAGTATTATATGTATTATATAAACTTTATTATATATA-------------** 607

**Hoya megalaster MW719063.1**  **ATTATATATATATATAAATATAGTATTATAGAAATAGTATTATATAAACTTTATTAT-------------** 632

**Hoya meliflua NC_069571.1**  **---TTATAT--ATTTATA----------------------------------------------------** 558

**Hoya monetteae MW719053.1**  **----TATAT--ATATATATATTATATATATATATATATAAATACAGTATTATATAAA-------------** 589

**Hoya omlorii MW719060.1**  **--------------ATTATATAGTATTATATATTTATATATATA--------------------------** 583

**Hoya ovalifolia NC_069563.1**  **-----ATAT-------------------------------------------------------------** 548

**Hoya pandurata NC_069562.1**  **----------------------------------------------------------------------** 532

**Hoya pottsii OL754664.1**  **------TATT-ATATAAATATATAATTAT-----------------------------------------** 566

**Hoya pubicalyx NC_069561.1**  **-----GTATT-ATATATTTATATATATAAATACAGTATTATATAAACTTTATTATAT-------------** 615

**Hoya radicalis NC_067961.1**  **---ATATATA---------------------------------------TATATTAT-------------** 556

**Hoya rigida NC_067962.1**  **TATTTATAT--ATATATATATTATTTAACAGTATTATATATAACAGTATTATATAAA-------------** 595

**Hoya silvatica NC_067963.1**  **------TATT-ATATAAATATATAATTAT-----------------------------------------** 566

**Hoya thomsonii NC_067612.1**  **-----GTATT-ATATATTTATATA----------TATAAATACAG-------------------------** 576

**Hoya verticillata NC_085236.1**  **-----ATAT-------------------------------------------------------------** 544

**Papuahoya urniflora MW719062.1** **TATATTTATATATATAAATATAGTATTATATAAATATTATTATATAAACTTTAT----------------** 618

**Stephanotis volubilis OP133576** **-----ATAT--------------TTATATAAAGTTAGTATT-----------------------------** 539

**780 790 800 810 820 830 840**

**....|....|....|....|....|....|....|....|....|....|....|....|....|....|**

**Dischidia australis NC_067885.** **----------------------------------------------------------------------** 613

**Gymnema yunnanense NC_079598.1** **----------------------------------------------------------------------** 578

**Hoya ariadna NC_069568.1**  **----------------------------------------------------------------------** 583

**Hoya commutata NC_067958.1**  **----------------------------------------------------------------------** 579

**Hoya dimorpha NC_067959.1**  **------------------------------ATTTATATATATAAATATACTATTATAGAAATA-GTATTA** 660

**Hoya exilis MW719054.1**  **AAATATATTTTTAATAAATAAATAAGATATATAAATATAAAAAAAAGATATATAAAATATATAAATATAT** 697

**Hoya griffithii NC_069565.1**  **----------------------------------------------------------------------** 583

**Hoya kerrii NC_069570.1**  **----------------------------------------------------------------------** 551

**Hoya lacunosa NC_069564.1**  **----------------------------------------------------------------------** 543

**Hoya lanceolata NC_067960.1**  **------------------------------TTATATAT--------------------------------** 593

**Hoya liangii OL826865.1**  **----------------------------------------------------------------------** 584

**Hoya lithophytica MW719058.1**  **----------------------------------------------------------------------** 618

**Hoya lockii OR475243.1**  **------------------------------TTATATATATATA---------------------------** 662

**Hoya longifolia NC_069560.1**  **----------------------------------------------------------------------** 585

**Hoya lyi MW719055.1**  **------------------------------TATTATAAAGTTAGTATTATATAAATATATTATAGTATTA** 647

**Hoya megalaster MW719063.1**  **------------------------------ATATAT-TATATAAAGTTAGTATTATATAAATATATTTTA** 671

**Hoya meliflua NC_069571.1**  **----------------------------------------------------------------------** 558

**Hoya monetteae MW719053.1**  **------------------------------TATTATATAAACTTATATATATATATATTATAAAGTTAGT** 629

**Hoya omlorii MW719060.1**  **----------------------------------------------------------------------** 583

**Hoya ovalifolia NC_069563.1**  **----------------------------------------------------------------------** 548

**Hoya pandurata NC_069562.1**  **----------------------------------------------------------------------** 532

**Hoya pottsii OL754664.1**  **----------------------------------------------------------------------** 566

**Hoya pubicalyx NC_069561.1**  **------------------------------ATATATATTAATTATATATATATTTATATATTAATTATAT** 655

**Hoya radicalis NC_067961.1**  **------------------------------ATATAT------------------------ATA-------** 565

**Hoya rigida NC_067962.1**  **------------------------------CTTTATTATATAT---------------------------** 608

**Hoya silvatica NC_067963.1**  **----------------------------------------------------------------------** 566

**Hoya thomsonii NC_067612.1**  **----------------------------------------------------------------------** 576

**Hoya verticillata NC_085236.1**  **----------------------------------------------------------------------** 544

**Papuahoya urniflora MW719062.1** **----------------------------------------------------------------------** 618

**Stephanotis volubilis OP133576** **----------------------------------------------------------------------** 539

**850 860 870 880 890 900 910**

**....|....|....|....|....|....|....|....|....|....|....|....|....|....|**

**Dischidia australis NC_067885.** **--------------------AACTTTATTATATATATATTATAAAGTTATA---------------ATAT** 648

**Gymnema yunnanense NC_079598.1** **-----------------------ATAGTATTATTAGTATTATATAAACTT--------------TATTAT** 611

**Hoya ariadna NC_069568.1**  **--------------------ATAAATAA-ATAAATAAAATATATAAATATATAT------------ATAA** 620

**Hoya commutata NC_067958.1**  **----------------------------------------------------------------------** 579

**Hoya dimorpha NC_067959.1**  **GATAAAC---TTTATTAT-ATATATTATAATATATATTATATAAAGTTA---GTATTATATAAATATATT** 723

**Hoya exilis MW719054.1**  **AATTAATATATATAAAATATTTTAAATAAATAAATAAGATATATAAATATAAAAAAAAGATATATAAAAT** 767

**Hoya griffithii NC_069565.1**  **-------TACAGTATTACAGTATTATATAAACTTTATTATATATATATTATAAAGTTAGTATT-----AT** 641

**Hoya kerrii NC_069570.1**  **---------ATATAT----AAATAAAGTATTATATAAACTTTAT-TATATATATATTATAAA--GTTAGT** 605

**Hoya lacunosa NC_069564.1**  **----------------------------------------------------------------------** 543

**Hoya lanceolata NC_067960.1**  **----------------------------------------------------------TATATATATTAT** 605

**Hoya liangii OL826865.1**  **-------TACAGTATTACAGTATTATATAAACTTTATTATATATATATTATAAAGTTAGTATT-----AT** 642

**Hoya lithophytica MW719058.1**  **------TATATAAATTTATTATAAATAA-ATAAATTAAATATATAAATATAAATAAAT--------ATAA** 673

**Hoya lockii OR475243.1**  **----TACATATATATATATATATATATATATATAATTAATTATATAGTATTATTATTATATATATATTAT** 728

**Hoya longifolia NC_069560.1**  **-------TACAGTATTACAGTATTATATAAACTTTATTATATATATATTATAAAGTTAGTATT-----AT** 643

**Hoya lyi MW719055.1**  **TATATTTATATATAT----AAATACAGTATTATATAAACTTTAT-TATATATATATTATAAA--GTTAGT** 710

**Hoya megalaster MW719063.1**  **AATAAATAAATAAAATAT-ATAAATATAAATAAATATAAAAAAAAGATATATATAATATATAAATATAAA** 740

**Hoya meliflua NC_069571.1**  **----------TATAT----AAATAAAATATTATATAAACTTTAT-TATATATATATTATAAA--GTTAGT** 611

**Hoya monetteae MW719053.1**  **ATTATATAAATATATTATAAAATATATTATAAAATATATTATATATATAAATATAATATAAATTATAAAT** 699

**Hoya omlorii MW719060.1**  **----------------------AATTAA-ATAAAA-ATATATATAAATATAAATAAAT--------ATAA** 621

**Hoya ovalifolia NC_069563.1**  **----------------------------------------------------------------------** 548

**Hoya pandurata NC_069562.1**  **----------------------------------------------------------------------** 532

**Hoya pottsii OL754664.1**  **----------------------------------------------------------------------** 566

**Hoya pubicalyx NC_069561.1**  **ATATATTATAAAGTTAGTATTATATAAATTATATAAATATATTA--------------------------** 699

**Hoya radicalis NC_067961.1**  **----------------------------------------------------------------------** 565

**Hoya rigida NC_067962.1**  **--------ATATTATATTTAGTATTATAAAAATATATTATATATATATATATATATTATAGT-------T** 663

**Hoya silvatica NC_067963.1**  **----------------------------------------------------------------------** 566

**Hoya thomsonii NC_067612.1**  **-------TATAGTATTATA-TAAACTTTATTATATATTATATATATATTATAAAGTTAGTATT-----AT** 633

**Hoya verticillata NC_085236.1**  **----------------------------------------------------------------------** 544

**Papuahoya urniflora MW719062.1** **------TATATATATTATATAAAGTTAGTATTATATTATTATATAAATATATATAATTAGTATTTTATAA** 682

**Stephanotis volubilis OP133576** **-----------------------ATAGTAATATTAGTATTATAGAAGCTT--------------TATTAT** 572

**920 930 940 950 960 970 980**

**....|....|....|....|....|....|....|....|....|....|....|....|....|....|**

**Dischidia australis NC_067885.** **ATAT------------------------------------------------------------------** 652

**Gymnema yunnanense NC_079598.1** **ATAAA-----------------------------------------------------------GTTAGT** 622

**Hoya ariadna NC_069568.1**  **ATAT------------------------------------------------------------------** 624

**Hoya commutata NC_067958.1**  **--------------------------------------------------------------ATATTATT** 587

**Hoya dimorpha NC_067959.1**  **TTAAA--------------------------------------------------------TAAATAAAT** 737

**Hoya exilis MW719054.1**  **ATATAAATATATAATTAATATATATAAAATATTTTAAATAAATAAATAAGATATATAAATATAAAAAAAA** 837

**Hoya griffithii NC_069565.1**  **ATAAA--------------------------------------------------------TATATTATA** 655

**Hoya kerrii NC_069570.1**  **ATTAT--------------------------------------------------------ATAAATATA** 619

**Hoya lacunosa NC_069564.1**  **----------------------------------------------------------------------** 543

**Hoya lanceolata NC_067960.1**  **ATA-------------------------------------------------------------------** 608

**Hoya liangii OL826865.1**  **ATAAA--------------------------------------------------------TATATTATA** 656

**Hoya lithophytica MW719058.1**  **ATATA--------------------------------------------------------TAAAAAA--** 685

**Hoya lockii OR475243.1**  **ATATT--------------------------------------------------------ATAAAAAAA** 742

**Hoya longifolia NC_069560.1**  **ATAAA--------------------------------------------------------TATATTATA** 657

**Hoya lyi MW719055.1**  **ATTAT--------------------------------------------------------ATAAATATA** 724

**Hoya megalaster MW719063.1**  **ATATA--------------------------------------------------------TAAAAAAAA** 754

**Hoya meliflua NC_069571.1**  **ATTAT--------------------------------------------------------ATAAATATA** 625

**Hoya monetteae MW719053.1**  **ATAAT--------------------------------------------------------ATAAATATA** 713

**Hoya omlorii MW719060.1**  **ATAT------------------------------------------------------------------** 625

**Hoya ovalifolia NC_069563.1**  **----------------------------------------------------------------------** 548

**Hoya pandurata NC_069562.1**  **---AA--------------------------------------------------------TATAAAATA** 543

**Hoya pottsii OL754664.1**  **----------------------------------------------------------------------** 566

**Hoya pubicalyx NC_069561.1**  **--TAA--------------------------------------------------------AATATATTA** 711

**Hoya radicalis NC_067961.1**  **----------------------------------------------------------------------** 565

**Hoya rigida NC_067962.1**  **AGTAT--------------------------------------------------------TATATAAAT** 677

**Hoya silvatica NC_067963.1**  **----------------------------------------------------------------------** 566

**Hoya thomsonii NC_067612.1**  **ATAAA--------------------------------------------------------TATATTATA** 647

**Hoya verticillata NC_085236.1**  **----------------------------------------------------------------------** 544

**Papuahoya urniflora MW719062.1** **ATATA--------------------------------------------------------TTATAAATA** 696

**Stephanotis volubilis OP133576** **ATAAA-----------------------------------------------------------GTTAGT** 583

**990 1000 1010 1020 1030 1040 1050**

**....|....|....|....|....|....|....|....|....|....|....|....|....|....|**

**Dischidia australis NC_067885.** **--------AAATATATAAA---------------------------------------------------** 663

**Gymnema yunnanense NC_079598.1** **ATTATATAAAT-ATATAAATATA-----------------------------------------------** 644

**Hoya ariadna NC_069568.1**  **-----ATAAAATATATAAA---------------------------------------------------** 638

**Hoya commutata NC_067958.1**  **ATTATATAAATAT-ATAAATATA-----------------------------------------------** 609

**Hoya dimorpha NC_067959.1**  **AAAATATATAA-ATATAAATA-------------------------------------------------** 757

**Hoya exilis MW719054.1**  **GATATATAAAATATATAAATATATAATTAATATATATAAAATATTTTAAATAAATAAATAAGATATATAA** 907

**Hoya griffithii NC_069565.1**  **AATATAAAAATATAATAAATATA-----------------------------------------------** 678

**Hoya kerrii NC_069570.1**  **TTATAAAATATATTATAAATATA-----------------------------------------------** 642

**Hoya lacunosa NC_069564.1**  **---------ATATTATATATATA-----------------------------------------------** 557

**Hoya lanceolata NC_067960.1**  **----------------------------------------------------------------------** 608

**Hoya liangii OL826865.1**  **AATATAAAAATATAATAAATATA-----------------------------------------------** 679

**Hoya lithophytica MW719058.1**  **GATATATAAAATATATAAA---------------------------------------------------** 704

**Hoya lockii OR475243.1**  **TATATATATATTATAAAAAAATA-----------------------------------------------** 765

**Hoya longifolia NC_069560.1**  **AATATAAAAATATAATAAATATA-----------------------------------------------** 680

**Hoya lyi MW719055.1**  **TTATAAAATATATTATAAATATA-----------------------------------------------** 747

**Hoya megalaster MW719063.1**  **GATATATATAATATATAAATATA-----------------------------------------------** 777

**Hoya meliflua NC_069571.1**  **TTATAAAATATATTATAAATATA-----------------------------------------------** 648

**Hoya monetteae MW719053.1**  **AAAATATAAATAT-ATAAATATA-----------------------------------------------** 735

**Hoya omlorii MW719060.1**  **-----ATAAAATATATAAA---------------------------------------------------** 639

**Hoya ovalifolia NC_069563.1**  **-----ATATATAT-ATAAATATA-----------------------------------------------** 565

**Hoya pandurata NC_069562.1**  **AATATATAAATATAA-------------------------------------------------------** 558

**Hoya pottsii OL754664.1**  **--------------ATAAATATA-----------------------------------------------** 575

**Hoya pubicalyx NC_069561.1**  **TAAATATATATAT-ATAAATATA-----------------------------------------------** 733

**Hoya radicalis NC_067961.1**  **-AAATATATAA-----------------------------------------------------------** 575

**Hoya rigida NC_067962.1**  **ATATTATAAATAT-ATAAATATA-----------------------------------------------** 699

**Hoya silvatica NC_067963.1**  **--------------ATAAATATA-----------------------------------------------** 575

**Hoya thomsonii NC_067612.1**  **AATATATAAATATAA-------------------------------------------------------** 662

**Hoya verticillata NC_085236.1**  **-----ATATATAT-ATATATATA-----------------------------------------------** 561

**Papuahoya urniflora MW719062.1** **AATATACAAAATATATAAATATA-----------------------------------------------** 719

**Stephanotis volubilis OP133576** **ATTATATATAT-ATTTAAATATA-----------------------------------------------** 605

**1060 1070 1080 1090 1100 1110 1120**

**....|....|....|....|....|....|....|....|....|....|....|....|....|....|**

**Dischidia australis NC_067885.** **----------------------------------------------------------------------** 663

**Gymnema yunnanense NC_079598.1** **----------------------------------------------------------------------** 644

**Hoya ariadna NC_069568.1**  **----------------------------------------------------------------------** 638

**Hoya commutata NC_067958.1**  **----------------------------------------------------------------------** 609

**Hoya dimorpha NC_067959.1**  **----------------------------------------------------------------------** 757

**Hoya exilis MW719054.1**  **ATATAAAAAAAAGATATATAAAATATATAAATATATAATTAATATATATAAAATATTTTAAATAAATAAA** 977

**Hoya griffithii NC_069565.1**  **----------------------------------------------------------------------** 678

**Hoya kerrii NC_069570.1**  **----------------------------------------------------------------------** 642

**Hoya lacunosa NC_069564.1**  **----------------------------------------------------------------------** 557

**Hoya lanceolata NC_067960.1**  **----------------------------------------------------------------------** 608

**Hoya liangii OL826865.1**  **----------------------------------------------------------------------** 679

**Hoya lithophytica MW719058.1**  **----------------------------------------------------------------------** 704

**Hoya lockii OR475243.1**  **----------------------------------------------------------------------** 765

**Hoya longifolia NC_069560.1**  **----------------------------------------------------------------------** 680

**Hoya lyi MW719055.1**  **----------------------------------------------------------------------** 747

**Hoya megalaster MW719063.1**  **----------------------------------------------------------------------** 777

**Hoya meliflua NC_069571.1**  **----------------------------------------------------------------------** 648

**Hoya monetteae MW719053.1**  **----------------------------------------------------------------------** 735

**Hoya omlorii MW719060.1**  **----------------------------------------------------------------------** 639

**Hoya ovalifolia NC_069563.1**  **----------------------------------------------------------------------** 565

**Hoya pandurata NC_069562.1**  **----------------------------------------------------------------------** 558

**Hoya pottsii OL754664.1**  **----------------------------------------------------------------------** 575

**Hoya pubicalyx NC_069561.1**  **----------------------------------------------------------------------** 733

**Hoya radicalis NC_067961.1**  **----------------------------------------------------------------------** 575

**Hoya rigida NC_067962.1**  **----------------------------------------------------------------------** 699

**Hoya silvatica NC_067963.1**  **----------------------------------------------------------------------** 575

**Hoya thomsonii NC_067612.1**  **----------------------------------------------------------------------** 662

**Hoya verticillata NC_085236.1**  **----------------------------------------------------------------------** 561

**Papuahoya urniflora MW719062.1** **----------------------------------------------------------------------** 719

**Stephanotis volubilis OP133576** **----------------------------------------------------------------------** 605

**1130 1140 1150 1160 1170 1180 1190**

**....|....|....|....|....|....|....|....|....|....|....|....|....|....|**

**Dischidia australis NC_067885.** **-------------------AA-------------------------------------------------** 665

**Gymnema yunnanense NC_079598.1** **----------------------------------------------------------TAAAAAAATAAA** 656

**Hoya ariadna NC_069568.1**  **-------------------AA-------------------------------------------------** 640

**Hoya commutata NC_067958.1**  **-------------------AAA----------------------------------------TATATAAA** 620

**Hoya dimorpha NC_067959.1**  **--------------------AATATA-AAAAAAAAGATATATAT-AATATATAAATATAAAATATATAAA** 805

**Hoya exilis MW719054.1**  **TAAGATATATAAATATAAAAAAAAGATATATAAAATATATAAATATATAATTAATATATAAATATATAAA** 1047

**Hoya griffithii NC_069565.1**  **-------------------AATATAT------AAATATAAAA-------TATATAAAAAAAATATATAAA** 716

**Hoya kerrii NC_069570.1**  **-------------------AA-----------ATATATAAAA--------ATAAATATAAAATATATAAA** 674

**Hoya lacunosa NC_069564.1**  **-------------------AA-----------------------------------------TATATAAA** 567

**Hoya lanceolata NC_067960.1**  **--------------------------------------------------------------------AA** 610

**Hoya liangii OL826865.1**  **-------------------AATATAT------AAA-----------------------------------** 689

**Hoya lithophytica MW719058.1**  **-------------------AATAAATATAAATATATAAAAAAGATATATAAAATATATAAAAAA---GGG** 752

**Hoya lockii OR475243.1**  **-------------------AAAAAAA-------AAGATATATAA-AAAATATAAATAAAATATATA-AAA** 807

**Hoya longifolia NC_069560.1**  **-------------------AATATAT------AAATATAAAA-------TAT-------------ATAAA** 705

**Hoya lyi MW719055.1**  **-------------------AATATAT------AAATATAAAATA-AATATATAAATATAAAATATATAAA** 791

**Hoya megalaster MW719063.1**  **-------------------AAATATATAAAAAAAAGATATATAT-AATATATAAATATAAAATATATAAA** 827

**Hoya meliflua NC_069571.1**  **-------------------CA----A------ATATATATAA----ATATATAAATATAAAATATATAAA** 685

**Hoya monetteae MW719053.1**  **-------------------AA-----------ATATATAAAA----ATATATAAAAAAGGGATATATAAA** 771

**Hoya omlorii MW719060.1**  **-------------------AA-------------------------------------------------** 641

**Hoya ovalifolia NC_069563.1**  **-------------------TAA--------------------------------------AATATATAAA** 578

**Hoya pandurata NC_069562.1**  **-------------------AATATAT------AAA-----------------------------------** 568

**Hoya pottsii OL754664.1**  **-------------------AAA----------------------------------------TATATAAA** 586

**Hoya pubicalyx NC_069561.1**  **-------------------AAATA----------------------------TATAAAAAAATATATAAA** 756

**Hoya radicalis NC_067961.1**  **------------------------------------------AT-AATATATAAATATAAAATATATAAA** 602

**Hoya rigida NC_067962.1**  **-------------------AAA-----------------------------TATATAAAAAATATATAAA** 721

**Hoya silvatica NC_067963.1**  **-------------------AAA----------------------------------------TATATAAA** 586

**Hoya thomsonii NC_067612.1**  **-------------------AATATAT------AAA-----------------------------------** 672

**Hoya verticillata NC_085236.1**  **-------------------TAT--------------------------------------AATATATAAA** 574

**Papuahoya urniflora MW719062.1** **-------------------AATAAATATAAATATATATAAAATATAAAAAAGATATATAAAATATATAAA** 770

**Stephanotis volubilis OP133576** **----------------------------------------------------------TAAAAAAATAAA** 617

**1200 1210 1220 1230 1240 1250 1260**

**....|....|....|....|....|....|....|....|....|....|....|....|....|....|**

**Dischidia australis NC_067885.** **-AAGG-------------------------------------------GAA------TAGGCAATATTCA** 685

**Gymnema yunnanense NC_079598.1** **AAAGT-------------------------------------------GAA------TAGGCAATATTCA** 677

**Hoya ariadna NC_069568.1**  **--AGG-------------------------------------------GAA------TAGGCAATATTCA** 659

**Hoya commutata NC_067958.1**  **AAAGG-------------------------------------------GAA------TAGGCAATATTCA** 641

**Hoya dimorpha NC_067959.1**  **AAAGG-------------------------------------------GAA------TAGGCAATATTCA** 826

**Hoya exilis MW719054.1**  **AAAGATATATAAAATATATAAATATATAATTAATATATAAATATATAAAAAAGGGAATAGGCAATATTCA** 1117

**Hoya griffithii NC_069565.1**  **AAAGG-------------------------------------------GAA------TAGGCAATATTCA** 737

**Hoya kerrii NC_069570.1**  **AAAGG-------------------------------------------GAA------TAGGCAATATTCA** 695

**Hoya lacunosa NC_069564.1**  **AAAGG-------------------------------------------GAA------TAGGCAATATTCA** 588

**Hoya lanceolata NC_067960.1**  **AAAGG-------------------------------------------GAA------TAGGCAATATTCA** 631

**Hoya liangii OL826865.1**  **AAAGG-------------------------------------------GAA------TAGGCAATATTCA** 710

**Hoya lithophytica MW719058.1**  **AAAGG-------------------------------------------GAA------TAGGCAATATTCA** 773

**Hoya lockii OR475243.1**  **AAAGG-------------------------------------------GAA------TAGGCAATATTCA** 828

**Hoya longifolia NC_069560.1**  **AAAGG-------------------------------------------GAA------TAGGCAATATTCA** 726

**Hoya lyi MW719055.1**  **AAAGG-------------------------------------------GAAAGGGAATAGGCAATATTCA** 818

**Hoya megalaster MW719063.1**  **AAAGG-------------------------------------------GAA------TAGGCAATATTCA** 848

**Hoya meliflua NC_069571.1**  **AAAGG-------------------------------------------GAA------TAGGCAATATTCA** 706

**Hoya monetteae MW719053.1**  **AAAGG-------------------------------------------GAA------TAGGCAATATTCA** 792

**Hoya omlorii MW719060.1**  **--AGG-------------------------------------------GAA------TAGGCAATATTCA** 660

**Hoya ovalifolia NC_069563.1**  **AAAGG-------------------------------------------GAA------TAGGCAATATTCA** 599

**Hoya pandurata NC_069562.1**  **AAAGG-------------------------------------------GAA------TAGGCAATATTCA** 589

**Hoya pottsii OL754664.1**  **AAAGG-------------------------------------------GAA------TAGGCAATATTCA** 607

**Hoya pubicalyx NC_069561.1**  **AAAGG-------------------------------------------GAA------TAGGCAATATTCA** 777

**Hoya radicalis NC_067961.1**  **AAA------------------------------------------------------------ATAT---** 609

**Hoya rigida NC_067962.1**  **AAAGG-------------------------------------------GAA------TAGGCAATATTCA** 742

**Hoya silvatica NC_067963.1**  **AAAGG-------------------------------------------GAA------TAGGCAATATTCA** 607

**Hoya thomsonii NC_067612.1**  **AAAGG-------------------------------------------GAA------TAGGCAATATTCA** 693

**Hoya verticillata NC_085236.1**  **AAAGG-------------------------------------------GAA------TAGGCAATATTCA** 595

**Papuahoya urniflora MW719062.1** **AAAGG-------------------------------------------GAA------TAGGCAATATTCA** 791

**Stephanotis volubilis OP133576** **AAAGT-------------------------------------------GAA------TAGGCAATATTCA** 638

**1270 1280 1290 1300 1310 1320 1330**

**....|....|....|....|....|....|....|....|....|....|....|....|....|....|**

**Dischidia australis NC_067885.** **AGCACGAACAAAAAAAGAAGAACGACTATTTCCATCCGCGAAAAC-GAAAAAAAAA-GAGGGTAAATACG** 753

**Gymnema yunnanense NC_079598.1** **AGCACGAACAAAAAAAGAAGAAAGACTATTTCCATCCGCGAAAACGAAAAAAA-G--GAGGGTAAATACT** 744

**Hoya ariadna NC_069568.1**  **AGCACGAACAAAAAAAGAAGAAGGACTATTTCCATCCGCGAAAAC-AAAAAAAA-G-GAGGGTAAATACG** 726

**Hoya commutata NC_067958.1**  **AGCACGAACAAAAAAAGAAGAAGGACTATTTCCATCCGCGAAAACGAAAAAAAAA--GAGGGTAAATACG** 709

**Hoya dimorpha NC_067959.1**  **AGCACGAACAAAAAAAGAAGAAGGACTATTTCCATCCGCGAAAACGAAAAAAAAG--GAGGGTAAATACG** 894

**Hoya exilis MW719054.1**  **AGCACGAACAAAAAAAGAAGAAGGACTATTTCCATCCGCGAAAACAAAAAAAAAG--GAGGGTAAATACG** 1185

**Hoya griffithii NC_069565.1**  **AGCACGAACAAAAAAAGAAGAAGGACTATTTCCATCCGCGAAAACGAAAAAAAAA--GAGGGTAAATACG** 805

**Hoya kerrii NC_069570.1**  **AGCACGAACAAAAAAAGAAGAAGGACTGTTTCCATCCGCGAAAACGAAAAAAAAAAAGAGGGTAAATACG** 765

**Hoya lacunosa NC_069564.1**  **AGCACGAACAAAAAAAGAAGAAGGACTATTTCCATCCGCGAAAACGAAAAAAAAA--AAGGGTAAATACG** 656

**Hoya lanceolata NC_067960.1**  **AGCACGAACAAAAAAAGAAGAAGGACTATTTCCATCCGCGAAAACGAAAAAAAAAG-GAGGGTAAATACG** 700

**Hoya liangii OL826865.1**  **AGCACGAACAAAAAAAGAAGAAGGACTATTTCCATCCGCGAAAACGAAAAAAAAA--GAGGGTAAATACG** 778

**Hoya lithophytica MW719058.1**  **AGCACGAACAAAAAAAGAAGAAGGACTATTTCCATCCGCGAAAAC-AAAAAAAAAG-GAGGGTAAATACG** 841

**Hoya lockii OR475243.1**  **AGCACGAACAAAAAAAGAAGAAGGACTATTTCCATCCGCGAAAAC-AAAAATAAAG-GAG--------CG** 888

**Hoya longifolia NC_069560.1**  **AGCACGAACAAAAAAAGAAGAAGGACTATTTCCATCCGCGAAAACGAAAAAAAAA--GAGGGTAAATACG** 794

**Hoya lyi MW719055.1**  **AGCACGAACAAAAAAAGAAGAAGGACTATTTCCATCCGCGAAAACGAAAAAAAAAA-GAGGGTAAATACG** 887

**Hoya megalaster MW719063.1**  **AGCACGAACAAAAAAAGAAGAAGGACTATTTCCATCCGCGAAAACGAAAAAAAAG--GAGGGTAAATACG** 916

**Hoya meliflua NC_069571.1**  **AGCACGAACAAAAAAAGAAGAAGGACTATTTCCATCCGCGAAAACGAAAAAAA----GAGGGTAAATACG** 772

**Hoya monetteae MW719053.1**  **AGCACGAACAAAAAAAGAAGAAGGACTATTTCCATCCGCGAAAACGAAAAAAAAA--GAGGGTAAATACG** 860

**Hoya omlorii MW719060.1**  **AGCACGAACAAAAAAAGAAGAAGGACTATTTCCATCCGCGAAAAC-AAAAAAAAAG-GAGGGTAAATACG** 728

**Hoya ovalifolia NC_069563.1**  **AGCACGAACAAAAAAAGAAGAAGGACTATTTCCATCCGCGAAAACGAAAAAAAAAAAGAGGGTAAATACG** 669

**Hoya pandurata NC_069562.1**  **AGCACGAACAAAAAAAGAAGAAGGACTATTTCCATCCGCGAAAACGAAAAAAAAA--GAGGGTAAATACG** 657

**Hoya pottsii OL754664.1**  **AGCACGAACAAAAAAAGAAGAAGGACTATTTCCATCCGCGAAAACGAAAAAAAAAA-GAGGGTAAATACG** 676

**Hoya pubicalyx NC_069561.1**  **AGCACGAACAAAAAAAGAAGAAGGACTATTTCCATCCGCGAAAACGAAAAAAAAAA-GAGGGTAAATACG** 846

**Hoya radicalis NC_067961.1**  **-------ATAAAAAAGGGAAAACGA--------------------AAAAAAAAAA--GAGGGTAAATACG** 650

**Hoya rigida NC_067962.1**  **AGCACGAACAAAAAAAGAAGAAGGACTATTTCCATCCGCGAAAACGAAAAAAAAA--GAGGGTAAATACG** 810

**Hoya silvatica NC_067963.1**  **AGCACGAACAAAAAAAGAAGAAGGACTATTTCCATCCGCGAAAACGAAAAAAAAAA-GAGGGTAAATACG** 676

**Hoya thomsonii NC_067612.1**  **AGCACGAACAAAAAAAGAAGAAGGACTATTTCCATCCGCGAAAACGAAAAAAAA---GAGGGTAAATACG** 760

**Hoya verticillata NC_085236.1**  **AGCACGAACAAAAAAAGAAGAAGGACTATTTCCATCCGCGAAAACGAAAAAAAAA--GAGGGTAAATACG** 663

**Papuahoya urniflora MW719062.1** **AGCACGAACAAAAAAAGAAGAAGGACTATTTCCATCCGCGAAAACGAAAAAAAAAG-GAGGGTAAATACG** 860

**Stephanotis volubilis OP133576** **AGCACGAACAAAAAAAGAAGAAAGACTATTTCCATCCGCGAAAACGAAAAAAAAG--GAGGGTAAATACT** 706

**1340 1350 1360 1370 1380 1390 1400**

**....|....|....|....|....|....|....|....|....|....|....|....|....|....|**

**Dischidia australis NC_067885.** **CTAAATTTTTTGATTCTTTGATGATCCCATCTTATGATACCC-AT------TTTCCGGTTCGACAAAAGG** 816

**Gymnema yunnanense NC_079598.1** **CTAAATTTTTTGATTCTTTGATGATCCCATCTTATTATACCCCATTTTCCTTTTCCGGTTCGACAAAAGG** 814

**Hoya ariadna NC_069568.1**  **CTAAATTTTTTGATTCTTTGATGATCCCATCTTATTATACCC-AT------TTTCCGGTTCGACAAAAGG** 789

**Hoya commutata NC_067958.1**  **CTAAATTTTAGGATTCTTTGATGATCCCATCTTATTATACCC-AT------TTTCCGGTTCGACAAAAGG** 772

**Hoya dimorpha NC_067959.1**  **CTAAATTTTTTGATTCCTTGATGATCCCATCTTATTATACCC-AT------TTTCCGGTTCGACAAAAGG** 957

**Hoya exilis MW719054.1**  **CTAAATTTTTGGATTCTTTGATGATCCCATCTTATTATACCC-AT------TTTCCGGTTCGACAAAAGG** 1248

**Hoya griffithii NC_069565.1**  **CTAAATTTTTGGATTCTTTGATGATCCCATCTTATTATACCC-AT------TTTCCGGTTCGACAAAAGG** 868

**Hoya kerrii NC_069570.1**  **CTAAATTTTTGGATTCTTTGATGATCCCATCTTATTATACCC-AT------TTTCCGGTTCGACAAAAGG** 828

**Hoya lacunosa NC_069564.1**  **CTAAATTTTTGGATTCTTTGATGATCCCATCTTATTATACCC-AT------TTTCCGGTTCGACAAAAGG** 719

**Hoya lanceolata NC_067960.1**  **CTAAATTTTTGGATTCTTTGATGATCCCATCTTATTATACCC-AT------TTTCCGGTTCGACAAAAGG** 763

**Hoya liangii OL826865.1**  **CTAAATTTTTGGATTCTTTGATGATCCCATCTTATTATACCC-AT------TTTCCGGTTCGACAAAAGG** 841

**Hoya lithophytica MW719058.1**  **CTAAATTTTTTGATTCTTTGATGATCCCATCTTATTATACCC-AT------TTTCCGGTTCGACAAAAGG** 904

**Hoya lockii OR475243.1**  **CTAAATTTTTGGATTCTTTGATGATCCCATCTTATTATACCC-AT------TTTCCGGTTCGACAAAAGG** 951

**Hoya longifolia NC_069560.1**  **CTAAATTTTTGGATTCTTTGATGATCCCATCTTATTATACCC-AT------TTTCCGGTTCGACAAAAGG** 857

**Hoya lyi MW719055.1**  **CTAAATTTTTGGATTCTTTGATGATCCCATCTTATTATACCC-AT------TTTCCGGTTCGACAAAAGG** 950

**Hoya megalaster MW719063.1**  **CTAAATTTTTGGATTCCTTGATGATCCCATCTTATTATACCC-AT------TTTCCGGTTCGACAAAAGG** 979

**Hoya meliflua NC_069571.1**  **CTAAATTTTTGGATTCTTTGATGATCCCATCTTATTATACCC-AT------TTTCCGGTTCGACAAAAGG** 835

**Hoya monetteae MW719053.1**  **CTAAATTTTTGGATTCTTTGATGATCCCATCTTATTATACCC-AT------TTTCCGGTTCGACAAAAGG** 923

**Hoya omlorii MW719060.1**  **CTAAATTTTTTGATTCTTTGATGATCCCATCTTATTATACCC-AT------TTTCCGGTTCGACAAAAGG** 791

**Hoya ovalifolia NC_069563.1**  **CTAAATTTTTGGATTCTTTGATGATCCCATCTTATTATACCC-AT------TTTCCGGTTCGACAAAAGG** 732

**Hoya pandurata NC_069562.1**  **CTAAATTTTTGGATTCTTTGATGATCCCATCTTATTATACCC-AT------TTTCCGGTTCGACAAAAGG** 720

**Hoya pottsii OL754664.1**  **CTAAATTTTTGGATTCTTTGATGATCCCATCTTATTATACCC-AT------TCTCCGGTTCGACAAAAGG** 739

**Hoya pubicalyx NC_069561.1**  **CTAAATTTTTGGATTCTTTGATGATCCCATCTTATTATACCC-AT------TTTCCGGTTCGACAAAAGG** 909

**Hoya radicalis NC_067961.1**  **CTAAATTTTTTGATTCTTTGATGATCCCATCTTATTATACCC-AT------TTTCCGGTTCGACAAAAGG** 713

**Hoya rigida NC_067962.1**  **CTAAATTTTTGGATTCTTTGATGATCCCATCTTATTATACCC-AT------TTTCCGGTTCGACAAAAGG** 873

**Hoya silvatica NC_067963.1**  **CTAAATTTTTGGATTCTTTGATGATCCCATCTTATTATACCC-AT------TCTCCGGTTCGACAAAAGG** 739

**Hoya thomsonii NC_067612.1**  **CTAAATTTTTGGATTCTTTGATGATCCCATCTTATTATACCC-AT------TTTCCGGTTCGACAAAAGG** 823

**Hoya verticillata NC_085236.1**  **CTAAATTTTTGGATTCTTTGATGATCCCATCTTATTATACCC-AT------TTTCCGGTTCGACAAAAGG** 726

**Papuahoya urniflora MW719062.1** **CTAAATTTTTAGATTCTTTGATGATCCCATCTTATTATACCC-AT------TTTCCGGTTCGACAAAAGG** 923

**Stephanotis volubilis OP133576** **CTAAATTTTTAGATTCTTTGATGATCCCATCTTATTATACCCCAT------TTTCCGGTTCGACAAAAGG** 770

**1410 1420 1430 1440 1450 1460 1470**

**....|....|....|....|....|....|....|....|....|....|....|....|....|....|**

**Dischidia australis NC_067885.** **TCCAGTTGTATACAATAATTGAATTGTAGCGGGTATAGTTTAGTGGTAAAAGTGTGATTCGTTTTTTTAA** 886

**Gymnema yunnanense NC_079598.1** **TCCAGTTGCATACAATAATCGAATAGTAGCGGGTATAGTTTAGTGGTAAAAGTGTGATTCGTTTTTTTAA** 884

**Hoya ariadna NC_069568.1**  **TCCAGTTGTATACAATAATCGAATTGTAGCGGGTATAGTTTAGTGGTAAAAGTGTGATTCGTTTTTTTAA** 859

**Hoya commutata NC_067958.1**  **TCCAGTTGTATACAATAATCGAATTGTAGCGGGTATAGTTTAGTGGTAAAAGTGTGATTCGTTTTTTTAA** 842

**Hoya dimorpha NC_067959.1**  **TCCAGTTGTATACAATAATCGAATTGTAGCGGGTATAGTTTAGTGGTAAAAGTGTGATTCGTTTTTTTAA** 1027

**Hoya exilis MW719054.1**  **TACAGTTGTATACAATAATCGAATTGTAGCGGGTATAGTTTAGTGGTAAAAGTGTGATTCGTTTTTTTAA** 1318

**Hoya griffithii NC_069565.1**  **TCCAGTTGTATACAATAATCGAATTGTAGCGGGTATAGTTTAGTGGTAAAAGTGTGATTCGTTTTTTTAA** 938

**Hoya kerrii NC_069570.1**  **TCCAGTTGTATACAATAATCGAATTGTAGCGGGTATAGTTTAGTGGTAAAAGTGTGATTCGTTTTTTTAA** 898

**Hoya lacunosa NC_069564.1**  **TCCAGTTGTATACAATAATCGAATTGTAGCGGGTATAGTTTAGTGGTAAAAGTGTGATTCGTTTTTTTAA** 789

**Hoya lanceolata NC_067960.1**  **TCCAGTTGTATACAATAATCGAATTGTAGCGGGTATAGTTTAGTGGTAAAAGTGTGATTCGTTTTTTTAA** 833

**Hoya liangii OL826865.1**  **TCCAGTTGTATACAATAATCGAATTGTAGCGGGTATAGTTTAGTGGTAAAAGTGTGATTCGTTTTTTTAA** 911

**Hoya lithophytica MW719058.1**  **TCCAGTTGTATACAATAATCGAATTGTAGCGGGTATAGTTTAGTGGTAAAAGTGTGATTCGTTTTTTTAA** 974

**Hoya lockii OR475243.1**  **TCCAGTTGTATACAATAATCGAATTGTAGCGGGTATAGTTTAGTGGTAAAAGTGTGATTCGTTTTTTTAA** 1021

**Hoya longifolia NC_069560.1**  **TCCAGTTGTATACAATAATCGAATTGTAGCGGGTATAGTTTAGTGGTAAAAGTGTGATTCGTTTTTTTAA** 927

**Hoya lyi MW719055.1**  **TCCAGTTGTATACAATAATCGAATTGTAGCGGGTATAGTTTAGTGGTAAAAGTGTGATTCGTTTTTTTAA** 1020

**Hoya megalaster MW719063.1**  **TCCAGTTGTATACAATAATCGAATTGTAGCGGGTATAGTTTAGTGGTAAAAGTGTGATTCGTTTTTTTAA** 1049

**Hoya meliflua NC_069571.1**  **TCCAGTTGTATACAATAATCGAATTGTAGCGGGTATAGTTTAGTGGTAAAAGTGTGATTCGTTTTTTTAA** 905

**Hoya monetteae MW719053.1**  **TCCAGTTGTATACAATAATCGAATTGTAGCGGGTATAGTTTAGTGGTAAAAGTGTGATTCGTTTTTTTAA** 993

**Hoya omlorii MW719060.1**  **TCCAGTTGTATACAATAATCGAATTGTAGCGGGTATAGTTTAGTGGTAAAAGTGTGATTCGTTTTTTTAA** 861

**Hoya ovalifolia NC_069563.1**  **TCCAGTTGTATACAATAATCGAATTGTAGCGGGTATAGTTTAGTGGTAAAAGTGTGATTCGTTTTTTTAA** 802

**Hoya pandurata NC_069562.1**  **TCCAGTTGTATACAATAATCGAATTGTAGCGGGTATAGTTTAGTGGTAAAAGTGTGATTCGTTTTTTTAA** 790

**Hoya pottsii OL754664.1**  **TCCAGTTGTATACAATAATCGAATTGTAGCGGGTATAGTTTAGTGGTAAAAGTGTGATTCGTTTTTTTAA** 809

**Hoya pubicalyx NC_069561.1**  **TCCAGTTGTATACAATAATCGAATTGTAGCGGGTATAGTTTAGTGGTAAAAGTGTGATTCGTTTTTTTAA** 979

**Hoya radicalis NC_067961.1**  **TCCAGTTGTATACAATAATCGAATTGTAGCGGGTATAGTTTAGTGGTAAAAGTGTGATTCGTTTTTTTAA** 783

**Hoya rigida NC_067962.1**  **TCCAGTTGTATACAATAATCGAATTGTAGCGGGTATAGTTTAGTGGTAAAAGTGTGATTCGTTTTTTTAA** 943

**Hoya silvatica NC_067963.1**  **TCCAGTTGTATACAATAATCGAATTGTAGCGGGTATAGTTTAGTGGTAAAAGTGTGATTCGTTTTTTTAA** 809

**Hoya thomsonii NC_067612.1**  **TCCAGTTGTATACAATAATCGAATTGTAGCGGGTATAGTTTAGTGGTAAAAGTGTGATTCGTTTTTTTAA** 893

**Hoya verticillata NC_085236.1**  **TCCAGTTGTATACAATAATCGAATTGTAGCGGGTATAGTTTAGTGGTAAAAGTGTGATTCGTTTTTTTAA** 796

**Papuahoya urniflora MW719062.1** **TCCAGTTGTATACAATAATCGAATTGTAGCGGGTATAGTTTAGTGGTAAAAGTGTGATTCGTTTTTTTAA** 993

**Stephanotis volubilis OP133576** **TTCAGTTGCATACAATAATCGAATTGTAGCGGGTATAGTTTAGTGGTAAAAGTGTGATTCGTTTTTTTAA** 840

**1480 1490 1500 1510 1520 1530 1540**

**....|....|....|....|....|....|....|....|....|....|....|....|....|....|**

**Dischidia australis NC_067885.** **CCCTTTGATAGTTAAAGGGTCTTTGTTTTCGGTTTGATTCGTATTCCGACCAAAAACTTTATTTGAAAAA** 956

**Gymnema yunnanense NC_079598.1** **CCCTTTGATAGTTAAAGGGTCTTTGTTTTCGGTTTGATTCGTATTCCGACCAAAAACTTTATTTGAAAAA** 954

**Hoya ariadna NC_069568.1**  **CCCTTTGATAGTTAAAGGGTCTTTGTTTTCGGTTTGATTCGTATTCCGACCAAAAACTTTATTTGAAAAA** 929

**Hoya commutata NC_067958.1**  **CCCTTTGATAGTTAAAGGGTCTTTGTTTTCGGTTTGATTCGTATTCCGACCAAAAACTTTATTTGAAAAA** 912

**Hoya dimorpha NC_067959.1**  **CCCTTTGGTAGTTAAAGGGTCTTTGTTTTCGGTTTGATTCCTATTCCGACCAAAAACTTTATTTGAAAAA** 1097

**Hoya exilis MW719054.1**  **CCCTTTGATAGTTAAAGGGTCTTTGTTTTCGGTTTGATTCGTATTCCGACAAAAAACTTTATTTGAAAAA** 1388

**Hoya griffithii NC_069565.1**  **CCCTTTGATAGTTAAAGGGTCTTTGTTTTCGGTTTGATTCGTATTCCGACCAAAAACTTTATTTGAAAAA** 1008

**Hoya kerrii NC_069570.1**  **CCCTTTGATAGTTAAAGGGTCTTTGTTTTCGGTTTGATTCGTATTCCGACCAAAAACTTTATTTGAAAAA** 968

**Hoya lacunosa NC_069564.1**  **CCCTTTGATAGTTAAAGGGTCTTTGTTTTCGGTTTGATTCGTATTCCGACCAAAAACTTTATTTCAAAAA** 859

**Hoya lanceolata NC_067960.1**  **CCCTTTGATAGTTAAAGGGTCTTTGTTTTCGGTTTGATTCGTATTCCGACCAAAAACTTTATTTGAAAAA** 903

**Hoya liangii OL826865.1**  **CCCTTTGATAGTTAAAGGGTCTTTGTTTTCGGTTTGATTCGTATTCCGACCAAAAACTTTATTTGAAAAA** 981

**Hoya lithophytica MW719058.1**  **CCCTTTGATAGTTAAAGGGTCTTTGTTTTCGGTTTGATTCGTATTCCGACCAAAAACTTTATTTGAAAAA** 1044

**Hoya lockii OR475243.1**  **CCCTTTGATAGTTAAAGGGTCTTTGTTTTCGGTTTGATTCGTATTCCGACAAAAAACTTTATTTGAAAAA** 1091

**Hoya longifolia NC_069560.1**  **CCCTTTGATAGTTAAAGGGTCTTTGTTTTCGGTTTGATTCGTATTCCGACCAAAAACTTTATTTGAAAAA** 997

**Hoya lyi MW719055.1**  **CCCTTTGATAGTTAAAGGGTCTTTGTTTTCGGTTTGATTCGTATTCCGACCAAAAACTTTATTTGAAAAA** 1090

**Hoya megalaster MW719063.1**  **CCCTTTGGTAGTTAAAGGGTCTTTGTTTTCGGTTTGATTCCTATTCCGACCAAAAACTTTATTTGAAAAA** 1119

**Hoya meliflua NC_069571.1**  **CCCTTTGATAGTTAAAGGGTCCTTGTTTTCGGTTTGATTCGTATTCCGACCAAAAACTTTATTTGAAAAA** 975

**Hoya monetteae MW719053.1**  **CCCTTTGATAGTTAAAGGGTCTTTGTTTTCGGTTTGATTCGTATTCCGACCAAAAACTTTATTTGAAAAA** 1063

**Hoya omlorii MW719060.1**  **CCCTTTGATAGTTAAAGGGTCTTTGTTTTCGGTTTGATTCGTATTCCGACCAAAAACTTTATTTGAAAAA** 931

**Hoya ovalifolia NC_069563.1**  **CCCTTTGATAGTTAAAGGGTCTTTGTTTTCGGTTTGATTCGTATTCCGACCAAAAACTTTATTTGAAAAA** 872

**Hoya pandurata NC_069562.1**  **CCCTTTGATAGTTAAAGGGTCTTTGTTTTCGGTTTGATTCGTATTCCGACCAAAAACTTTATTTGAAAAA** 860

**Hoya pottsii OL754664.1**  **CCCTTTGATAGTTAAAGGGTCTTTGTTTTCGGTTTGATTCGTATTCCGACCAAAAACTTTATTTGAAAAA** 879

**Hoya pubicalyx NC_069561.1**  **CCCTTTGATAGTTAAAGGGTCTTTGTTTTCGGTTTGATTCGTATTCCGACCAAAAACTTTATTTGAAAAA** 1049

**Hoya radicalis NC_067961.1**  **CCCTTTGATAGTTAAAGGGTCTTTGTTTTCGGTTTGATTCGTATTCCGACCAAAAACTTTATTTGAAAAA** 853

**Hoya rigida NC_067962.1**  **CCCTTTGATAGTTAAAGGGTCTTTGTTTTCGGTTTGATTCGTATTCCGACCAAAAACTTTATTTGAAAAA** 1013

**Hoya silvatica NC_067963.1**  **CCCTTTGATAGTTAAAGGGTCTTTGTTTTCGGTTTGATTCGTATTCCGACCAAAAACTTTATTTGAAAAA** 879

**Hoya thomsonii NC_067612.1**  **CCCTTTGCTAGTTAAAGGGTCTTTGTTTTCGGTTTGATTCGTATTCCGACCAAAAACTTTATTTGAAAAA** 963

**Hoya verticillata NC_085236.1**  **CCCTTTGATAGTTAAAGGGTCTTTGTTTTCGGTTTGATTCGTATTCCGACCAAAAACTTTATTTGAAAAA** 866

**Papuahoya urniflora MW719062.1** **CCCTTTGATAGTTAAAGGGTCTTTGTTTTCGGTTTGATTCGTATTCCGACCAAAAACTTTATTTGAAAAA** 1063

**Stephanotis volubilis OP133576** **CCCTTTGATAGTTAAGGGGTCTTTGTTTTCGGTTTGATTCGTATTCCGACCAAAAACTTTATTTGAAAAA** 910

**1550 1560 1570 1580 1590 1600 1610**

**....|....|....|....|....|....|....|....|....|....|....|....|....|....|**

**Dischidia australis NC_067885.** **AGAAAAGGATTTAATCCTTTACCTCTCAATCACGGATTCGAGAAAAAA-TATACATTCTCGTGATTTGTA** 1025

**Gymnema yunnanense NC_079598.1** **AAAAAAGGATTTAATCCTTTACCTCTCAATCACGGATTCGAGAAAAAA-TATACATTCTCGTGATTTGTA** 1023

**Hoya ariadna NC_069568.1**  **AGAAAAGGATTTAATCCTTTACCTCTCAATCACGGATTCGAGAAAAAA-TATACATTCTCGTGATTTGTA** 998

**Hoya commutata NC_067958.1**  **AGAAAAGGATTAAATCCTTTACCTCTCAATCACGGATTCGAGAAAAAA-TATACATTCTCGTGATTTGTA** 981

**Hoya dimorpha NC_067959.1**  **AGAAAAGGATTTAATCCTTTACCTCTCAATCACGGATTCGAGAAAAAA-TATACATTCTCGTGATTTGTA** 1166

**Hoya exilis MW719054.1**  **AGAAAAGGATTTAATCCTTTACCTCTCAATCACGGATTCGAGAAAAAA-TATACATTCTCGTGATTTGTA** 1457

**Hoya griffithii NC_069565.1**  **AGAAAAGGATTTAATCCTTTACCTCTCAATCACGGATTCGAGAAAAAA-TATACATTCTCGTGATTTGTA** 1077

**Hoya kerrii NC_069570.1**  **AGAAAAGGATTTAATCCTTTACCTCTCAATCACGGATTCGAGAAAAAAATATACATTCTCGTGATTTGTA** 1038

**Hoya lacunosa NC_069564.1**  **AGAAAAGGATTAAATCCTTTACCTCTCAATCACGGATTCGAGAAAAAA-TATACATTCTCGTGATTTGTA** 928

**Hoya lanceolata NC_067960.1**  **AGAAAAGGATTTAATCCTTTACCTCTCAATCACGGATTCGAGAAAAAA-TATACATTCTCGTGATTTGTA** 972

**Hoya liangii OL826865.1**  **AGAAAAGGATTTAATCCTTTACCTCTCAATCACGGATTCGAGAAAAAA-TATACATTCTCGTGATTTGTA** 1050

**Hoya lithophytica MW719058.1**  **AGAAAAGGATTTAATCCTTTACCTCTCAATCACGGATTCGAGAAAAAA-TATACATTCTCGTGATTTGTA** 1113

**Hoya lockii OR475243.1**  **A-AAAAGGATTTAATCCTTTACCTCTCAATCACGGATTCGAGAAAAAA-TATACATTCTCGTGATTTGTA** 1159

**Hoya longifolia NC_069560.1**  **AGAAAAGGATTTAATCCTTTACCTCTCAATCACGGATTCGAGAAAAAA-TATACATTCTCGTGATTTGTA** 1066

**Hoya lyi MW719055.1**  **AGAAAAGGATTTAATCCTTTACCTCTCAATCACGGATTCGAGAAAAAA-TATACATTCTCGTGATTTGTA** 1159

**Hoya megalaster MW719063.1**  **AGAAAAGGATTAAATCCTTTACCTCTCAATCACGGATTCGAGAAAAAA-TATACATTCTCGTGATTTGTA** 1188

**Hoya meliflua NC_069571.1**  **AGAAAAGGATTTAATCCTTTACCTCTCAATCACGGATTCGAGAAAAAA-TATACATTCTCGTGATTTGTA** 1044

**Hoya monetteae MW719053.1**  **AAAAAAGGATTTAATCCTTTACCTCTCAATCACGGATTCGAGAAAAAA-TATACATTCTCGTGATTTGTA** 1132

**Hoya omlorii MW719060.1**  **AGAAAAGGATTTAATCCTTTACCTCTCAATCACGGATTCGAGAAAAAA-TATACATTCTCGTGATTTGTA** 1000

**Hoya ovalifolia NC_069563.1**  **AGAAAAGGATTTAATCCTTTACCTCTCAATCACGGATTCGAGAAAAAA-TATACATTCTCGTGATTTGTA** 941

**Hoya pandurata NC_069562.1**  **AGAAAAGGATTTAATCCTTTACCTCTCAATCACGGATTCGAGAAAAAA-TATAAATTCTCGTGATTTGTA** 929

**Hoya pottsii OL754664.1**  **AGAAAAGGATTTAATCCTTTACCTCTCAATCACGGATTCGAGAAAAAA-TATACATTCTCGTGATTTGTA** 948

**Hoya pubicalyx NC_069561.1**  **AGAAAAGGATTTAATCCTTTACCTCTCAATCACGGATTCGAGAAAAAA-TATACATTCTCGTGATTTGTA** 1118

**Hoya radicalis NC_067961.1**  **AGAAAAGGATTTAATCCTTTACCTCTCAATTACGGATTCGAGAAAAAA-TATACATTCTCGTGATTTGTA** 922

**Hoya rigida NC_067962.1**  **AGAAAAGGATTTAATCCTTTACCTCTCAATCACGGATTCGAGAAAAAA-TATACATTCTCGTGATTTGTA** 1082

**Hoya silvatica NC_067963.1**  **AGAAAAGGATTTAATCCTTTACCTCTCAATCACGGATTCGAGAAAAAA-TATACATTCTCGTGATTTGTA** 948

**Hoya thomsonii NC_067612.1**  **AGAAAAGGATTTAATCCTTTACCTCTCAATCACGGATTCGAGAAAAAA-TATACATTCTCGTGATTTGTA** 1032

**Hoya verticillata NC_085236.1**  **AGAAAAGGATTTAATCCTTTACCTCTCAATCACGGATTCGAGAAAAAA-TATACATTCTCGTGATTTGTA** 935

**Papuahoya urniflora MW719062.1** **AGAAAAGGATTTAATCCTTTACCTCTCAATCACGGATTCGAGAAAAAA-TATACATTCTCGTGATTTGTA** 1132

**Stephanotis volubilis OP133576** **ATAAAAGGATTTAATCCTTTACCTCTCAATCACGGATTCGAGAAAAA--TATACATTCTCGTGATTTGTA** 978

**1620 1630 1640 1650 1660 1670 1680**

**....|....|....|....|....|....|....|....|....|....|....|....|....|....|**

**Dischidia australis NC_067885.** **TCCAAGGATCACTTAGAAAGTGACAAATTGGATTATGAAATTACGAAACATAATTTTGGAATTGGATTA-** 1094

**Gymnema yunnanense NC_079598.1** **TCCAAGGATCACTTAGAAAGTGACAAATTGGATTATGAAATTACGAAACATAATTTTGGAATTGGATTA-** 1092

**Hoya ariadna NC_069568.1**  **TCCAAGGATCACTTAGAAAGTGACAAATTGGATTATGAAATTACGAAACATAATTTTGGAATTGGATTA-** 1067

**Hoya commutata NC_067958.1**  **TCCAAGGATCACTTAGAAAGTGACAAATTGGATTATGAAATTACGAAACATAATTTTGGAATTGGATTAT** 1051

**Hoya dimorpha NC_067959.1**  **TCCAAGGATCACTTAGAAAGTGACAAATTGGATTATGAAATTACGAAACATAATTTTGGAATTGGATTA-** 1235

**Hoya exilis MW719054.1**  **TCCAAGGATCACTTAGAAAGTGACAAATTGGATTATGAAATTACGAAACATAATTTTGGAATTGGATTA-** 1526

**Hoya griffithii NC_069565.1**  **TCCAAGGATCACTTAGAAAGTGACAAATTGGATTATGAAATTACGAAACATAATTTTGGAATTGGATTA-** 1146

**Hoya kerrii NC_069570.1**  **TCCAAGGATCACTTAGAAAGTGACAAATTGGATTATGAAATTACGAAACATAATTTTGGAATTGGATTA-** 1107

**Hoya lacunosa NC_069564.1**  **TCCAAGGATCACTTAGAAAGTGACAAATTGGATTATGAAATTACGAAACATAATTTTGGAATTGGATTA-** 997

**Hoya lanceolata NC_067960.1**  **TCCAAGGATCACTTAGAAAGTGACAAATTGGATTATGAAATTACGAAACATAATTTTTGAATTGGATTA-** 1041

**Hoya liangii OL826865.1**  **TCCAAGGATCACTTAGAAAGTGACAAATTGGATTATGAAATTACGAAACATAATTTTGGAATTGGATTA-** 1119

**Hoya lithophytica MW719058.1**  **TCCAAGGATCACTTAGAAAGTGACAAATTGGATTATGAAATTACGAAACATAATTTTGGAATTGGATTA-** 1182

**Hoya lockii OR475243.1**  **TCCAAGGATCACTTAGAAAGTGACAAATTGGATTATGAAATTACGAAACATAATTTTGGAATTGGATTA-** 1228

**Hoya longifolia NC_069560.1**  **TCCAAGGATCACTTAGAAAGTGACAAATTGGATTATGAAATTACGAAACATAATTTTGGAATTGGATTA-** 1135

**Hoya lyi MW719055.1**  **TCCAAGGATCACTTAGAAAGTGACAAATTGGATTATGAAATTACGAAACATAATTTTGGAATTGGATTA-** 1228

**Hoya megalaster MW719063.1**  **TCCAAGGATCACTTAGAAAGTGACAAATTGGATTATGAAATTACGAAACATAATTTTGGAATTGGATTA-** 1257

**Hoya meliflua NC_069571.1**  **TCCAAGGATCACTTAGAAAGTGACAAATTGGATTATGAAATTACGAAACATAATTTTGGAATTGGATTA-** 1113

**Hoya monetteae MW719053.1**  **TCCAAGGATCACTTAGAAAGTGACAAATTGGATTATGAAATTACGAAACATAATTTTGGAATTGGATTA-** 1201

**Hoya omlorii MW719060.1**  **TCCAAGGATCACTTATAAAGTGACAAATTGGATTATGAAATTACGAAACATAATTTTGGAATTGGATTA-** 1069

**Hoya ovalifolia NC_069563.1**  **TCCAAGGATCACTTAGAAAGTGACAAATTGGATTATGAAATTACGAAACATAATTTTGGAATTGGATTA-** 1010

**Hoya pandurata NC_069562.1**  **TCCAAGGATCACTTAGAAAGTGACAAATTGGATTATGAAATTACGAAACATAATTTTGGAATTGGATTA-** 998

**Hoya pottsii OL754664.1**  **TCCAAGGATCACTTAGAAAGTGACAAATTGGATTATGAAATTACGAAACATAATTTTGGAATTGGATTA-** 1017

**Hoya pubicalyx NC_069561.1**  **TCCAAGGATCACTTAGAAAGTGACAAATTGGATTATGAAATTACGAAACATAATTTTGGAATTGGATTA-** 1187

**Hoya radicalis NC_067961.1**  **TCCAAGGATCACTTAGAAAGTGACAAATTGGATTATGAAATTACGAAACATAATTTTGGAATTGGATTA-** 991

**Hoya rigida NC_067962.1**  **TCCAAGGATCACTTAGAAAGTGACAAATTGGATTATGAAATTACGAAACATAATTTTGGAATTGGATTA-** 1151

**Hoya silvatica NC_067963.1**  **TCCAAGGATCACTTAGAAAGTGACAAATTGGATTATGAAATTACGAAACATAATTTTGGAATTGGATTA-** 1017

**Hoya thomsonii NC_067612.1**  **TCCAAGGATCACTTAGAAAGTGACAAATTGGATTATGAAATTACGAAACATAATTTTGGAATTGGATTA-** 1101

**Hoya verticillata NC_085236.1**  **TCCAAGGATCACTTAGAAAGTGACAAATTGGATTATGAAATTACGAAACATAATTTTGGAATTGGATTA-** 1004

**Papuahoya urniflora MW719062.1** **TCCAAGGATCACTTAGAAAGTGACAAATTGGATTATGAAATTACGAAACATAATTTTGGAATTGGATTA-** 1201

**Stephanotis volubilis OP133576** **TCCAAGGATCACTTAGAAAGTGACAAATTGGATTATGAAATTACGAAACATAATTTTGGAATTGGATTA-** 1047

**1690 1700 1710 1720 1730 1740 1750**

**....|....|....|....|....|....|....|....|....|....|....|....|....|....|**

**Dischidia australis NC_067885.** **---------------------------------ATACTTCCAATTGAATCAGTATGAGTAAAGGATCCAT** 1131

**Gymnema yunnanense NC_079598.1** **---------------------------------ATACTTCCAATTGAATCAGTATGAGTAAAGGATCCAT** 1129

**Hoya ariadna NC_069568.1**  **---------------------------------ATACTTCCAATTGAATCAGTATGAGTAAAGGATCCAT** 1104

**Hoya commutata NC_067958.1**  **GAAATTACGAAACATAATTTTGGAATTGGATTAATACTTCCAATTGAATCAGTATGAGTAAAGGATCCAT** 1121

**Hoya dimorpha NC_067959.1**  **---------------------------------ATACTTCCAATTGAATCAGTATGAGTAAAGGATCCAT** 1272

**Hoya exilis MW719054.1**  **---------------------------------ATACTTCAAATTGAATCAGTATGAGTAAAGGATCCAT** 1563

**Hoya griffithii NC_069565.1**  **---------------------------------ATACTTCCAATTGAATCAGTATGAGTAAAGGATCCAT** 1183

**Hoya kerrii NC_069570.1**  **---------------------------------ATACTTCCAATTGAATCAGTATGAGTAAAGGATCCAT** 1144

**Hoya lacunosa NC_069564.1**  **---------------------------------ATACTTCCAATTGAATCAGTATGAGTAAAGGATCCAT** 1034

**Hoya lanceolata NC_067960.1**  **---------------------------------ATACTTCCAATTGAATCAGTATGAGTAAAGGATCCAT** 1078

**Hoya liangii OL826865.1**  **---------------------------------ATACTTCCAATTGAATCAGTATGAGTAAAGGATCCAT** 1156

**Hoya lithophytica MW719058.1**  **---------------------------------ATACTTCCAATTGAATCAGTATGAGTAAAGGATCCAT** 1219

**Hoya lockii OR475243.1**  **---------------------------------ATACTTCAAATTGAATCAGTATGAGTAAAGGATCCAT** 1265

**Hoya longifolia NC_069560.1**  **---------------------------------ATACTTCCAATTGAATCAGTATGAGTAAAGGATCCAT** 1172

**Hoya lyi MW719055.1**  **---------------------------------ATACTTCCAATTGAATCAGTATGAGTAAAGGATCCAT** 1265

**Hoya megalaster MW719063.1**  **---------------------------------ATACTTCCAATTGAATCAGTATGAGTAAAGGATCCAT** 1294

**Hoya meliflua NC_069571.1**  **---------------------------------ATACTTCCAATTGAATCAGTATGAGTAAAGGATCCAT** 1150

**Hoya monetteae MW719053.1**  **---------------------------------ATACTTCCAATTGAATCAGTATGAGTAAAGGATCCAT** 1238

**Hoya omlorii MW719060.1**  **---------------------------------ATACTTCCAATTGAATCAGTATGAGTAAAGGATCCAT** 1106

**Hoya ovalifolia NC_069563.1**  **---------------------------------ATACTTCCAATTGAATCAGTATGAGTAAAGGATCCAT** 1047

**Hoya pandurata NC_069562.1**  **---------------------------------ATACTTCCAATTGAATCAGTATGAGTAAAGGATCCAT** 1035

**Hoya pottsii OL754664.1**  **---------------------------------ATACTTCCAATTGAATCAGTATGAGTAAAGGATCCAT** 1054

**Hoya pubicalyx NC_069561.1**  **---------------------------------ATACTTCCAATTGAATCAGTATGAGTAAAGGATCCAT** 1224

**Hoya radicalis NC_067961.1**  **---------------------------------ATACTTCCAATTGAATCAGTATGAGTAAAGGATCCAT** 1028

**Hoya rigida NC_067962.1**  **---------------------------------ATACTTCCAATTGAATCAGTATGAGTAAAGGATCCAT** 1188

**Hoya silvatica NC_067963.1**  **---------------------------------ATACTTCCAATTGAATCAGTATGAGTAAAGGATCCAT** 1054

**Hoya thomsonii NC_067612.1**  **---------------------------------ATACTTCCAATTGAATCAGTATGAGTAAAGGATCCAT** 1138

**Hoya verticillata NC_085236.1**  **---------------------------------ATACTTCCAATTGAATCAGTATGAGTAAAGGATCCAT** 1041

**Papuahoya urniflora MW719062.1** **---------------------------------ATACTTCCAATTGAATCAGTATGAGTAAAGGATCCAT** 1238

**Stephanotis volubilis OP133576** **---------------------------------ATACTTCCAATTGAATCAGTATGAGTAAAGGATCCAT** 1084

**1760 1770 1780 1790 1800 1810 1820**

**....|....|....|....|....|....|....|....|....|....|....|....|....|....|**

**Dischidia australis NC_067885.** **GGATAAAGATAGCAAGTAGGTTTCTAATCGTAACTAAATCTTCAATTTTTGCTTTACAAATAAAAAATTG** 1201

**Gymnema yunnanense NC_079598.1** **GGATGAAGATAGAAAGTAGGTTTCTAATCGTAACTAAATCTTCAATTTTTGCTTTACAAATAAAAAATTG** 1199

**Hoya ariadna NC_069568.1**  **GGATAAAGATAGCAAGTAGGTTTCTAATCGTAACTAAATCTTCAATTTTTGCTTTACAAATACAAAATTG** 1174

**Hoya commutata NC_067958.1**  **GGATAAAGATAGCAAGTAGGTTTCTAATCGTAACTAAATCTTCAATTTTTGCTTTACAAATACAAAATTG** 1191

**Hoya dimorpha NC_067959.1**  **GGATAAAGATAGCAAGTAGGTTTCTAATCGTAACTAAATCTTCAATTTTTGCTTTACAAAAAAAAAATTG** 1342

**Hoya exilis MW719054.1**  **GGATAAAGATAGCAAGTAGGTTTCTAATCGTAACTAAATCTTCAATTTTTGCTTTACAAATAAAAAATTG** 1633

**Hoya griffithii NC_069565.1**  **GGATAAAGATAGCAAGTAGGTTTCTAATCGTAACTAAATCTTCAATTTTTGCTTTACAAATACAAAATTG** 1253

**Hoya kerrii NC_069570.1**  **GGATAAAGATAGCAAGTAGGTTTCTAATCGTAACTAAATCTTCAATTTTTGCTTTACAAATACAAAATTG** 1214

**Hoya lacunosa NC_069564.1**  **GGATAAAGATAGCAAGTAGGTTTCTAATCGTAACTAAATCTTCAATTTTTGCTTTACAAATACAAAATTG** 1104

**Hoya lanceolata NC_067960.1**  **GGATAAAGATAGCAAGTAGGTTTCTAATCGTAACTAAATCTTCAATTTTTGCTTTACAAATAAAAAATTG** 1148

**Hoya liangii OL826865.1**  **GGATAAAGATAGCAAGTAGGTTTCTAATCGTAACTAAATCTTCAATTTTTGCTTTACAAATACAAAATTG** 1226

**Hoya lithophytica MW719058.1**  **GGATAAAGATAGCAAGTAGGTTTCTAATCGTAACTAAATCTTCAATTTTTGCTTTACAAATAAAAAATTG** 1289

**Hoya lockii OR475243.1**  **GGATAAAGATAGCAAGTAGGTTTCTAATCGTAACTAAATCTTCAATTTTTTCTTTACAAATAAAAAATTG** 1335

**Hoya longifolia NC_069560.1**  **GGATAAAGATAGCAAGTAGGTTTCTAATCGTAACTAAATCTTCAATTTTTGCTTTACAAATACAAAATTG** 1242

**Hoya lyi MW719055.1**  **GGATAAAGATAGCAAGTAGGTTTCTAATCGTAACTAAATCTTCAATTTTTGCTTTACAAATACAAAATTG** 1335

**Hoya megalaster MW719063.1**  **GGATAAAGATAGCAAGTAGGTTTCTAATCGTAACTAAATCTTCAATTTTTGCTTTACAAATAAAAAATTG** 1364

**Hoya meliflua NC_069571.1**  **GGATAAAGATAGCAAGTAGGTTTCTAATCGTAACTAAATCTTCAATTTTTGCTTTACAAATACAAAATTG** 1220

**Hoya monetteae MW719053.1**  **GGATAAAGATAGCAAGTAGGTTTCTAATCGTAACTAAATCTTCAATTTTTGCTTTACAAATACAAAATTG** 1308

**Hoya omlorii MW719060.1**  **GGATAAAGATAGCAAGTAGGTTTCTAATCGTAACTAAATCTTCAATTTTTGCTTTACAAATACAAAATTG** 1176

**Hoya ovalifolia NC_069563.1**  **GGATAAAGATAGCAAGTAGGTTTCTAATCGTAACTAAATCTTCAATTTTTGCTTTACAAATACAAAATTG** 1117

**Hoya pandurata NC_069562.1**  **GGATAAAGATAGCAAGTAGGTTTCTAATCGTAACTAAATCTTCAATTTTTGCTTTACAAATACAAAATTG** 1105

**Hoya pottsii OL754664.1**  **GGATAAAGATAGCAAGTAGGTTTCTAATCGTAACTAAATCTTCAATTTTTGCTTTACAAATACAAAATTG** 1124

**Hoya pubicalyx NC_069561.1**  **GGATAAACATAGCAAGTAGGTTTCTAATCGTAACTAAATCTTCAATTTTTGCTTTACAAATACAAAATTG** 1294

**Hoya radicalis NC_067961.1**  **GGATAAAGATAGCAAGTAGGTTTCTAATCGTAACTAAATCTTCAATTTTTGCTTTACAAATACAAAATTG** 1098

**Hoya rigida NC_067962.1**  **GGATAAAGATAGCAAGTAGGTTTCTAATCGTAACTAAATCTTCAATTTTTGCTTTACAAATACAAAATTG** 1258

**Hoya silvatica NC_067963.1**  **GGATAAAGATAGCAAGTAGGTTTCTAATCGTAACTAAATCTTCAATTTTTGCTTTACAAATACAAAATTG** 1124

**Hoya thomsonii NC_067612.1**  **GGATAAAGATAGCAAGTAGGTTTCTAATCGTAACTAAATCTTCAATTTTTGCTTTACAAATACAAAATTG** 1208

**Hoya verticillata NC_085236.1**  **GGATAAAGATAGCAAGTAGGTTTCTAATCGTAACTAAATCTTCAATTTTTGCTTTACAAATACAAAATTG** 1111

**Papuahoya urniflora MW719062.1** **GGATAAAGATAGCAAGTAGGTTTCTAATCGTAACTAAATCTTCAATTTTTGCTTTACAAATAAAAAATTG** 1308

**Stephanotis volubilis OP133576** **GGATGAAGATAGAAAGTAGGTTTCTAATCGTAACTAAATCTTCAATTTTTGCTTTACAAATAAAAAATTG** 1154

**1830 1840 1850 1860 1870 1880 1890**

**....|....|....|....|....|....|....|....|....|....|....|....|....|....|**

**Dischidia australis NC_067885.** **AATCAAAATAGCTATTAAAT-----GATGACTCTGGTTTACTAGAGGCATCGACCCGTTTTTTTAGCTCG** 1266

**Gymnema yunnanense NC_079598.1** **AATAAAAATAGCTATTAAAT-----GATGACTCTGGTTTACTAGAGGCATCGACCTGTTTTTTTAGCTCG** 1264

**Hoya ariadna NC_069568.1**  **AATCAAAATAGCTATTAAAT-----GATGACTCTGGTTTACTAGAGGCATCGACCCGTTTTTTTAGCTCG** 1239

**Hoya commutata NC_067958.1**  **AATAAAAATAGCTATTAAAT-----GATGCCTCTGGTTTACTAGAGGCATCGACCCGTTTTTTTAGCTCG** 1256

**Hoya dimorpha NC_067959.1**  **AATCAAAATAGCTATTAAAT-----GATGACTCTGGTTTACTAGAGGCATCGACCTGTTTTTTTAGCTCG** 1407

**Hoya exilis MW719054.1**  **AATCAAAATAGCTATTAAAT-----GATGACTCTGGTTTACTAGAGGCATCGACCTGTTTTTTTAGCTCG** 1698

**Hoya griffithii NC_069565.1**  **AATAAAAATAGCTATTAAAT-----GATGACTCTGGTTTACTAGAGGCATCGACCCGTTTTTTTAGCTCG** 1318

**Hoya kerrii NC_069570.1**  **AATAAAAATAGCTATTAAAT-----GATGACTCTGGTTTACTAGAGGCATCGACCCGTTTTTTTAGCTCG** 1279

**Hoya lacunosa NC_069564.1**  **AATAAAAATAGCTATTAAATTAAATGATGACTCTGGTTTACTAGAGGCATCGACCCGTTTTTTTAGCTCG** 1174

**Hoya lanceolata NC_067960.1**  **AATCAAAATAGCTATTAAAT-----GATGACTCTGGTTTACTAGAGGCATCGACCCGTTTTTTTAGCTCG** 1213

**Hoya liangii OL826865.1**  **AATAAAAATAGCTATTAAAT-----GATGACTCTGGTTTACTAGAGGCATCGACCCGTTTTTTTAGCTCG** 1291

**Hoya lithophytica MW719058.1**  **AATCAAAATAGCTATTAAAT-----GATGCCTCTGGTTTACTAGAGGCATCGACCTGTTTTTTTAGCTCG** 1354

**Hoya lockii OR475243.1**  **AATCAAAATAGCTATTAAAT-----GATGACTCTGGTTTACTAGAGTCATCGACCCGTTTTTTTAGCTCG** 1400

**Hoya longifolia NC_069560.1**  **AATAAAAATAGCTATTAAAT-----GATGACTCTGGTTTACTAGAGGCATCGACCCGTTTTTTTAGCTCG** 1307

**Hoya lyi MW719055.1**  **AATAAAAATAGCTATTAAAT-----GATGACTCTGGTTTACTAGAGGCATCGACCTGTTTTTTTAGCTCG** 1400

**Hoya megalaster MW719063.1**  **AATCAAAATAGCTATTAAAT-----GATGACTCTGGTTTACTAGAGGCACCGACCTGTTTTTTTAGCTCG** 1429

**Hoya meliflua NC_069571.1**  **AATAAAAATAGCTATTAAAT-----GATGACTCTGGTTTACTAGAGGCATCGACCCGTTTTTTTAGCTCG** 1285

**Hoya monetteae MW719053.1**  **AATAAAAATAGCTATTAAAT-----GATGACTCTGGTTTACTAGAGGCATCAACCTGTTTTTTTAGCTCG** 1373

**Hoya omlorii MW719060.1**  **AATCAAAATAGCTATTAAAT-----GATGACTCTGGTTTACTAGAGGCATCGACCCGTTTTTTTAGCTCG** 1241

**Hoya ovalifolia NC_069563.1**  **AATAAAAATAGCTATTAAAT-----GATGACTCTGGTTTACTAGAGGCATCGACCTGTTTTTTTAGCTCG** 1182

**Hoya pandurata NC_069562.1**  **AATAAAAATAGCTATTAAAT-----GATGACTCTGGTTTACTAGAGGCATCGACCCGTTTTTTTAGCTCG** 1170

**Hoya pottsii OL754664.1**  **AATAAAAATAGCTATTAAAT-----GATGCCTCTGGTTTACTAGAGGCATCGACCCGTTTTTTTAGCTCG** 1189

**Hoya pubicalyx NC_069561.1**  **AATAAAAATAGCTATTAAAT-----GATGACTCTGGTTTACTAGAGGCATCGACCTGTTTTTTTAGCTCG** 1359

**Hoya radicalis NC_067961.1**  **AATAAAAATAGCTATTAAAT-----GATGACTCTGGTTTACTAGAGGCATCGACCCGTTTTTTTAGCTCG** 1163

**Hoya rigida NC_067962.1**  **AATAAAAATAGCTATTAAAT-----GATGACTCTGGTTTACTAGAGGCATCGACCCGTTTTTTTAGCTCG** 1323

**Hoya silvatica NC_067963.1**  **AATAAAAATAGCTATTAAAT-----GATGCCTCTGGTTTACTAGAGGCATCGACCCGTTTTTTTAGCTCG** 1189

**Hoya thomsonii NC_067612.1**  **AATAAAAATAGCTATTAAAT-----GATGACTCTGGTTTACTAGAGGCATCGACCCGTTTTTTTAGCTCG** 1273

**Hoya verticillata NC_085236.1**  **AATAAAAATAGCTATTAAAT-----GATGACTCTGGTTTACTAGAGGCATCGACCTGTTTTTTTAGCTCG** 1176

**Papuahoya urniflora MW719062.1** **AATCAAAATAGCTATTAAAT-----GATGACTCTGGTTTACTAGAGGCATCGACCCGTTTTTTTAGCTCG** 1373

**Stephanotis volubilis OP133576** **AATCAAAATAGCTATTAAAT-----GATGACTCTGGTTTACTAGAGGCATCGACCTGTTTTTTTAGCTCG** 1219

**1900 1910 1920 1930 1940 1950 1960**

**....|....|....|....|....|....|....|....|....|....|....|....|....|....|**

**Dischidia australis NC_067885.** **GTGGAAACAAAATCCCTTTCCTCAGGACCGTCTCAAATAAAAATAAAGAACGAAGTAACTAGAAAGATTG** 1336

**Gymnema yunnanense NC_079598.1** **GTGGAAACAAAATCCCTTTCCTCAGGACCGTCTCAAATAAAAATAGAGAACGAAGTAACTAGAAAGATTG** 1334

**Hoya ariadna NC_069568.1**  **GTGGAAACAAAATCCCTTTCCTCAGGACCGTCTCAAATAAAAATAGAGAACGAAGTAACTAGAAAGATTG** 1309

**Hoya commutata NC_067958.1**  **GTGGAAACAAAATCCCTTTCCTCAGGACCGTCTCAAATAAAAATAGAGAACGAAGTAACTAGAAAGATTG** 1326

**Hoya dimorpha NC_067959.1**  **GTGGAAACAAAATCCCTTTCCTCAGGACCCTCTCAAATAAAAATAGAGAACGAAGTAACTAGAAAGATTG** 1477

**Hoya exilis MW719054.1**  **GTGGAAACAAAATCCCTTTCCTCAGGACCGTCTCAAATAAAAATAGAGAACGAAGTAACTAGAAAGATTG** 1768

**Hoya griffithii NC_069565.1**  **GTGGAAACAAAATCCCTTTCCTCAGGACCGTCTCAAATAAAAATAGAGAACGAAGTAACTAGAAAGATTG** 1388

**Hoya kerrii NC_069570.1**  **GTGGAAACAAAATCCCTTTCCTCAGGACCGTCTCAAATAAAAATAGAGAACGAAGTAACTAGAAAGATTG** 1349

**Hoya lacunosa NC_069564.1**  **GTGGAAACAAAATCCCTTTCCTCAGGACCGTCTCAAATAAAAATAGAGAACGAAGTAACTAGAAAGATTG** 1244

**Hoya lanceolata NC_067960.1**  **GTGGAAACAAAATCCCTTTCCTCAGGACCGTCTCAAATAAAAATAGAGAACGAAGTAACTAGAAAGATTG** 1283

**Hoya liangii OL826865.1**  **GTGGAAACAAAATCCCTTTCCTCAGGACCGTCTCAAATAAAAATAGAGAACGAAGTAACTAGAAAGATTG** 1361

**Hoya lithophytica MW719058.1**  **GTGGAAACAAAATCCCTTTCCTCAGGACCGTCTCAAATAAAAATAGAGAACGAAGTAACTAGAAAGATTG** 1424

**Hoya lockii OR475243.1**  **GTGGAAACAAAATCTCTTTCCTCAGGACCGTCTCAAATAAAAATAGAGAACGAAGTAACTAGAAAGATTG** 1470

**Hoya longifolia NC_069560.1**  **GTGGAAACAAAATCCCTTTCCTCAGGACCGTCTCAAATAAAAATAGAGAACGAAGTAACTAGAAAGATTG** 1377

**Hoya lyi MW719055.1**  **GTGGAAACAAAATCCCTTTCCTCAGGACCGTCTCAAATAAAAATAGAGAACGAAGTAACTAGAAAGATTG** 1470

**Hoya megalaster MW719063.1**  **GTGGAAACAAAATCCCTTTCCTCAGGATCCTCTCAAATAAAAATAGAGAACGAAGTAACTAGAAAGATTG** 1499

**Hoya meliflua NC_069571.1**  **GTGGAAACAAAATCCCTTTCCTCAGGACCGTCTCAAA-AAAAATAGAGAACGAAGTAACTAGAAAGATTG** 1354

**Hoya monetteae MW719053.1**  **GTGGAAACAAAATCCCTTTCCTCAGGACCGTCTCAAATAAAAATAGAGAACGAAGTAACTAGAAAGATTG** 1443

**Hoya omlorii MW719060.1**  **GTGGAAACAAAATCCCTTTCCTCAGGACCGTCTCAAATAAAAATAGAGAACGAAGTAACTAGAAAGATTG** 1311

**Hoya ovalifolia NC_069563.1**  **GTGGAAACAAAATCCCTTTCCTCAGGACCGTCTCAAATAAAAATAGAGAACGAAGTAACTAGAAAGATTG** 1252

**Hoya pandurata NC_069562.1**  **GTGGAAACAAAATCCCTTTCCTCAGGACCGTCTCAAATAAAAATAGAGAACGAAGTAACTAGAAAGATTG** 1240

**Hoya pottsii OL754664.1**  **GTGGAAACAAAATCCCTTTCCTCAGGACCGTCTCAAATAAAAATAGAGAACGAAGTAACTAGAAAGATTG** 1259

**Hoya pubicalyx NC_069561.1**  **GTGGAAACAAAATCCCTTTCCTCAGGACCGTCTCAAATAAAAATAGAGAACGAAGTAACTAGAAAGATTG** 1429

**Hoya radicalis NC_067961.1**  **GTGGAAACAAAATCCCTTTCCTCAGGACCGTCTCAAATAAAAATAGAGAACGAAGTAACTAGAAAGATTG** 1233

**Hoya rigida NC_067962.1**  **GTGGAAACAAAATCCCTTTCCTCAGGACCGTCTCAAATAAAAATAGAGAACGAAGTAACTAGAAAGATTG** 1393

**Hoya silvatica NC_067963.1**  **GTGGAAACAAAATCCCTTTCCTCAGGACCGTCTCAAATAAAAATAGAGAACGAAGTAACTAGAAAGATTG** 1259

**Hoya thomsonii NC_067612.1**  **GTGGAAACAAAATCCCTTTCCTCAGGACCGTCTCAAATAAAAATAGAGAACGAAGTAACTAGAAAGATTG** 1343

**Hoya verticillata NC_085236.1**  **GTGGAAACAAAATCCCTTTCCTCAGGACCGTCTCAAATAAAAATAGAGAACGAAGTAACTAGAAAGATTG** 1246

**Papuahoya urniflora MW719062.1** **GTGGAAACAAAATCCCTTTCCTCAGGACCGTCTCAAATAAAAATAGAGAACGAAGTAACTAGAAAGATTG** 1443

**Stephanotis volubilis OP133576** **GTGGAAACAAAATCCCTTTCCTCAGGACCGTCTCAAATAAAAATAGAGAACGAAGTAACTAGAAAGATTG** 1289

**1970 1980 1990 2000 2010 2020 2030**

**....|....|....|....|....|....|....|....|....|....|....|....|....|....|**

**Dischidia australis NC_067885.** **TTAGAATTACTCTCTTCTAGAGGGATCATCTAGAAAGAAATAAGTAGTCAGACAAAAGTTGACATAGATG** 1406

**Gymnema yunnanense NC_079598.1** **TTAGAATTACTCTCTTCTAGAGGGATCATCTAGAAAACAATTAGTAGTCAGACAAAAGTTGACATAGATG** 1404

**Hoya ariadna NC_069568.1**  **TTAGAATTACTCTCTTCTAGAGGGATCATCTAGAAAGAAATTAGTAGTCAGACAAAAGTTGACATAGATG** 1379

**Hoya commutata NC_067958.1**  **TTAGAATTACTCTCTTCTAGAGGGATCATCTAGAAAGAAATAAGTAGTCAGACAAAAGTTGACATAGATG** 1396

**Hoya dimorpha NC_067959.1**  **TTAGAATTACTCTCTTCTAGAGGGATCATCTAGAAAGAAATTAGTAGTCAGACAAAAGTTGACATAGATG** 1547

**Hoya exilis MW719054.1**  **TTAGAATTACTCTCTTCTAGAGGGATCATCTAGAAAGAAATTAGTAGTCAGACAAAAGTTGACATAGATG** 1838

**Hoya griffithii NC_069565.1**  **TTAGAATTACTCTCTTCTAGAGGGATCATCTAGAAAGAAATAAGTAGTCAGACAAAAGTTGACATAGATG** 1458

**Hoya kerrii NC_069570.1**  **TTAGAATTACTCTCTTCTAGAGGGATCATCTAGAAAGAAATAAGTAGTCAGACAAAAGTTGACATAGATG** 1419

**Hoya lacunosa NC_069564.1**  **TTAGAATTACTCTCTTCTAGAGGGATCATCTAGAAAGAAATAAGTAGTCAGACAAAAGTTGACATAGATG** 1314

**Hoya lanceolata NC_067960.1**  **TTAGAATTACTCTCTTCTAGAGGGATCATCTAGAAAGAAATTAGTAGTCAGACAAAAGTTGACATAGATG** 1353

**Hoya liangii OL826865.1**  **TTAGAATTACTCTCTTCTAGAGGGATCATCTAGAAAGAAATAAGTAGTCAGACAAAAGTTGACATAGATG** 1431

**Hoya lithophytica MW719058.1**  **TTAGAATTACTCTCTTCTAGAGGGATCATCTAGAAAGAAATTAGTAGTCAGACAAAAGTTGACATAGATG** 1494

**Hoya lockii OR475243.1**  **TTAGAATTACTCTCTTCTAGAGGGATCATCTATAAAGAAATTAGTAGTCAGACAAAAGTTGACATAGATG** 1540

**Hoya longifolia NC_069560.1**  **TTAGAATTACTCTCTTCTAGAGGGATCATCTAGAAAGAAATAAGTAGTCAGACAAAAGTTGACATAGATG** 1447

**Hoya lyi MW719055.1**  **TTAGAATTACTCTCTTCTAGAGGGATCATCTAGAAAGAAATAAGTAGTCAGACAAAAGTTGACATAGATG** 1540

**Hoya megalaster MW719063.1**  **TTAGAATTACTCTCTTCTAGAGGGATCATCTAGAAAGAAATTAGTAGTCAGACAAAAGTTGACATAGATG** 1569

**Hoya meliflua NC_069571.1**  **TTAGAATTACTCTCTTCTAGAGGGATCATCTAGAAAGAAATAAGTAGTCAGACAAAAGTTGACATAGATG** 1424

**Hoya monetteae MW719053.1**  **TTAGAATTACTCTCTTCTAGAGGGATCATCTAGAAAGAAATAAGTA---------AAGTTGACATAGATG** 1504

**Hoya omlorii MW719060.1**  **TTAGAATTACTCTCTTCTAGAGGGATCATCTAGAAAGAAATTAGTAGTCAGACAAAAGTTGACATAGATG** 1381

**Hoya ovalifolia NC_069563.1**  **TTAGAATTACTCTCTTCTAGAGGGATCATCTAGAAAGAAATAAGTAGTCAGACAAAAGTTGACATAGATG** 1322

**Hoya pandurata NC_069562.1**  **TTAGAATTACTCTCTTCTAGAGGGATCATCTAGAAAGAAATAAGTAGTCAGACAAAAGTTGACATAGATG** 1310

**Hoya pottsii OL754664.1**  **TTAGAATTACTCTCTTCTAGAGGGATCATCTAGAAAGAAATAAGTAGTCAGACAAAAGTTGACATAGATG** 1329

**Hoya pubicalyx NC_069561.1**  **TTAGAATTACTCTCTTCTAGAGGGATCATCTAGAAAGAAATAAGTAGTCAGACAAAAGTTGACATAGATG** 1499

**Hoya radicalis NC_067961.1**  **TTAGAATTACTCTCTTCTAGAGGGATCATCTAGAAAGAAATAAGTAGTCAGACAAAAGTTGACATAGATG** 1303

**Hoya rigida NC_067962.1**  **TTAGAATTACTCTCTTCTAGAGGGATCATCTAGAAAGAAATAAGTAGTCAGACAAAAGTTGACATAGATG** 1463

**Hoya silvatica NC_067963.1**  **TTAGAATTACTCTCTTCTAGAGGGATCATCTAGAAAGAAATAAGTAGTCAGACAAAAGTTGACATAGATG** 1329

**Hoya thomsonii NC_067612.1**  **TTAGAATTACTCTCTTCTAGAGGGATCATCTAGAAAGAAATAAGTAGTCAGACAAAAGTTGACATAGATG** 1413

**Hoya verticillata NC_085236.1**  **TTAGAATTACTCTCTTCTAGAGGGATCATCTAGAAAGAAATAAGTAGTCAGACAAAAGTTGACATAGATG** 1316

**Papuahoya urniflora MW719062.1** **TTATAATTACTCTCTTCTAGAGGGATCATCTAGAAAGAAATAAGTAGTCAGACAAAAGTTGACATAGATG** 1513

**Stephanotis volubilis OP133576** **TTAGAATTACTCTCTTCTAGAGGGATCATCTAGAAAGCAATTAGTAGTCAGACAAAAGTTGACATAGATG** 1359

**2040 2050 2060 2070 2080 2090 2100**

**....|....|....|....|....|....|....|....|....|....|....|....|....|....|**

**Dischidia australis NC_067885.** **TTATGGGTAGAATTTTTTTT-GTAAGTTTGTTCACATACATATCCATAAAGGAGCCGAATGAAACCAAAG** 1475

**Gymnema yunnanense NC_079598.1** **TTATGGGTAGAATTTTTTTTTGTAAGTTTGTTCACATCCATATCCATAAAGGAGCCGAATGAAACCAAAG** 1474

**Hoya ariadna NC_069568.1**  **TTATGGGTAGAATTTTTTTT-GTAAGTTTGTTCACATCCATATCCATAAAGGAGCCGAATGAAACCAAAG** 1448

**Hoya commutata NC_067958.1**  **TTATGGGTATAATTTTTTTT-GTAAGTTTGTTCACATCCATATCCATAAAGGAGCCGAATGAAACCAAAG** 1465

**Hoya dimorpha NC_067959.1**  **TTATGGGTAGAATTTTTTTT-GTAAGTTTGTTCACATCCATATCCATAAAGGAGCCGAATGAAACCAAAG** 1616

**Hoya exilis MW719054.1**  **TTATGGGTAGAATTTTTTTT-GTAAGTTTGTTCACATCCATATCCATAAAGGAGCCGAATGAAACCAAAG** 1907

**Hoya griffithii NC_069565.1**  **TTATGGGTAGAATTTTTTTT-GTAAGTTTGTTCACATCCATATCCATAAAGGAGCCGAATGAAACCAAAG** 1527

**Hoya kerrii NC_069570.1**  **TTATGGGTAGAATTTTTTTT-GTAAATTTGTTCACATCCATATCCATAAAGGAGCCGAATGAAACCAAAG** 1488

**Hoya lacunosa NC_069564.1**  **TTATGGGTAGAATTTTTTTT-GTAAGTTTGTTCACATCCATATCCATAAAGGAGCCGAATGAAACCAAAG** 1383

**Hoya lanceolata NC_067960.1**  **TTATGGGTAGAATTTTTTTT-GTAAGTTTGTTTACATCCATATCCATAAAGGAGCCGAATGAAACCAAAG** 1422

**Hoya liangii OL826865.1**  **TTATGGGTAGAATTTTTTTT-GTAAGTTTGTTCACATCCATATCCATAAAGGAGCCGAATGAAACCAAAG** 1500

**Hoya lithophytica MW719058.1**  **TTATGGGTAGAATTTTTTTT-GTAAGTTTGTTCACATCCATATCCATAAAGGAGCCGAATGAAACCAAAG** 1563

**Hoya lockii OR475243.1**  **TTATGGGTAGAATTTTTTTTTGTAAGTTTGTTCACATCCATATCCATAAAGGAGCCGAATGAAACCAAAG** 1610

**Hoya longifolia NC_069560.1**  **TTATGGGTAGAATTTTTTTT-GTAAGTTTGTTCACATCCATATCCATAAAGGAGCCGAATGAAACCAAAG** 1516

**Hoya lyi MW719055.1**  **TTATGGGTAGAATTTTTTTT-GTAAGTTTGTTCACATCCATATCCATAAAGGAGCCGAATGAAACCAAAG** 1609

**Hoya megalaster MW719063.1**  **TTATGGGTAGAATTTTTTTT-GTAAGTTTGTTCACATCCATATCCATAAAGGAGCCGAATGAAACCAAAG** 1638

**Hoya meliflua NC_069571.1**  **TTATGGGTAGAATTTTTTTT-GTAAGTTTGTTCACATCCATATCCATAAAGGAGCCGAATGAAACCAAAG** 1493

**Hoya monetteae MW719053.1**  **TTATGGGTAGAATTTTTTTT-GTAAGTTTGTTCACATCCATATCCATAAAGGAGCCGAATGAAACCAAAG** 1573

**Hoya omlorii MW719060.1**  **TTATGGGTAGAATTTTTTTT-GTAAGTTTGTTCACATCCATATCCATAAAGGAGCCGAATGAAACCAAAG** 1450

**Hoya ovalifolia NC_069563.1**  **TTATGGGTAGAATTTTTTCT-GTAAGTTTGTTCACATCCATATCCATAAAGGAGCCGAATGAAACCAAAG** 1391

**Hoya pandurata NC_069562.1**  **TTATGGGTAGAATTTTTTTT-GTAAGTTTGTTCACATCCATATCCATAAAGGAGCCGAATGAAACCAAAG** 1379

**Hoya pottsii OL754664.1**  **TTATGGGTATAATTTTTTTT-GTAAGTTTGTTCACATCCATATCCATAAAGGAGCCGAATGAAACCAAAG** 1398

**Hoya pubicalyx NC_069561.1**  **TTATGGGTAGAATTTTTTTT-GTAAGTTTGTTCACATCCATATCCATAAAGGAGCCGAATGAAACCAAAG** 1568

**Hoya radicalis NC_067961.1**  **TTATGGGTAGAATTTTTTTT-GTAAGTTTGTTTACATCCATATCCATAAAGGAGCCGAATGAAACCAAAG** 1372

**Hoya rigida NC_067962.1**  **TTATGGGTAGAATTTTTTTT-GTAAGTTTGTTCACATCCATATCCATAAAGGAGCCGAATGAAACCAAAG** 1532

**Hoya silvatica NC_067963.1**  **TTATGGGTATAATTTTTTTT-GTAAGTTTGTTCACATCCATATCCATAAAGGAGCCGAATGAAACCAAAG** 1398

**Hoya thomsonii NC_067612.1**  **TTATGGGTAGAATTTTTTTT-GTAAGTTTGTTCACATCCATATCCATAAAGGAGCCGAATGAAACCAAAG** 1482

**Hoya verticillata NC_085236.1**  **TTATGGGTAGAATTTTTTTT-GTAAGTTTGTTCACATCCATATCCATAAAGGAGCCGAATGAAACCAAAG** 1385

**Papuahoya urniflora MW719062.1** **TTATGGGTAGAATTTTTTTTTGTAAGTTTGTTCACATCCATATCCATAAAGGAGCCGAATGAAACCAAAG** 1583

**Stephanotis volubilis OP133576** **TTATGGGTAGAATTTTTTTTTGTAAGTTTGGTCACATCCATATCCATAAAGGAGCCGAATGAAACCAAAG** 1429

**2110 2120 2130 2140 2150 2160 2170**

**....|....|....|....|....|....|....|....|....|....|....|....|....|....|**

**Dischidia australis NC_067885.** **TTTCATGTTCGGTTTTGAATTAGAGACGTTCAAAATGCTGAATCGACGTCGACTATAACCCCTAGCCTTC** 1545

**Gymnema yunnanense NC_079598.1** **TTTCATGTTCGGTTTTGAATTAGAGACGTTCAAAATGCTGAATCGACGTCGACTATAACCCCTAGCCTTC** 1544

**Hoya ariadna NC_069568.1**  **TTTCATGTTCGGTTTTGAATTAGAGACGTTCAAAATGCTGAATCGACGTCGACTATAACCCCTAGCCTTC** 1518

**Hoya commutata NC_067958.1**  **TTTCATGTTCGGTTTTGAATTAGAGACGTTCAAAATGCTGAATCGACGTCGACTATAACCCCTAGCCTTC** 1535

**Hoya dimorpha NC_067959.1**  **TTTCATGTTCGGTTTTGAATTAGAGACGTTCAAAATGTTGAATCGACGTCGACTATAACCCCTAGCCTTC** 1686

**Hoya exilis MW719054.1**  **TTTCATGTTCGGTTTTGAATTAGAGACGTTCAAAATGCTGAATCGACGTCGACTATAACCCCTAGCCTTC** 1977

**Hoya griffithii NC_069565.1**  **TTTCATGTTCGGTTTTGAATTAGAGACGTTCAAAATGCTGAATCGACGTCGACTATAACCCCTAGCCTTC** 1597

**Hoya kerrii NC_069570.1**  **TTTCATGTTCGGTTTTGAATTAGAGACGTTCAAAATGCTGAATCGACGTCGACTATAACCCCTAGCCTTC** 1558

**Hoya lacunosa NC_069564.1**  **TTTCATGTTCGGTTTTGAATTAGAGACGTTCAAAATGCTGAATCGACGTCGACTATAACCCCTAGCCTTC** 1453

**Hoya lanceolata NC_067960.1**  **TTTCATGTTCGGTTTTGAATTAGAGACGTTCAAAATGCTGAATCGACGTCGACTATAACCCCTAGCCTTC** 1492

**Hoya liangii OL826865.1**  **TTTCATGTTCGGTTTTGAATTAGAGACGTTCAAAATGCTGAATCGACGTCGACTATAACCCCTAGCCTTC** 1570

**Hoya lithophytica MW719058.1**  **TTTCATGTTCGGTTTTGAATTAGAGACGTTCAAAATGCTGAATCGACGTCGACTATAACCCCTAGCCTTC** 1633

**Hoya lockii OR475243.1**  **TTTCATGTTCGGTTTTGAATTAGAGACGTTCAAAATGCTGAATCGACGTCGACTATAACCCCTAGCCTTC** 1680

**Hoya longifolia NC_069560.1**  **TTTCATGTTCGGTTTTGAATTAGAGACGTTCAAAATGCTGAATCGACGTCGACTATAACCCCTAGCCTTC** 1586

**Hoya lyi MW719055.1**  **TTTCATGTTCGGTTTTGAATTAGAGACGTTCAAAATGCTGAATCGACGTCGACTATAACCCCTAGCCTTC** 1679

**Hoya megalaster MW719063.1**  **TTTCATGTTCGGTTTTGAATTAGAGACGTTCAAAATGTTGAATCGACGTCGACTATAACCCCTAGCCTTC** 1708

**Hoya meliflua NC_069571.1**  **TTTCATGTTCGGTTTTGAATTAGAGACGTTCAAAATGCTGAATCGACGTCGACTATAACCCCTAGCCTTC** 1563

**Hoya monetteae MW719053.1**  **TTTCATGTTCGGTTTTGAATTAGAGACGTTCAAAATGCTGAATCGACGTCGACTATAACCCCTAGCCTTC** 1643

**Hoya omlorii MW719060.1**  **TTTCATGTTCGGTTTTGAATTAGAGACGTTCAAAATGCTGAATCGACGTCGACTATAACCCCTAGCCTTC** 1520

**Hoya ovalifolia NC_069563.1**  **TTTCATGTTCGGTTTTGAATTAGAGACGTTCAAAATGCTGAATCGACGTCGACTATAACCCCTAGCCTTC** 1461

**Hoya pandurata NC_069562.1**  **TTTCATGTTCGGTTTTGAATTAGAGACGTTCAAAATGCTGAATCGACGTCGACTATAACCCCTAGCCTTC** 1449

**Hoya pottsii OL754664.1**  **TTTCATGTTCGGTTTTGAATTAGAGACGTTCAAAATGCTGAATCGACGTCGACTATAACCCCTAGCCTTC** 1468

**Hoya pubicalyx NC_069561.1**  **TTTCATGTTCGGTTTTGAATTAGAGACGTTCAAAATGCTGAATCGACGTCGACTATAACCCCTAGCCTTC** 1638

**Hoya radicalis NC_067961.1**  **TTTCATGTTCGGTTTTGAATTAGAGACGTTCAAAATGCTGAATCGACGTCGACTATAACCCCTAGCCTTC** 1442

**Hoya rigida NC_067962.1**  **TTTCATGTTCGGTTTTGAATTAGAGACGTTCAAAATGCTGAATCGACGTCGACTATAACCCCTAGCCTTC** 1602

**Hoya silvatica NC_067963.1**  **TTTCATGTTCGGTTTTGAATTAGAGACGTTCAAAATGCTGAATCGACGTCGACTATAACCCCTAGCCTTC** 1468

**Hoya thomsonii NC_067612.1**  **TTTCATGTTCGGTTTTGAATTAGAGACGTTCAAAATGCTGAATCGACGTCGACTATAACCCCTAGCCTTC** 1552

**Hoya verticillata NC_085236.1**  **TTTCATGTTCGGTTTTGAATTAGAGACGTTCAAAATGCTGAATCGACGTCGACTATAACCCCTAGCCTTC** 1455

**Papuahoya urniflora MW719062.1** **TTTCATGTTCGGTTTTGAATTAGAGACGTTCAAAATGCTGAATCGACGTCGACTATAACCCCTAGCCTTC** 1653

**Stephanotis volubilis OP133576** **TTTCATGTTCGGTTTTGAATTAGAGACGTTCAAAATGCTGAATCGACGTCGACTATAACCCCTAGCCTTC** 1499

**2180 2190 2200 2210 2220 2230 2240**

**....|....|....|....|....|....|....|....|....|....|....|....|....|....|**

**Dischidia australis NC_067885.** **CAAGCTAACGATGCGGGTTCGATTCCCGCTACCCGCTTTCAATTCTTTTTTTT----TTCGAAATTGAAA** 1611

**Gymnema yunnanense NC_079598.1** **CAAGCTAACGATGCGGGTTCGATTCCCGCTACCCGCTTTCTATTCTTTTTTTTTTTATTCGAAATTGAAA** 1614

**Hoya ariadna NC_069568.1**  **CAAGCTAACGATGCGGGTTCGATTCCCGCTACCCGCTTTCAATTCTTTTTTTTT--ATTCGAAATTGAAA** 1586

**Hoya commutata NC_067958.1**  **CAAGCTAACGATGCGGGTTCGATTCCCGCTACCCGCTTTCAATTCTTTTTTTTT--ATTCGCAATTGAAA** 1603

**Hoya dimorpha NC_067959.1**  **CAAGCTAACGATGCGGGTTCGATTCCCGCTACCCGCTTT-----------TTTTT-ATTCGAAATTGAAA** 1744

**Hoya exilis MW719054.1**  **CAAGCTAACGATGCGGGTTCGATTCCCGCTACCCGCTTTCCATTCTTTTTTTTT--ATTCGAAATTGAAA** 2045

**Hoya griffithii NC_069565.1**  **CAAGCTAACGATGCGGGTTCGATTCCCGCTACCCGCTTTCAATTCTTTTTTTT---ATTCGCAATTGAAA** 1664

**Hoya kerrii NC_069570.1**  **CAAGCTAACGATGCGGGTTCGATTCCCGCTACCCGCTTTCAATTCTTTTTTTTT--ATTCGCAATTGAAA** 1626

**Hoya lacunosa NC_069564.1**  **CAAGCTAACGATGCGGGTTCGATTCCCGCTACCCGCTTTCAATTCTTTTTTTTTTTATTCGCAATTGAAA** 1523

**Hoya lanceolata NC_067960.1**  **CAAGCTAACGATGCGGGTTCGATTCCCGCTACCCGCTTTCAATTCTTTTTTTT---ATTCGAAATTGAAA** 1559

**Hoya liangii OL826865.1**  **CAAGCTAACGATGCGGGTTCGATTCCCGCTACCCGCTTTCAATTCTTTTTTTT---ATTCGCAATTGAAA** 1637

**Hoya lithophytica MW719058.1**  **CAAGCTAACGATGCGGGTTCGATTCCCGCTACCCGCTTTCAATTCTTTTTTTTT--ATTCGAAATTGAAA** 1701

**Hoya lockii OR475243.1**  **CAAGCTAACGATGCGGGTTCGATTCCCGCTACCCGCTTTCAATTCTTTTTTTTT--ATTCGAAATTGAAA** 1748

**Hoya longifolia NC_069560.1**  **CAAGCTAACGATGCGGGTTCGATTCCCGCTACCCGCTTTCAATTCTTTTTTTT---ATTCGCAATTGAAA** 1653

**Hoya lyi MW719055.1**  **CAAGCTAACGATGCGGGTTCGATTCCCGCTACCCGCTTTCAATTCTTTTTTTTT--ATTCGCAATTGAAA** 1747

**Hoya megalaster MW719063.1**  **CAAGCTAACGATGCGGGTTCGATTCCCGCTACCCGCTTTCAATTCTTT--TTTTT-ATTCGAAATTTAAA** 1775

**Hoya meliflua NC_069571.1**  **CAAGCTAACGATGCGGGTTCGATTCCCGCTACCCGCTTTCAATTCTTTTTTTTTTTATTCGCAATTGAAA** 1633

**Hoya monetteae MW719053.1**  **CAAGCTAACGATGCGGGTTCGATTCCCGCTACCCGCTTTCAATTCTTTTTTTTT--ATTCGCAATTGAAA** 1711

**Hoya omlorii MW719060.1**  **CAAGCTAACGATGCGGGTTCGATTCCCGCTACCCGCTTTCAATTCTTTTTTTT---ATTCGAAATTGAAA** 1587

**Hoya ovalifolia NC_069563.1**  **CAAGCTAACGATGCGGGTTCGATTCCCGCTACCCGCTTTCAATTCTTTTTTTTT--ATTCGCAATTGAAA** 1529

**Hoya pandurata NC_069562.1**  **CAAGCTAACGATGCGGGTTCGATTCCCGCTACCCGCTTTCAATTCTTTTTTTTT--ATTCGCAATTGAAA** 1517

**Hoya pottsii OL754664.1**  **CAAGCTAACGATGCGGGTTCGATTCCCGCTACCCGCTTTCAATTCTTTTTTTTT--ATTCGCAATTGAAA** 1536

**Hoya pubicalyx NC_069561.1**  **CAAGCTAACGATGCGGGTTCGATTCCCGCTACCCGCTTTCAATTCTTTTTTTTT--ATTCGCAATTGAAA** 1706

**Hoya radicalis NC_067961.1**  **CAAGCTAACGATGCGGGTTCGATTCCCGCTACCCGCTTTCAATTCTTTTTTTTTT-ATTCGCAATTGAAA** 1511

**Hoya rigida NC_067962.1**  **CAAGCTAACGATGCGGGTTCGATTCCCGCTACCCGCTTTCAATTCTTTTTTTTT--ATTCGCAATTGAAA** 1670

**Hoya silvatica NC_067963.1**  **CAAGCTAACGATGCGGGTTCGATTCCCGCTACCCGCTTTCAATTCTTTTTTTTT--ATTCGCAATTGAAA** 1536

**Hoya thomsonii NC_067612.1**  **CAAGCTAACGATGCGGGTTCGATTCCCGCTACCCGCTTTCAATTCTTTTTTTTT--ATTCGCAATTGAAA** 1620

**Hoya verticillata NC_085236.1**  **CAAGCTAACGATGCGGGTTCGATTCCCGCTACCCGCTTTCAATTCTTTTTTTTT--ATTCGCAATTGAAA** 1523

**Papuahoya urniflora MW719062.1** **CAAGCTAACGATGCGGGTTCGATTCCCGCTACCCGCTTTCAATTCTTTTTTTT---ATTCGAAATTGAAA** 1720

**Stephanotis volubilis OP133576** **CAAGCTAACGATGCGGGTTCGATTCCCGCTACCCGCTTTCTATTCTTTTTTTTTTTATTCGAAATTGAAA** 1569

**2250 2260 2270 2280 2290 2300 2310**

**....|....|....|....|....|....|....|....|....|....|....|....|....|....|**

**Dischidia australis NC_067885.** **TTATTATATATTCTAATTCTAGACTTAATGCATCATTTAAT------------ATACAGTTTCAAAAATT** 1669

**Gymnema yunnanense NC_079598.1** **TTATTATATATTCTAATTCTAGACTTAATGCATCATTTAAT------------ATACAGTTTCAAAAATT** 1672

**Hoya ariadna NC_069568.1**  **TTATTATATATTCTAATTCTAGACTTAATGCATCATTTAATGCATCATTTAATATACAGTTTCAAAAATT** 1656

**Hoya commutata NC_067958.1**  **TTTTTATATATTCTAATTCTAGACTTAATGCATCATTTAAT------------ATACAGTTTCAAAAATT** 1661

**Hoya dimorpha NC_067959.1**  **TTTATATATATTCTAATTCTAGACTTAATGCATCATTTAAT------------ATACAGTTTCAAAAATT** 1802

**Hoya exilis MW719054.1**  **TTTATATATATTATAATTCTAGACTTAATGCATCATTTAAT------------ATACAGTTTCAAAAATT** 2103

**Hoya griffithii NC_069565.1**  **TTTTTATATATTCTAATTCTAGACTTAATGCATCATTTAAT------------ATACAGTTTCCAAAATT** 1722

**Hoya kerrii NC_069570.1**  **TTTATATATATTCTAATTCTAGACTTAATGCATCATTTAAT------------ATACAGTTTCAAAAATT** 1684

**Hoya lacunosa NC_069564.1**  **TTTTTATATATTCGAATTCTAGACTTAATGCATCATTTAAT------------ATACAGTTTCAAAAATT** 1581

**Hoya lanceolata NC_067960.1**  **TTTATATATATTCTAATTCTAGACTTAATGCATCATTTAAT------------ATACAGTTTAAAAAATT** 1617

**Hoya liangii OL826865.1**  **TTTTTATATATTCTAATTCTAGACTTAATGCATCATTTAAT------------ATACAGTTTCCAAAATT** 1695

**Hoya lithophytica MW719058.1**  **TTATTATATATTCTAATTCTAGACTTAATGCATCATTTAAT------------ATACAGTTTCAAAAATT** 1759

**Hoya lockii OR475243.1**  **TTTATATATATTATAATTCTAGACTTAATGCACCATTTAAT------------ATACAGTTTCAAAAATT** 1806

**Hoya longifolia NC_069560.1**  **TTTTTATATATTCTAATTCTAGACTTAATGCATCATTTAAT------------ATACAGTTTCCAAAATT** 1711

**Hoya lyi MW719055.1**  **TTTTTATATATTCTAATTCTAGACTTAATGCATCATTTAAT------------ATACAGTTTCAAAAATT** 1805

**Hoya megalaster MW719063.1**  **TTTATATATATTCTAATTCTAGACTTAATGCATCATTTAAT------------ATACAGTTTCAAAAATT** 1833

**Hoya meliflua NC_069571.1**  **TTTTTATATATTCTAATTCTAGACTTAATGCATCATTTAAT------------ATACAGTTTCAAAAATT** 1691

**Hoya monetteae MW719053.1**  **TTTTTATATATTCTAATTCTAGACTTAATGCATCATTTAAT------------ATACAGTTTAAAAAATT** 1769

**Hoya omlorii MW719060.1**  **TTATTATATATTCTAATTCTAGACTTAATGCATCATTTAAT------------ATACAGTTTCAAAAATT** 1645

**Hoya ovalifolia NC_069563.1**  **TTTATATATATTCTAATTCTAGACTTAATGCATCATTTAAT------------ATACAGTTTCAAAAATT** 1587

**Hoya pandurata NC_069562.1**  **TTTTTATATATTCTAATTCTAGACTTAATGCATCATTTAAT------------ATACAGTTTCCAAAATT** 1575

**Hoya pottsii OL754664.1**  **TTTATATATATTCTAATTCTAGACTTAATGCATCATTTAAT------------ATACAGTTTCAAAAATT** 1594

**Hoya pubicalyx NC_069561.1**  **TTTTTATATATTCTAATTCTAGACTTAATGCATCATTTAAT------------ATACAGTTTCAAAAATT** 1764

**Hoya radicalis NC_067961.1**  **TTTATATATATTCTAATTCTAGACTTAATGCATCATTTAAT------------ATACAGTTTCCAAAATT** 1569

**Hoya rigida NC_067962.1**  **TTTATATATATTCTAATTCTAGACTTAATGCATCATTTAAT------------ATACAGTTTCAAAAATT** 1728

**Hoya silvatica NC_067963.1**  **TTTATATATATTCTAATTCTAGACTTAATGCATCATTTAAT------------ATACAGTTTCAAAAATT** 1594

**Hoya thomsonii NC_067612.1**  **TTTTTATATATTCTAATTCTAGACTTAATGCATCATTTAAT------------ATACAGTTTCCAAAATT** 1678

**Hoya verticillata NC_085236.1**  **TTTATATATATTCTAATTCTAGACTTAATGCATCATTTAAT------------ATACAGTTTCAAAAATT** 1581

**Papuahoya urniflora MW719062.1** **TTATTATATATTATAATTCTAGACTTAATGCATCATTTAAT------------ATACAGTTTCCAAAATT** 1778

**Stephanotis volubilis OP133576** **TTATTATATATAATAATTCTAGACTTAATGCATCATTTAAT------------ATACAGTTTCAAAAATT** 1627

**2320 2330 2340 2350 2360 2370 2380**

**....|....|....|....|....|....|....|....|....|....|....|....|....|....|**

**Dischidia australis NC_067885.** **ATCTCACATACAATCCGATTATTTTTTTTTT-CGGCGAAAAAGTGGGGAAAGTAAAAATAGGAAAAAAAT** 1738

**Gymnema yunnanense NC_079598.1** **ATCTCACATACAATCCGATTCTTTTTTTTT--CAGCGAAGAGGTGGGGAAAGTCAAAATACGAAAAAAAC** 1740

**Hoya ariadna NC_069568.1**  **ATCTCACATACAATCCGATTCTTTTTTTTTT-CGGCGAAAAAGTGGGGAAAGTCAAAATACGAAAAAAAT** 1725

**Hoya commutata NC_067958.1**  **ATCTCACATACAATCCGATTCTTGTTTTTTT-CGGCGAAAAAGTGGGGAAAGTCAAAATACGAAAAAAAT** 1730

**Hoya dimorpha NC_067959.1**  **ATCTCACCTACAATCCGATTCTTTTTTTTT--CGGCGAAAAAGTGGGGAAAGTAAAAATACGAAAAAAAT** 1870

**Hoya exilis MW719054.1**  **ATCTCACATACAATCCGATTCTTTTTTTTTT-CGGCGAAAAAGTGGGGAAAGTCAAAATACGAAAAAAAT** 2172

**Hoya griffithii NC_069565.1**  **ATCTCACATACAATCCGATTCTTTTTTTTTT-CGGCGAAAAAGTGGGGAAAGTCAAAATACGAAAAAAAT** 1791

**Hoya kerrii NC_069570.1**  **ATCTCACATACAATCCCATTCTTGTTTTTTT-CGGCGAAAAAGTGGGGAAAGTCAAAATACGAAAAAAAT** 1753

**Hoya lacunosa NC_069564.1**  **ATCTCACATACAATCCGATTCTTTTTTTTTT-CGGCGAAAAAGTGTGGAAAGTCAAAATACGAAAAAAAT** 1650

**Hoya lanceolata NC_067960.1**  **ATATCACATACAATCCGATTCTTTTTTTTTT-CGGCGAAAAAGTGGGGAAAGTCAAAATACGAAAAAAA-** 1685

**Hoya liangii OL826865.1**  **ATCTCACATACAATCCGATTCTTTTTTTTTT-CGGCGAAAAAGTGGGGAAAGTCAAAATACGAAAAAAAT** 1764

**Hoya lithophytica MW719058.1**  **ATCTCACATACAATCCGATTCTTTTTTTTTT-CGGCGAAAAAGTGGGGAAAGTCAAAATACGAAAAAAAT** 1828

**Hoya lockii OR475243.1**  **ATCTCACATACAATCCGATTCTTTTTTTTTT-CGGCGAAAAAGTGGGGAAAGTCAAAATACGAAAAAAAT** 1875

**Hoya longifolia NC_069560.1**  **ATCTCACATACAATCCGATTCTTTTTTTTTT-CGGCGAAAAAGTGGGGAAAGTCAAAATACGAAAAAAAT** 1780

**Hoya lyi MW719055.1**  **ATCTCACATACAATCCGATTCTTGTTTTTTT-CGGCGAAAAAGTGGGGAAAGTCAAAATACGAAAAAAAT** 1874

**Hoya megalaster MW719063.1**  **ATCTCACATACAATCCGATTCTTTTTTTTTT-CGGCGAAAAAGTGGGGAAAGTCAAAATACGAAAAAAAT** 1902

**Hoya meliflua NC_069571.1**  **ATCTCACATACAATCCGATTCTTGTTTTTTT-CGGCGAAAAAGTGGGGAAAGTCAAAATACGAAAAAAAT** 1760

**Hoya monetteae MW719053.1**  **ATCTCACATACAATCCGATTCTTGTTTTTTT-CGGCGAAAAAGTGGGGAAAGTCAAAATACGAAAAAAAT** 1838

**Hoya omlorii MW719060.1**  **ATCTCACATACAATCCGATTCTTTTTTTTTT-CGGCGAAAAAGTGGGGAAAGTCAAAATACGAAAAAAAT** 1714

**Hoya ovalifolia NC_069563.1**  **ATCTCACATACAATCCGATTCTTGTTTTTTT-CGGCGAAAAAGTGGGGAAAGTCAAAATACGAAAAAAAT** 1656

**Hoya pandurata NC_069562.1**  **ATCTCACATACAATCCGATTCTTTTTTTTTTTCGGCGAAAAAGTGGGGAAAGTCAAAATACGAAAAAAAT** 1645

**Hoya pottsii OL754664.1**  **ATCTCACATACAATCCGATTCTTGTTTTTTT-CGGCGAAAAAGTGGGGAAAGTCAAAATACGAAAAAAAT** 1663

**Hoya pubicalyx NC_069561.1**  **ATCTCACATACAATCCGATTCTTGTTTTTTT-CGGCGAAAAAGTGGGGAAAGTCAAAATACGAAAAAAAT** 1833

**Hoya radicalis NC_067961.1**  **ATCTCACATACAATCCGATTCTTTTTTTTT--CGGCGAAAAAGTGGGGAAAGTCAAAATACGAAAAAAAT** 1637

**Hoya rigida NC_067962.1**  **ATCTCACATACAATCCGATTCTTTTTTTTTT-CCGCGAAAAAGTGGGGAAAGTCAAAATACGAAAAAAAT** 1797

**Hoya silvatica NC_067963.1**  **ATCTCACATACAATCCGATTCTTGTTTTTTT-CGGCGAAAAAGTGGGGAAAGTCAAAATACGAAAAAAAT** 1663

**Hoya thomsonii NC_067612.1**  **ATCTCACATACAATCCGATTCTTTTTTTTTTTCGGCGAAAAAGTGGGGAAAGTCAAAATACGAAAAAAAT** 1748

**Hoya verticillata NC_085236.1**  **ATCTCACATACAATCCGATTCTTGTTTTTTT-CGGCGAAAAAGTGGGGAAAGTCAAAATACGAAAAAAAT** 1650

**Papuahoya urniflora MW719062.1** **ATCTCACATACAATCCGATTCTTTTTTTTTTTCGGCGAAAAAGTGGGGAAAGTCAAAATACGAAAAAAAT** 1848

**Stephanotis volubilis OP133576** **ATCTCACATACAATCCGATTCTTTTTTTT---CAGCGAAGAGGTGGGGAA-GTCAAAATACGAAAAAAAT** 1693

**2390 2400 2410 2420 2430 2440 2450**

**....|....|....|....|....|....|....|....|....|....|....|....|....|....|**

**Dischidia australis NC_067885.** **CGGAA---------TGAAAAGCGTCCATTGTCTAATGGATAGGACAGGGGTCTTCTAAACCTTTGGTATA** 1799

**Gymnema yunnanense NC_079598.1** **CGGAA---------TGAAAAGCGTCCATTGTCTAATGGATAGGACAGAGGTCTTCTAAACCTTTGGTATA** 1801

**Hoya ariadna NC_069568.1**  **CGGAA---------TGAAAAGCGTCCATTGTCTAATGGATAGGACAGGGGTCTTCTAAACCTTTGGTATA** 1786

**Hoya commutata NC_067958.1**  **CGGAA---------TGAAAAGCGTCCATTGTCTAATGGATAGGACAGGGGTCTTCTAAACCTTTGGTATA** 1791

**Hoya dimorpha NC_067959.1**  **CGGAA---------TGAAAAGCGTCCATTGTCTAATGGATAGGACAGGGGTCTTCTAAACCTTTGGTATA** 1931

**Hoya exilis MW719054.1**  **CGGAA---------TGAAAAGCGTCCATTGTCTAATGGATAGGACAGGGGTCTTCTAAACCTTTGGTATA** 2233

**Hoya griffithii NC_069565.1**  **CGGAATGATCGGAATGAAAGGCGTCCATTGTCTAATGGATAGGACAGGGGTCTTCTAAACCTTTGGTATA** 1861

**Hoya kerrii NC_069570.1**  **CGGAA---------TGAAAGGCGTCCATTGTCTAATGGATAGGACAGGGGTCTTCTAAACCTTTGGTATA** 1814

**Hoya lacunosa NC_069564.1**  **CGGAA---------TGAAAAGCGTCCATTGTCTAATGGATAGGACAGGGGTCTTCTAAACCTTTGGTATA** 1711

**Hoya lanceolata NC_067960.1**  **CGGAA---------TGAAAAGCGTCCATTGTCTAATGGATAGGACAGGGGTCTTCTAAACCTTTGGTATA** 1746

**Hoya liangii OL826865.1**  **CGGAA---------TGAAAGGCGTCCATTGTCTAATGGATAGGACAGGGGTCTTCTAAACCTTTGGTATA** 1825

**Hoya lithophytica MW719058.1**  **CGGAA---------TGAAAAGCGTCCATTGTCTAATGGATAGGACAGGGGTCTTCTAAACCTTTGGTATA** 1889

**Hoya lockii OR475243.1**  **CGGAA---------TGAAAAGCGTCCATTGTCTAATGGATAGGACAGGGGTCTTCTAAACCTTTGGTATA** 1936

**Hoya longifolia NC_069560.1**  **CGGAA---------TGAAAGGCGTCCATTGTCTAATGGATAGGACAGGGGTCTTCTAAACCTTTGGTATA** 1841

**Hoya lyi MW719055.1**  **CGGAA---------TGAAAAGCGTCCATTGTCTAATGGATAGGACAGGGGTCTTCTAAACCTTTGGTATA** 1935

**Hoya megalaster MW719063.1**  **CGGAA---------TGAAAAGCGTCCATTGTCTAATGGATAGGACAGGGGTCTTCTAAACCTTTGGTATA** 1963

**Hoya meliflua NC_069571.1**  **CGGAA---------TGAAAGGCGTCCATTGTCTAATGGATAGGACAGGGGTCTTCTAAACCTTTGGTATA** 1821

**Hoya monetteae MW719053.1**  **CGGAA---------TGAAAAGCGTCCATTGTCTAATGGATAGGACAGGGGTCTTCTAAACCTTTGGTATA** 1899

**Hoya omlorii MW719060.1**  **CGGAA---------TGAAAAGCGTCCATTGTCTAATGGATAGGACAGGGGTCTTCTAAACCTTTGGTATA** 1775

**Hoya ovalifolia NC_069563.1**  **CGGAA---------TGAAAAGCGTCCATTGTCTAATGGATAGGACAGGGGTCTTCTAAACCTTTGGTATA** 1717

**Hoya pandurata NC_069562.1**  **CGGAA---------TGAAAAGCGTCCATTGTCTAATGGATAGGACAGGGGTCTTCTAAACCTTTGGTATA** 1706

**Hoya pottsii OL754664.1**  **CGGAA---------TGAAAAGCGTCCATTGTCTAATGGATAGGACAGGGGTCTTCTAAACCTTTGGTATA** 1724

**Hoya pubicalyx NC_069561.1**  **CGGAA---------TGAAAAGCGTCCATTGTCTAATGGATAGGACAGGGGTCTTCTAAACCTTTGGTATA** 1894

**Hoya radicalis NC_067961.1**  **CGGAA---------TGAAAAGCGTCCATTGTCTAATGGATAGGACAGGGGTCTTCTAAACCTTTGGTATA** 1698

**Hoya rigida NC_067962.1**  **CGGAA---------TGAAAAGCGTCCATTGTCTAATGGATAGGACAGGGGTCTTCTAAACCTTTGGTATA** 1858

**Hoya silvatica NC_067963.1**  **CGGAA---------TGAAAAGCGTCCATTGTCTAATGGATAGGACAGGGGTCTTCTAAACCTTTGGTATA** 1724

**Hoya thomsonii NC_067612.1**  **CGGAA---------TGAAAAGCGTCCATTGTCTAATGGATAGGACAGGGGTCTTCTAAACCTTTGGTATA** 1809

**Hoya verticillata NC_085236.1**  **CGGAA---------TGAAAAGCGTCCATTGTCTAATGGATAGGACAGGGGTCTTCTAAACCTTTGGTATA** 1711

**Papuahoya urniflora MW719062.1** **CGGAA---------TGAAAAGCGTCCATTGTCTAATGGATAGGACAGGGGTCTTCTAAACCTTTGGTATA** 1909

**Stephanotis volubilis OP133576** **CGGAA---------TGAAAAGCGTCCATTGTCTAATGGATAGGACAGAGGTCTTCTAAACCTTTGGTATA** 1754

**2460 2470 2480 2490 2500 2510 2520**

**....|....|....|....|....|....|....|....|....|....|....|....|....|....|**

**Dischidia australis NC_067885.** **GGTTCAAATCCTATTGGACGCAAATTTTTTCCATATAT-CTAGTTTTTTAT--ATTTT--GATACCACGA** 1864

**Gymnema yunnanense NC_079598.1** **GGTTCAAATCCTATTGGACGCAAATTTTTTCCATATAT-CTATTTTTTTAT--ATTTT--GATACCACGA** 1866

**Hoya ariadna NC_069568.1**  **GGTTCAAATCCTATTGGACGCAAATTTTTTCCATATAT-CTATTTTTTTAT--ATTTT--GATACCACGA** 1851

**Hoya commutata NC_067958.1**  **GGTTCAAATCCTATTGGACGCAAATTTTTTCCATATAT-CTATTTTTTTCT--ATTTT--GATACCACGA** 1856

**Hoya dimorpha NC_067959.1**  **GGTTCAAATCCTATTGGACGCAAATTTTTTCCATATAT-CTATTTTTTTTTT-ATTTT--GATACCACGA** 1997

**Hoya exilis MW719054.1**  **GGTTCAAATCCTATTGGACGCAAATTTTTTCCATATAT-CTATTTTTTTTTTTATTTT--GATACCACGA** 2300

**Hoya griffithii NC_069565.1**  **GGTTCAAATCCTATTGGACGCAAATTTTTTCCATATAT-CTATTTTTTTAT--ATTTT--GATACCACGA** 1926

**Hoya kerrii NC_069570.1**  **GGTTCAAATCCTATTGGACGCAAATTTTTTCCATATAT-CTCTTTTTTTCT--ATTTT--GATACCACGA** 1879

**Hoya lacunosa NC_069564.1**  **GGTTCAAATCCTATTGGACGCAAATTTTTTCCATATATATTTTTTTTTTCT--ATTTT--GATACCACGA** 1777

**Hoya lanceolata NC_067960.1**  **GGTTCAAATCCTATTGGACGCAAATTTTTTCCATATAT-CTATTTTTTTATA-TTTT---GATACCACGA** 1811

**Hoya liangii OL826865.1**  **GGTTCAAATCCTATTGGACGCAAATTTTTTCCATATAT-CTATTTTTTTAT--ATTTT--GATACCACGA** 1890

**Hoya lithophytica MW719058.1**  **GGTTCAAATCCTATTGGACGCAAATTTTTTCCATATAT-CTATTTTTTTAT--ATTTT--GATACCACGA** 1954

**Hoya lockii OR475243.1**  **GGTTCAAATCCTATTGGACGCAAATTTTTTCCATATAT-CTATTTTTTTTTT-TTTTTTTGATACCACGA** 2004

**Hoya longifolia NC_069560.1**  **GGTTCAAATCCTATTGGACGCAAATTTTTTCCATATAT-CTATTTTTTTAT--ATTTT--GATACCACGA** 1906

**Hoya lyi MW719055.1**  **GGTTCAAATCCTATTGGACGCAAATTTTTTCCATATAT-CTATTTTTTTCT--ATTTT--GATACCACGA** 2000

**Hoya megalaster MW719063.1**  **GGTTCAAATCCTATTGGACGCAAATTTTTTCCATATAT-CTATTTTTTTTT--ATTTT--GATACCACGA** 2028

**Hoya meliflua NC_069571.1**  **GGTTCAAATCCTATTGGACGCAAATTTTTTCCATATAT-CTCTTTTTTTCT--ATTTT--GATACCACGA** 1886

**Hoya monetteae MW719053.1**  **GGTTCAAATCCTATTGGACGCAAATTTTTTCCATATAT-CTTTTTTTTTCT--ATTTT--GATACCACGA** 1964

**Hoya omlorii MW719060.1**  **GGTTCAAATCCTATTGGACGCAAATTTTTTCCATATAT-CTATTTTTTTAT--ATTTT--GATACCACGA** 1840

**Hoya ovalifolia NC_069563.1**  **GGTTCAAATCCTATTGGACGCAAATTTTTTCCATATAT-CTATTTTTTTCT--ATTTT--GATACCACGA** 1782

**Hoya pandurata NC_069562.1**  **GGTTCAAATCCTATTGGACGCAAATTTTTTCCATATAT-CTATTTTTTTCT--ATTTT--GATACCACGA** 1771

**Hoya pottsii OL754664.1**  **GGTTCAAATCCTATTGGACGCAAATTTTTTCCATATAT-CTATTTTTTTCT--ATTTT--GATACCACGA** 1789

**Hoya pubicalyx NC_069561.1**  **GGTTCAAATCCTATTGGACGCAAATTTTTTCCATATAT-CTATTTTTTTAT--ATTTT--GATACCACGA** 1959

**Hoya radicalis NC_067961.1**  **GGTTCAAATCCTATTGGACGCAAATTTTTTCCATATAT-CTATTTTTTTCT--ATTTT--GATACCACGA** 1763

**Hoya rigida NC_067962.1**  **GGTTCAAATCCTATTGGACGCAAATTTTTTCCATATAT-CTATTTTTTTCT--ATTTT--GATACCACGA** 1923

**Hoya silvatica NC_067963.1**  **GGTTCAAATCCTATTGGACGCAAATTTTTTCCATATAT-CTATTTTTTTCT--ATTTT--GATACCACGA** 1789

**Hoya thomsonii NC_067612.1**  **GGTTCAAATCCTATTGGACGCAAATTTTTTCCATATAT-CTATTTTTTTCT--ATTTT--GATACCACGA** 1874

**Hoya verticillata NC_085236.1**  **GGTTCAAATCCTATTGGACGCAAATTTTTTCCATATAT-CTATTTTTTTAT--ATTTT--GATACCACGA** 1776

**Papuahoya urniflora MW719062.1** **GGTTCAAATCCTATTGGACGCAAATTTTTTCCATATAT-CCATTTTTTTTT--ATTTT--GATACCACGA** 1974

**Stephanotis volubilis OP133576** **GGTTCAAATCCTATTGGACGCAAATTTTTTCCATATAT-CTATTTTTTTAT--ATTTT--GATACCACGA** 1819

**2530 2540 2550 2560 2570 2580 2590**

**....|....|....|....|....|....|....|....|....|....|....|....|....|....|**

**Dischidia australis NC_067885.** **AAGCCTTTTCGAATAACTTGAATTTGAGTGAATTTGAGACCCTTAA-----TTACATTACCATTACCTTT** 1929

**Gymnema yunnanense NC_079598.1** **AAGCCTTTTCGAGTAACTTGAATTTGAGTGAATTTGAGACCCTTAA-----TTACATTACCATTACCTTT** 1931

**Hoya ariadna NC_069568.1**  **AAGCCTTTTCGAATAACTTTAATTTGAGTGAATTTGAGACCCTTAA-----TTACATTACCATTACCTTT** 1916

**Hoya commutata NC_067958.1**  **AAGCCTTTTCGAATAACTTGAATTTGAGTGGATTTGAGACCCTTAA-----TTACATTACCATTACCTTT** 1921

**Hoya dimorpha NC_067959.1**  **AAGCCTTTTCGAATAACTTGAATTTGAGTGAATTTGAGACCCTTAA-----TTACATTACCATTACCTTT** 2062

**Hoya exilis MW719054.1**  **AAGCCTTTTCGAATAACTTGAATTTGAGTGAATTTGAGACCCTTAA-----TTACATTACCATTACCTTT** 2365

**Hoya griffithii NC_069565.1**  **AAGCCTTTTCGAATAACTTGAATTTGAGTGGATTTGAGACCCTTAA-----TTACATTACCATTACCTTT** 1991

**Hoya kerrii NC_069570.1**  **AAGCCTTTTCGAATAACTTGAATTTGAGTGGATTTGAGACCCTTAA-----TTACATTACCATTACCTTT** 1944

**Hoya lacunosa NC_069564.1**  **AAGCCTTTTCGAATAACTTGAATTTGAGTGGATTTGAGACCCTTAA-----TTACATTACCATTACCCTT** 1842

**Hoya lanceolata NC_067960.1**  **AAGCCTTTTCGAATAACTTGAATTTGAGTGAATTTGAGACCCTTAA-----TTACATTACCATTACCTTT** 1876

**Hoya liangii OL826865.1**  **AAGCCTTTTCGAATAACTTGAATTTGAGTGGATTTGAGACCCTTAA-----TTACATTACCATTACCTTT** 1955

**Hoya lithophytica MW719058.1**  **AAGCCTTTTCGAATAACTTGAATTTGAGTGAATTTGAGACCCTTAA-----TTACATTACCATTACCTTT** 2019

**Hoya lockii OR475243.1**  **AAGCCTTTTCGAATAACTTGAATTTGAGTGAATTTGAGACCCTTAA-----TTACATTACCATTACCTTT** 2069

**Hoya longifolia NC_069560.1**  **AAGCCTTTTCGAATAACTTGAATTTGAGTGGATTTGAGACCCTTAA-----TTACATTACCATTACCTTT** 1971

**Hoya lyi MW719055.1**  **AAGCCTTTTCGAATAACTTGAATTTGAGTGGATTTGAGACCCTTAA-----TTACATTACCATTACCTTT** 2065

**Hoya megalaster MW719063.1**  **AAGCCTTTTCGAATAACTTGAATTTGAGTGAATTTGAGACCCTTAA-----TTACATTACCATTACCTTT** 2093

**Hoya meliflua NC_069571.1**  **AAGCCTTTTCGAATAACTTGAATTTGAGTGGATTTGAGACCCTTAA-----TTACATTACCATTACCTTT** 1951

**Hoya monetteae MW719053.1**  **AAGCCTTTTCGAATAACTTGAATTTGAGTGGATTTGAGACCCTTAA-----TTACATTACCATTACCTTT** 2029

**Hoya omlorii MW719060.1**  **AAGCCTTTTCGAATAACTTGAATTTGAGTGAATTTGAGACCCTTAA-----TTACATTACCATTACCTTT** 1905

**Hoya ovalifolia NC_069563.1**  **AAGCCTTTTTGAATAACTTGAATTTGAGTGGATTTGAGACCCTTAA-----TTACATTACCATTACCTTT** 1847

**Hoya pandurata NC_069562.1**  **AAGCCTTTTCGAATAACTTGAATTTGAGTGGATTTGAGACCCTTAA-----TTACATTACCATTACCTTT** 1836

**Hoya pottsii OL754664.1**  **AAGCCTTTTCGAATAACTTGAATTTGAGTGGATTTGAGACCCTTAA-----TTACATTACCATTACCTTT** 1854

**Hoya pubicalyx NC_069561.1**  **AAGCCTTTTCGAATAACTTGAATTTGAGTGGATTTGAGACCCTTAA-----TTACATTACCATTACCTTT** 2024

**Hoya radicalis NC_067961.1**  **AAGCCTTTTCGAATAACTTGAATTTGAGTGGATTTGAGACCCTTAA-----TTACATTACCATTACCTTT** 1828

**Hoya rigida NC_067962.1**  **AAGCCTTTTCGAATAACTTGAATTTGAGTGGATTTGAGACCCTTAACTTAATTACATTACCATTACCTTT** 1993

**Hoya silvatica NC_067963.1**  **AAGCCTTTTCGAATAACTTGAATTTGAGTGGATTTGAGACCCTTAA-----TTACATTACCATTACCTTT** 1854

**Hoya thomsonii NC_067612.1**  **AAGCCTTTTCGAATAACTTGAATTTGAGTGGATTTGAGACCCTTAA-----TTACATTACCATTACCTTT** 1939

**Hoya verticillata NC_085236.1**  **AAGCCTTTTTGAATAACTTGAATTTGAGTGGATTTGAGACCCTTAA-----TTACATTACCATTACCTTT** 1841

**Papuahoya urniflora MW719062.1** **AAGCCTTTTCGAATAACTT----------GAATTTGAGACCCTTAA-----TTACATTACCATTACCTTT** 2029

**Stephanotis volubilis OP133576** **AAGCCTTTTCGAGTAACTTGAATTTGAG----------ACCCTTAA-----TTACATTACCATTACC---** 1871

**2600 2610 2620 2630 2640 2650**

**....|....|....|....|....|....|....|....|....|....|....|....|....|...**

**Dischidia australis NC_067885.** **TTA--AGAT--------------------AAATTTAAGATAAAAGAA---------TCCTTTTTGTGC** 1966

**Gymnema yunnanense NC_079598.1** **TAATTACATTACCATTACCTTTTTAAGATAAATTTAAGGTAAAAGAAAGTAAGTGATCCTTTTTGTGC** 1999

**Hoya ariadna NC_069568.1**  **TTA--AGAA--------------------AAATTTAAGATAAAAGAA---------TCCTTTTTGTGC** 1953

**Hoya commutata NC_067958.1**  **TTA--AGAT--------------------AAATTTAAGATAAAAGAA---------TCCTTTTTGTGC** 1958

**Hoya dimorpha NC_067959.1**  **TTA--AGAA--------------------AAATTTAAGATAAAAGAA---------TCCTTTTTGTGC** 2099

**Hoya exilis MW719054.1**  **TTA--AGAT--------------------AAATTTAAGATAAAAGAA---------TCCTTTTTGTGC** 2402

**Hoya griffithii NC_069565.1**  **TTA--AGAT--------------------AAATTTAAGATAAAAGAA---------TCCTTTTTGTGC** 2028

**Hoya kerrii NC_069570.1**  **TTA--AGAT--------------------AAATTTAAGATAAAAGAA---------TCCTTTTTGTGC** 1981

**Hoya lacunosa NC_069564.1**  **TTA--AGAT--------------------AAATTTAAGATAAAAGAA---------TCCTTTTTGTGC** 1879

**Hoya lanceolata NC_067960.1**  **TTA--AGAT--------------------AAATTTAAGATAAAAGAA---------TCCTTTTTGTGC** 1913

**Hoya liangii OL826865.1**  **TTA--AGAT--------------------AAATTTAAGATAAAAGAA---------TCCTTTTTGTGC** 1992

**Hoya lithophytica MW719058.1**  **TTA--AGAT--------------------AAATTTAAGATAAAAGAA---------TCCTTTTTGTGC** 2056

**Hoya lockii OR475243.1**  **TTA--AGAT--------------------AAATTTAAGATAAAAGAA---------TCCTTTTTGTGC** 2106

**Hoya longifolia NC_069560.1**  **TTA--AGAT--------------------AAATTTAAGATAAAAGAA---------TCCTTTTTGTGC** 2008

**Hoya lyi MW719055.1**  **TTA--AGAT--------------------AAATTTAAGATAAAAGAA---------TTCTTTTTGTGC** 2102

**Hoya megalaster MW719063.1**  **TTA--AGAA--------------------AAATTTAAGATAAAAGAA---------TCCTTTTTGTGC** 2130

**Hoya meliflua NC_069571.1**  **TTA--AGAT--------------------AAATTTAAGATAAAAGAA---------TCCTTTTTGTGC** 1988

**Hoya monetteae MW719053.1**  **TTA--AGAT--------------------AAATTTAAGATAAAAGAA---------TCCTTTTTGTGC** 2066

**Hoya omlorii MW719060.1**  **TTA--ATAA--------------------AAATTTAAGATAAAAGAA---------TCCTTTTTGTGC** 1942

**Hoya ovalifolia NC_069563.1**  **TTA--AGATAAATTTAAGATAAATAAGATAAATTTAAGATAAAAGAA---------TCCTTTTTGTGC** 1904

**Hoya pandurata NC_069562.1**  **TTA--AGAT--------------------AAATTTAAGATAAAAGAA---------TCCTTTTTGTGC** 1873

**Hoya pottsii OL754664.1**  **TTA--AGAT--------------------AAATTTAAGATAAAAGAA---------TCCTTTTTGTGC** 1891

**Hoya pubicalyx NC_069561.1**  **TTA--AGAT--------------------AAATTTAAGATAAAAGAA---------TCCTTTTTGTGC** 2061

**Hoya radicalis NC_067961.1**  **TTA--AGAT--------------------AAATTTAAGATAAAAGAA---------TCCTTTTTGTGC** 1865

**Hoya rigida NC_067962.1**  **TTA--AGAT--------------------AAATTTAAGATAAAAGAA---------TCCTTTTTGTGC** 2030

**Hoya silvatica NC_067963.1**  **TTA--AGAT--------------------AAATTTAAGATAAAAGAA---------TCCTTTTTGTGC** 1891

**Hoya thomsonii NC_067612.1**  **TTA--AGAT--------------------AAATTTAAGATAAAAGAA---------TCCTTTTTGTGC** 1976

**Hoya verticillata NC_085236.1**  **TTA--AGAT--------------------AAATTTAAGATAAAAGAA---------TCCTTTTTGTGC** 1878

**Papuahoya urniflora MW719062.1** **TTA--AGAT--------------------AAATTTAAGATAAAAGAA---------TCCTTTTTGTGC** 2066

**Stephanotis volubilis OP133576** **------------------CTTTTTAAGATAAATTTAAGATAAAAGAAAGTAAGTGATCCCTTTTGTGC** 1921

**Figure 3.** Alignment results of 30 *psbK-psbI* sequences from the studied species

**10 20 30 40 50 60 70**

**....|....|....|....|....|....|....|....|....|....|....|....|....|....|**

**Dischidia australis NC_067885.** **GATCCTTAATCTAAAAGATATAAAAAAGAAATTATTTAA------------------------------A** 40

**Gymnema yunnanense NC_079598.1** **GATCCTTAATCTAAAATC------------AAAATT---------------------------ATTTAAA** 31

**Hoya ariadna NC_069568.1**  **GATCCTTAATCTAAAATATCTAAAATCTAAATTATTTAA------------------TAAATTATTTAAA** 52

**Hoya commutata NC_067958.1**  **GATCCTTAATCTAAAATATCTAAAATCTAAATTATTTAA------------------TAAATTATTTAAA** 52

**Hoya dimorpha NC_067959.1**  **GATCCTTAATCTAAAATATCTAAAATCTAAATTATTTAA------------------TAAATTATTTAAA** 52

**Hoya exilis MW719054.1**  **GATCCTTAATCTAAAATATCTAAAATCAAAATTATTTAAAAAATTAAAATTATTTAATAAATTATTTAAA** 70

**Hoya griffithii NC_069565.1**  **GATCCTTAATCTAAAATATCTAAAATATCTAAAATCTAAATT---------ATTTAATAAATTATTTAAA** 61

**Hoya kerrii NC_069570.1**  **GATCCTTAATCTAAAATATCTAAAATCTAAATTATTTAATAA------ATAATTTAA------------A** 52

**Hoya lacunosa NC_069564.1**  **GATCCTTAATCTAAAATATCTAAAATCTAAATTATTAAA------------------TAAATTATTTAAA** 52

**Hoya lanceolata NC_067960.1**  **GATCCTTAATCTAAAA-ATCTAAAATATCTAAAATCTAAATT---------ATTTAATAAATTATTTAAA** 60

**Hoya liangii OL826865.1**  **GATCCTTAATCTAAAATATCTAAAATCTAAATTATTTAA------------------TAAATTATTTAAA** 52

**Hoya lithophytica MW719058.1**  **GATCCTTAATCTAAAATATCTAAAATCTAAATTATTTAA------------------TAAATTATTTAAA** 52

**Hoya lockii OR475243.1**  **GATCCTTAATCTAAAATATCTAAAATAGAAATTTTTTAA------------------------------A** 40

**Hoya longifolia NC_069560.1**  **GATCCTTAATCTAAAATATCTAAAATATCTAAAATCTAAATT---------ATTTAATAAATTATTTAAA** 61

**Hoya lyi MW719055.1**  **GATCCTTAATCTAAAATATCTAAAATC------------------------------TAAATTATTTAAA** 40

**Hoya megalaster MW719063.1**  **GATCCTTAATCTAAAATATCTAAAATCTAAATTATTTAA------------------TAAATTATTTAAA** 52

**Hoya meliflua NC_069571.1**  **GATCCTTAATCTAAAATATCTAAAATATAAATTATTTAA------------------------------A** 40

**Hoya monetteae MW719053.1**  **GATCCTTAATCTAAAATATCTAAAATCTAAATTATTTAA------------------TAAATTATTTAAA** 52

**Hoya omlorii MW719060.1**  **GATCCTTAATCTAAAATATCTAAAATCTAAATTATTTAA------------------TAAATTATTTAAA** 52

**Hoya ovalifolia NC_069563.1**  **GATCCTTAATCTAAAATATCTAAAATCTAAATTATTTAA------------------TAAATTATTTAAA** 52

**Hoya pandurata NC_069562.1**  **GATCCTTAATCTAAAATATCTAAAATCTAAATTATTTAA------------------------------A** 40

**Hoya pottsii OL754664.1**  **GATCCTTAATCTAAAATATCTAAAATCTAAATTATT------------------------------TAAA** 40

**Hoya pubicalyx NC_069561.1**  **GATCCTTAATCTAAAATATCTAAAATCTAAATTATTTAATAT------ATTATTTAATATATTATTTAAA** 64

**Hoya radicalis NC_067961.1**  **GATCCTTAATCTAAAATATCTAAAATCTAAATT------------------------------ATTTAAA** 40

**Hoya rigida NC_067962.1**  **GATCCTTAATCTAAAATCTAAATTATTTAAAAAATT---------------------------ATTTAAA** 43

**Hoya silvatica NC_067963.1**  **GATCCTTAATCTAAAATATCTAAAATCTAAATTATT------------------------------TAAA** 40

**Hoya thomsonii NC_067612.1**  **GATCCTTAATCTAAAATATCTAAAATCTAAATTTTTT------------------AATAAATTATTTAAA** 52

**Hoya verticillata NC_085236.1**  **GATCCTTAATCTAAAATATCTAAAATCTAAATTATTTAA------------------TAAATTATTTAAA** 52

**Papuahoya urniflora MW719062.1** **GATCCTTAATCTAAAATATCTAAAATCTAAATTATTTAA------------------------------A** 40

**Stephanotis volubilis OP133576** **GATCCTTAATCTAAAATC------------TAAATT---------------------------ATTTTAA** 31

**80 90 100 110 120 130 140**

**....|....|....|....|....|....|....|....|....|....|....|....|....|....|**

**Dischidia australis NC_067885.** **TTGAAAAATTTCTTACAAA-TTTCCTAGATTTCCTCGAAAGTTCGTGATTTTTTCTAGAAAGAAACTCGG** 109

**Gymnema yunnanense NC_079598.1** **TTGAATAATTTCTTACTAA-TTTCCTAGATTTCCTCGAAAGTTCGTGATTTTTTCTAGAAAAAAACTCGG** 100

**Hoya ariadna NC_069568.1**  **TTGAAAAATTTCTTACAAA-TTTCCTAGATTTCCTCGAAAGTTCGTGATTTTTTCTAGAAAGAAACTCGG** 121

**Hoya commutata NC_067958.1**  **TTGAAAAATTTCTTAAAAA-TTTCCTAGATTTCCTCGAAAGTTCGTGATTTTTTCTAGAAAGAAACTCGG** 121

**Hoya dimorpha NC_067959.1**  **TTGAAAAATTTCTTACAAA-TTTCCTAGATTTCCTCGAAAGTTCGTGATTTTTTCTAGAAAGAAACTCGG** 121

**Hoya exilis MW719054.1**  **TTGAAAAATTTCTTACAAA-TTTCCTAGATTTCCTCGAAAGTTCGTGATTTTTTCTAGAAAGAAACTCGG** 139

**Hoya griffithii NC_069565.1**  **TTGAAAAATTTCTTACAAA-TTTCCTAGATTTCCTCGAAAGTTCGTGATTTTTTCTAGAAAGAAACTCGG** 130

**Hoya kerrii NC_069570.1**  **TTGAAAAATTTCTTACAAA-TTTGCTAGATTTCCTCGAAAGTTCGTGATTTTTTCTAGAAAGAAACTCGG** 121

**Hoya lacunosa NC_069564.1**  **TTGAAAAATTTCTTAAAAA-TTTCCTAGATTTCCTCGAAAGTTCGTGATTTTTTCTAGAAAGAAACTCGG** 121

**Hoya lanceolata NC_067960.1**  **TTGAAAAATTTCTTACAAA-TTTCCTAGATTTCCTCGAAAGTTCGTGATTTTTTCTAGAAAGAAACTCGG** 129

**Hoya liangii OL826865.1**  **TTGAAAAATTTCTTACAAA-TTTCCTAGATTTCCTCGAAAGTTCGTGATTTTTTCTAGAAAGAAACTCGG** 121

**Hoya lithophytica MW719058.1**  **TTGAAAAATTTCTTACAAA-TTTCCTAGATTTCCTCGAAAGTTCGTGATTTTTTCTAGAAAGAAACTCGG** 121

**Hoya lockii OR475243.1**  **TTTAAAAATTTCTTACAAA-TTTCCTAGATTTCCTCGAAAGTTCGTGATTTTTTCTAGAAAGAAACTCGG** 109

**Hoya longifolia NC_069560.1**  **TTGAAAAATTTCTTACAAA-TTTCCTAGATTTCCTCGAAAGTTCGTGATTTTTTCTAGAAAGAAACTCGG** 130

**Hoya lyi MW719055.1**  **TTGAAAAATTTCTTACAAA-TTTCCTAGATTTCCTCGAAAGTTCGTTATTTTTTCTAGAAAGAAACTCGG** 109

**Hoya megalaster MW719063.1**  **TTGAAAAATTTCTTACAAA-TTTCCTAGATTTCCTCGAAAGTTCGTGATTTTTTCTAGAAAGAAACTAGG** 121

**Hoya meliflua NC_069571.1**  **TTGAAAAATTTCTTACAAA-TTTCCTAGATTTCCTCGAAAGTTCGTGATTTTTTCTAGAAAGAAACTCGG** 109

**Hoya monetteae MW719053.1**  **TTGAAAAATTTCTTACAAA-TTTCCTAGATTTCCTCGAAAGTTCGTGATTTTTTCTAGAAAGAAACTCGG** 121

**Hoya omlorii MW719060.1**  **TTGAAAAATTTCTTACAAA-TTTCCTAGATTTCCTCGAAAGTTCGTGATTTTTTCTAGAAAGAAACTCGG** 121

**Hoya ovalifolia NC_069563.1**  **TTGAAAAATTTCTTACAAA-TTTCCTAGATTTCCTCGAAAGTTCGTGATTTTTTCTAGAAAGAAACTCGG** 121

**Hoya pandurata NC_069562.1**  **TTGAAAAATTTCTTACAAA-TTTTCTAGATTTCCTCGAAAGTTCGTGATTTTTTCTAGAAAGAAACTCGG** 109

**Hoya pottsii OL754664.1**  **TTGAAAAATTTCTTAAAAAATTTCCTAGATTTCCTCGAAAGTTCGTGATTTTTTCTAGAAAGAAACTCGG** 110

**Hoya pubicalyx NC_069561.1**  **TTGAAAAATTTCTTACAAA-TTTCCTAGATTTCCTCGAAAGTTCGTGATTTTTTCTAGAAAGAAACTCGG** 133

**Hoya radicalis NC_067961.1**  **TTGAAAAATTTCTTACAAA-TTTCCTAGATTTCCTCGAAAGTTCGTGATTTTTTCTAGAAAGAAACTCGG** 109

**Hoya rigida NC_067962.1**  **TTGAAAAATTTCTTACAAA-TTTCCTAGATTTCCTCGAAAGTTCATGATTTTTTCTAGAAAGAAACTCGG** 112

**Hoya silvatica NC_067963.1**  **TTGAAAAATTTCTTAAAAAATTTCCTAGATTTCCTCGAAAGTTCGTGATTTTTTCTAGAAAGAAACTCGG** 110

**Hoya thomsonii NC_067612.1**  **TTGAAAAATTTCTTACAAA-TTTCCTAGATTTCCTCGAAAGTTCGTGATTTTTTCTAGAAAGAAACTCGG** 121

**Hoya verticillata NC_085236.1**  **TTGAAAAATTTCTTACAAA-TTTCCTAGATTTCCTCGAAAGTTCGTGATTTTTTCTAGAAAGAAACTCGG** 121

**Papuahoya urniflora MW719062.1** **TTGAAAAATTTCTTACAAA-TTTCCTAGATTTCCTCGAAAGTTCGTGATTTTTTCTAGAAAGAAACTCGG** 109

**Stephanotis volubilis OP133576** **TTGAATAATTTCTTACTAA-TTTCCTAGATTTCCTCGAAAGTTCGTGATTTTTTCTAGAAAGAAACTCGG** 100

**150 160 170 180 190 200 210**

**....|....|....|....|....|....|....|....|....|....|....|....|....|....|**

**Dischidia australis NC_067885.** **CTCTTGATATCCAAATAGGATATGTGGTAGAAAAATGGAGGATCTATTCTCTTTTTTTTTTTAATTATCT** 179

**Gymnema yunnanense NC_079598.1** **TTCTTGATATCCAAATAGGATATGTGGTAGAAAAATGGAGGATCTATTCTCTTTTTTTTTTTAATTATCT** 170

**Hoya ariadna NC_069568.1**  **CTCTTGATATCCAAATAGGATATGTGGTAGAAAAATGGAGGATCTATTCTCTTTTTTTTTT-AATTATCT** 190

**Hoya commutata NC_067958.1**  **CTCTTGATATCCAAATAGGATATGTGGTAGAAAAATGGAGGATCTATTCTCTTTTTTTTTTTAATTATCT** 191

**Hoya dimorpha NC_067959.1**  **CTCTTGATATCCAAATAGGATATGTGGTAGAAAAATGGAGGATCTATTCTCTTTTTTTTTT-AATTATCT** 190

**Hoya exilis MW719054.1**  **CTCTTGATATCCAAATAGGATATGTGGTAGAAAAATGGAGGATCTATTCTCTTTTTTTTTT-AATTATCT** 208

**Hoya griffithii NC_069565.1**  **CTCTTGATATCCAAATAGGATATGTGGTAGAAAAATGGAGGATCTATTCTCTTTTTTTTTT-AATTATCT** 199

**Hoya kerrii NC_069570.1**  **CTCTTGATATCCAAATAGGATATGTGGTAGAAAAATGGAGGATCTATTCTCTTTTTTTTTTTAATTATCT** 191

**Hoya lacunosa NC_069564.1**  **CTCTTGATATCCAAATAGGATATGTGGTAGAAAAATGGAGGATCTATTCCCTTTTTTTTTTTAATTATCT** 191

**Hoya lanceolata NC_067960.1**  **CTCTTGATATCCAAATAGGATATGTGGTAGAAAAATGGAGGATCTATTC--TTTTTTTTTT-AATTATCT** 196

**Hoya liangii OL826865.1**  **CTCTTGATATCCAAATAGGATATGTGGTAGAAAAATGGAGGATCTATTCTCTTTTTTTTTT-AATTATCT** 190

**Hoya lithophytica MW719058.1**  **CTCTTGATATCCAAATAGGATATGTGGTAGAAAAATGGAGGATCTATTCTCTTTTTTTTTTTAATTATCT** 191

**Hoya lockii OR475243.1**  **CTCTTGATATCCAAATAGGATATGTGGTAGAAAAATGGAGGATCTATTCTCTTTTTTTTT--AATTATCT** 177

**Hoya longifolia NC_069560.1**  **CTCTTGATATCCAAATAGGATATGTGGTAGAAAAATGGAGGATCTATTCTCTTTTTTTTTT-AATTATCT** 199

**Hoya lyi MW719055.1**  **CTCTTGATATCCAAATAGGATATGTGGTAGAAAAATGGAGGATCTATTCTCTTTTTTTTTT-AATTATCT** 178

**Hoya megalaster MW719063.1**  **CTCTTGATATCCAAATAGGATATGTGGTAGAAAAATGGAGGATCTATTCTCTTTTTTTTTT-AATTATCT** 190

**Hoya meliflua NC_069571.1**  **CTCTTGATATCCAAATAGGATATGTGGTAGAAAAATGGAGGATCTATTCTCTTTTTTTTTTTAATTATCT** 179

**Hoya monetteae MW719053.1**  **CTCTTGATATCCAAATAGGATATGTGGTAGAAAAATGGAGGATCTATTCTCTTTTTTTTTTTAATTATCT** 191

**Hoya omlorii MW719060.1**  **CTCTTGATATCCAAATAGGATATGTGGTAGAAAAATAGAGGATCTATTCTCTTTTTTTTTT-AATTATCT** 190

**Hoya ovalifolia NC_069563.1**  **CTCTTGATATCCAAATAGGATATGTGGTAGAAAAATGGAGGATCTATTCTCTTTTTTTTTTTAATTATCT** 191

**Hoya pandurata NC_069562.1**  **CTCTTGATATCCAAATAGGATATGTGGTAGAAAAATGGAGGATCTATTCTCTTTTTTTTT--AATTATCT** 177

**Hoya pottsii OL754664.1**  **CTCTTGATATCCAAATAGGATATGTGGTAGAAAAATGGAGGATCTATTCTCTTTTTTTTTTTAATTATCT** 180

**Hoya pubicalyx NC_069561.1**  **CTCTTGATATCCAAATAGGATATGTGGTAGAAAAATGGAGGATCTATTCTCTTTTTTTTTT-AATTATCT** 202

**Hoya radicalis NC_067961.1**  **CTCTTGATATCCAAATAGGATATGTGGTAGAAAAATGGAGGATCTATTCTCTTTTTTTTTT-AATTATCT** 178

**Hoya rigida NC_067962.1**  **CTCTTGATATCCAAATAGGATATGTGGTAGAAAAATGGAGGATCTATTCTCTTTTTTTTTT-AATTATCT** 181

**Hoya silvatica NC_067963.1**  **CTCTTGATATCCAAATAGGATATGTGGTAGAAAAATGGAGGATCTATTCTCTTTTTTTTTTTAATTATCT** 180

**Hoya thomsonii NC_067612.1**  **CTCTTGATATCCAAATAGGATATGTGGTAGAAAAATGGAGGATCTATTCTCTTTTTTTTTT-AATTATCT** 190

**Hoya verticillata NC_085236.1**  **CTCTTGATATCCAAATAGGATATGTGGTAGAAAAATGGAGGATCTATTCTCTTTTTTTTTT-AATTATCT** 190

**Papuahoya urniflora MW719062.1** **CTCTTGATATCCAAATAGGATATGTGGTAGAAAAATGGAGGATCTATTCTCTTTTTTTTTT-AATTATCT** 178

**Stephanotis volubilis OP133576** **TTCTTGATATCCAAATAGGATATGTGGTAGAAAAATGGAGGATCTATTCTCTTTTTTTTTT-AATTATCT** 169

**220**

**....|....|...**

**Dischidia australis NC_067885.** **TGGAGATTGTGTA** 192

**Gymnema yunnanense NC_079598.1** **TGGAGATTGTGTA** 183

**Hoya ariadna NC_069568.1**  **TGGAGATTGTGTA** 203

**Hoya commutata NC_067958.1**  **TGGAGATTGTGTA** 204

**Hoya dimorpha NC_067959.1**  **TGGAGATTGTGTA** 203

**Hoya exilis MW719054.1**  **TGGAGATTGTGTA** 221

**Hoya griffithii NC_069565.1**  **TGGAGATTGTGTA** 212

**Hoya kerrii NC_069570.1**  **TGGAGATTGTGTA** 204

**Hoya lacunosa NC_069564.1**  **TGGAGATTGTGTA** 204

**Hoya lanceolata NC_067960.1**  **TGGAGATTGTGTA** 209

**Hoya liangii OL826865.1**  **TGGAGATTGTGTA** 203

**Hoya lithophytica MW719058.1**  **TGGAGATTGTGTA** 204

**Hoya lockii OR475243.1**  **TGGAGATTGTGTA** 190

**Hoya longifolia NC_069560.1**  **TGGAGATTGTGTA** 212

**Hoya lyi MW719055.1**  **TGGAGATTGTGTA** 191

**Hoya megalaster MW719063.1**  **TGGAGATTGTGTA** 203

**Hoya meliflua NC_069571.1**  **TGGAGATTGTGTA** 192

**Hoya monetteae MW719053.1**  **TGGAGATTGTGTA** 204

**Hoya omlorii MW719060.1**  **TGGAGATTGTGTA** 203

**Hoya ovalifolia NC_069563.1**  **TGGAGATTGTGTA** 204

**Hoya pandurata NC_069562.1**  **TGGAGATTGTGTA** 190

**Hoya pottsii OL754664.1**  **TGGAGATTGTGTA** 193

**Hoya pubicalyx NC_069561.1**  **TGGAGATTGTGTA** 215

**Hoya radicalis NC_067961.1**  **TGGAGATTGTGTA** 191

**Hoya rigida NC_067962.1**  **TGGAGATTGTGTA** 194

**Hoya silvatica NC_067963.1**  **TGGAGATTGTGTA** 193

**Hoya thomsonii NC_067612.1**  **TGGAGATTGTGTA** 203

**Hoya verticillata NC_085236.1**  **TGGAGATTGTGTA** 203

**Papuahoya urniflora MW719062.1** **TGGAGATTGTGTA** 191

**Stephanotis volubilis OP133576** **TGGAGATTGTGTA** 182

## Figure S4. Alignment results of 30 rbcL-accD sequences from the studied species

**10 20 30 40 50 60 70**

**....|....|....|....|....|....|....|....|....|....|....|....|....|....|**

**Dischidia australis NC_067885.** **----------------------------------------------------------------------** 1

**Gymnema yunnanense NC_079598.1** **----------------------------------------------------------------------** 1

**Hoya ariadna NC_069568.1**  **----------------------------------------------------------------------** 1

**Hoya commutata NC_067958.1**  **----------------------------------------------------------------------** 1

**Hoya dimorpha NC_067959.1**  **----------------------------------------------------------------------** 1

**Hoya exilis MW719054.1**  **----------------------------------------------------------------------** 1

**Hoya griffithii NC_069565.1**  **----------------------------------------------------------------------** 1

**Hoya kerrii NC_069570.1**  **----------------------------------------------------------------------** 1

**Hoya lacunosa NC_069564.1**  **----------------------------------------------------------------------** 1

**Hoya lanceolata NC_067960.1**  **----------------------------------------------------------------------** 1

**Hoya liangii OL826865.1**  **----------------------------------------------------------------------** 1

**Hoya lithophytica MW719058.1**  **----------------------------------------------------------------------** 1

**Hoya lockii OR475243.1**  **----------------------------------------------------------------------** 1

**Hoya longifolia NC_069560.1**  **----------------------------------------------------------------------** 1

**Hoya lyi MW719055.1**  **----------------------------------------------------------------------** 1

**Hoya megalaster MW719063.1**  **ATGTCACCACAAACAGAGACTAAAGCAAGTGTTGGATTCAAAGCCGGTGTTAAAGAGTACAAATTGACTT** 70

**Hoya meliflua NC_069571.1**  **----------------------------------------------------------------------** 1

**Hoya monetteae MW719053.1**  **----------------------------------------------------------------------** 1

**Hoya omlorii MW719060.1**  **ATGTCACCACAAACAGAGACTAAAGCAAGTGTTGGATTCAAAGCCGGTGTTAAAGAGTACAAATTGACTT** 70

**Hoya ovalifolia NC_069563.1**  **----------------------------------------------------------------------** 1

**Hoya pandurata NC_069562.1**  **----------------------------------------------------------------------** 1

**Hoya pottsii OL754664.1**  **----------------------------------------------------------------------** 1

**Hoya pubicalyx NC_069561.1**  **----------------------------------------------------------------------** 1

**Hoya radicalis NC_067961.1**  **----------------------------------------------------------------------** 1

**Hoya rigida NC_067962.1**  **----------------------------------------------------------------------** 1

**Hoya silvatica NC_067963.1**  **----------------------------------------------------------------------** 1

**Hoya thomsonii NC_067612.1**  **----------------------------------------------------------------------** 1

**Hoya verticillata NC_085236.1**  **----------------------------------------------------------------------** 1

**Papuahoya urniflora MW719062.1** **----------------------------------------------------------------------** 1

**Stephanotis volubilis OP133576** **----------------------------------------------------------------------** 1

**80 90 100 110 120 130 140**

**....|....|....|....|....|....|....|....|....|....|....|....|....|....|**

**Dischidia australis NC_067885.** **----------------------------------------------------------------------** 1

**Gymnema yunnanense NC_079598.1** **----------------------------------------------------------------------** 1

**Hoya ariadna NC_069568.1**  **----------------------------------------------------------------------** 1

**Hoya commutata NC_067958.1**  **----------------------------------------------------------------------** 1

**Hoya dimorpha NC_067959.1**  **----------------------------------------------------------------------** 1

**Hoya exilis MW719054.1**  **----------------------------------------------------------------------** 1

**Hoya griffithii NC_069565.1**  **----------------------------------------------------------------------** 1

**Hoya kerrii NC_069570.1**  **----------------------------------------------------------------------** 1

**Hoya lacunosa NC_069564.1**  **----------------------------------------------------------------------** 1

**Hoya lanceolata NC_067960.1**  **----------------------------------------------------------------------** 1

**Hoya liangii OL826865.1**  **----------------------------------------------------------------------** 1

**Hoya lithophytica MW719058.1**  **----------------------------------------------------------------------** 1

**Hoya lockii OR475243.1**  **----------------------------------------------------------------------** 1

**Hoya longifolia NC_069560.1**  **----------------------------------------------------------------------** 1

**Hoya lyi MW719055.1**  **----------------------------------------------------------------------** 1

**Hoya megalaster MW719063.1**  **ATTATACTCCTGAATACGAAACAAAAGATACTGATATCTTGGCAGCATTCCGAGTAACTCCTCAACCCGG** 140

**Hoya meliflua NC_069571.1**  **----------------------------------------------------------------------** 1

**Hoya monetteae MW719053.1**  **----------------------------------------------------------------------** 1

**Hoya omlorii MW719060.1**  **ATTATACTCCTGAATACGAAACAAAAGATACTGATATCTTGGCAGCATTCCGAGTAACTCCTCAACCCGG** 140

**Hoya ovalifolia NC_069563.1**  **----------------------------------------------------------------------** 1

**Hoya pandurata NC_069562.1**  **----------------------------------------------------------------------** 1

**Hoya pottsii OL754664.1**  **----------------------------------------------------------------------** 1

**Hoya pubicalyx NC_069561.1**  **----------------------------------------------------------------------** 1

**Hoya radicalis NC_067961.1**  **----------------------------------------------------------------------** 1

**Hoya rigida NC_067962.1**  **----------------------------------------------------------------------** 1

**Hoya silvatica NC_067963.1**  **----------------------------------------------------------------------** 1

**Hoya thomsonii NC_067612.1**  **----------------------------------------------------------------------** 1

**Hoya verticillata NC_085236.1**  **----------------------------------------------------------------------** 1

**Papuahoya urniflora MW719062.1** **----------------------------------------------------------------------** 1

**Stephanotis volubilis OP133576** **----------------------------------------------------------------------** 1

**150 160 170 180 190 200 210**

**....|....|....|....|....|....|....|....|....|....|....|....|....|....|**

**Dischidia australis NC_067885.** **----------------------------------------------------------------------** 1

**Gymnema yunnanense NC_079598.1** **----------------------------------------------------------------------** 1

**Hoya ariadna NC_069568.1**  **----------------------------------------------------------------------** 1

**Hoya commutata NC_067958.1**  **----------------------------------------------------------------------** 1

**Hoya dimorpha NC_067959.1**  **----------------------------------------------------------------------** 1

**Hoya exilis MW719054.1**  **----------------------------------------------------------------------** 1

**Hoya griffithii NC_069565.1**  **----------------------------------------------------------------------** 1

**Hoya kerrii NC_069570.1**  **----------------------------------------------------------------------** 1

**Hoya lacunosa NC_069564.1**  **----------------------------------------------------------------------** 1

**Hoya lanceolata NC_067960.1**  **----------------------------------------------------------------------** 1

**Hoya liangii OL826865.1**  **----------------------------------------------------------------------** 1

**Hoya lithophytica MW719058.1**  **----------------------------------------------------------------------** 1

**Hoya lockii OR475243.1**  **----------------------------------------------------------------------** 1

**Hoya longifolia NC_069560.1**  **----------------------------------------------------------------------** 1

**Hoya lyi MW719055.1**  **----------------------------------------------------------------------** 1

**Hoya megalaster MW719063.1**  **AGTTCCACCCGAAGAAGCAGGGGCCGCGGTAGCTGCCGAATCTTCTACTGGTACATGGACAACTGTTTGG** 210

**Hoya meliflua NC_069571.1**  **----------------------------------------------------------------------** 1

**Hoya monetteae MW719053.1**  **----------------------------------------------------------------------** 1

**Hoya omlorii MW719060.1**  **AGTTCCACCCGAAGAAGCAGGGGCCGCGGTAGCTGCCGAATCTTCTACTGGTACATGGACAACTGTTTGG** 210

**Hoya ovalifolia NC_069563.1**  **----------------------------------------------------------------------** 1

**Hoya pandurata NC_069562.1**  **----------------------------------------------------------------------** 1

**Hoya pottsii OL754664.1**  **----------------------------------------------------------------------** 1

**Hoya pubicalyx NC_069561.1**  **----------------------------------------------------------------------** 1

**Hoya radicalis NC_067961.1**  **----------------------------------------------------------------------** 1

**Hoya rigida NC_067962.1**  **----------------------------------------------------------------------** 1

**Hoya silvatica NC_067963.1**  **----------------------------------------------------------------------** 1

**Hoya thomsonii NC_067612.1**  **----------------------------------------------------------------------** 1

**Hoya verticillata NC_085236.1**  **----------------------------------------------------------------------** 1

**Papuahoya urniflora MW719062.1** **----------------------------------------------------------------------** 1

**Stephanotis volubilis OP133576** **----------------------------------------------------------------------** 1

**220 230 240 250 260 270 280**

**....|....|....|....|....|....|....|....|....|....|....|....|....|....|**

**Dischidia australis NC_067885.** **----------------------------------------------------------------------** 1

**Gymnema yunnanense NC_079598.1** **----------------------------------------------------------------------** 1

**Hoya ariadna NC_069568.1**  **----------------------------------------------------------------------** 1

**Hoya commutata NC_067958.1**  **----------------------------------------------------------------------** 1

**Hoya dimorpha NC_067959.1**  **----------------------------------------------------------------------** 1

**Hoya exilis MW719054.1**  **----------------------------------------------------------------------** 1

**Hoya griffithii NC_069565.1**  **----------------------------------------------------------------------** 1

**Hoya kerrii NC_069570.1**  **----------------------------------------------------------------------** 1

**Hoya lacunosa NC_069564.1**  **----------------------------------------------------------------------** 1

**Hoya lanceolata NC_067960.1**  **----------------------------------------------------------------------** 1

**Hoya liangii OL826865.1**  **----------------------------------------------------------------------** 1

**Hoya lithophytica MW719058.1**  **----------------------------------------------------------------------** 1

**Hoya lockii OR475243.1**  **----------------------------------------------------------------------** 1

**Hoya longifolia NC_069560.1**  **----------------------------------------------------------------------** 1

**Hoya lyi MW719055.1**  **----------------------------------------------------------------------** 1

**Hoya megalaster MW719063.1**  **ACCGATGGACTTACCAGCCTTGATCGTTACAAAGGGCGATGCTACCATATCGAGGCCGTTCCTGGAGAAG** 280

**Hoya meliflua NC_069571.1**  **----------------------------------------------------------------------** 1

**Hoya monetteae MW719053.1**  **----------------------------------------------------------------------** 1

**Hoya omlorii MW719060.1**  **ACCGATGGACTTACCAGCCTTGATCGTTACAAAGGGCGATGCTACCATATCGAGGCCGTTCCTGGAGAAG** 280

**Hoya ovalifolia NC_069563.1**  **----------------------------------------------------------------------** 1

**Hoya pandurata NC_069562.1**  **----------------------------------------------------------------------** 1

**Hoya pottsii OL754664.1**  **----------------------------------------------------------------------** 1

**Hoya pubicalyx NC_069561.1**  **----------------------------------------------------------------------** 1

**Hoya radicalis NC_067961.1**  **----------------------------------------------------------------------** 1

**Hoya rigida NC_067962.1**  **----------------------------------------------------------------------** 1

**Hoya silvatica NC_067963.1**  **----------------------------------------------------------------------** 1

**Hoya thomsonii NC_067612.1**  **----------------------------------------------------------------------** 1

**Hoya verticillata NC_085236.1**  **----------------------------------------------------------------------** 1

**Papuahoya urniflora MW719062.1** **----------------------------------------------------------------------** 1

**Stephanotis volubilis OP133576** **----------------------------------------------------------------------** 1

**290 300 310 320 330 340 350**

**....|....|....|....|....|....|....|....|....|....|....|....|....|....|**

**Dischidia australis NC_067885.** **----------------------------------------------------------------------** 1

**Gymnema yunnanense NC_079598.1** **----------------------------------------------------------------------** 1

**Hoya ariadna NC_069568.1**  **----------------------------------------------------------------------** 1

**Hoya commutata NC_067958.1**  **----------------------------------------------------------------------** 1

**Hoya dimorpha NC_067959.1**  **----------------------------------------------------------------------** 1

**Hoya exilis MW719054.1**  **----------------------------------------------------------------------** 1

**Hoya griffithii NC_069565.1**  **----------------------------------------------------------------------** 1

**Hoya kerrii NC_069570.1**  **----------------------------------------------------------------------** 1

**Hoya lacunosa NC_069564.1**  **----------------------------------------------------------------------** 1

**Hoya lanceolata NC_067960.1**  **----------------------------------------------------------------------** 1

**Hoya liangii OL826865.1**  **----------------------------------------------------------------------** 1

**Hoya lithophytica MW719058.1**  **----------------------------------------------------------------------** 1

**Hoya lockii OR475243.1**  **----------------------------------------------------------------------** 1

**Hoya longifolia NC_069560.1**  **----------------------------------------------------------------------** 1

**Hoya lyi MW719055.1**  **----------------------------------------------------------------------** 1

**Hoya megalaster MW719063.1**  **AGGATCAATATATTGCTTATGTAGCTTACCCTTTAGACCTTTTTGAAGAAGGTTCTGTTACTAACATGCT** 350

**Hoya meliflua NC_069571.1**  **----------------------------------------------------------------------** 1

**Hoya monetteae MW719053.1**  **----------------------------------------------------------------------** 1

**Hoya omlorii MW719060.1**  **AGGATCAATATATTGCTTATGTAGCTTACCCTTTAGACCTTTTTGAAGAAGGTTCTGTTACTAACATGCT** 350

**Hoya ovalifolia NC_069563.1**  **----------------------------------------------------------------------** 1

**Hoya pandurata NC_069562.1**  **----------------------------------------------------------------------** 1

**Hoya pottsii OL754664.1**  **----------------------------------------------------------------------** 1

**Hoya pubicalyx NC_069561.1**  **----------------------------------------------------------------------** 1

**Hoya radicalis NC_067961.1**  **----------------------------------------------------------------------** 1

**Hoya rigida NC_067962.1**  **----------------------------------------------------------------------** 1

**Hoya silvatica NC_067963.1**  **----------------------------------------------------------------------** 1

**Hoya thomsonii NC_067612.1**  **----------------------------------------------------------------------** 1

**Hoya verticillata NC_085236.1**  **----------------------------------------------------------------------** 1

**Papuahoya urniflora MW719062.1** **----------------------------------------------------------------------** 1

**Stephanotis volubilis OP133576** **----------------------------------------------------------------------** 1

**360 370 380 390 400 410 420**

**....|....|....|....|....|....|....|....|....|....|....|....|....|....|**

**Dischidia australis NC_067885.** **----------------------------------------------------------------------** 1

**Gymnema yunnanense NC_079598.1** **----------------------------------------------------------------------** 1

**Hoya ariadna NC_069568.1**  **----------------------------------------------------------------------** 1

**Hoya commutata NC_067958.1**  **----------------------------------------------------------------------** 1

**Hoya dimorpha NC_067959.1**  **----------------------------------------------------------------------** 1

**Hoya exilis MW719054.1**  **----------------------------------------------------------------------** 1

**Hoya griffithii NC_069565.1**  **----------------------------------------------------------------------** 1

**Hoya kerrii NC_069570.1**  **----------------------------------------------------------------------** 1

**Hoya lacunosa NC_069564.1**  **----------------------------------------------------------------------** 1

**Hoya lanceolata NC_067960.1**  **----------------------------------------------------------------------** 1

**Hoya liangii OL826865.1**  **----------------------------------------------------------------------** 1

**Hoya lithophytica MW719058.1**  **----------------------------------------------------------------------** 1

**Hoya lockii OR475243.1**  **----------------------------------------------------------------------** 1

**Hoya longifolia NC_069560.1**  **----------------------------------------------------------------------** 1

**Hoya lyi MW719055.1**  **----------------------------------------------------------------------** 1

**Hoya megalaster MW719063.1**  **TACTTCCATTGTAGGTAATGTATTTGGGTTCAAAGCCCTACGCGCTCTACGTCTGGAAGATTTGCGAATC** 420

**Hoya meliflua NC_069571.1**  **----------------------------------------------------------------------** 1

**Hoya monetteae MW719053.1**  **----------------------------------------------------------------------** 1

**Hoya omlorii MW719060.1**  **TACTTCCATTGTAGGTAATGTATTTGGGTTCAAAGCCCTACGCGCTCTACGTCTGGAAGATTTGCGAATC** 420

**Hoya ovalifolia NC_069563.1**  **----------------------------------------------------------------------** 1

**Hoya pandurata NC_069562.1**  **----------------------------------------------------------------------** 1

**Hoya pottsii OL754664.1**  **----------------------------------------------------------------------** 1

**Hoya pubicalyx NC_069561.1**  **----------------------------------------------------------------------** 1

**Hoya radicalis NC_067961.1**  **----------------------------------------------------------------------** 1

**Hoya rigida NC_067962.1**  **----------------------------------------------------------------------** 1

**Hoya silvatica NC_067963.1**  **----------------------------------------------------------------------** 1

**Hoya thomsonii NC_067612.1**  **----------------------------------------------------------------------** 1

**Hoya verticillata NC_085236.1**  **----------------------------------------------------------------------** 1

**Papuahoya urniflora MW719062.1** **----------------------------------------------------------------------** 1

**Stephanotis volubilis OP133576** **----------------------------------------------------------------------** 1

**430 440 450 460 470 480 490**

**....|....|....|....|....|....|....|....|....|....|....|....|....|....|**

**Dischidia australis NC_067885.** **----------------------------------------------------------------------** 1

**Gymnema yunnanense NC_079598.1** **----------------------------------------------------------------------** 1

**Hoya ariadna NC_069568.1**  **----------------------------------------------------------------------** 1

**Hoya commutata NC_067958.1**  **----------------------------------------------------------------------** 1

**Hoya dimorpha NC_067959.1**  **----------------------------------------------------------------------** 1

**Hoya exilis MW719054.1**  **----------------------------------------------------------------------** 1

**Hoya griffithii NC_069565.1**  **----------------------------------------------------------------------** 1

**Hoya kerrii NC_069570.1**  **----------------------------------------------------------------------** 1

**Hoya lacunosa NC_069564.1**  **----------------------------------------------------------------------** 1

**Hoya lanceolata NC_067960.1**  **----------------------------------------------------------------------** 1

**Hoya liangii OL826865.1**  **----------------------------------------------------------------------** 1

**Hoya lithophytica MW719058.1**  **----------------------------------------------------------------------** 1

**Hoya lockii OR475243.1**  **----------------------------------------------------------------------** 1

**Hoya longifolia NC_069560.1**  **----------------------------------------------------------------------** 1

**Hoya lyi MW719055.1**  **----------------------------------------------------------------------** 1

**Hoya megalaster MW719063.1**  **CCCAAGGCTTATATTAAAACCTTCCAAGGCCCACCGCATGGCATCCAGGTTGAGAGAGATAAATTGAACA** 490

**Hoya meliflua NC_069571.1**  **----------------------------------------------------------------------** 1

**Hoya monetteae MW719053.1**  **----------------------------------------------------------------------** 1

**Hoya omlorii MW719060.1**  **CCTCCGGCTTATATTAAAACCTTCCAAGGCCCACCGCATGGCATCCAGGTTGAGAGAGATAAATTGAACA** 490

**Hoya ovalifolia NC_069563.1**  **----------------------------------------------------------------------** 1

**Hoya pandurata NC_069562.1**  **----------------------------------------------------------------------** 1

**Hoya pottsii OL754664.1**  **----------------------------------------------------------------------** 1

**Hoya pubicalyx NC_069561.1**  **----------------------------------------------------------------------** 1

**Hoya radicalis NC_067961.1**  **----------------------------------------------------------------------** 1

**Hoya rigida NC_067962.1**  **----------------------------------------------------------------------** 1

**Hoya silvatica NC_067963.1**  **----------------------------------------------------------------------** 1

**Hoya thomsonii NC_067612.1**  **----------------------------------------------------------------------** 1

**Hoya verticillata NC_085236.1**  **----------------------------------------------------------------------** 1

**Papuahoya urniflora MW719062.1** **----------------------------------------------------------------------** 1

**Stephanotis volubilis OP133576** **----------------------------------------------------------------------** 1

**500 510 520 530 540 550 560**

**....|....|....|....|....|....|....|....|....|....|....|....|....|....|**

**Dischidia australis NC_067885.** **----------------------------------------------------------------------** 1

**Gymnema yunnanense NC_079598.1** **----------------------------------------------------------------------** 1

**Hoya ariadna NC_069568.1**  **----------------------------------------------------------------------** 1

**Hoya commutata NC_067958.1**  **----------------------------------------------------------------------** 1

**Hoya dimorpha NC_067959.1**  **----------------------------------------------------------------------** 1

**Hoya exilis MW719054.1**  **----------------------------------------------------------------------** 1

**Hoya griffithii NC_069565.1**  **----------------------------------------------------------------------** 1

**Hoya kerrii NC_069570.1**  **----------------------------------------------------------------------** 1

**Hoya lacunosa NC_069564.1**  **----------------------------------------------------------------------** 1

**Hoya lanceolata NC_067960.1**  **----------------------------------------------------------------------** 1

**Hoya liangii OL826865.1**  **----------------------------------------------------------------------** 1

**Hoya lithophytica MW719058.1**  **----------------------------------------------------------------------** 1

**Hoya lockii OR475243.1**  **----------------------------------------------------------------------** 1

**Hoya longifolia NC_069560.1**  **----------------------------------------------------------------------** 1

**Hoya lyi MW719055.1**  **----------------------------------------------------------------------** 1

**Hoya megalaster MW719063.1**  **AATATGGTCGTCCCCTGTTGGGATGTACTATTAAACCAAAATTGGGGTTATCAGCTAAAAACTACGGTAG** 560

**Hoya meliflua NC_069571.1**  **----------------------------------------------------------------------** 1

**Hoya monetteae MW719053.1**  **----------------------------------------------------------------------** 1

**Hoya omlorii MW719060.1**  **AATATGGTCGTCCCCTGTTGGGATGTACTATTAAACCAAAATTGGGGTTATCAGCTAAAAACTACGGTAG** 560

**Hoya ovalifolia NC_069563.1**  **----------------------------------------------------------------------** 1

**Hoya pandurata NC_069562.1**  **----------------------------------------------------------------------** 1

**Hoya pottsii OL754664.1**  **----------------------------------------------------------------------** 1

**Hoya pubicalyx NC_069561.1**  **----------------------------------------------------------------------** 1

**Hoya radicalis NC_067961.1**  **----------------------------------------------------------------------** 1

**Hoya rigida NC_067962.1**  **----------------------------------------------------------------------** 1

**Hoya silvatica NC_067963.1**  **----------------------------------------------------------------------** 1

**Hoya thomsonii NC_067612.1**  **----------------------------------------------------------------------** 1

**Hoya verticillata NC_085236.1**  **----------------------------------------------------------------------** 1

**Papuahoya urniflora MW719062.1** **----------------------------------------------------------------------** 1

**Stephanotis volubilis OP133576** **----------------------------------------------------------------------** 1

**570 580 590 600 610 620 630**

**....|....|....|....|....|....|....|....|....|....|....|....|....|....|**

**Dischidia australis NC_067885.** **----------------------------------------------------------------------** 1

**Gymnema yunnanense NC_079598.1** **----------------------------------------------------------------------** 1

**Hoya ariadna NC_069568.1**  **----------------------------------------------------------------------** 1

**Hoya commutata NC_067958.1**  **----------------------------------------------------------------------** 1

**Hoya dimorpha NC_067959.1**  **----------------------------------------------------------------------** 1

**Hoya exilis MW719054.1**  **----------------------------------------------------------------------** 1

**Hoya griffithii NC_069565.1**  **----------------------------------------------------------------------** 1

**Hoya kerrii NC_069570.1**  **----------------------------------------------------------------------** 1

**Hoya lacunosa NC_069564.1**  **----------------------------------------------------------------------** 1

**Hoya lanceolata NC_067960.1**  **----------------------------------------------------------------------** 1

**Hoya liangii OL826865.1**  **----------------------------------------------------------------------** 1

**Hoya lithophytica MW719058.1**  **----------------------------------------------------------------------** 1

**Hoya lockii OR475243.1**  **----------------------------------------------------------------------** 1

**Hoya longifolia NC_069560.1**  **----------------------------------------------------------------------** 1

**Hoya lyi MW719055.1**  **----------------------------------------------------------------------** 1

**Hoya megalaster MW719063.1**  **GGCGGTTTATGAATGTCTTCGTGGTGGACTTGATTTTACCAAAGATGATGAAAACGTGAATTCCCAGCCG** 630

**Hoya meliflua NC_069571.1**  **----------------------------------------------------------------------** 1

**Hoya monetteae MW719053.1**  **----------------------------------------------------------------------** 1

**Hoya omlorii MW719060.1**  **GGCGGTTTATGAATGTCTTCGTGGTGGACTTGATTTTACCAAAGATGATGAAAACGTGAACTCCCAGCCG** 630

**Hoya ovalifolia NC_069563.1**  **----------------------------------------------------------------------** 1

**Hoya pandurata NC_069562.1**  **----------------------------------------------------------------------** 1

**Hoya pottsii OL754664.1**  **----------------------------------------------------------------------** 1

**Hoya pubicalyx NC_069561.1**  **----------------------------------------------------------------------** 1

**Hoya radicalis NC_067961.1**  **----------------------------------------------------------------------** 1

**Hoya rigida NC_067962.1**  **----------------------------------------------------------------------** 1

**Hoya silvatica NC_067963.1**  **----------------------------------------------------------------------** 1

**Hoya thomsonii NC_067612.1**  **----------------------------------------------------------------------** 1

**Hoya verticillata NC_085236.1**  **----------------------------------------------------------------------** 1

**Papuahoya urniflora MW719062.1** **----------------------------------------------------------------------** 1

**Stephanotis volubilis OP133576** **----------------------------------------------------------------------** 1

**640 650 660 670 680 690 700**

**....|....|....|....|....|....|....|....|....|....|....|....|....|....|**

**Dischidia australis NC_067885.** **----------------------------------------------------------------------** 1

**Gymnema yunnanense NC_079598.1** **----------------------------------------------------------------------** 1

**Hoya ariadna NC_069568.1**  **----------------------------------------------------------------------** 1

**Hoya commutata NC_067958.1**  **----------------------------------------------------------------------** 1

**Hoya dimorpha NC_067959.1**  **----------------------------------------------------------------------** 1

**Hoya exilis MW719054.1**  **----------------------------------------------------------------------** 1

**Hoya griffithii NC_069565.1**  **----------------------------------------------------------------------** 1

**Hoya kerrii NC_069570.1**  **----------------------------------------------------------------------** 1

**Hoya lacunosa NC_069564.1**  **----------------------------------------------------------------------** 1

**Hoya lanceolata NC_067960.1**  **----------------------------------------------------------------------** 1

**Hoya liangii OL826865.1**  **----------------------------------------------------------------------** 1

**Hoya lithophytica MW719058.1**  **----------------------------------------------------------------------** 1

**Hoya lockii OR475243.1**  **----------------------------------------------------------------------** 1

**Hoya longifolia NC_069560.1**  **----------------------------------------------------------------------** 1

**Hoya lyi MW719055.1**  **----------------------------------------------------------------------** 1

**Hoya megalaster MW719063.1**  **TTTATGCGTTGGAGAGATCGTTTCTTGTTTTGTGCCGAAGCAATTTTTAAATCACAGGCTGAAACTGGCG** 700

**Hoya meliflua NC_069571.1**  **----------------------------------------------------------------------** 1

**Hoya monetteae MW719053.1**  **----------------------------------------------------------------------** 1

**Hoya omlorii MW719060.1**  **TTTATGCGTTGGAGAGATCGTTTCTTGTTTTGTGCCGAAGCACTTTTTAAATCACAGGCTGAAACTGGCG** 700

**Hoya ovalifolia NC_069563.1**  **----------------------------------------------------------------------** 1

**Hoya pandurata NC_069562.1**  **----------------------------------------------------------------------** 1

**Hoya pottsii OL754664.1**  **----------------------------------------------------------------------** 1

**Hoya pubicalyx NC_069561.1**  **----------------------------------------------------------------------** 1

**Hoya radicalis NC_067961.1**  **----------------------------------------------------------------------** 1

**Hoya rigida NC_067962.1**  **----------------------------------------------------------------------** 1

**Hoya silvatica NC_067963.1**  **----------------------------------------------------------------------** 1

**Hoya thomsonii NC_067612.1**  **----------------------------------------------------------------------** 1

**Hoya verticillata NC_085236.1**  **----------------------------------------------------------------------** 1

**Papuahoya urniflora MW719062.1** **----------------------------------------------------------------------** 1

**Stephanotis volubilis OP133576** **----------------------------------------------------------------------** 1

**710 720 730 740 750 760 770**

**....|....|....|....|....|....|....|....|....|....|....|....|....|....|**

**Dischidia australis NC_067885.** **----------------------------------------------------------------------** 1

**Gymnema yunnanense NC_079598.1** **----------------------------------------------------------------------** 1

**Hoya ariadna NC_069568.1**  **----------------------------------------------------------------------** 1

**Hoya commutata NC_067958.1**  **----------------------------------------------------------------------** 1

**Hoya dimorpha NC_067959.1**  **----------------------------------------------------------------------** 1

**Hoya exilis MW719054.1**  **----------------------------------------------------------------------** 1

**Hoya griffithii NC_069565.1**  **----------------------------------------------------------------------** 1

**Hoya kerrii NC_069570.1**  **----------------------------------------------------------------------** 1

**Hoya lacunosa NC_069564.1**  **----------------------------------------------------------------------** 1

**Hoya lanceolata NC_067960.1**  **----------------------------------------------------------------------** 1

**Hoya liangii OL826865.1**  **----------------------------------------------------------------------** 1

**Hoya lithophytica MW719058.1**  **----------------------------------------------------------------------** 1

**Hoya lockii OR475243.1**  **----------------------------------------------------------------------** 1

**Hoya longifolia NC_069560.1**  **----------------------------------------------------------------------** 1

**Hoya lyi MW719055.1**  **----------------------------------------------------------------------** 1

**Hoya megalaster MW719063.1**  **AAATCAAAGGACATTACTTGAATGCTACTGCAGGTACATGTGAAGAAATGTACAAAAGAGCTATATTTGC** 770

**Hoya meliflua NC_069571.1**  **----------------------------------------------------------------------** 1

**Hoya monetteae MW719053.1**  **----------------------------------------------------------------------** 1

**Hoya omlorii MW719060.1**  **AAATCAAAGGACATTACTTGAATGCTACTGCAGGTACATGTGAAGAAATGTACAAAAGAGCTATATTTGC** 770

**Hoya ovalifolia NC_069563.1**  **----------------------------------------------------------------------** 1

**Hoya pandurata NC_069562.1**  **----------------------------------------------------------------------** 1

**Hoya pottsii OL754664.1**  **----------------------------------------------------------------------** 1

**Hoya pubicalyx NC_069561.1**  **----------------------------------------------------------------------** 1

**Hoya radicalis NC_067961.1**  **----------------------------------------------------------------------** 1

**Hoya rigida NC_067962.1**  **----------------------------------------------------------------------** 1

**Hoya silvatica NC_067963.1**  **----------------------------------------------------------------------** 1

**Hoya thomsonii NC_067612.1**  **----------------------------------------------------------------------** 1

**Hoya verticillata NC_085236.1**  **----------------------------------------------------------------------** 1

**Papuahoya urniflora MW719062.1** **----------------------------------------------------------------------** 1

**Stephanotis volubilis OP133576** **----------------------------------------------------------------------** 1

**780 790 800 810 820 830 840**

**....|....|....|....|....|....|....|....|....|....|....|....|....|....|**

**Dischidia australis NC_067885.** **----------------------------------------------------------------------** 1

**Gymnema yunnanense NC_079598.1** **----------------------------------------------------------------------** 1

**Hoya ariadna NC_069568.1**  **----------------------------------------------------------------------** 1

**Hoya commutata NC_067958.1**  **----------------------------------------------------------------------** 1

**Hoya dimorpha NC_067959.1**  **----------------------------------------------------------------------** 1

**Hoya exilis MW719054.1**  **----------------------------------------------------------------------** 1

**Hoya griffithii NC_069565.1**  **----------------------------------------------------------------------** 1

**Hoya kerrii NC_069570.1**  **----------------------------------------------------------------------** 1

**Hoya lacunosa NC_069564.1**  **----------------------------------------------------------------------** 1

**Hoya lanceolata NC_067960.1**  **----------------------------------------------------------------------** 1

**Hoya liangii OL826865.1**  **----------------------------------------------------------------------** 1

**Hoya lithophytica MW719058.1**  **----------------------------------------------------------------------** 1

**Hoya lockii OR475243.1**  **----------------------------------------------------------------------** 1

**Hoya longifolia NC_069560.1**  **----------------------------------------------------------------------** 1

**Hoya lyi MW719055.1**  **----------------------------------------------------------------------** 1

**Hoya megalaster MW719063.1**  **TAGAGAATTGGGAGCTCCTATCGTAATGCATGACTACTTAACAGGGGGATTCACTGCAAATACGAGCTTG** 840

**Hoya meliflua NC_069571.1**  **----------------------------------------------------------------------** 1

**Hoya monetteae MW719053.1**  **----------------------------------------------------------------------** 1

**Hoya omlorii MW719060.1**  **TAGAGAATTGGGAGTTCCTATCGTAATGCATGACTACTTAACAGGGGGATTCACTGCAAATACGAGCTTG** 840

**Hoya ovalifolia NC_069563.1**  **----------------------------------------------------------------------** 1

**Hoya pandurata NC_069562.1**  **----------------------------------------------------------------------** 1

**Hoya pottsii OL754664.1**  **----------------------------------------------------------------------** 1

**Hoya pubicalyx NC_069561.1**  **----------------------------------------------------------------------** 1

**Hoya radicalis NC_067961.1**  **----------------------------------------------------------------------** 1

**Hoya rigida NC_067962.1**  **----------------------------------------------------------------------** 1

**Hoya silvatica NC_067963.1**  **----------------------------------------------------------------------** 1

**Hoya thomsonii NC_067612.1**  **----------------------------------------------------------------------** 1

**Hoya verticillata NC_085236.1**  **----------------------------------------------------------------------** 1

**Papuahoya urniflora MW719062.1** **----------------------------------------------------------------------** 1

**Stephanotis volubilis OP133576** **----------------------------------------------------------------------** 1

**850 860 870 880 890 900 910**

**....|....|....|....|....|....|....|....|....|....|....|....|....|....|**

**Dischidia australis NC_067885.** **----------------------------------------------------------------------** 1

**Gymnema yunnanense NC_079598.1** **----------------------------------------------------------------------** 1

**Hoya ariadna NC_069568.1**  **----------------------------------------------------------------------** 1

**Hoya commutata NC_067958.1**  **----------------------------------------------------------------------** 1

**Hoya dimorpha NC_067959.1**  **----------------------------------------------------------------------** 1

**Hoya exilis MW719054.1**  **----------------------------------------------------------------------** 1

**Hoya griffithii NC_069565.1**  **----------------------------------------------------------------------** 1

**Hoya kerrii NC_069570.1**  **----------------------------------------------------------------------** 1

**Hoya lacunosa NC_069564.1**  **----------------------------------------------------------------------** 1

**Hoya lanceolata NC_067960.1**  **----------------------------------------------------------------------** 1

**Hoya liangii OL826865.1**  **----------------------------------------------------------------------** 1

**Hoya lithophytica MW719058.1**  **----------------------------------------------------------------------** 1

**Hoya lockii OR475243.1**  **----------------------------------------------------------------------** 1

**Hoya longifolia NC_069560.1**  **----------------------------------------------------------------------** 1

**Hoya lyi MW719055.1**  **----------------------------------------------------------------------** 1

**Hoya megalaster MW719063.1**  **GCTCATTATTGCCGAGATAATGGTCTACTTCTTCACATCCACCGTGCAATGCACGCAGTTATTGATAGAC** 910

**Hoya meliflua NC_069571.1**  **----------------------------------------------------------------------** 1

**Hoya monetteae MW719053.1**  **----------------------------------------------------------------------** 1

**Hoya omlorii MW719060.1**  **GCTCATTATTGCCGAGATAATGGTCTACTTCTTCACATCCACCGTGCAATGCACGCAGTTATTGATAGAC** 910

**Hoya ovalifolia NC_069563.1**  **----------------------------------------------------------------------** 1

**Hoya pandurata NC_069562.1**  **----------------------------------------------------------------------** 1

**Hoya pottsii OL754664.1**  **----------------------------------------------------------------------** 1

**Hoya pubicalyx NC_069561.1**  **----------------------------------------------------------------------** 1

**Hoya radicalis NC_067961.1**  **----------------------------------------------------------------------** 1

**Hoya rigida NC_067962.1**  **----------------------------------------------------------------------** 1

**Hoya silvatica NC_067963.1**  **----------------------------------------------------------------------** 1

**Hoya thomsonii NC_067612.1**  **----------------------------------------------------------------------** 1

**Hoya verticillata NC_085236.1**  **----------------------------------------------------------------------** 1

**Papuahoya urniflora MW719062.1** **----------------------------------------------------------------------** 1

**Stephanotis volubilis OP133576** **----------------------------------------------------------------------** 1

**920 930 940 950 960 970 980**

**....|....|....|....|....|....|....|....|....|....|....|....|....|....|**

**Dischidia australis NC_067885.** **----------------------------------------------------------------------** 1

**Gymnema yunnanense NC_079598.1** **----------------------------------------------------------------------** 1

**Hoya ariadna NC_069568.1**  **----------------------------------------------------------------------** 1

**Hoya commutata NC_067958.1**  **----------------------------------------------------------------------** 1

**Hoya dimorpha NC_067959.1**  **----------------------------------------------------------------------** 1

**Hoya exilis MW719054.1**  **----------------------------------------------------------------------** 1

**Hoya griffithii NC_069565.1**  **----------------------------------------------------------------------** 1

**Hoya kerrii NC_069570.1**  **----------------------------------------------------------------------** 1

**Hoya lacunosa NC_069564.1**  **----------------------------------------------------------------------** 1

**Hoya lanceolata NC_067960.1**  **----------------------------------------------------------------------** 1

**Hoya liangii OL826865.1**  **----------------------------------------------------------------------** 1

**Hoya lithophytica MW719058.1**  **----------------------------------------------------------------------** 1

**Hoya lockii OR475243.1**  **----------------------------------------------------------------------** 1

**Hoya longifolia NC_069560.1**  **----------------------------------------------------------------------** 1

**Hoya lyi MW719055.1**  **----------------------------------------------------------------------** 1

**Hoya megalaster MW719063.1**  **AGAAGAATCATGGCATGCACTTCCGCGTACTAGCTAAAGCGTTACGTATGTCTGGTGGAGATCATATTCA** 980

**Hoya meliflua NC_069571.1**  **----------------------------------------------------------------------** 1

**Hoya monetteae MW719053.1**  **----------------------------------------------------------------------** 1

**Hoya omlorii MW719060.1**  **AGAAGAATCATGGCATGCACTTCCGCGTACTAGCTAAAGCGTTACGTATGTCTGGTGGAGATCATATTCA** 980

**Hoya ovalifolia NC_069563.1**  **----------------------------------------------------------------------** 1

**Hoya pandurata NC_069562.1**  **----------------------------------------------------------------------** 1

**Hoya pottsii OL754664.1**  **----------------------------------------------------------------------** 1

**Hoya pubicalyx NC_069561.1**  **----------------------------------------------------------------------** 1

**Hoya radicalis NC_067961.1**  **----------------------------------------------------------------------** 1

**Hoya rigida NC_067962.1**  **----------------------------------------------------------------------** 1

**Hoya silvatica NC_067963.1**  **----------------------------------------------------------------------** 1

**Hoya thomsonii NC_067612.1**  **----------------------------------------------------------------------** 1

**Hoya verticillata NC_085236.1**  **----------------------------------------------------------------------** 1

**Papuahoya urniflora MW719062.1** **----------------------------------------------------------------------** 1

**Stephanotis volubilis OP133576** **----------------------------------------------------------------------** 1

**990 1000 1010 1020 1030 1040 1050**

**....|....|....|....|....|....|....|....|....|....|....|....|....|....|**

**Dischidia australis NC_067885.** **----------------------------------------------------------------------** 1

**Gymnema yunnanense NC_079598.1** **----------------------------------------------------------------------** 1

**Hoya ariadna NC_069568.1**  **----------------------------------------------------------------------** 1

**Hoya commutata NC_067958.1**  **----------------------------------------------------------------------** 1

**Hoya dimorpha NC_067959.1**  **----------------------------------------------------------------------** 1

**Hoya exilis MW719054.1**  **----------------------------------------------------------------------** 1

**Hoya griffithii NC_069565.1**  **----------------------------------------------------------------------** 1

**Hoya kerrii NC_069570.1**  **----------------------------------------------------------------------** 1

**Hoya lacunosa NC_069564.1**  **----------------------------------------------------------------------** 1

**Hoya lanceolata NC_067960.1**  **----------------------------------------------------------------------** 1

**Hoya liangii OL826865.1**  **----------------------------------------------------------------------** 1

**Hoya lithophytica MW719058.1**  **----------------------------------------------------------------------** 1

**Hoya lockii OR475243.1**  **----------------------------------------------------------------------** 1

**Hoya longifolia NC_069560.1**  **----------------------------------------------------------------------** 1

**Hoya lyi MW719055.1**  **----------------------------------------------------------------------** 1

**Hoya megalaster MW719063.1**  **CGCGGGTACCGTAGTAGGTAAACTTGAAGGGGAAAGAGAAATCACTTTGGGCTTTGTTGATTTACTGCGT** 1050

**Hoya meliflua NC_069571.1**  **----------------------------------------------------------------------** 1

**Hoya monetteae MW719053.1**  **----------------------------------------------------------------------** 1

**Hoya omlorii MW719060.1**  **CGCGGGTACCGTAGTAGGTAAACTTGAAGGGGAAAGAGAAATCACTTTGGGCTTTGTTGATTTACTGCGT** 1050

**Hoya ovalifolia NC_069563.1**  **----------------------------------------------------------------------** 1

**Hoya pandurata NC_069562.1**  **----------------------------------------------------------------------** 1

**Hoya pottsii OL754664.1**  **----------------------------------------------------------------------** 1

**Hoya pubicalyx NC_069561.1**  **----------------------------------------------------------------------** 1

**Hoya radicalis NC_067961.1**  **----------------------------------------------------------------------** 1

**Hoya rigida NC_067962.1**  **----------------------------------------------------------------------** 1

**Hoya silvatica NC_067963.1**  **----------------------------------------------------------------------** 1

**Hoya thomsonii NC_067612.1**  **----------------------------------------------------------------------** 1

**Hoya verticillata NC_085236.1**  **----------------------------------------------------------------------** 1

**Papuahoya urniflora MW719062.1** **----------------------------------------------------------------------** 1

**Stephanotis volubilis OP133576** **----------------------------------------------------------------------** 1

**1060 1070 1080 1090 1100 1110 1120**

**....|....|....|....|....|....|....|....|....|....|....|....|....|....|**

**Dischidia australis NC_067885.** **----------------------------------------------------------------------** 1

**Gymnema yunnanense NC_079598.1** **----------------------------------------------------------------------** 1

**Hoya ariadna NC_069568.1**  **----------------------------------------------------------------------** 1

**Hoya commutata NC_067958.1**  **----------------------------------------------------------------------** 1

**Hoya dimorpha NC_067959.1**  **----------------------------------------------------------------------** 1

**Hoya exilis MW719054.1**  **----------------------------------------------------------------------** 1

**Hoya griffithii NC_069565.1**  **----------------------------------------------------------------------** 1

**Hoya kerrii NC_069570.1**  **----------------------------------------------------------------------** 1

**Hoya lacunosa NC_069564.1**  **----------------------------------------------------------------------** 1

**Hoya lanceolata NC_067960.1**  **----------------------------------------------------------------------** 1

**Hoya liangii OL826865.1**  **----------------------------------------------------------------------** 1

**Hoya lithophytica MW719058.1**  **----------------------------------------------------------------------** 1

**Hoya lockii OR475243.1**  **----------------------------------------------------------------------** 1

**Hoya longifolia NC_069560.1**  **----------------------------------------------------------------------** 1

**Hoya lyi MW719055.1**  **----------------------------------------------------------------------** 1

**Hoya megalaster MW719063.1**  **GATGATTTTATTGAAAAAGATCGAAGTCGCGGTATTTATTTCACTCAAGATTGGGTCTCTCTACCAGGCG** 1120

**Hoya meliflua NC_069571.1**  **----------------------------------------------------------------------** 1

**Hoya monetteae MW719053.1**  **----------------------------------------------------------------------** 1

**Hoya omlorii MW719060.1**  **GATGATTTTATTGAAAAAGATCGAAGTCGCGGTATTTATTTCACTCAAGATTGGGTCTCTCTACCAGGCG** 1120

**Hoya ovalifolia NC_069563.1**  **----------------------------------------------------------------------** 1

**Hoya pandurata NC_069562.1**  **----------------------------------------------------------------------** 1

**Hoya pottsii OL754664.1**  **----------------------------------------------------------------------** 1

**Hoya pubicalyx NC_069561.1**  **----------------------------------------------------------------------** 1

**Hoya radicalis NC_067961.1**  **----------------------------------------------------------------------** 1

**Hoya rigida NC_067962.1**  **----------------------------------------------------------------------** 1

**Hoya silvatica NC_067963.1**  **----------------------------------------------------------------------** 1

**Hoya thomsonii NC_067612.1**  **----------------------------------------------------------------------** 1

**Hoya verticillata NC_085236.1**  **----------------------------------------------------------------------** 1

**Papuahoya urniflora MW719062.1** **----------------------------------------------------------------------** 1

**Stephanotis volubilis OP133576** **----------------------------------------------------------------------** 1

**1130 1140 1150 1160 1170 1180 1190**

**....|....|....|....|....|....|....|....|....|....|....|....|....|....|**

**Dischidia australis NC_067885.** **----------------------------------------------------------------------** 1

**Gymnema yunnanense NC_079598.1** **----------------------------------------------------------------------** 1

**Hoya ariadna NC_069568.1**  **----------------------------------------------------------------------** 1

**Hoya commutata NC_067958.1**  **----------------------------------------------------------------------** 1

**Hoya dimorpha NC_067959.1**  **----------------------------------------------------------------------** 1

**Hoya exilis MW719054.1**  **----------------------------------------------------------------------** 1

**Hoya griffithii NC_069565.1**  **----------------------------------------------------------------------** 1

**Hoya kerrii NC_069570.1**  **----------------------------------------------------------------------** 1

**Hoya lacunosa NC_069564.1**  **----------------------------------------------------------------------** 1

**Hoya lanceolata NC_067960.1**  **----------------------------------------------------------------------** 1

**Hoya liangii OL826865.1**  **----------------------------------------------------------------------** 1

**Hoya lithophytica MW719058.1**  **----------------------------------------------------------------------** 1

**Hoya lockii OR475243.1**  **----------------------------------------------------------------------** 1

**Hoya longifolia NC_069560.1**  **----------------------------------------------------------------------** 1

**Hoya lyi MW719055.1**  **----------------------------------------------------------------------** 1

**Hoya megalaster MW719063.1**  **TTCTGCCGGTGGCTTCAGGGGGTATTCACGTTTGGCATATGCCCGCTCTGACCGAGATCTTTGGGGATGA** 1190

**Hoya meliflua NC_069571.1**  **----------------------------------------------------------------------** 1

**Hoya monetteae MW719053.1**  **----------------------------------------------------------------------** 1

**Hoya omlorii MW719060.1**  **TTCTGCCGGTGGCTTCAGGGGGTATTCACGTTTGGCATATGCCCGCTCTGACCGAGATCTTTGGGGATGA** 1190

**Hoya ovalifolia NC_069563.1**  **----------------------------------------------------------------------** 1

**Hoya pandurata NC_069562.1**  **----------------------------------------------------------------------** 1

**Hoya pottsii OL754664.1**  **----------------------------------------------------------------------** 1

**Hoya pubicalyx NC_069561.1**  **----------------------------------------------------------------------** 1

**Hoya radicalis NC_067961.1**  **----------------------------------------------------------------------** 1

**Hoya rigida NC_067962.1**  **----------------------------------------------------------------------** 1

**Hoya silvatica NC_067963.1**  **----------------------------------------------------------------------** 1

**Hoya thomsonii NC_067612.1**  **----------------------------------------------------------------------** 1

**Hoya verticillata NC_085236.1**  **----------------------------------------------------------------------** 1

**Papuahoya urniflora MW719062.1** **----------------------------------------------------------------------** 1

**Stephanotis volubilis OP133576** **----------------------------------------------------------------------** 1

**1200 1210 1220 1230 1240 1250 1260**

**....|....|....|....|....|....|....|....|....|....|....|....|....|....|**

**Dischidia australis NC_067885.** **----------------------------------------------------------------------** 1

**Gymnema yunnanense NC_079598.1** **----------------------------------------------------------------------** 1

**Hoya ariadna NC_069568.1**  **----------------------------------------------------------------------** 1

**Hoya commutata NC_067958.1**  **----------------------------------------------------------------------** 1

**Hoya dimorpha NC_067959.1**  **----------------------------------------------------------------------** 1

**Hoya exilis MW719054.1**  **----------------------------------------------------------------------** 1

**Hoya griffithii NC_069565.1**  **----------------------------------------------------------------------** 1

**Hoya kerrii NC_069570.1**  **----------------------------------------------------------------------** 1

**Hoya lacunosa NC_069564.1**  **----------------------------------------------------------------------** 1

**Hoya lanceolata NC_067960.1**  **----------------------------------------------------------------------** 1

**Hoya liangii OL826865.1**  **----------------------------------------------------------------------** 1

**Hoya lithophytica MW719058.1**  **----------------------------------------------------------------------** 1

**Hoya lockii OR475243.1**  **----------------------------------------------------------------------** 1

**Hoya longifolia NC_069560.1**  **----------------------------------------------------------------------** 1

**Hoya lyi MW719055.1**  **----------------------------------------------------------------------** 1

**Hoya megalaster MW719063.1**  **TTCTGTACTACAGTTCGGTGGAGGAACTTTAGGACATCCTTGGGGTAATGCGCCAGGTGCCGTAGCGAAT** 1260

**Hoya meliflua NC_069571.1**  **----------------------------------------------------------------------** 1

**Hoya monetteae MW719053.1**  **----------------------------------------------------------------------** 1

**Hoya omlorii MW719060.1**  **TTCTGTACTACAGTTCGGTGGAGGAACTTTAGGACACCCTTGGGGTAATGCGCCAGGTGCCGTAGCGAAT** 1260

**Hoya ovalifolia NC_069563.1**  **----------------------------------------------------------------------** 1

**Hoya pandurata NC_069562.1**  **----------------------------------------------------------------------** 1

**Hoya pottsii OL754664.1**  **----------------------------------------------------------------------** 1

**Hoya pubicalyx NC_069561.1**  **----------------------------------------------------------------------** 1

**Hoya radicalis NC_067961.1**  **----------------------------------------------------------------------** 1

**Hoya rigida NC_067962.1**  **----------------------------------------------------------------------** 1

**Hoya silvatica NC_067963.1**  **----------------------------------------------------------------------** 1

**Hoya thomsonii NC_067612.1**  **----------------------------------------------------------------------** 1

**Hoya verticillata NC_085236.1**  **----------------------------------------------------------------------** 1

**Papuahoya urniflora MW719062.1** **----------------------------------------------------------------------** 1

**Stephanotis volubilis OP133576** **----------------------------------------------------------------------** 1

**1270 1280 1290 1300 1310 1320 1330**

**....|....|....|....|....|....|....|....|....|....|....|....|....|....|**

**Dischidia australis NC_067885.** **----------------------------------------------------------------------** 1

**Gymnema yunnanense NC_079598.1** **----------------------------------------------------------------------** 1

**Hoya ariadna NC_069568.1**  **----------------------------------------------------------------------** 1

**Hoya commutata NC_067958.1**  **----------------------------------------------------------------------** 1

**Hoya dimorpha NC_067959.1**  **----------------------------------------------------------------------** 1

**Hoya exilis MW719054.1**  **----------------------------------------------------------------------** 1

**Hoya griffithii NC_069565.1**  **----------------------------------------------------------------------** 1

**Hoya kerrii NC_069570.1**  **----------------------------------------------------------------------** 1

**Hoya lacunosa NC_069564.1**  **----------------------------------------------------------------------** 1

**Hoya lanceolata NC_067960.1**  **----------------------------------------------------------------------** 1

**Hoya liangii OL826865.1**  **----------------------------------------------------------------------** 1

**Hoya lithophytica MW719058.1**  **----------------------------------------------------------------------** 1

**Hoya lockii OR475243.1**  **----------------------------------------------------------------------** 1

**Hoya longifolia NC_069560.1**  **----------------------------------------------------------------------** 1

**Hoya lyi MW719055.1**  **----------------------------------------------------------------------** 1

**Hoya megalaster MW719063.1**  **CGAGTAGCTCTAGAAGCATGTGTACAAGCTCGTAATGAAGGGCGCGATCTTGCTGTTGAGGGTAATGAAA** 1330

**Hoya meliflua NC_069571.1**  **----------------------------------------------------------------------** 1

**Hoya monetteae MW719053.1**  **----------------------------------------------------------------------** 1

**Hoya omlorii MW719060.1**  **CGAGTAGCTCTAGAAGCATGTGTACAAGCTCGTAATGAAGGGCGCGATCTTGCTGTTGAGGGTAATGAAA** 1330

**Hoya ovalifolia NC_069563.1**  **----------------------------------------------------------------------** 1

**Hoya pandurata NC_069562.1**  **----------------------------------------------------------------------** 1

**Hoya pottsii OL754664.1**  **----------------------------------------------------------------------** 1

**Hoya pubicalyx NC_069561.1**  **----------------------------------------------------------------------** 1

**Hoya radicalis NC_067961.1**  **----------------------------------------------------------------------** 1

**Hoya rigida NC_067962.1**  **----------------------------------------------------------------------** 1

**Hoya silvatica NC_067963.1**  **----------------------------------------------------------------------** 1

**Hoya thomsonii NC_067612.1**  **----------------------------------------------------------------------** 1

**Hoya verticillata NC_085236.1**  **----------------------------------------------------------------------** 1

**Papuahoya urniflora MW719062.1** **----------------------------------------------------------------------** 1

**Stephanotis volubilis OP133576** **----------------------------------------------------------------------** 1

**1340 1350 1360 1370 1380 1390 1400**

**....|....|....|....|....|....|....|....|....|....|....|....|....|....|**

**Dischidia australis NC_067885.** **----------------------------------------------------------------------** 1

**Gymnema yunnanense NC_079598.1** **----------------------------------------------------------------------** 1

**Hoya ariadna NC_069568.1**  **----------------------------------------------------------------------** 1

**Hoya commutata NC_067958.1**  **----------------------------------------------------------------------** 1

**Hoya dimorpha NC_067959.1**  **----------------------------------------------------------------------** 1

**Hoya exilis MW719054.1**  **----------------------------------------------------------------------** 1

**Hoya griffithii NC_069565.1**  **----------------------------------------------------------------------** 1

**Hoya kerrii NC_069570.1**  **----------------------------------------------------------------------** 1

**Hoya lacunosa NC_069564.1**  **----------------------------------------------------------------------** 1

**Hoya lanceolata NC_067960.1**  **----------------------------------------------------------------------** 1

**Hoya liangii OL826865.1**  **----------------------------------------------------------------------** 1

**Hoya lithophytica MW719058.1**  **----------------------------------------------------------------------** 1

**Hoya lockii OR475243.1**  **----------------------------------------------------------------------** 1

**Hoya longifolia NC_069560.1**  **----------------------------------------------------------------------** 1

**Hoya lyi MW719055.1**  **----------------------------------------------------------------------** 1

**Hoya megalaster MW719063.1**  **TTATCCGTGAGGCTAGCAAATGGAGTCCTGAACTGGCTGCTGCTTGTGAGGTATGGAAGGAGATCAGATT** 1400

**Hoya meliflua NC_069571.1**  **----------------------------------------------------------------------** 1

**Hoya monetteae MW719053.1**  **----------------------------------------------------------------------** 1

**Hoya omlorii MW719060.1**  **TTATCCGTGAGGCTAGCAAATGGAGTCCTGAACTGGCTGCTGCTTGTGAGGTATGGAAGGAGATCAGATT** 1400

**Hoya ovalifolia NC_069563.1**  **----------------------------------------------------------------------** 1

**Hoya pandurata NC_069562.1**  **----------------------------------------------------------------------** 1

**Hoya pottsii OL754664.1**  **----------------------------------------------------------------------** 1

**Hoya pubicalyx NC_069561.1**  **----------------------------------------------------------------------** 1

**Hoya radicalis NC_067961.1**  **----------------------------------------------------------------------** 1

**Hoya rigida NC_067962.1**  **----------------------------------------------------------------------** 1

**Hoya silvatica NC_067963.1**  **----------------------------------------------------------------------** 1

**Hoya thomsonii NC_067612.1**  **----------------------------------------------------------------------** 1

**Hoya verticillata NC_085236.1**  **----------------------------------------------------------------------** 1

**Papuahoya urniflora MW719062.1** **----------------------------------------------------------------------** 1

**Stephanotis volubilis OP133576** **----------------------------------------------------------------------** 1

**1410 1420 1430 1440 1450 1460 1470**

**....|....|....|....|....|....|....|....|....|....|....|....|....|....|**

**Dischidia australis NC_067885.** **----------------------------TTACTTTTTGTTATCTTAGTTGAATTGTAATTAAACTCGGCC** 42

**Gymnema yunnanense NC_079598.1** **----------------------------TTACTTTTTGTTATCTTAGTTGAATTGTAATTAAACTCGGCC** 42

**Hoya ariadna NC_069568.1**  **----------------------------TTACTTTTTGTTATCTTAGTTGAATTGTAATTAAACTCGGCC** 42

**Hoya commutata NC_067958.1**  **----------------------------TTACTTTTTGTTATCTTAGTTGAATTGTAATTAAACTCGGCC** 42

**Hoya dimorpha NC_067959.1**  **----------------------------TTACTTTTTGTTATCTTAGTTGAATTGTAATTAAACTCGGCC** 42

**Hoya exilis MW719054.1**  **----------------------------TTACTTTTTGTTATCTTAGTTGAATTTTAATTAAACTCGGCC** 42

**Hoya griffithii NC_069565.1**  **----------------------------TTACTTTTTGTTATCTTAGTTGAATTGTAATTAAACTCGGCC** 42

**Hoya kerrii NC_069570.1**  **----------------------------TTACTTTTTGTTATCTTAGTTGAATTGTAATTAAACTCGGCC** 42

**Hoya lacunosa NC_069564.1**  **----------------------------TTACTTTTTGTTATCTTAGTTGAATTGTAATTAAACTCGGCC** 42

**Hoya lanceolata NC_067960.1**  **----------------------------TTACTTTTTGTTATCTTAGTTGAATTGTAATTAAACTCGGCC** 42

**Hoya liangii OL826865.1**  **----------------------------TTACTTTTTGTTATCTTAGTTGAATTGTAATTAAACTCGGCC** 42

**Hoya lithophytica MW719058.1**  **----------------------------TTACTTTTTGTTATCTTAGTTGAATTGTAATTAAACTCGGCC** 42

**Hoya lockii OR475243.1**  **----------------------------TTACTTTTTGTTATCTTAGTTGAATTGTAATTAAACTCGGCC** 42

**Hoya longifolia NC_069560.1**  **----------------------------TTACTTTTTGTTATCTTAGTTGAATTGTAATTAAACTCGGCC** 42

**Hoya lyi MW719055.1**  **----------------------------TTACTTTTTGTTATCTTAGTTGAATTGTAATTAAACTCGGCC** 42

**Hoya megalaster MW719063.1**  **TAATTTTCAAGCAGTGGATACTTTGTAATTACTTTTTGTTATCTTAGTTGAATTGTAATTAAACTCGGCC** 1470

**Hoya meliflua NC_069571.1**  **----------------------------TTACTTTTTGTTATCTTAGTTGAATTGTAATTAAACTCGGCC** 42

**Hoya monetteae MW719053.1**  **----------------------------TTACTTTTTGTTATCTTAGTTGAATTGTAATTAAACTCGGCC** 42

**Hoya omlorii MW719060.1**  **TAATTTTCAAGCAGTGGATACTTTGTAATTACTTTTTGTTATCTTAGTTGAATTGTAATTAAACTCGGCC** 1470

**Hoya ovalifolia NC_069563.1**  **----------------------------TTACTTTTTGTTATCTTAGTTGAATTGTAATTAAACTCGGCC** 42

**Hoya pandurata NC_069562.1**  **----------------------------TTACTTTTTGTTATCTTAGTTGAATTGTAATTAAACTCGGCC** 42

**Hoya pottsii OL754664.1**  **----------------------------TTACTTTTTGTTATCTTAGTTGAATTGTAATTAAACTCGGCC** 42

**Hoya pubicalyx NC_069561.1**  **----------------------------TTACTTTTTGTTATCTTAGTTGAATTGTAATTAAACTCGGCC** 42

**Hoya radicalis NC_067961.1**  **----------------------------TTACTTTTTGTTATCTTAGTTGAATTGTAATTAAACTCGGCC** 42

**Hoya rigida NC_067962.1**  **----------------------------TTACTTTTTGTTATCTTAGTTGAATTGTAATTAAACTCGGCC** 42

**Hoya silvatica NC_067963.1**  **----------------------------TTACTTTTTGTTATCTTAGTTGAATTGTAATTAAACTCGGCC** 42

**Hoya thomsonii NC_067612.1**  **----------------------------TTACTTTTTGTTATCTTAGTTGAATTGTAATTAAACTCGGCC** 42

**Hoya verticillata NC_085236.1**  **----------------------------TTACTTTTTGTTATCTTAGTTGAATTGTAATTAAACTCGGCC** 42

**Papuahoya urniflora MW719062.1** **----------------------------TTACTTTTTGTTATCTTAGTTGAATTGTAATTAAACTCGGCC** 42

**Stephanotis volubilis OP133576** **----------------------------TTACTTTTTGTTATCTTAGTTGAATTGTAATTAAACTCGGCC** 42

**1480 1490 1500 1510 1520 1530 1540**

**....|....|....|....|....|....|....|....|....|....|....|....|....|....|**

**Dischidia australis NC_067885.** **CAATCTTTTCCTAAAAGGATTGAGCCGAAT------ACAAAGATTCTATTTTTTTTAT------------** 94

**Gymnema yunnanense NC_079598.1** **CAATCTTTTCCTAAAAGGATTGAGCCGAAT------ACAAAGATTCTATTTTTTTTTA-TTTAATTATTT** 105

**Hoya ariadna NC_069568.1**  **CAATCTTTTCCTAAAAGGATTGAGCCGAAT------ACAAAGATTCTATTTATTTTTT------------** 94

**Hoya commutata NC_067958.1**  **CAATCTTTTCCTAAAAGGATTGAGCCGAAT------ACAAAGATTCTTTTTT--TTTTTAT---------** 95

**Hoya dimorpha NC_067959.1**  **CAATCTTTTCCTAAAAGGATTGAGCCGAAT------ACAAAGATTCTATTTTTTGTTTT-----------** 95

**Hoya exilis MW719054.1**  **CAATCTTTTCCTAAAAGGATTGAGCCGAAT------ACAAAGATTCTATTTTTTTTTTTTT---------** 97

**Hoya griffithii NC_069565.1**  **CAATCTTTTCCTAAAAGGATTGAGCCGAAT------ACAAAGATTCTATTTTTTTTTTT-----------** 95

**Hoya kerrii NC_069570.1**  **CAATCTTTTCCTAAAAGGATTGAGCCGAAT------ACAAAGATTCTTTTTTTTTTTA------------** 94

**Hoya lacunosa NC_069564.1**  **CAATCTTTTCCTAAAAGGATTGAGCCGAAT------ACAAAGATTCTATTTTTATTTTTTA---------** 97

**Hoya lanceolata NC_067960.1**  **CAATCTTTTCCTAAAAGGATTGAGCCGAAT------ACAAAGATTCTATTTT-TTTTTT-----------** 94

**Hoya liangii OL826865.1**  **CAATCTTTTCCTAAAAGGATTGAGCCGAAT------ACAAAGATTCTATTTTTTTTTTTT----------** 96

**Hoya lithophytica MW719058.1**  **CAATCTTTTCCTAAAAGGATTGAGCCGAAT------ACAAAGATTCTATTTTCTTTTTT-----------** 95

**Hoya lockii OR475243.1**  **CAATCTTTTCCTAAAAGGATTGAGCCGAAT------ACAAAGATTCTCTTTTTTTTTT------------** 94

**Hoya longifolia NC_069560.1**  **CAATCTTTTCCTAAAAGGATTGAGCCGAAT------ACAAAGATTCTATTTTTTTTTTT-----------** 95

**Hoya lyi MW719055.1**  **CAATCTTTTCCTAAAAGGATTGAGCCGAAT------ACAAAGATTCTATTTTGTTTTTTAT---------** 97

**Hoya megalaster MW719063.1**  **CAATCTTTTCCTAAAAGGATTGAGCCGAAT------ACAAAGATTCTATTTTTTTTTTT-----------** 1523

**Hoya meliflua NC_069571.1**  **CAATCTTTTCCTAAAAGGATTGAGCCGAAT------ACAAAGATTCTTTTTTTTTTT-------------** 93

**Hoya monetteae MW719053.1**  **CAATCTTTTCCTAAAAGGATTGAGCCGAAT------ACAAAGATTCTATTTTTTTTTTT-----------** 95

**Hoya omlorii MW719060.1**  **CAATCTTTTCCTAAAAGGATTGAGCCGAAT------ACAAAGATTCTTTTTTTTTTTTT-----------** 1523

**Hoya ovalifolia NC_069563.1**  **CAATCTTTTCCTAAAAGGATTGAGCCGAATACAAATACAAAGATTCTTTTTTTTTTT------------T** 100

**Hoya pandurata NC_069562.1**  **CAATCTTTTCCTAAAAGGATTGAGCCGAAT------ACAAAGATTCTATTTTTTTTTTAT----------** 96

**Hoya pottsii OL754664.1**  **CAATCTTTTCCTAAAAGGATTGAGCCGAAT------ACAAAGATTCTTTTTTTTTTT------------T** 94

**Hoya pubicalyx NC_069561.1**  **CAATCTTTTCCTAAAAGGATTGAGCCGAAT------ACAAAGATTCTCTTTTTTTTT-------------** 93

**Hoya radicalis NC_067961.1**  **CAATCTTTTCCTAAAAGGATTGAGCCGAAT------ACAAAGATTCTATTTTTTTTTTTAT---------** 97

**Hoya rigida NC_067962.1**  **CAATCTTTTCCTAAAAGGATTGAGCCGAAT------ACAAAGATTCTATTTTTTTTT-------------** 93

**Hoya silvatica NC_067963.1**  **CAATCTTTTCCTAAAAGGATTGAGCCGAAT------ACAAAGATTCTTTTTTTTTTT------------T** 94

**Hoya thomsonii NC_067612.1**  **CAATCTTTTCCTAAAAGGATTGAGCCGAAT------ACAAAGATTCTATTTTTTTTTTATTTAATTATTT** 106

**Hoya verticillata NC_085236.1**  **CAATCTTTTCCTAAAAGGATTGAGCCGAAT------ACAAAGATTCTTTTTTTTTTT------------T** 94

**Papuahoya urniflora MW719062.1** **CAATCTTTTCCTAAAAGGATTGAGCCGAAT------ACAAAGATTCCATTTTTTTTTAT-----------** 95

**Stephanotis volubilis OP133576** **CAATCTTTTCCTAAAAGGATTGAGCCGAAT------ACAAAGATTCTATTTTTTTTT------------T** 94

**1550 1560 1570 1580 1590 1600 1610**

**....|....|....|....|....|....|....|....|....|....|....|....|....|....|**

**Dischidia australis NC_067885.** **------------------------TTA--ATTATTTATTAA---------GTATTTAATATT-------A** 122

**Gymnema yunnanense NC_079598.1** **TTTA----------------------TTAATTATTTTTTAA---------GTATTTAATATT-------A** 137

**Hoya ariadna NC_069568.1**  **------------------------ATTTCATTATTTTTTAAA-------AGTATTTAATATT-------A** 126

**Hoya commutata NC_067958.1**  **--------------------------TTAATTATTTTTTA------------------------------** 109

**Hoya dimorpha NC_067959.1**  **------------------------ATTTAATTATTTTTTAA---------GTATTTAATATT-------A** 125

**Hoya exilis MW719054.1**  **--------------------------ATTATTATTTTTTAT------TTCAAATCTTATATC-------T** 128

**Hoya griffithii NC_069565.1**  **------------------------ATTTAATTATTTTTTAA---------GTATTTAATATT-------A** 125

**Hoya kerrii NC_069570.1**  **-------------------------TTTAATTATTTTTTAA---------GTATTTAATATT-------A** 123

**Hoya lacunosa NC_069564.1**  **--------------------------TTTA--ATTATTT--------TTTAAGTATT----T-------A** 120

**Hoya lanceolata NC_067960.1**  **------------------------ATTTAATTATTTT--AAA-------AGTATTTAATATT-------A** 124

**Hoya liangii OL826865.1**  **------------------------ATTTAATTATTTTTTAA---------GTATTTAATATT-------A** 126

**Hoya lithophytica MW719058.1**  **------------------------ATTTAATTATTTTTTAAA-------AGTATTTAATATT-------A** 127

**Hoya lockii OR475243.1**  **------------------------ATT--ATTATTTTTTAA---------GTATTTAATATT-------A** 122

**Hoya longifolia NC_069560.1**  **------------------------ATTTAATTATTTTTTAA---------GTATTTAATATT-------A** 125

**Hoya lyi MW719055.1**  **--------------------------TTAATTATTTTTTAA---------GTATTTAATATT-------A** 125

**Hoya megalaster MW719063.1**  **------------------------ATTTAATTATTTTTGAA---------GTATTTAATATT-------A** 1553

**Hoya meliflua NC_069571.1**  **--AT----------------------TTAATTATTTTTTAA---------GTATTTAATATT-------A** 123

**Hoya monetteae MW719053.1**  **------------------------ATTTAATTATTTTTTAA---------GTATTTAATATT-------A** 125

**Hoya omlorii MW719060.1**  **------------------------ATTTAATTATTTTTTAAA-------AGTATTTAATATTTATATTTA** 1562

**Hoya ovalifolia NC_069563.1**  **T-AT----------------------TTAATTATTTTTAAA---------GTATTTAATATT-------A** 131

**Hoya pandurata NC_069562.1**  **--------------------------TTAATTATTTTTTAA---------GTATTTAATATT-------A** 124

**Hoya pottsii OL754664.1**  **TTAT----------------------TTAATTATTTTTTAT---------AT------------------** 115

**Hoya pubicalyx NC_069561.1**  **------------------------ATTTAATTATTTTTTAA---------GTATTTAATATT-------A** 123

**Hoya radicalis NC_067961.1**  **--------------------------TTAATTATTTTTTAA---------GTATTTAATAT---------** 123

**Hoya rigida NC_067962.1**  **------------------------------TTTTTTTTTAA---------GTATTTAATAT---------** 115

**Hoya silvatica NC_067963.1**  **TTAT----------------------TTAATTATTTTTTAT---------AT------------------** 115

**Hoya thomsonii NC_067612.1**  **TTTAAGTATTTAATATTAATAAGTAATTAATAAGTATTTAATATTAATAAGTATTTAATATT-------A** 169

**Hoya verticillata NC_085236.1**  **T-AT----------------------TTAATTATTTTTAAA---------GTATTTAATAT---------** 123

**Papuahoya urniflora MW719062.1** **--------------------------TTAATTATTTTTTAA---------GTATTTAATATT--------** 122

**Stephanotis volubilis OP133576** **TTAT----------------------TTAATTATTTCTTAA---------TTATTTAATATT-------A** 126

**1620 1630 1640 1650 1660 1670 1680**

**....|....|....|....|....|....|....|....|....|....|....|....|....|....|**

**Dischidia australis NC_067885.** **ATA------TATACTTATATAGATATAG---------TCTAGA-TATAGAAGATTTGAAATAAAAAAA--** 174

**Gymnema yunnanense NC_079598.1** **ATATTAATACATACTTATATAGATAT----------------AG---TCTAGATATAG----AAGATTTG** 184

**Hoya ariadna NC_069568.1**  **ATATTAATACATACTTATATAGATATAG---TCTAGATATAGAAGATTTGAAATAAAAAATAAAAAAATC** 193

**Hoya commutata NC_067958.1**  **-TATTAATACATACTTATATAGATAT----------------AAGATTTGAAATAAAA----AAAATCAA** 158

**Hoya dimorpha NC_067959.1**  **ATA------CATACTTATATAGATATAAGATTATATAGATATAAGATTTGAAATAAAAAAGAAAAAAATC** 189

**Hoya exilis MW719054.1**  **ATATAAGTATGTATTAATATTAATATTA--------------AATACTTAAAAAATAATAATAAAAAAAA** 184

**Hoya griffithii NC_069565.1**  **ATA------CATACTTATATAGATATAA----------------GATTTGAAAT----AAAAAAAAAATA** 169

**Hoya kerrii NC_069570.1**  **ATATTAATACATACTTATATAGATAT----------------AAGATTTGAAATAAAA----AAAAATCA** 173

**Hoya lacunosa NC_069564.1**  **ATATTAATACATACTTATATAGATAT----------------AAGATTTGAAAAAAAAAA--AAAAAAAA** 172

**Hoya lanceolata NC_067960.1**  **ATA------CATACTTATATAGATATA----------------AGATTTGAAATAAAAAATCAAAAAATA** 172

**Hoya liangii OL826865.1**  **ATA------CATACTTATATAGATATAA----------------GATTTGAAAT----AAAAAAAAAATA** 170

**Hoya lithophytica MW719058.1**  **ATATTAATACATACTTATATACATATAGTC---TAGATATAGAAGATTTTAAATAAAAAAGAAAAAAATA** 194

**Hoya lockii OR475243.1**  **ATA------CATACTTATATAGATATAGATATAAGATTATATA-GATATAAGATTTGAAATAAAAAAATC** 185

**Hoya longifolia NC_069560.1**  **ATA------CATACTTATATAGATATAA----------------GATTTGAAAT----AAAAAAAAAATA** 169

**Hoya lyi MW719055.1**  **ATATTAATACATACTTATATAGATAT----------------AAGATTTGAAATAAAA----AAAAAAAA** 175

**Hoya megalaster MW719063.1**  **ATATTAATACATACTTATATAGATATAAGATTATATAGATATAAGATTTGAAATAAAAAAGAAAAAAATC** 1623

**Hoya meliflua NC_069571.1**  **ATATTAATACATACTTATATAGATAT----------------AAGATTTGAAATAAAA----AAAA--TC** 171

**Hoya monetteae MW719053.1**  **ATA------CATACTTATATAGATATAA----------------GATTTGAAAT----AAAAAAAAATCA** 169

**Hoya omlorii MW719060.1**  **ATATTAATACATACTTATATAGATATAG---TCTAGATATAGAAGATTTGAAAT---AAAAAAAAAAATC** 1626

**Hoya ovalifolia NC_069563.1**  **ATATTAATACATACTTATATAGATAT----------------AAGATTTGAAATAAAA----AAAAA-TC** 180

**Hoya pandurata NC_069562.1**  **A------TACATACTTATATAGATAT----------------AAGATTTGAAATAAAA----AAAAAATC** 168

**Hoya pottsii OL754664.1**  **----TAATACATACTTATATAGATAT----------------AAGATTTGAAATAAAA----AAAA--TC** 159

**Hoya pubicalyx NC_069561.1**  **ATATTAATACATACTTATATAGATATAA----------------GATTTGAAAT----AAAAAAAAATAA** 173

**Hoya radicalis NC_067961.1**  **----TAATACATACTTATATAGATAT----------------AAGATTTGAAATAAAA----AAAAAATC** 169

**Hoya rigida NC_067962.1**  **----TAATACATACTTATATAGATAT----------------AAGATTTGAAATAAAA----AAA--ATC** 159

**Hoya silvatica NC_067963.1**  **----TAATACATACTTATATAGATAT----------------AAGATTTGAAATAAAA----AAAA--TC** 159

**Hoya thomsonii NC_067612.1**  **ATATTAATACATACTTATATAGATAT----------------AAGATTTGAAATAAAA----AAAAAATC** 219

**Hoya verticillata NC_085236.1**  **----TAATACATACTTATATAGATAT----------------AAGATTTGAAATAAAA----AAAAAATC** 169

**Papuahoya urniflora MW719062.1** **-----AATACATACTTATATAGATAT-------------------AGTCTAGATATAG----AAGATTTG** 164

**Stephanotis volubilis OP133576** **ATATTAATACATACTTATATAGATAT----------------AG---TCTAGATATAG----AAGATTTG** 173

**1690 1700 1710 1720 1730 1740 1750**

**....|....|....|....|....|....|....|....|....|....|....|....|....|....|**

**Dischidia australis NC_067885.** **-AA---AAAATC--------TAAGGCTCAAATCTTTCTATTGTT-GTCTTGGATCTACAATTAATCTACG** 231

**Gymnema yunnanense NC_079598.1** **AAATACAAAATC--------TAAGGCTCAAATCTTTCTATTGTT-GTCTTGGATCTACAATTAATCTACG** 245

**Hoya ariadna NC_069568.1**  **AAA---AAAATC--------TAAGGCTCAAATCTTTCTATTGTT-GTCTTGGATCTACAATTAATCTACG** 251

**Hoya commutata NC_067958.1**  **-----AAAAATC--------TAA---------CTTTCTATTGTT-GTCTTGGATCTACAATTAATCTACG** 205

**Hoya dimorpha NC_067959.1**  **AAA---AAAATC--------TAAGGCTCAAATCTTTCTATTGTT-GTCTTGGATCTACAATTAATCTACG** 247

**Hoya exilis MW719054.1**  **AATCAAAAAATC--------TAAGGCTCAAATCTTTCTATTGTT-GTCTTGGATCTACAATTAATCTACG** 245

**Hoya griffithii NC_069565.1**  **AAT---AAAATC--------TAAGGCTCAAATCTTTCTATTGTT-GTCTTGGATCTACAATTAATCTACG** 227

**Hoya kerrii NC_069570.1**  **AAA----AAATA--------TAAGGCTCAAATCTTTCTATTGTT-GTCTTGGACCTACAATTAATCTACG** 230

**Hoya lacunosa NC_069564.1**  **AATCAAAAAATC--------TAAGGCTCAAATTTTTCTATTGTT-GTCTTGGATCTACAATTAATCTACG** 233

**Hoya lanceolata NC_067960.1**  **AAA-AAAAAATC--------TAAGGCTCAAATCTTTCTATTGTT-GTCTTGGATCTACAATTAATCTAC-** 231

**Hoya liangii OL826865.1**  **AAT---AAAATCATAAAATCTAAGGCTCAAATCTTTCTATTGTT-GTCTTGGATCTACAATTAATCTACG** 236

**Hoya lithophytica MW719058.1**  **AAA-AAA--ATC--------TAAGGCTCAAATCTTTATATTGTT-GTCTTGGATCTACAATTAATCTACG** 252

**Hoya lockii OR475243.1**  **AAA---AAAATC--------TAAGGCTCAAATCTTTCTATTGTT-GTCTTGGATCTACAATTAATCTACG** 243

**Hoya longifolia NC_069560.1**  **AAT---AAAATC--------TAAGGCTCAAATCTTTCTATTGTT-GTCTTGGATCTACAATTAATCTACG** 227

**Hoya lyi MW719055.1**  **TAAAAAAAAATC--------TAAGGCTCAAATCTTTCTATTGTT-GTCTTGGATCTACAATTAATCTACG** 236

**Hoya megalaster MW719063.1**  **AAA---AAAATC--------TAAGGCTCAAATCTTTCTATTGTT-GTCTTGGATCTACAATTAATCTACG** 1681

**Hoya meliflua NC_069571.1**  **AAA---AAAATC--------TAAGGCTCAAATCTTTCTATTGTT-GTCTTGGACCTACAATTAATCTACG** 229

**Hoya monetteae MW719053.1**  **AA----AAAATC--------TAAGGCTCAAATCTTTCTATTGTT-GTCTTGGATCTACAATTAATCTACG** 226

**Hoya omlorii MW719060.1**  **AAA---AAAATC--------TAAGGCTCAAATCTTTCTATTGTT-GTCTTGGATCTACAATTAATCTACG** 1684

**Hoya ovalifolia NC_069563.1**  **AAA---AAAATC--------TAAGGCTCAAATCTTTCTATTGTT-GTCTTGGACCTACAATTAATCTACG** 238

**Hoya pandurata NC_069562.1**  **AAA---AAAATC--------TAAGGCTCAAATCTTTCTATTGTT-GTCTTGGATCTACAATTAATCTACG** 226

**Hoya pottsii OL754664.1**  **AAA---AAAATC--------TAAGGCTCAAATCTTTCTATTGTTTGTCTTGGATCTACAATTAATCTACG** 218

**Hoya pubicalyx NC_069561.1**  **AAA---AAAATC--------TAAGGCTCAAATCTTTCTATTGTT-GTCTTGGATCTACAATTAATCTACG** 231

**Hoya radicalis NC_067961.1**  **AAA---AAAATC--------TAAGGCTCAAATCTTTCTATTGTT-GTCTTGGATCTACAATTAATCTACG** 227

**Hoya rigida NC_067962.1**  **AAA---AAAATC--------TAAGGCTCAAATCTTTCTATTGTT-GTCTTGGATCTACAATTAATCTACG** 217

**Hoya silvatica NC_067963.1**  **AAA---AAAATC--------TAAGGCTCAAATCTTTCTATTGTTTGTCTTGGATCTACAATTAATCTACG** 218

**Hoya thomsonii NC_067612.1**  **AAA---AAAATC--------TAAGGCTCAAATCTTTCTATTGTT-GTCTTGGATCTACAATTAATCTACG** 277

**Hoya verticillata NC_085236.1**  **AAA---AAAATC--------TAAGGCTCAAATCTTTCTATTGTT-GTCTTGGACCTACAATTAATCTACG** 227

**Papuahoya urniflora MW719062.1** **AAATAAAAAATAAAAAAATCTAAGGCTCAAATCTTTCTATTGTT-GTCTTGGATCTACAATTAATCTACG** 233

**Stephanotis volubilis OP133576** **AAATACAAAATC--------TAAGGCTCAAACCTTTCTATTGTT-GTCTTGGATCTACAATTAATCTACG** 234

**1760 1770 1780 1790 1800 1810 1820**

**....|....|....|....|....|....|....|....|....|....|....|....|....|....|**

**Dischidia australis NC_067885.** **GATC------CTTGGGATGGGGCTATTCTTT----TATATCCTGCAGTTTTCCTGAATCAAACCAAGTAT** 291

**Gymnema yunnanense NC_079598.1** **GATC------CTTGGGATTGGTCTATTCTTT----TATATCCTGCAGTTTTCCTGAATCAAGCCAAGTAT** 305

**Hoya ariadna NC_069568.1**  **GATC------CTTGGGATTGGGCTATTCTTT----TATATCCTGCAGTTTTCCTGAATCAAGCCAAGTAT** 311

**Hoya commutata NC_067958.1**  **GATC------CTTGGGATTGGGCTATTCTTT----TATATCCTGCAGTTTTCCTGAATCAAGCCAAGTAT** 265

**Hoya dimorpha NC_067959.1**  **GATC------CTTGGGATTGGGCTATTCTTT----TATATCCTGCAGTTTTCCTGAATCAAGCCAAGTAT** 307

**Hoya exilis MW719054.1**  **GATC------CTTGGGATTGGGCTATTCTTT----TATATCCTGCAGTTTTCCTGAATCAAGCCAAGTAT** 305

**Hoya griffithii NC_069565.1**  **GATC------CTTGGGATTGGGCTATTCTTT----TATATCCTGCAGTTTTCCTGAATCAAGCCAAGTAT** 287

**Hoya kerrii NC_069570.1**  **GATC------CTTGGGATTGGGCTATTCTTT----TATATCCTGCAGTTTTCCTGAATCAAGCCAAGTAT** 290

**Hoya lacunosa NC_069564.1**  **GATC------CTTGGGATTGGGCTATTCTTT----TATATCCTGCAGTTTTCCTGAATCAAGCCAAGTAT** 293

**Hoya lanceolata NC_067960.1**  **----------------------------------------------------------------------** 231

**Hoya liangii OL826865.1**  **GATC------CTTGGGATTGGGCTATTCTTT----TATATCCTGCAGTTTTCCTGAATCAAGCCAAGTAT** 296

**Hoya lithophytica MW719058.1**  **GATC------CTTGGGATTGGGCTATTCTTT------TATATCCTGCAGTTTTCCTGAATCAAGCCAAGT** 310

**Hoya lockii OR475243.1**  **GATCTTTGGGCTTGGGATTGGGCTATTCTTTCTTTTATATCCTGCAGTTTTACTGAATCAAGCCAAGTAT** 313

**Hoya longifolia NC_069560.1**  **GATC------CTTGGGATTGGGCTATTCTTT----TATATCCTGCAGTTTTCCTGAATCAAGCCAAGTAT** 287

**Hoya lyi MW719055.1**  **GATC------CTTGGGATTGGGCTATTCTTT----TATATCCTGCAGTTTTCCTGAATCAAGCCAAGTAT** 296

**Hoya megalaster MW719063.1**  **GATC------CTTGGGATTGGGCTATTCTTT----TATATCCTGCAGTTTTCCTGAATCAAGCCAAGTAT** 1741

**Hoya meliflua NC_069571.1**  **GATC------CTTGGGATTGGGCTATTCTTT----TATATCCTGCAGTTTTCCTGAATCAAGCCAAGTAT** 289

**Hoya monetteae MW719053.1**  **GATC------CTTGGGATTGGGCTATTCTTT----TATATCCTGCAGTTTTCCTGAATCAAGCCAAGTAT** 286

**Hoya omlorii MW719060.1**  **GATC------CTTGGGATTGGGCTATTCTTT----TATATCCTGCAGTTTTCCTGAATCAAGCCAAGTAT** 1744

**Hoya ovalifolia NC_069563.1**  **GATC------CTTGGGATTGGGCTATTCTTT----TATATCCTGCAGTTTTCCTGAATCAAGCCAAGTAT** 298

**Hoya pandurata NC_069562.1**  **GATC------CTTGGGATTGGGCTATTCTTT----TATATCCTGCAGTTTTCCTGAATCAAGCCAAGTAT** 286

**Hoya pottsii OL754664.1**  **GATC------CTTGGGATTGGGCTATTCTTT----TATATCCTGCAGTTTTCCTGAATCAAGCCAAGTAT** 278

**Hoya pubicalyx NC_069561.1**  **GATC------CTTGGGATTGGGCTATTCTTT----TATATCCTGCAGTTTTCCTGAATCAAGCCAAGTAT** 291

**Hoya radicalis NC_067961.1**  **GATC------CTTGGGATTGGGCTATTCTTT----TATATCCTGCAGTTTTCCTGAATCAAGCCAAGTAT** 287

**Hoya rigida NC_067962.1**  **GATC------CTTGGGATTGGGCTATTCTTT----TATATCCTGCAGTTTTCCTGAATCAAGCCAAGTAT** 277

**Hoya silvatica NC_067963.1**  **GATC------CTTGGGATTGGGCTATTCTTT----TATATCCTGCAGTTTTCCTGAATCAAGCCAAGTAT** 278

**Hoya thomsonii NC_067612.1**  **GATC------CTTGGGATTGGGCTATTCTTT----TATATCCTGCAGTTTTCCTGAATCAAGCCAAGTAT** 337

**Hoya verticillata NC_085236.1**  **GATC------CTTGGGATTGGGCTATTCTTT----TATATCCTGCAGTTTTCCTGAATCAAGCCAAGTAT** 287

**Papuahoya urniflora MW719062.1** **GATC------CTTGGGATTGGGCTATTCTTT----TATATCCTGCAGTTTTCCTGAATCAAGCCAAGTAT** 293

**Stephanotis volubilis OP133576** **GATC------CTTGGGATTGGTCTATTCTTT-----ATATCCTGCAGTTTTCCTGAATCAAGCCAAGTAT** 293

**1830 1840 1850 1860 1870 1880 1890**

**....|....|....|....|....|....|....|....|....|....|....|....|....|....|**

**Dischidia australis NC_067885.** **CAC---AATTCTTTCTACCCCATCTTGTATATTGTCCTTTTCTTTCCATATTG-------GTGAAATAGA** 351

**Gymnema yunnanense NC_079598.1** **CAC---AATTCTTTCTACCC-ATCCTGTATATTGTCCTTTTCTTTCCATATTGCATATTGGTGGAATAGA** 371

**Hoya ariadna NC_069568.1**  **CAC---AATTCTTTCTACCC-ATCCTGTATATTGTCCTTTTCTTTCCATATTG-------GTGAAATAGA** 370

**Hoya commutata NC_067958.1**  **CAC---AATTCTTTCTACCC-ATCCCGTATATTGTCCTTTTCTTTCCATATTG-------GTGAAATAGA** 324

**Hoya dimorpha NC_067959.1**  **CACCACAATTCTTTCTACCC-ATCCTGTATATTGTCCTTTTCGTTCCATATTG-------GTGAAATAGA** 369

**Hoya exilis MW719054.1**  **CAC---AATTCTTTCTACCC-ATCCTGTATATTGTCCTTTTCTTTCCATATTG-------GTGAAATAGA** 364

**Hoya griffithii NC_069565.1**  **CAC---AATTCTTTCTACCC-ATCCTGTATATTGTCCTTTTCTTTCCATATTG-------GTGAAATAGA** 346

**Hoya kerrii NC_069570.1**  **CAC---AATTCTTTCTACCC-ATCCCGTATATTGTCCTTTTCTTTCCATAT-------TGGTGAAATAGA** 349

**Hoya lacunosa NC_069564.1**  **CAC---AATTCTTTCTACCC-ATCCCGTATATTGTCCTTTTCTTTCCATATTG-------GTGAAATAGA** 352

**Hoya lanceolata NC_067960.1**  **----------------------------------------------------------------------** 231

**Hoya liangii OL826865.1**  **CAC---AATTCTTTCTACCC-ATCCTGTATATTGTCCTTTTCTTTCCATATTG-------GTGAAATAGA** 355

**Hoya lithophytica MW719058.1**  **ATCACAATTCTTTCTACCCATCCTGTATATTGTCCTTTTCTTTCCATATTGGT---------GAAATAGA** 371

**Hoya lockii OR475243.1**  **CAC---AATTCTTTCTACCC-ATCCTGTATATTGTCCTTTTCTTTCCATATTG-------GTGAAATAGA** 372

**Hoya longifolia NC_069560.1**  **CAC---AATTCTTTCTACCC-ATCCTGTATATTGTCCTTTTCTTTCCATATTG-------GTGAAATAGA** 346

**Hoya lyi MW719055.1**  **CAC---AATTCTTTCTACCC-ATCCCGTATATTGTCCTTTTCTTTCCATATTG-------GTGAAATAGA** 355

**Hoya megalaster MW719063.1**  **CACCACAATTCTTTCTACCC-ATCCTGTATATTGTCCTTTTCTTTCCATATTG-------GTGAAATAGA** 1803

**Hoya meliflua NC_069571.1**  **CAC---AATTCTTTCTACCC-ATCCCGTATATTGTCCTTTTCTTTCCATATTG---GTTGGTGAAATAGA** 352

**Hoya monetteae MW719053.1**  **CGC---AATTCTTTCTACCC-ATCCCGTATATTGTCCTTTTCTTTCCATATTG-------GTGAAATAGA** 345

**Hoya omlorii MW719060.1**  **CAC---AATTCTTTCTACCC-ATCCTGTATATTGTCCTTTTCTTTCCATATTG-------GTGAAATAGA** 1803

**Hoya ovalifolia NC_069563.1**  **CAC---AATTCTTTCTACCC-ATCCCGTATATTGTCCTTTTCTTTCCATATTG---GT----GAAATAGA** 357

**Hoya pandurata NC_069562.1**  **CAC---AATTCTTTCTACCC-ATCCTGTATATTGTCCTTTTCTTTCCATAT-------TGGTGAAATAGA** 345

**Hoya pottsii OL754664.1**  **CAC---AATTCTTTCTACCC-ATCCCGTATATTGTCCTTTTCTTTCCATATTG---GT----GAAATAGA** 337

**Hoya pubicalyx NC_069561.1**  **CAC---AATTCTTTCTACCC-ATCCCGTATATTGTCCTTTTCTTTCCATATTG-------GTGAAATAGA** 350

**Hoya radicalis NC_067961.1**  **CAC---AATTCTTTCTACCC-ATCCTGTATATTGTCCTTTTCTTTCCATATTG-------GTGAAATAGA** 346

**Hoya rigida NC_067962.1**  **CAC---AATTCTTTCTACCC-ATCCTGTATATTGTCCTTTTCTTTCCATATTG-------GTGAAATAGA** 336

**Hoya silvatica NC_067963.1**  **CAC---AATTCTTTCTACCC-ATCCCGTATATTGTCCTTTTCTTTCCATATTG---GT----GAAATAGA** 337

**Hoya thomsonii NC_067612.1**  **CAC---AATTCTTTCTACCC-ATCCTGTATATTGTCCTTTTCTTTCCATAT-------TGGTGAAATAGA** 396

**Hoya verticillata NC_085236.1**  **CAC---AATTCTTTCTATCC-ATCCCGTATATTGTCCTTTTCTTTCCATATTG---GT----GAAATAGA** 346

**Papuahoya urniflora MW719062.1** **CAC---AATTCTTTCTACCC-ATCCTGTATATTGTCCTTTTCTTTCCATATTG-------GTGAAATAGA** 352

**Stephanotis volubilis OP133576** **CAC---AATTCTTTCTACCC-ATCCTGTATATTGTCCTTTTCTTTCCATAT-------TGGTGGAATAGA** 352

**1900 1910 1920 1930 1940 1950 1960**

**....|....|....|....|....|....|....|....|....|....|....|....|....|....|**

**Dischidia australis NC_067885.** **ACCTGAAATTTTTACGTTCTTGGGCGAAATTTTACGAAAAAAA-GGATTTATAAAATATTTTTTTT-CGA** 419

**Gymnema yunnanense NC_079598.1** **ACCTGAAATTATTACGTTCTTAGGCGAAATTTTACGAAAAAAA-AGATTTCTAAAATCTTTTTTTT-CGA** 439

**Hoya ariadna NC_069568.1**  **ACCTGAAATTTTTACGTTCTTGGGCGAAATTTTACGAAAAAAA-GGATTTATAAAATATTTTTTTT-CGA** 438

**Hoya commutata NC_067958.1**  **ACCTGAAATTTTTACGTTCTTGGGCGAAATTTTACGAAAAAAA-GGATTTATAAAATATTTTTTTT-CGA** 392

**Hoya dimorpha NC_067959.1**  **ACCTGAAATTTTTACGTTCTTGGGCGAAATTTTACGAAAAAAA-GGATTTATAAAATATTTTTTTT-CGA** 437

**Hoya exilis MW719054.1**  **ACCTGAAATTTTTACGTTCTTGGGCGAAATTTTACGAAAAAAA-GGATTTATAAAATATTTTTTTT-CGA** 432

**Hoya griffithii NC_069565.1**  **ACCTGAAATTTTTACGTTCTTGGGCGAAATTTTACGAAAAAAA-GGATTTATAAAATATTTTTTTT-CGA** 414

**Hoya kerrii NC_069570.1**  **ACCTGAAATTTTTACGTTCTTGGGCGAAATTTTACGAAAAAAA-GGATTTATAAAATATTTTTTTTTCGA** 418

**Hoya lacunosa NC_069564.1**  **ACCTGAAATTTTTACG--------CGAAATTTTACGAAAAAAA-GGATTTATAAAATATTTTTTTT-CGA** 412

**Hoya lanceolata NC_067960.1**  **------------------CTTGGGCGAAATTTTACGAAAAAAA-GGATTTATAAAATATTTTTTTT-CGA** 281

**Hoya liangii OL826865.1**  **ACCTGAAATTTTTACGTTCTTGGGCGAAATTTTACGAAAAAAA-GGATTTATAAAATATTTTTTTT-CGA** 423

**Hoya lithophytica MW719058.1**  **ACCTGAAACTTTTACGTTCTTGGGCGAAATTTTACCAATAAAA-GGATTTATAAAATATTTTTTTT-CGA** 439

**Hoya lockii OR475243.1**  **ACCTGAAATTTTTACGTTCTTGGGCGAAATTTTA-----------GATTTATAAAATATTTTTTTT-CGA** 430

**Hoya longifolia NC_069560.1**  **ACCTGAAATTTTTACGTTCTTGGGCGAAATTTTACGAAAAAAA-GGATTTATAAAATATTTTTTTT-CGA** 414

**Hoya lyi MW719055.1**  **ACCTGAAATTTTTACGTTCTTGGGCGAAATTTTACGAAAAAAA-GGATTTATAAAATATTTTTTTT-CGA** 423

**Hoya megalaster MW719063.1**  **ACCTGAAATTTTTACGTTCTTGGGCGAAATTTTACGAAAAAAA-GGATTTATAAAATATTTTTTTT-CGA** 1871

**Hoya meliflua NC_069571.1**  **ACCTGAAATTTTTACGTTCTTGGGCGAAATTTTACGAAAAAAA-GGATTTATAAAATATTTTTTTT-CGA** 420

**Hoya monetteae MW719053.1**  **ACCTGAAATTTTTACGTTCTTGGGCGAAATTTTACGAAAAAAAAGGATTTATAAAATATTTTTTTT-CGA** 414

**Hoya omlorii MW719060.1**  **ACCTGAAATTTTTACGTTCTTGGGCGAAATTTTACGAAAAAAA-GGATTTATAAAATATTTTTTTT-CGA** 1871

**Hoya ovalifolia NC_069563.1**  **ACCTGAAATTTTTACGTTCTTGGGCGAAATTTTACGAAAAAAA-GGATTTATAAAATATTTTTTTT-CGA** 425

**Hoya pandurata NC_069562.1**  **ACCTGAAATTTTTACGTTCTTGGGCGAAATTTTACGAAAAAAA-GGATTTATAAAATATTTTTTTT-CGA** 413

**Hoya pottsii OL754664.1**  **ACCTGAAATTTTTACGTTCTTGGGCGAAATTTTACGAAAAAAA-GGATTTATAAAATATTTTTTTT-CGA** 405

**Hoya pubicalyx NC_069561.1**  **ACCTGAAATTTTTACGTTCTTGGGCGAAATTTTACTAAAAAAA-GGATTTATAAAATATTTTTTTT-CG-** 417

**Hoya radicalis NC_067961.1**  **ACCTGAAATTTTTACGTTCTTGGGCGAAATTTTACGAAAAAAA-GGATTTATAAAATATTTTTTTT-CGA** 414

**Hoya rigida NC_067962.1**  **ACCTGAAATTTTTACGTTCTTGGGCGAAATTTTACGAAAAAAAAGGATTTATAAAATATTTTTTTT-CGA** 405

**Hoya silvatica NC_067963.1**  **ACCTGAAATTTTTACGTTCTTGGGCGAAATTTTACGAAAAAAA-GGATTTATAAAATATTTTTTTT-CGA** 405

**Hoya thomsonii NC_067612.1**  **ACCTGAAATTTTTACGTTCTTGGGCGAAATTTTACGAAAAAAA-GGATTTATAAAATATTTTTTTT-CGA** 464

**Hoya verticillata NC_085236.1**  **ACCTGAAATTTTTACGTTCTTGGGCGAAATTTTACGAAAAAAA-GGATTTATAAAATATTTTTTTT-CGA** 414

**Papuahoya urniflora MW719062.1** **ACCTGAAATTTTTACGTTCTTGGGCGAAATTTTACGAAAAAAA-GGATTTATTAAATATTTTTTTTTCGA** 421

**Stephanotis volubilis OP133576** **ACCTGAAATTATTACGTTCTTAGGCGAAATTTTACGAAAAAAT--GATTTCTAAAATATTTTTTTT-CGA** 419

**1970 1980 1990 2000 2010 2020 2030**

**....|....|....|....|....|....|....|....|....|....|....|....|....|....|**

**Dischidia australis NC_067885.** **TGCGAATTTGATACGACATAAGAAAAGGCGCTCTTTATCATTGTATTTATAATGT---------------** 474

**Gymnema yunnanense NC_079598.1** **TGCGAATTTGATACGACATAAGAAAAAGCGCTCTTTATCATTGTATTTATAATGACAAGAGATTCCCTGA** 509

**Hoya ariadna NC_069568.1**  **TGCGAATTTGATACGACATAAGAAAAGGCGCTCTTTATCATTGTATTTATAATGACAAGAGATTCCCTGA** 508

**Hoya commutata NC_067958.1**  **TGCGAATTTGATACGACATAAGAAAAGGCGCTCTTTATCATTGTATTTATAATGACAAGAGATTCCCTGA** 462

**Hoya dimorpha NC_067959.1**  **TGCGAATTTGATACGACATAAGAAAAGGCGCTCTTTATCATTGTATTTATAATGACAAGAGATTCCCTGA** 507

**Hoya exilis MW719054.1**  **TGCGAATTTGATACGACATAAGAAAAGGCGCTCTTTATCATTGTATTTATAATGACAAGAGATTCCCTGA** 502

**Hoya griffithii NC_069565.1**  **TGCGAATTTGATACGACATAAGAAAAGGCGCTCTTTATCATTGTATTTATAATGACAAGAGATTCCCTGA** 484

**Hoya kerrii NC_069570.1**  **TGCGAATTTCATACGACATAAGAAAAGGCGCTCTTTATCATTGTATTTATAATGACAAGAGATTCCCTGA** 488

**Hoya lacunosa NC_069564.1**  **TGCGAATTTGATACGACATAAGAAAGGGCGCTCTTTATCATTGTATTTATAATGACAAGAGATTCCCTGA** 482

**Hoya lanceolata NC_067960.1**  **TGCGAATTTGATACGACATAAGAAAAGGCGCTCTTTATCATTGTATTTATAATGACAAGAGATTCCCTGA** 351

**Hoya liangii OL826865.1**  **TGCGAATTTGATACGACATAAGAAAAGGCGCTCTTTATCATTGTATTTATAATGACAAGAGATTCCCTGA** 493

**Hoya lithophytica MW719058.1**  **TGCGAATTTGATACGACATAAGAAAAGGCGCTCTTTATCATTGTATTTATAATGACAAGAGATTCCCTGA** 509

**Hoya lockii OR475243.1**  **TGCGAATTTGATACGACATAAGAAAAGGCGCTCTTTATCATTGTATTTATAATGACAAGAGATTACCTGA** 500

**Hoya longifolia NC_069560.1**  **TGCGAATTTGATACGACATAAGAAAAGGCGCTCTTTATCATTGTATTTATAATGACAAGAGATTCCCTGA** 484

**Hoya lyi MW719055.1**  **TGCGAATTTGATACGACATAAGAAAAGGCGCTCTTTATCATTGTATTTATAATGACAAGAGATTCCCTGA** 493

**Hoya megalaster MW719063.1**  **TGCGAATTTGATACGACATAAGAAAAGGCGCTCTTTATCATTGTATTTATAATGACAAGAGATTCCCTGA** 1941

**Hoya meliflua NC_069571.1**  **TGCGAATTTGATACGACATAAGAAAAGGCGCTCTTTATCATTGTATTTATAATGACAAGAGATTCCCTGA** 490

**Hoya monetteae MW719053.1**  **TGCGAATTTGATACGACATAAGAAAAGGCGCTCTTTATCATTGTATTTATAATGACAAGAGATTCCCTGA** 484

**Hoya omlorii MW719060.1**  **TGCGAATTTGATACGACATAAGAAAAGGCGCTCTTTATCATTGTATTTATAATGACAAGAGATTCCCTGA** 1941

**Hoya ovalifolia NC_069563.1**  **TGCGAATTTGATACGACATAAGAAAAGGCGCTCTTTATCATTGTATTTATAATGACAAGAGATTCCCTGA** 495

**Hoya pandurata NC_069562.1**  **TGCGAATTTGATACGACATAAGAAAAGGCGCTCTTTATCATTGTATTTATAATGACAAGAGATTCCCTGA** 483

**Hoya pottsii OL754664.1**  **TGCGAATTTGATACGACATAAGAAAAGGCGCTCTTTATCATTGTATTTATAATGACAAGAGATTCCCTGA** 475

**Hoya pubicalyx NC_069561.1**  **------TTTGATACGACATAAGAAAAGGCGCTCTTTATCATTGTATTTATAATGACAAGAGATTCCCTGA** 481

**Hoya radicalis NC_067961.1**  **TGCGAATTTGATACGACATAAGAAAAGGCGCTCTTTATCATTGTATTTATAATGACAAGAGATTCCCTGA** 484

**Hoya rigida NC_067962.1**  **TGCGAATTTGATACGACATAAGAAAAGGCGCTCTTTATCATTGTATTTATAATGACAAGAGATTCCCTGA** 475

**Hoya silvatica NC_067963.1**  **TGCGAATTTGATACGACATAAGAAAAGGCGCTCTTTATCATTGTATTTATAATGACAAGAGATTCCCTGA** 475

**Hoya thomsonii NC_067612.1**  **TGCGAATTTGATACGACATAAGAAAAGGCGCTCTTTATCATTGTATTTATAATGACAAGAGATTCCCTGA** 534

**Hoya verticillata NC_085236.1**  **TGCGAATTTGATACGACATAAGAAAAGGCGCTCTTTATCATTGTATTTATAATGACAAGAGATTCCCTGA** 484

**Papuahoya urniflora MW719062.1** **TGCGAATTTGATACGACATAAGAAAAGGCGCTCTTTATCATTGTATTTATAATGACAAGGGATTCCCTGA** 491

**Stephanotis volubilis OP133576** **TGCGAATTTGATACGACATAAGAAAAAGCGCTCTTTATCATTGTATTTATAATGACAAGAGATTCCCTGA** 489

**2040 2050 2060 2070 2080 2090 2100**

**....|....|....|....|....|....|....|....|....|....|....|....|....|....|**

**Dischidia australis NC_067885.** **-----TATAATGACAAGGGATTCCC--CATTCAAAAAATAAT----------------------------** 509

**Gymnema yunnanense NC_079598.1** **TATCA-----TATT--------------------------------------------------------** 518

**Hoya ariadna NC_069568.1**  **-----TATGATATTAATATATTGACAGTATTCATAAAATAATGTATA-----------------------** 550

**Hoya commutata NC_067958.1**  **TATAATATCATATTAATATATTGACAATATTCAAAAAATAATGTATAAT---------------------** 511

**Hoya dimorpha NC_067959.1**  **TATCATATCATATTAATATATTGACAACATTCAAAAAATAAT----------------------------** 549

**Hoya exilis MW719054.1**  **TATCATATCATATTAATATATTGACAGTATTCAAAAAATAATATATAATGTATAAAATG-----------** 561

**Hoya griffithii NC_069565.1**  **TATCATATCATATTAATATATTGACAATATTCAAAAAATAAT----------------------------** 526

**Hoya kerrii NC_069570.1**  **TATCA-----TATTAATATATTGACAATATTCAAAAAATAATGTATAA----------------------** 531

**Hoya lacunosa NC_069564.1**  **TATCATATCATATTAATATATTGACATTATTCAAAAAATAATGTATAATGTATAAAA-------------** 539

**Hoya lanceolata NC_067960.1**  **TATCA-----TATTAATATATTGACAATATTCAAAAAATAATGTATAAAGATAATGACAAGGGATTCCCT** 416

**Hoya liangii OL826865.1**  **TATCATATCATATTAATATATTGACAATATTCAAAAAATAAT----------------------------** 535

**Hoya lithophytica MW719058.1**  **TATCA-----TATTAATATATTGACAATATTCAAAAAATAATGTATAAAAATAATGACAAGAGATTCCCT** 574

**Hoya lockii OR475243.1**  **-----TATCATATTAATATATTGACAACATTCAAAGAATAATATATATAATATATAAT------------** 553

**Hoya longifolia NC_069560.1**  **TATCATATCATATTAATATATTGACAATATTCAAAAAATAAT----------------------------** 526

**Hoya lyi MW719055.1**  **TATCATATCATATTAATATATTGACAATATTCAAAAAATAATGTATAAAAAAAATGAC------------** 551

**Hoya megalaster MW719063.1**  **-----TATCATATTAATATATTGACAACATTCAAAAAATAATGTATAAAAATAATGACAAGGGATTCCCT** 2006

**Hoya meliflua NC_069571.1**  **TATCA-----TATTAATATATTGACAATATTCAAAAAATAATGTATAAAAAAAATGAC------------** 543

**Hoya monetteae MW719053.1**  **TATCATATCATATTAATATATTGACAATATTCAAAAAATAAT----------------------------** 526

**Hoya omlorii MW719060.1**  **-----TATGATATTAATATATTGACAGTATTCATAAAATAATGTATAATGTATAAAAATAATGACAAGAG** 2006

**Hoya ovalifolia NC_069563.1**  **TATCA-----TATTAATATATTGACAATATTCAAAAAATAATGTATAAAAAAAATGAC------------** 548

**Hoya pandurata NC_069562.1**  **TATCA-----TATCATATTAATATATTGACAATATTCAAAAAATAATG----------------------** 526

**Hoya pottsii OL754664.1**  **TATCA-----TATTAATATATTGACAATATTCAAAAAAAAA-----------------------------** 511

**Hoya pubicalyx NC_069561.1**  **TATCATATCATATTAATATATTAACAATATTCAAAAAATAAT----------------------------** 523

**Hoya radicalis NC_067961.1**  **TATCATATCATATTAATATATTGACAATATTCAAAAAATAATGTATAAAAAAAATGAC------------** 542

**Hoya rigida NC_067962.1**  **TATCA-----TATTAATATATTGACAATATTCAAAAAATAATGTATAAAAAAAATGAC------------** 528

**Hoya silvatica NC_067963.1**  **TATCA-----TATTAATATATTGACAATATTCAAAAAAAAA-----------------------------** 511

**Hoya thomsonii NC_067612.1**  **TATCA-----TATTAATATATTGACAATATTCAAAAAATAATGTATAA----------------------** 577

**Hoya verticillata NC_085236.1**  **TATCA-----TATTAATATATTGACAATATTCAAAAAAAAATGTATAAAAAAAATGAC------------** 537

**Papuahoya urniflora MW719062.1** **TATGA-----TATTAATATATTGACAATATTCAAAAAATAATGTATAA----------------------** 534

**Stephanotis volubilis OP133576** **TATCA-----TATT--------------------------------------------------------** 498

**2110 2120 2130 2140 2150 2160 2170**

**....|....|....|....|....|....|....|....|....|....|....|....|....|....|**

**Dischidia australis NC_067885.** **-----------------------------------------------------GTATAAAAAAAATGACA** 526

**Gymnema yunnanense NC_079598.1** **----------------------------------------------------------------------** 518

**Hoya ariadna NC_069568.1**  **---------------------------------------------------ATGTATAAAAATAATGACA** 569

**Hoya commutata NC_067958.1**  **----------------------------------------------------------------------** 511

**Hoya dimorpha NC_067959.1**  **-----------------------------------------------------GTATAAAAATAATGACA** 566

**Hoya exilis MW719054.1**  **---------------------------------------------------T---ATAAAAAAAATGACA** 577

**Hoya griffithii NC_069565.1**  **-----------------------------------------------------GTATAAAAAAAATGACA** 543

**Hoya kerrii NC_069570.1**  **----------------------------------------------------------------------** 531

**Hoya lacunosa NC_069564.1**  **-------------------------------------------------------------ATAATGACA** 548

**Hoya lanceolata NC_067960.1**  **GATATCA-------TATTAATATATTGACAATATTCAAAAAATAATGTATAA-------AAAGAATGACA** 472

**Hoya liangii OL826865.1**  **-----------------------------------------------------GTATAAAAAAAATGACA** 552

**Hoya lithophytica MW719058.1**  **GATATGAATATGA-TATTAATATATTGACAGTATTCATAAAATAATGTATAATGTATAAAAATAATGACA** 643

**Hoya lockii OR475243.1**  **--------------------------------------------GTATAAAATGTATAAAAATAATGACA** 579

**Hoya longifolia NC_069560.1**  **-----------------------------------------------------GTATAAAAAAAATGACA** 543

**Hoya lyi MW719055.1**  **------------------------------------------------------------AAGGGATTCT** 561

**Hoya megalaster MW719063.1**  **GATATC--ATATCATATTAATATATTGACAGTATTCATAAAATAATGTATAATGTATAAAAATAATGACA** 2074

**Hoya meliflua NC_069571.1**  **------------------------------------------------------------AAGGGATTCT** 553

**Hoya monetteae MW719053.1**  **-----------------------------------------------------GTATAAAAAAAATGACA** 543

**Hoya omlorii MW719060.1**  **ATTCCCTGATATGATATTAATATATTGACAGTATTCATAAAATAATGTATAATGTATAAAAATAATGACA** 2076

**Hoya ovalifolia NC_069563.1**  **------------------------------------------------------------AAGGGATTCT** 558

**Hoya pandurata NC_069562.1**  **----------------------------------------------------------------------** 526

**Hoya pottsii OL754664.1**  **----------------------------------------------------------------------** 511

**Hoya pubicalyx NC_069561.1**  **-----------------------------------------------------GTATAAAAAAAATGACA** 540

**Hoya radicalis NC_067961.1**  **------------------------------------------------------------AAGGGATTCT** 552

**Hoya rigida NC_067962.1**  **------------------------------------------------------------AAGGGATTCT** 538

**Hoya silvatica NC_067963.1**  **----------------------------------------------------------------------** 511

**Hoya thomsonii NC_067612.1**  **----------------------------------------------------------------------** 577

**Hoya verticillata NC_085236.1**  **------------------------------------------------------------AAGGGATTCT** 547

**Papuahoya urniflora MW719062.1** **-----------------------------------------------------------AAATAATGACA** 545

**Stephanotis volubilis OP133576** **----------------------------------------------------------------------** 498

**2180 2190 2200 2210 2220 2230 2240**

**....|....|....|....|....|....|....|....|....|....|....|....|....|....|**

**Dischidia australis NC_067885.** **AGGGATTCCCTGATATCATATCATATTAATATATTGACAATATTCAAAAAATAATATAAAATGTATAAAA** 596

**Gymnema yunnanense NC_079598.1** **----------------------------------------------------------------------** 518

**Hoya ariadna NC_069568.1**  **AGAGATTCCCTGATATGATAT-----TAATATATTGACAGTATTCATAAAATAATGTATAATGTATAAAA** 634

**Hoya commutata NC_067958.1**  **------------------------------------------------------TGTATAATGTATAAAA** 527

**Hoya dimorpha NC_067959.1**  **AGGGATTCCCTGATATCATAT-----TAATATATTGACAGTATTCATAAAATAATGTATAATGTATAAAA** 631

**Hoya exilis MW719054.1**  **AGAGATTCCCTGATATCATATCATATTAATATATTGACAGTATTCAAAAAATAATATATAATGTATAAAA** 647

**Hoya griffithii NC_069565.1**  **AGGGATTCTCTGATATCATATCATATTAATATATTGACAATATTCAAAAAATAATGTATAATGTATAAAA** 613

**Hoya kerrii NC_069570.1**  **---------------------------------------------------TGT----------ATAAAA** 540

**Hoya lacunosa NC_069564.1**  **AGAGATTCCCTGATATGATAT-ATAT---------------------------ATATATAGTGTGTA---** 587

**Hoya lanceolata NC_067960.1**  **AGGGATTCCCTGATATCA----------------------------------------------------** 490

**Hoya liangii OL826865.1**  **AGGGATTCTCTGATATCATATCATATTAATATATTGACAATATTCAAAAAATAATGTATAATGTATAAAA** 622

**Hoya lithophytica MW719058.1**  **AGAGATTCCCTGATATGAATATGATATTAATATATTGACAGTATTCATAAAATAATGTATAATGTATAAA** 713

**Hoya lockii OR475243.1**  **AGGGATTCCCTGATATCATATCATATTAATATATTGACAGTATTCAAAAAATAATATATAATGTATAAAA** 649

**Hoya longifolia NC_069560.1**  **AGGGATTCTCTGATATCATATCATATTAATATATTGACAATATTCAAAAAATAATGTATAATGTATAAAA** 613

**Hoya lyi MW719055.1**  **CTGATATCAT--------------ATTAATATATTGACAATATTCAAAAAATAATGTATAATGTATAAAA** 617

**Hoya megalaster MW719063.1**  **AGAGATTCCCTGATATCATAT-----TAATATATTGACAACATTCAAAAAATAATGTATAATGTATAAAA** 2139

**Hoya meliflua NC_069571.1**  **CTGATATCAT--------------ATTAATATATTGACAATCTTCAAAAAATAATGTATAATGTATAAAA** 609

**Hoya monetteae MW719053.1**  **AGGGATTCTCTGATATCAT-----ATTAATATATTGACAATATTCAAAAAATAATGTATAATGTATAAA-** 607

**Hoya omlorii MW719060.1**  **AGAGATTCCCTGATATGATAT-----TAATATATTGACAGTATTCATAAAATAATGTATAATGTATAAAA** 2141

**Hoya ovalifolia NC_069563.1**  **CTGATATCAT--------------ATTAATATATTGACATTATTCAAAAAATAATGTATAATGTATAAAA** 614

**Hoya pandurata NC_069562.1**  **---------------------------------------------------TATAATGTATAAAATGTAT** 545

**Hoya pottsii OL754664.1**  **-----------------------------------------------------ATGTATAATGTATAAAA** 528

**Hoya pubicalyx NC_069561.1**  **AGGGATTCTCTGATATCAT-----ATTAATATATTGACAATATTCAAAAAATAATGTATAATGTATAAA-** 604

**Hoya radicalis NC_067961.1**  **CTGATATCATATCATATTAATATATTGACAATATTCAAAAAATAATGTATAATGTATAAAATGTATAAAA** 622

**Hoya rigida NC_067962.1**  **CTGATATCA-----TATTAATATATTGACAATATTCAAAAAATAATGTTTAATGTATAAAATGTATAAAA** 603

**Hoya silvatica NC_067963.1**  **-----------------------------------------------------ATGTATAATGTATAAAA** 528

**Hoya thomsonii NC_067612.1**  **---------------------------------------------------TGTATAAAATGT-ATAAAA** 595

**Hoya verticillata NC_085236.1**  **CTGATATCAT--------------ATTAATATATTGACATTATTCAAAAAATAATGTATAATGTATAAAA** 603

**Papuahoya urniflora MW719062.1** **AGAGATTCCCTGATATGATAT-----TAATATATTGACAGTATTCAAAAAATAATGTATAATGTATAAAA** 610

**Stephanotis volubilis OP133576** **----------------------------------------------------------------------** 498

**2250 2260 2270 2280 2290 2300 2310**

**....|....|....|....|....|....|....|....|....|....|....|....|....|....|**

**Dischidia australis NC_067885.** **----------------------------------------------------------------------** 596

**Gymnema yunnanense NC_079598.1** **----------------------------------------------------------------------** 518

**Hoya ariadna NC_069568.1**  **----------------------------------------------------------------------** 634

**Hoya commutata NC_067958.1**  **----------------------------------------------------------------------** 527

**Hoya dimorpha NC_067959.1**  **----------------------------------------------------------------------** 631

**Hoya exilis MW719054.1**  **TGTATAAAA-------------------------------------------------------------** 656

**Hoya griffithii NC_069565.1**  **TGTATAAAA-------------------------------------------------------------** 622

**Hoya kerrii NC_069570.1**  **----------------------------------------------------------------------** 540

**Hoya lacunosa NC_069564.1**  **----------------------------------------------------------------------** 587

**Hoya lanceolata NC_067960.1**  **--------------------------------------------TATCATATTAATATATTGACAGTATT** 516

**Hoya liangii OL826865.1**  **TGTATAAAA-------------------------------------------------------------** 631

**Hoya lithophytica MW719058.1**  **AATAATGACAAGAGATTCCCTGATATGAATATGATATATATGAATATGATATTAATATATTGACAGTATT** 783

**Hoya lockii OR475243.1**  **TATAAAATAA------------------------------------------------------------** 659

**Hoya longifolia NC_069560.1**  **TGTATAAAA-------------------------------------------------------------** 622

**Hoya lyi MW719055.1**  **----------------------------------------------------------------------** 617

**Hoya megalaster MW719063.1**  **ATAATGACAAGAG-------------------------------ATTCCCTGATATGATATTAATATATT** 2178

**Hoya meliflua NC_069571.1**  **----------------------------------------------------------------------** 609

**Hoya monetteae MW719053.1**  **--------A-------------------------------------------------------------** 608

**Hoya omlorii MW719060.1**  **ATAATGACAAGAG-------------------------------ATTCCCTGATATGATATTAATATATT** 2180

**Hoya ovalifolia NC_069563.1**  **----------------------------------------------------------------------** 614

**Hoya pandurata NC_069562.1**  **----------------------------------------------------------------------** 545

**Hoya pottsii OL754664.1**  **----------------------------------------------------------------------** 528

**Hoya pubicalyx NC_069561.1**  **--------A-------------------------------------------------------------** 605

**Hoya radicalis NC_067961.1**  **----------------------------------------------------------------------** 622

**Hoya rigida NC_067962.1**  **----------------------------------------------------------------------** 603

**Hoya silvatica NC_067963.1**  **----------------------------------------------------------------------** 528

**Hoya thomsonii NC_067612.1**  **----------------------------------------------------------------------** 595

**Hoya verticillata NC_085236.1**  **----------------------------------------------------------------------** 603

**Papuahoya urniflora MW719062.1** **TGTATAAAA-------------------------------------------------------------** 619

**Stephanotis volubilis OP133576** **----------------------------------------------------------------------** 498

**2320 2330 2340 2350 2360 2370 2380**

**....|....|....|....|....|....|....|....|....|....|....|....|....|....|**

**Dischidia australis NC_067885.** **----------------------ATAATG-------------ACAAGAGATTCCCTGATATGATATTAATA** 631

**Gymnema yunnanense NC_079598.1** **------------------------------------------------------------------CATA** 522

**Hoya ariadna NC_069568.1**  **----------------------ATAATG-------------ACAAGAGATTCCCTGATATGATATTAATA** 669

**Hoya commutata NC_067958.1**  **----------------------ATAATG-------------ACAAGAGATTCCCTGATATGATATATATA** 562

**Hoya dimorpha NC_067959.1**  **----------------------ATAATG-------------ACAAGAGATTCCCTGATATGATATTAATA** 666

**Hoya exilis MW719054.1**  **----------------------AAAATG-------------ACAAGAGATTCCCTGATATGATATTAATA** 691

**Hoya griffithii NC_069565.1**  **----------------------ATAATG-------------ACAAGAGATTCCCCGATATGATATATATA** 657

**Hoya kerrii NC_069570.1**  **----------------------ATAATG-------------ACAATAGATTCCCTGATATGATATATATA** 575

**Hoya lacunosa NC_069564.1**  **--------------------------------------------------------------TAGTCATA** 595

**Hoya lanceolata NC_067960.1**  **CAAAAAATAATCTATAATGTATAAAATGTATAAAAATAATGACAAGAGATTCCCTGATATGATATTAATA** 586

**Hoya liangii OL826865.1**  **----------------------ATAATG-------------ACAAGAGATTCCCCGATATGATATATATA** 666

**Hoya lithophytica MW719058.1**  **CATAAAATAATGTATAATGTATAAAATGTATAAAAATAATGACAAGAGATTCCCTGATATGATATTAATA** 853

**Hoya lockii OR475243.1**  **-----------------AATATAAAATGT------------ATAAAAAACTCCCTGATATGATATTAATA** 700

**Hoya longifolia NC_069560.1**  **----------------------ATAATG-------------ACAAGAGATTCCCCGATATGATATATATA** 657

**Hoya lyi MW719055.1**  **----------------------ATAATG-------------ACAAGAGATTCCCTGATATGATATATATA** 652

**Hoya megalaster MW719063.1**  **GACAACATTCAAAAAATAATGTATAATGTATAAAAATAATGACAAGAGATTCCCTGATATGATATTAATA** 2248

**Hoya meliflua NC_069571.1**  **----------------------AAAATG-------------ACAAGAGATTCCCTGATATGATATATATA** 644

**Hoya monetteae MW719053.1**  **----------------------ATAATG-------------ACAAGAGATTCCCTGATATGATATATATA** 643

**Hoya omlorii MW719060.1**  **GACAGTATTCATAAAATAATGTATAATGTATAAAAATAATGACAAGAGATTCCCTGATATGATATTAATA** 2250

**Hoya ovalifolia NC_069563.1**  **----------------------ATAATG-------------ACAAGAGATTCCCTGATATGATATATATA** 649

**Hoya pandurata NC_069562.1**  **----------------------AAAAAT-------------AATGACAAGAGATTCCCTGATATGATATA** 580

**Hoya pottsii OL754664.1**  **----------------------ATAATG-------------ACAAGAGATTCCCTGATATGATATATATA** 563

**Hoya pubicalyx NC_069561.1**  **----------------------ATAATG-------------ACAAGAGATTCCCTGATATGATATATATA** 640

**Hoya radicalis NC_067961.1**  **----------------------ATAATG-------------ACAAGAGATTCCCTGATATGATATATATA** 657

**Hoya rigida NC_067962.1**  **----------------------AAAATG-------------ACAAGAGATTCCCGGATATGATATATATA** 638

**Hoya silvatica NC_067963.1**  **----------------------ATAATG-------------ACAAGAGATTCCCTGATATGATATATATA** 563

**Hoya thomsonii NC_067612.1**  **----------------------ATAATG-------------ACAAGAGATTCCCTGATATGATATATATA** 630

**Hoya verticillata NC_085236.1**  **----------------------ACAATG-------------ACAAGAGATTCCCTGATATGATATATATA** 638

**Papuahoya urniflora MW719062.1** **----------------------ATAATG-------------ACAAGAGATTCCCTGATATGATATTAATA** 654

**Stephanotis volubilis OP133576** **------------------------------------------------------------------CATA** 502

**2390 2400 2410 2420 2430 2440 2450**

**....|....|....|....|....|....|....|....|....|....|....|....|....|....|**

**Dischidia australis NC_067885.** **TATAGTGTATAGTCATATATAGCGTATAGTGAAGTATTACTCCGTATTTTCAAAAAA-GAGCATTTTTTT** 700

**Gymnema yunnanense NC_079598.1** **TATAGTGTATAGT-----------------GAAGTATTACTCCGGATTTCCAAAAAA-GAGCATTTTTTT** 574

**Hoya ariadna NC_069568.1**  **TATAGTGTATAGTCATATATAGCGTATAGTGAAGTATTACTCCGGATTTTCAAAAAA-GAGCATTTTTTT** 738

**Hoya commutata NC_067958.1**  **TATAGTGTATAGTCATATATATCGTATAGTGAAGTATTACTCCGGATTTTCAAAAAA-GAGCATTTTTTT** 631

**Hoya dimorpha NC_067959.1**  **TATAGTGTATAGTCATATATAGCGTATAGTGAAGTATTACTCCGGATTTTCAAAAAA-GAGCATTTTTTT** 735

**Hoya exilis MW719054.1**  **TATAGTGTATAGTCATATATAGCGTATAGTGAAGTATTACTCCGGATTTAAAAAAAA-GAGCATTTTTTT** 760

**Hoya griffithii NC_069565.1**  **TATAGTGTATAGTCATATATAGCGTATAGTGAAGTATTACTCCGGATTTTCAAAAAA-GAGCATTTTTTT** 726

**Hoya kerrii NC_069570.1**  **TATAGTGTATAGTCATATATATCGTATAGTGAAGTATTACTCCGGATTTTCAAAAAA-GAGCATTTTTTT** 644

**Hoya lacunosa NC_069564.1**  **TATATCGTATAGTCATATATATCGTATAGTGAAGTATTACTCCGGATTTAAAAAAAA-GAGCATTTTTTT** 664

**Hoya lanceolata NC_067960.1**  **TATAGTGTATAGTCATATATAGCGTATAGTGAAGTATTACTCCGAATTTTCAAAAAA-GAGCATTTTTTT** 655

**Hoya liangii OL826865.1**  **TATAGTGTATAGTCATATATAGCGTATAGTGAAGTATTACTCCGGATTTTCAAAAAA-GAGCATTTTTTT** 735

**Hoya lithophytica MW719058.1**  **TATAGTGTATAGTCATATATAGCGTATAGTGAAGTATTACTCCGGATTTTCAAAAAA-GAGCATTTTTTT** 922

**Hoya lockii OR475243.1**  **TATAGTGTATAGTCATATATAGCGTATAGTGAAGTATTACTCCGGATTTTCAAAAAA-GAGCATTTTTTT** 769

**Hoya longifolia NC_069560.1**  **TATAGTGTATAGTCATATATAGCGTATAGTGAAGTATTACTCCGGATTTTCAAAAAA-GAGCATTTTTTT** 726

**Hoya lyi MW719055.1**  **TATAGTGTATAGTCATATATATCGTATAGTGAAGTATTACTGCGGATTTTCAAAAAA-GAGCATTTTTTT** 721

**Hoya megalaster MW719063.1**  **TATWGTGTATAGTCATATATAGCGTATAGTGAAGTATTACTCCGGATTTTCAAAAAA-GAGCATTTTTTT** 2317

**Hoya meliflua NC_069571.1**  **TATAGTGTATAGTCATATATATCGTATAGTGAAGTATTACTCCGGATTTTCAAAAAA-GAGCATTTTTTT** 713

**Hoya monetteae MW719053.1**  **TATAGTGTATAGTCATATATATCGTATAGTGAAGTATTACTCCGGATTTTCAAAAAA-GAGCATTTTTTT** 712

**Hoya omlorii MW719060.1**  **TATAGTGTATAGTCATATATAGCGTATAGTGAAGTATTACTCCGGATTTTCAAAAAA-GAGCATTTTTTT** 2319

**Hoya ovalifolia NC_069563.1**  **TATAGTGTATAGTCATATATATCGTATAGTGAAGTATTACTCCGGATTTTCAAAAAA-GAGCATTTTTTT** 718

**Hoya pandurata NC_069562.1**  **TATAGTGTATAGTCATATATATCGTATAGTGAAGTATTACTCCGGATTTTAAAAAAAAGAGCATTTTTTT** 650

**Hoya pottsii OL754664.1**  **TATAGTGTATAGTCATATATATCGTATAGTGAAGTATTACTCCGGATTTTCAAAAAA-GAGCATTTTTTT** 632

**Hoya pubicalyx NC_069561.1**  **----GTGTATAGTCATATATATCGTATAGTGAAGTATTACTCCGGATTTTTAAAAAA-GAGCATTTTTTT** 705

**Hoya radicalis NC_067961.1**  **TATAGTGTATAGTCATATATATCGTATAGTGAAGTATTACTCCGGATTTTCAAAAAA-GAGCATTTTTTT** 726

**Hoya rigida NC_067962.1**  **TATAGTGTATAGTCATATATATCGTATAGTGAAGTATTACTCCGGATTTTCAAAAAA-GAGCATTTTTTT** 707

**Hoya silvatica NC_067963.1**  **TATAGTGTATAGTCATATATATCGTATAGTGAAGTATTACTCCGGATTTTCAAAAAA-GAGCATTTTTTT** 632

**Hoya thomsonii NC_067612.1**  **TATAGTGTATAGTCATATATATCGTATAGTGAAGTATTACTCCGGATTTTCAAAAAA-GAGCATTTTTTT** 699

**Hoya verticillata NC_085236.1**  **TATAGTGTATAGTCATATATATCGTATAGTGAAGTATTACTCCGGATTTTCAAAAAAAGAGCATTTTTTT** 708

**Papuahoya urniflora MW719062.1** **TATAGTGTATAGTCATATATAGCGTATAGTGAAGTATTACTCCGGATTTTCAAAAAA-GAGCATTTTTTT** 723

**Stephanotis volubilis OP133576** **TATAGTGTATAGTCATATATAGTGTATAGTGAAGTATTACTCCGGATTTCCAAAAAA-GAGCATTTTTT-** 570

**2460 2470 2480 2490 2500 2510 2520**

**....|....|....|....|....|....|....|....|....|....|....|....|....|....|**

**Dischidia australis NC_067885.** **-----CAATAATCACACCTAATACCTTCTTATTGTT--TTTTATTGGTTATCTT-------------TTT** 750

**Gymnema yunnanense NC_079598.1** **-----CAATACTCACACCTATTACCTTTTTCTTATTTTTCTTATTAGTTAATA-----------------** 622

**Hoya ariadna NC_069568.1**  **-----CAATAATGACACCTAATACCTTCTTATTGTT--TTTTATTGGTTATCTT-------------ATT** 788

**Hoya commutata NC_067958.1**  **-----CAATAATCACACCTAATACCTTCTTATTGTT--TCTTATTGGTTAACTT-------------ATT** 681

**Hoya dimorpha NC_067959.1**  **-----CAATAATCACACCTAATACCTTCTTATTGTT--TCTTATTGGTTATCTT-------------ATT** 785

**Hoya exilis MW719054.1**  **-----CAATAATCACACCTAATACCTTCTTATTCTT--TCTTATTGGTTTTATT-------------ATT** 810

**Hoya griffithii NC_069565.1**  **-----CAATAATCACACCTAATACCTTCTTATTGTT--TCTTATTGGTTAACTT-------------ATT** 776

**Hoya kerrii NC_069570.1**  **-----CAATAATCACACCTAATACCTTCTTATTGTT--TCTTATTGGTTAACTT-------------ATT** 694

**Hoya lacunosa NC_069564.1**  **-----CAATAATCACACCTAATACCTTCTTATTGTT--TCTTATTGGTTAACTT-------------ATT** 714

**Hoya lanceolata NC_067960.1**  **-----CAATAATCACACCTAATACCTTCTTATT---------ATTAGTTAA------------------T** 693

**Hoya liangii OL826865.1**  **-----CAATAATCACACCTAATACCTTCTTATTGTT--TCTTATTGGTTAACTT-------------ATT** 785

**Hoya lithophytica MW719058.1**  **-----CAATAATGACACCTAATACCTTCTTATTGTT--TTTTATTGGTTATCTTAT-------------T** 972

**Hoya lockii OR475243.1**  **TTTTTCAATAATCACACCTAATACCTTCTTATTGTT--TCTTATTGGTTATCTT-------------ATT** 824

**Hoya longifolia NC_069560.1**  **-----CAATAATCACACCTAATACCTTCTTATTGTT--TCTTATTGGTTAACTT-------------ATT** 776

**Hoya lyi MW719055.1**  **-----CAATAATCACACCTAATACCTTCTTATTGTT--TCTTATTGGTTAACTT-------------ATT** 771

**Hoya megalaster MW719063.1**  **-----CAATAATCACACCTAATACCTTCTTATTGTT--TCTTATTGGTTATCTT-------------ATT** 2367

**Hoya meliflua NC_069571.1**  **-----AAATAATCACACCTAATACCTTCTTATTGTT--TCTTATTGGTTAACTT-------------ATT** 763

**Hoya monetteae MW719053.1**  **-----CAATAATCACACCTAATACCTTCTTATTGTT--TCTTATTGGTTAACTT-------------ATT** 762

**Hoya omlorii MW719060.1**  **-----CAATAATGACACCTAATACCTTCTTATTGTT--TTTTATTGGTTATCTT-------------ATT** 2369

**Hoya ovalifolia NC_069563.1**  **-----CAATAATCACACCTAATACCTTCTTATTGTT--TCTTATTGGTTAACTT-------------ATT** 768

**Hoya pandurata NC_069562.1**  **-----CAATAATCACACCTAATACCTTCTTATTGTT--TCTTATTGGTTAACTT-------------ATT** 700

**Hoya pottsii OL754664.1**  **-----CAATAATCACACCTAATACCTTCTTATTGTT--TCTTATTGGTTAACTT-------------ATT** 682

**Hoya pubicalyx NC_069561.1**  **-----CAATAATCACACCTAATACCTTCTTATTGTT--TCTTATTGGTTAACTT-------------ATT** 755

**Hoya radicalis NC_067961.1**  **-----CAATAATCACACCTAATACCTTCTTATTGTT--TCTTATTGGTTAACTT-------------ATT** 776

**Hoya rigida NC_067962.1**  **-----CAATAATCACACCTAATACCTTCTTATTGTT--TCTTATTGGTTAACTT-------------ATT** 757

**Hoya silvatica NC_067963.1**  **-----CAATAATCACACCTAATACCTTCTTATTGTT--TCTTATTGGTTAACTT-------------ATT** 682

**Hoya thomsonii NC_067612.1**  **-----CAATAATCACACCTAATACCTTCTTATTGTT--TCTTATTGGTTAACTT-------------ATT** 749

**Hoya verticillata NC_085236.1**  **-----CAATAATCACACCTAATACCTTCTTATTGTT--TCTTATTGGTTAACTT-------------ATT** 758

**Papuahoya urniflora MW719062.1** **-----CAATAATCACACCTAATACCTTCTTATTGCT--TCTTATTGGTTATCTT-------------ATT** 773

**Stephanotis volubilis OP133576** **-----CAATACTCACACCTATTACCTTTTTCTTATTTTTCTTATTAGTTAATTTTCTTATTTTTCTTATT** 635

**2530 2540 2550 2560 2570 2580 2590**

**....|....|....|....|....|....|....|....|....|....|....|....|....|....|**

**Dischidia australis NC_067885.** **AGTTACTAAAAAATGCTAGC----------------------GATTGG---------TTTAGTCTAATAG** 789

**Gymnema yunnanense NC_079598.1** **-------AAAAAATGCTAGC----------------------GATTGGGTTTCTATGTTTAGTCTAATAG** 663

**Hoya ariadna NC_069568.1**  **AGTTAATAAAAAATGCTAGC----------------------GATTGG---------TTTAGTCTAATAG** 827

**Hoya commutata NC_067958.1**  **AGTTAATAAAAAATGCTAGC----------------------GATTGG---------TTTAGTCTAATAG** 720

**Hoya dimorpha NC_067959.1**  **AGTTAATCAAAAATGCTAGC----------------------GATTGG---------TTTAGTCTAATAG** 824

**Hoya exilis MW719054.1**  **AGTTAATAAAAAATGCTAGC----------------------GATTGG---------TTTAGTCTAATAG** 849

**Hoya griffithii NC_069565.1**  **AGTTAATAAAAAATGCTAGC----------------------GATTGG---------TTTAGTCTAATAG** 815

**Hoya kerrii NC_069570.1**  **AGTTAATAAAAAATGCTAGC----------------------GATTGG---------TTTAGTCTAATAG** 733

**Hoya lacunosa NC_069564.1**  **AGTTAATAAAAAATGCTAGC----------------------GATTGG---------TTTAGTCTAATAG** 753

**Hoya lanceolata NC_067960.1**  **AAATAATAAAGAATGCTAGT----------------------AATTGG---------TTTAGTCTAATAG** 732

**Hoya liangii OL826865.1**  **AGTTAATAAAAAATGCTAGC----------------------GATTGG---------TTTAGTCTAATAG** 824

**Hoya lithophytica MW719058.1**  **AGTTAATAAAAAATGCTAGCGATTGGTTTAGTCTAATAGGAAAATTGG---------TTTAGTCTAATAG** 1033

**Hoya lockii OR475243.1**  **AGTTAATAAAAAATGCTAGC----------------------GATTGG---------TTTAGTCTAATAG** 863

**Hoya longifolia NC_069560.1**  **AGTTAATAAAAAATGCTAGC----------------------GATTGG---------TTTAGTCTAATAG** 815

**Hoya lyi MW719055.1**  **AGTTAATAAAAAATGCTAGC----------------------GATTGG---------TTTAGTCTAATAG** 810

**Hoya megalaster MW719063.1**  **AGTTAATCAAAAATGCTAGC----------------------GATTGG---------TTTAGTCTAATAG** 2406

**Hoya meliflua NC_069571.1**  **AGTTAATAAAAAATGCTAGC----------------------GATTGG---------TTTAGTCTAATAG** 802

**Hoya monetteae MW719053.1**  **AGTTAATAAAAAATGCTAGC----------------------GATTGG---------TTTAGTCTAATAG** 801

**Hoya omlorii MW719060.1**  **AGTTAATAAAAAATGCTAGC----------------------GATTGG---------TTTAGTCTAATAG** 2408

**Hoya ovalifolia NC_069563.1**  **AGTTAATAAAAAATGCTAGC----------------------GATTGG---------TTTAGTCTAATAG** 807

**Hoya pandurata NC_069562.1**  **AGTTAATAAAAAATGCTAGC----------------------GATTGG---------TTTAGTCTAATAG** 739

**Hoya pottsii OL754664.1**  **AGTTAATAAAAAATGCTAGC----------------------GATTGG---------TTTAGTCTAATAG** 721

**Hoya pubicalyx NC_069561.1**  **AGTTAATAAAAAATGCTAGC----------------------GATTGG---------TTTAGTCTAATAG** 794

**Hoya radicalis NC_067961.1**  **AGTTAATAAAAAATGCTAGC----------------------GATTGG---------TTTAGTCTAATAG** 815

**Hoya rigida NC_067962.1**  **AGTTAATAAAAAATGCTAGC----------------------GATTGG---------TTTAGTCTAATAG** 796

**Hoya silvatica NC_067963.1**  **AGTTAATAAAAAATGCTAGC----------------------GATTGG---------TTTAGTCTAATAG** 721

**Hoya thomsonii NC_067612.1**  **AGTTAATAAAAAATGCTAGC----------------------GATTGG---------TTTAGTCTAATAG** 788

**Hoya verticillata NC_085236.1**  **AGTTAATAAAAAATGCTAGC----------------------GATTGG---------TTTAGTCTAATAG** 797

**Papuahoya urniflora MW719062.1** **AGTTAATAAAAAATGCTAGC----------------------GATTGG---------TTTAGTCTAATAG** 812

**Stephanotis volubilis OP133576** **AGTTAATAAAAAATGCTAGC----------------------GATTGGGTTTCTATGTTTAGTCTAATAG** 683

**2600 2610 2620 2630 2640 2650 2660**

**....|....|....|....|....|....|....|....|....|....|....|....|....|....|**

**Dischidia australis NC_067885.** **GAAAGAAGATATTCAAATAAATAA-----------------------------------------T----** 814

**Gymnema yunnanense NC_079598.1** **GAAAGAAGATATTCAAATAAAGAA-----------------------------------------T----** 688

**Hoya ariadna NC_069568.1**  **GAAAGAAGATATTCAAATAAAGAA-----------------------------------------T----** 852

**Hoya commutata NC_067958.1**  **GAAAGAAGATATTCAAATAAAGAA-----------------------------------------T----** 745

**Hoya dimorpha NC_067959.1**  **GAAAGAAGATATTCAAATAAATAA-----------------------------------------T----** 849

**Hoya exilis MW719054.1**  **GAAAGAAGATATTCAAATAAAGAA-----------------------------------------T----** 874

**Hoya griffithii NC_069565.1**  **GAAAGAAGATATTCAAATAAAGAA-----------------------------------------T----** 840

**Hoya kerrii NC_069570.1**  **GAAAGAAGATATTCAAATAAAGAA-----------------------------------------T----** 758

**Hoya lacunosa NC_069564.1**  **GAAAGAAGATATTCAAATAAAGAA-----------------------------------------T----** 778

**Hoya lanceolata NC_067960.1**  **GAAAGAAGATATTCAAATAAGGAATTTT---------------------------------------ATA** 763

**Hoya liangii OL826865.1**  **GAAAGAAGATATTCAAATAAAGAA-----------------------------------------T----** 849

**Hoya lithophytica MW719058.1**  **GAAAGAAGATATTCAAATAAAGAATTTTTGCAAATAAAGAATTTTTGCAAATAAAGAATTTTTGCAAATA** 1103

**Hoya lockii OR475243.1**  **GAAAGAAGATATTCAAATAAAGAA-----------------------------------------T----** 888

**Hoya longifolia NC_069560.1**  **GAAAGAAGATATTCAAATAAAGAA-----------------------------------------T----** 840

**Hoya lyi MW719055.1**  **GAAAGAAGATATTCAAATAAAGAA-----------------------------------------T----** 835

**Hoya megalaster MW719063.1**  **GAAAGAAGATATTCAAATAACGAA-----------------------------------------T----** 2431

**Hoya meliflua NC_069571.1**  **GAAAGAAGATATTCAAATAAAGAA-----------------------------------------T----** 827

**Hoya monetteae MW719053.1**  **GAAAGAAGATATTCAAATAAAGAA-----------------------------------------T----** 826

**Hoya omlorii MW719060.1**  **GAAAGAAGATATTCAAATAAAGAA-----------------------------------------T----** 2433

**Hoya ovalifolia NC_069563.1**  **GAAAGAAGATATTCAAATAAATAA-----------------------------------------T----** 832

**Hoya pandurata NC_069562.1**  **GAAAGAAGATATTCAAATAAAGAA-----------------------------------------T----** 764

**Hoya pottsii OL754664.1**  **GAAAGAAGATATTCAAATAAAGAA-----------------------------------------T----** 746

**Hoya pubicalyx NC_069561.1**  **GAAAGAAGATATTCAAATAAAGAA-----------------------------------------T----** 819

**Hoya radicalis NC_067961.1**  **GAAAGAAGATATTCAAATAAAGAA-----------------------------------------T----** 840

**Hoya rigida NC_067962.1**  **GAAAGAAGATATTCAAATAAAGAA-----------------------------------------T----** 821

**Hoya silvatica NC_067963.1**  **GAAAGAAGATATTCAAATAAAGAA-----------------------------------------T----** 746

**Hoya thomsonii NC_067612.1**  **GAAAGAAGATATTCAAATAAAGAA-----------------------------------------T----** 813

**Hoya verticillata NC_085236.1**  **GAAAGAAGATATTCAAATAAATAA-----------------------------------------T----** 822

**Papuahoya urniflora MW719062.1** **GAAAGAAGATATTCAAATAAAGAA-----------------------------------------T----** 837

**Stephanotis volubilis OP133576** **GAAAGAAGATATTCAAATAAAGAA-----------------------------------------T----** 708

**2670 2680 2690 2700 2710 2720 2730**

**....|....|....|....|....|....|....|....|....|....|....|....|....|....|**

**Dischidia australis NC_067885.** **------TTTTGATCGA------------------------------------------------------** 824

**Gymnema yunnanense NC_079598.1** **------TTTTGATCGA------------------------------------------------------** 698

**Hoya ariadna NC_069568.1**  **------TTTTGATCGA------------------------------------------------------** 862

**Hoya commutata NC_067958.1**  **------TTTTGATCGA------------------------------------------------------** 755

**Hoya dimorpha NC_067959.1**  **------TTTTGATCGAATGACTATTCATCTATTGTTTTTGTATTTTCTTACAAAACAAAAACAACAAAAC** 913

**Hoya exilis MW719054.1**  **------TTTTGATCGAATGACTATTCATCTATTGTTTTTATATTTTCTTACAAAACAAAAACAACAAAAC** 938

**Hoya griffithii NC_069565.1**  **------TTTTGATCGA------------------------------------------------------** 850

**Hoya kerrii NC_069570.1**  **------TTTTGATCGAATGACTATTCATCTATTGTTTTTATATTTTCTTACAAAACAAAAACAAAAAAAC** 822

**Hoya lacunosa NC_069564.1**  **------TTTGGATCGAATGGCTATTCATCTATTGTTTTTATATTTTCTTACAAAACAAAAA-AACAAAA-** 840

**Hoya lanceolata NC_067960.1**  **AAGAATTTTTGATCGAATGACTATTCATCTATTGTTTTTAGATTTTATTACAAAA---------------** 818

**Hoya liangii OL826865.1**  **------TTTTGATCGA------------------------------------------------------** 859

**Hoya lithophytica MW719058.1**  **AAGAATTTTTGATCGAATGACTATTCATCTATTGTTTTTATATTTTATTACAAAACAAAAACAACAAAAC** 1173

**Hoya lockii OR475243.1**  **------TTTTGATCAA------------------------------------------------------** 898

**Hoya longifolia NC_069560.1**  **------TTTTGATCGA------------------------------------------------------** 850

**Hoya lyi MW719055.1**  **------TTTTGATCGAATGACTATTCATCTATTGTTTTTATATTTTCTTACAAAACAAAAACAAAAAAAA** 899

**Hoya megalaster MW719063.1**  **------TTTTGATCGAATGACTATTCATCTATTGTTTTTATATTTTCTTACAAAACAAAAACAACAAAAC** 2495

**Hoya meliflua NC_069571.1**  **------TTTTGATCGAATGACTATTCATCTATTGTTTTTATATTTTCTTACAAAACAAAAACAAAAAAAC** 891

**Hoya monetteae MW719053.1**  **------TTTTGATCGA------------------------------------------------------** 836

**Hoya omlorii MW719060.1**  **------TTTTGATCGAATGACTATTCATCTATTGTTTTTAGATTTTCTTACAAAACAAAAACAACAAAAC** 2497

**Hoya ovalifolia NC_069563.1**  **------TTTTGATCGAATGACTATTCATCTATTGTTTTTATATTTTCTTACAAAACAAAAA------AAC** 890

**Hoya pandurata NC_069562.1**  **------TTTTGATCGAATGACTATTCATCTATTGTTTTTATATTTTCTTACAAAACAAAAACAACAAAAC** 828

**Hoya pottsii OL754664.1**  **------TTTTGATCGAATGACTATTCATCTATTGTTTTTATATTTTCTTACAAAACAAAAA---------** 801

**Hoya pubicalyx NC_069561.1**  **------TTTTGATCGA------------------------------------------------------** 829

**Hoya radicalis NC_067961.1**  **------TTTTGATCGAATGACTATTCATCTATTGTTTTTATATTTTCTTACAAAACAAAAACAACAAAAC** 904

**Hoya rigida NC_067962.1**  **------TTTTGATCGAATGACTATTCATCTATTGTTTTTATATTTTCTTACAAAACAAAAACAACAAAAC** 885

**Hoya silvatica NC_067963.1**  **------TTTTGATCGAATGACTATTCATCTATTGTTTTTATATTTTCTTACAAAACAAAAA---------** 801

**Hoya thomsonii NC_067612.1**  **------TTTTGATCGAATGACTATTCATCTATTGTTTTTATATTTTCTTACAAAACAAAAACAACAAAAC** 877

**Hoya verticillata NC_085236.1**  **------TTTTGATCGAATGACTATTCATCTATTGTTTTTATATTTTCTTACAAAACAAAAA------AAC** 880

**Papuahoya urniflora MW719062.1** **------TTTTGATCGAATGACTATTCATCTATTGTTTTTATATTTTCTTACAAAACAAAAACAACAAAAC** 901

**Stephanotis volubilis OP133576** **------TTTTGATCGA------------------------------------------------------** 718

**2740 2750 2760**

**....|....|....|....|....|....|**
[truncated: 79,212 more chars]
